# Bioinspired tetraamino-bisthiourea chiral macrocycles in catalyzing decarboxylative Mannich reactions

**DOI:** 10.3762/bjoc.18.51

**Published:** 2022-05-02

**Authors:** Hao Guo, Yu-Fei Ao, De-Xian Wang, Qi-Qiang Wang

**Affiliations:** 1 Beijing National Laboratory for Molecular Sciences, CAS Key Laboratory of Molecular Recognition and Function, Institute of Chemistry, Chinese Academy of Sciences, Beijing 100190, China; 2 University of Chinese Academy of Sciences, Beijing 100049, China

**Keywords:** chiral macrocycles, cooperative asymmetric catalysis, decarboxylative Mannich reactions, supramolecular catalysis, thiourea

## Abstract

A series of tetraamino-bisthiourea chiral macrocycles containing two diarylthiourea and two chiral diamine units were synthesized by a fragment-coupling approach in high yields. Different chiral diamine units, including cyclohexanediamines and diphenylethanediamines were readily incorporated by both homo and hetero [1 + 1] macrocyclic condensation of bisamine and bisisothiocyanate fragments. With the easy synthesis, gram-scale of macrocycle products can be readily obtained. These chiral macrocycles were applied in catalyzing bioinspired decarboxylative Mannich reactions. Only 5 mol % of the optimal macrocycle catalyst efficiently catalyzed the decarboxylative addition of a broad scope of malonic acid half thioesters to isatin-derived ketimines with excellent yields and good enantioselectivity. The rigid macrocyclic framework and the cooperation between the thiourea and tertiary amine sites were found to be crucial for achieving efficient activation and stereocontrol. As shown in control experiments, catalysis with the acyclic analogues having the same structural motifs were non-selective.

## Introduction

In the past decades, the development of supramolecular chemistry has enabled abundant host scaffolds and assembly tools for boosting catalytic processes, and stimulated the emergence of supramolecular catalysis [1–14]. Among which, macrocyclic compounds have attracted extensive attentions due to their enzyme-mimicking cavity and preorganized binding sites [4,6,15–16]. Various macrocyclic compounds including the privileged scaffolds like cyclodextrins [17–19], calixarenes [20–23], cucurbiturils [24–25], and cavitands [26–27] have been widely applied. While these conventional macrocycles can usually enable a confinement effect or serve as a supporting scaffold, they do not contain definite catalytic sites in their cyclic skeletons. When required, an additional catalytic functional group was commonly introduced through in-situ, noncovalent inclusion/encapsulation in the cavity or by covalent, post-functionalization of the macrocyclic scaffold. The encapsulated catalytic group could occupy the space for substrate entering, or has a risk to be squeezed out of the cavity under complex catalytic conditions. On the other hand, the covalently pendant catalytic group may reside far away from the center of the cavity.

**Figure 1:**
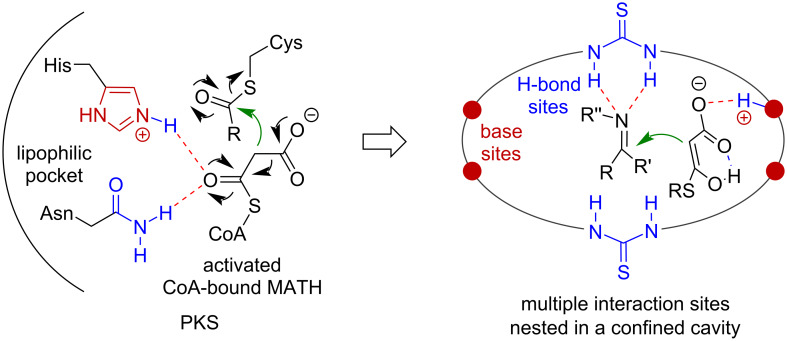
Design of PKS-inspired multifunctional amino-thiourea macrocycle catalysts.

On the other hand, we envisaged by use of tailored building units already containing definite catalytic sites to directly form a macrocycle scaffold could provide a different situation. In this way the catalytic functionalities are permanently installed within the macrocyclic skeleton by forming a persistent catalytic cavity. Following this idea, we have recently constructed a series of bisthiourea macrocycles [28–30]. Thiourea groups were introduced due to their superior anion binding property and potent electrophilic activation ability [31–36]. To incorporate extra functionality, tertiary amine groups can be also embedded as Lewis base sites for realizing electrophilic/nucleophilic cooperative catalysis [37–39]. For this purpose, one kind of tetraamino-bisthiourea chiral macrocycles were synthesized [30]. When applied in catalyzing the decarboxylative addition of phenyl β-ketoacids to cyclic imines bearing sulfamate heading group, an interesting substrate-induced assembly catalysis mode was uncovered [30]. To expand more applications, herein we report a systematic synthesis of tetraamino-bisthiourea chiral macrocycles and their performance in catalyzing the decarboxylative Mannich addition of malonic acid half thioesters (MAHTs) to isatin-derived ketimines. The macrocycle-enabled hydrogen-bonding activation network and the associated confined cavity could resemble the circumstance of the catalytic triad of Polyketide synthases (PKSs) [40–42] (Figure 1). On the other hand, the organocatalytic asymmetric decarboxylative addition reactions of MAHTs to imines provide an efficient means for accessing valuable chiral β-amino esters [43–52].

## Results and Discussion

### Synthesis of macrocycles

**Scheme 1:**
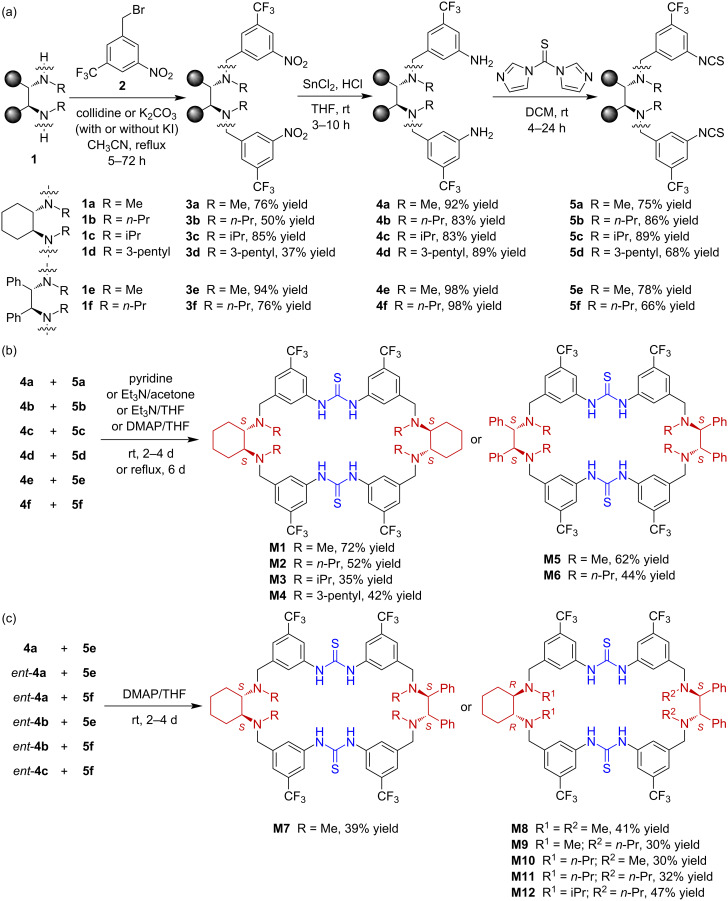
Synthesis of tetraamino-bisthiourea chiral macrocycles **M1**–**M12**. The synthesis of **M1**, **M5**, **M7**, and **M8** was previously reported [30].

The tetraamino-bisthiourea chiral macrocycles were synthesized by a stepwise strategy (Scheme 1). The easily available chiral diamines including 1,2-cyclohexanediamines and 1,2-diphenylethylenediamines were chosen as the linking components to afford Lewis base sites and also for introduction of chirality. Different alkyl substituents including methyl, *n*-propyl, isopropyl, and 3-pentyl were incorporated in order to tune the size and steric effect of the macrocyclic cavity and thus to enable diverse cavity environments. Among these macrocycles, **M1**, **M5**, **M7**, and **M8** were previously synthesized [30] and the route can be similarly followed for the synthesis of the other macrocycles. To start the synthesis, enantiopure *N*,*N’*-disubstituted (*S*,*S*)-1,2-cyclohexanediamines **1a**–**d** or (*S*,*S*)-1,2-diphenylethanediamines **1e**,**f** were firstly reacted with two equiv 3-nitro-5-(trifluoromethyl)benzyl bromide (**2**) in the presence of a base to afford the dinitro compounds **3a**–**f** in moderate to excellent yields (Scheme 1a). The diminished yield for product **3d** was probably caused by the large steric hindrance of the 3-pentyl substituent. Reduction of the nitro groups by SnCl_2_ under acidic conditions gave the bisamine fragments **4a**–**f** in 83–98% yields. The bisamine fragments were further converted to the bisisothiocyanates **5a**–**f** by reaction with 1,1'-thiocarbonyldiimidazole in 66–89% yields.

Having the bisamine and bisisothiocyanate fragments in hand, macrocyclic condensations were then pursued. The homo-condensations between the homologous bisamine and bisisothiocyanate fragments were firstly tried (Scheme 1b). In the presence of an organic base, reactions between **4a**–**f** and **5a**–**f** went smoothly and afforded the desired macrocycle products **M1**–**M6** in 35–72% yields. It is worth noting that common dilute conditions for macrocyclization reactions was not required here. Due to the very high efficiency, gram-scale preparation of the chiral macrocycles was readily achieved (see Supporting Information File 1). To enrich the diversity of the macrocyclic scaffolds, hetero-condensations between different bisamine and bisisothiocyanate fragments, including combination of different chiral configurations, were also investigated (Scheme 1c). Reactions between cyclohexanediamine-derived bisamine fragments **4a** or the enantiomers *ent*-**4a**–**c** with diphenylethylenediamine-derived bisisothiocyanate fragments **5e**,**f** afforded the desired hetero-combination macrocycles **M7**–**M12** without additional difficulties. It should be noted that the incorporation of CF_3_ groups on the aryl moieties was to increase the acidity of thiourea so as to provide better hydrogen-bonding complexation and activation ability.

### Catalytic reaction optimization

The synthesized macrocycles were then applied as catalysts in the decarboxylative addition of malonic acid half thioesters (MAHTs) to isatin-derived ketimines [48]. The reaction between ketimine **6a** and MAHT **7a** was initially performed in THF at room temperature with just 2 mol % loading of the chiral macrocycle catalysts (Table 1). All macrocycles were evaluated, and in all cases product **8a** was obtained in moderate yields. Different diamine linking components and different substituents on the tertiary amine sites showed an important influence on the reaction stereoselectivity. The cyclohexanediamine-linking macrocycles **M1**–**M4** afforded the product with overall higher enantiomeric excess (ee) (Table 1, entries 1–4). Among which the isopropyl-substituted macrocycle **M3** gave the best selectivity, i.e., 42% ee. This suggested a suitable crowding cavity environment may be good for stereocontrol. The diphenylethylenediamine-linking macrocycles **M5** and **M6**, however, gave very low ees (Table 1, entries 5 and 6). The hetero-macrocycles **M7**–**M12** did not afford better selectivity as well (Table 1, entries 7–12). It is interesting to note that **M9**–**M12** led to reversed selectivity, which may imply that the chiral cyclohexanediamine other than diphenylethylenediamine moiety governed the stereoselection process.

**Table 1:** Evaluation of different macrocycle catalysts^a^.

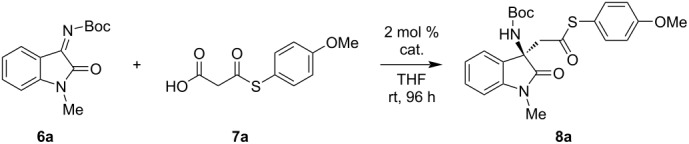			

Entry	Cat.	Yield (%)^b^	ee (%)^c^

1	**M1**	32	9
2	**M2**	43	11
**3**	**M3**	**41**	**42**
4	**M4**	49	29
5	**M5**	48	4
6	**M6**	51	0
7	**M7**	45	13
8	**M8**	39	9
9	**M9**	31	−4
10	**M10**	42	−12
11	**M11**	31	−12
12	**M12**	41	−4

^a^Reaction conditions: **6a** (0.2 mmol), **7a** (0.3 mmol), 1 mL of THF; ^b^isolated yields after column chromatography; ^c^determined by HPLC analysis on a chiral stationary phase.

Using **M3** as the optimal catalyst, the reaction solvent was then screened (Table 2). Ethyl ether was found to give a better conversion, but with decreased selectivity (Table 2, entry 2). The reaction in 1,4-dioxane afforded the product with a moderate yield and the best selectivity so far, 62% ee (Table 2, entry 3). To our delight, among the other ether solvents screened (Table 2, entries 4–7), cyclopentyl methyl ether (CPME) gave an excellent conversion (90% yield) and only a slightly diminished selectivity (58% ee). Reactions in other more polar solvents including toluene, ethyl acetate, halohydrocarbons, and acetonitrile gave overall very good conversions, but with very low selectivity except for the reaction in ethyl acetate which afforded the product in 43% ee (Table 2, entries 8–12).

**Table 2:** Evaluation of solvents^a^.

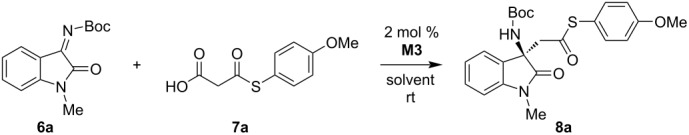				

Entry	Solvent	Time (h)	Yield (%)^b^	ee (%)^c^

1	THF	96	41	42
2	Et_2_O	96	73	34
3	1,4-dioxane	96	52	62
4	TBME	96	79	33
**5**	**CPME**	**96**	**90**	**58**
6	DME	96	47	49
7	EVE	96	39	9
8	toluene	36	96	2
9	EA	84	81	43
10	CH_2_Cl_2_	36	97	0
11	CHCl_3_	36	93	1
12	CH_3_CN	84	71	12

^a^Reaction conditions: **6a** (0.2 mmol), **7a** (0.3 mmol), 1 mL of solvent; ^b^isolated yields after column chromatography; ^c^determined by HPLC analysis on a chiral stationary phase. TBME: *tert*-butyl methyl ether; CPME: cyclopentyl methyl ether; DME: 1,2-dimethoxyethane; EVE: ethyl vinyl ether; EA: ethyl acetate.

Finally, the other reaction parameters, including catalyst loading, reaction temperature, and concentration were evaluated (Table 3). With CPME as the optimal solvent, increasing the loading of catalyst **M3** from 2 mol % to 5 mol % led to an obviously more rapid conversion and furnished the product in 92% yield in 36 h (Table 3, entries 1 and 2). To our delight, the selectivity was also increased to 71% ee. However, further increasing the macrocycle loading to 10 mol % led to a diminished yield and nearly unchanged selectivity (Table 3, entry 3). The decreased yield for the addition product was due to the competitive decarboxylation of the sole MAHT substrate in the presence of a higher loading of the macrocycle containing tertiary amine basic sites. Decreasing the amount of MAHT substrate from 1.5 equiv to 1.0 equiv or increasing to 2.0 equiv did not give better outcome (Table 3, entries 4 and 5). Performing the reaction at 0 °C led to a very slow conversion, while at 40 °C the reaction became much faster but gave a diminished yield due to the competitive decarboxylation side reaction (Table 3, entries 6 and 7). In both cases, the enantioselectivity did not turn out to be better. A suitable reaction concentration (0.1–0.2 M) was found to be important. A very high or low reaction concentration led to decreased stereoselectivity probably due to the existence of catalyst aggregation or background reactions (Table 3, entries 8–10).

**Table 3:** Evaluation of catalyst loading, reaction temperature, and concentration^a^.

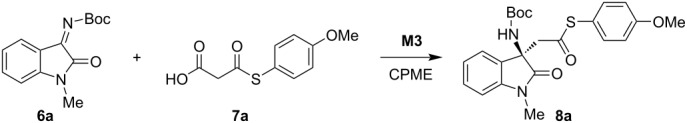						

Entry	Cat. (mol %)	Temp.	Conc. [M]^b^	Time (h)	Yield (%)^c^	ee (%)^d^

1	**M3** (2)	rt	0.2	96	90	58
2	**M3** (5)	rt	0.2	36	92	71
3	**M3** (10)	rt	0.2	24	72	72
4^e^	**M3** (5)	rt	0.2	24	82	72
5^f^	**M3** (5)	rt	0.2	48	94	60
6	**M3** (5)	0 °C	0.2	120	65	63
7	**M3** (5)	40 °C	0.2	12	75	64
8	**M3** (5)	rt	0.4	24	92	56
**9**	**M3 (5)**	**rt**	**0.1**	**44**	**88**	**72**
10	**M3** (5)	rt	0.05	48	54	59

^a^Reaction conditions: **6a** (0.2 mmol) and **7a** (0.3 mmol, 1.5 equiv) in CPME (cyclopentyl methyl ether) except otherwise noted; ^b^concentration of **6a**; ^c^isolated yields after column chromatography; ^d^determined by HPLC analysis on a chiral stationary phase; ^e^1.0 equiv **7a** used; ^f^2.0 equiv **7a** used.

### Substrate scope

**Scheme 2:**
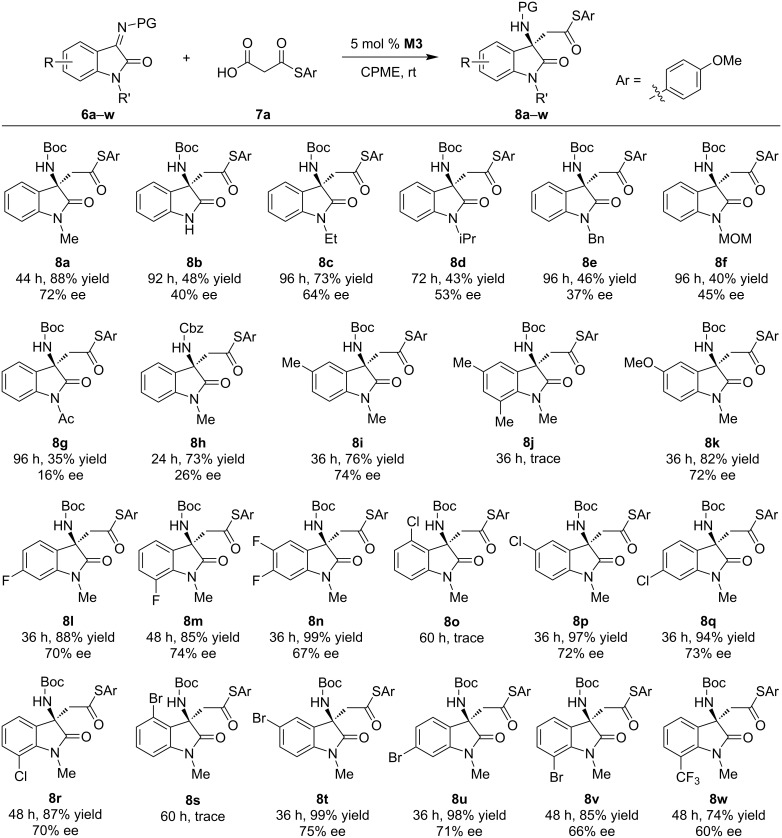
Substrate scope of isatin imines. Reaction conditions: **6** (0.2 mmol), **7a** (0.3 mmol), and 5 mol % **M3** in 2 mL of CPME (cyclopentyl methyl ether).

Having established the optimal reaction conditions, the substrate scope was explored. Reactions of various isatin imines **6a–w** with MAHT **7a** were firstly investigated (Scheme 2). Different *N*-substituents on isatins caused a significant effect. For non-substituted (**6b**) or other substrates with larger substituents (**6c**–**g**), the corresponding products **8b**–**g** were obtained in only moderate yields with decreased selectivity. Replacing the Boc-protecting group on the imine site by a Cbz group led to a largely decreased selectivity (**8h**). For a series of substrates with various substituents on the 5, 6, or 7-position, including 5-methyl (**8i**), 5-methoxy (**8k**), 6- or 7-fluoro (**8l**,**m**), 5,6-difluoro (**8n**), 5-, 6- or 7-chloro (**8p**–**r**), 5-, 6-, or 7-bromo (**8t**–**v**), and 7-trifluoromethyl (**8w**), all reactions completed within 36–48 h and afforded the products in good to excellent yields with 60–75% ee. In contrast, the reaction was disrupted by 4-substitution, and only trace conversion was observed for the 4-chloro or 4-bromo-substituted substrate (**8o**,**s**). This was probably caused by a steric effect as the 4-substituent is close to the imine reactive site and accordingly blocked it toward activation and nucleophilic attack. Interestingly, for the 5,7-dimethyl-substituted substrate, only trace conversion was observed as well (**8j**). This may be due to that the substrate was too bulky to fit within the macrocycle cavity.

**Scheme 3:**
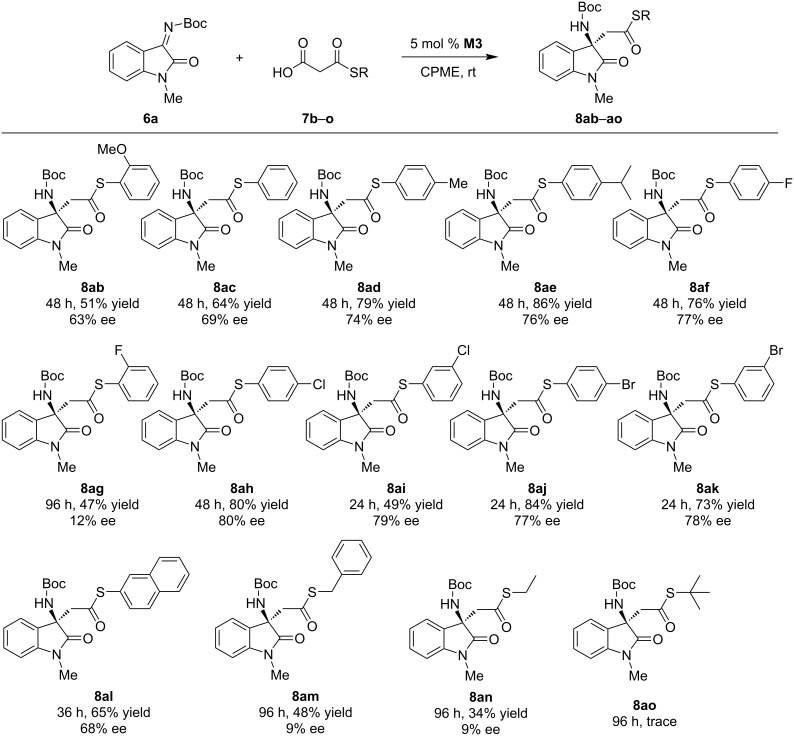
Substrate scope of MAHTs. Reaction conditions: **6a** (0.2 mmol), **7** (0.3 mmol), and 5 mol % **M3** in 2 mL of CPME (cyclopentyl methyl ether).

For MAHT substrates, various *p*- or *m*-substituents on the S-phenyl moiety caused negligible effects, and all the products were obtained in moderate to good yields with 69–80% ee (Scheme 3). For *o*-substitution, especially for the *o*-fluoro-substituent (**8ag**), however, only a moderate yield and low selectivity (12% ee) were obtained. For the S-naphthyl substrate, the reaction went smoothly as well and afforded the product in 65% yield with 68% ee (**8al**). The reactions for S-benzyl and S-ethyl substrates became sluggish and afforded the products in 34–48% yields in 96 h with very low selectivity (**8am** and **8an**). For the S-*tert*-butyl substrate, only trace conversion was observed.

### Macrocyclic effect and catalytic mechanism

The above results showed that the tetraamino-bisthiourea chiral macrocycles can efficiently catalyze the decarboxylative addition reactions with good yields and enantioselectivity. To check the role of the macrocyclic framework, two acyclic compounds (**9** and **3c**) containing the similar structural motifs as the macrocycle catalyst were applied as catalysts in the reaction (Table 4). In compound **9**, all the structural units of the macrocycle **M3**, including the two diarylthioureas and the four tertiary amine sites, were maximally maintained, except for one of the cyclohexanediamine units which was replaced by two dimethylamino groups to cut off the macrocyclic skeleton (for synthesis, see Supporting Information File 1). The compound **3c** contains one cyclohexanediamine unit but no thiourea moieties. As shown in Table 4, macrocycle **M3** catalyzed the reaction of **6a** and **7a** to afford **8a** in 88% yield with 72% ee. Under the same conditions, compound **9** also efficiently catalyzed the reaction and promoted an excellent conversion, however, it furnished the product in nearly racemic form. This suggested that the macrocyclic framework is essential in enabling a defined chiral environment for efficient stereocontrol. On the other hand, compound **3c** led to a much slower conversion and also nearly racemic product formation, indicating that the thiourea units must have engaged in activation and being cooperative with the tertiary amine sites.

**Table 4:** Evaluation of macrocyclic effect^a^.

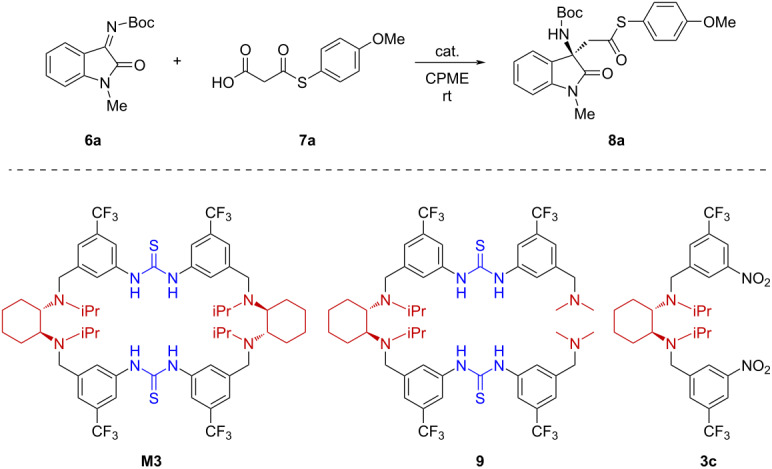				

Entry	Cat.	Time (h)	Yield (%)^b^	ee (%)^c^

1	**M3** (5 mol %)	44	88	72
2	**9** (5 mol %)	44	92	3
3	**3c** (10 mol %)	72	47	−2

^a^Reaction conditions: **6a** (0.2 mmol), **7a** (0.3 mmol), 2 mL of CPME (cyclopentyl methyl ether), room temperature; ^b^isolated yields after column chromatography; ^c^determined by HPLC analysis on a chiral stationary phase.

**Figure 2:**
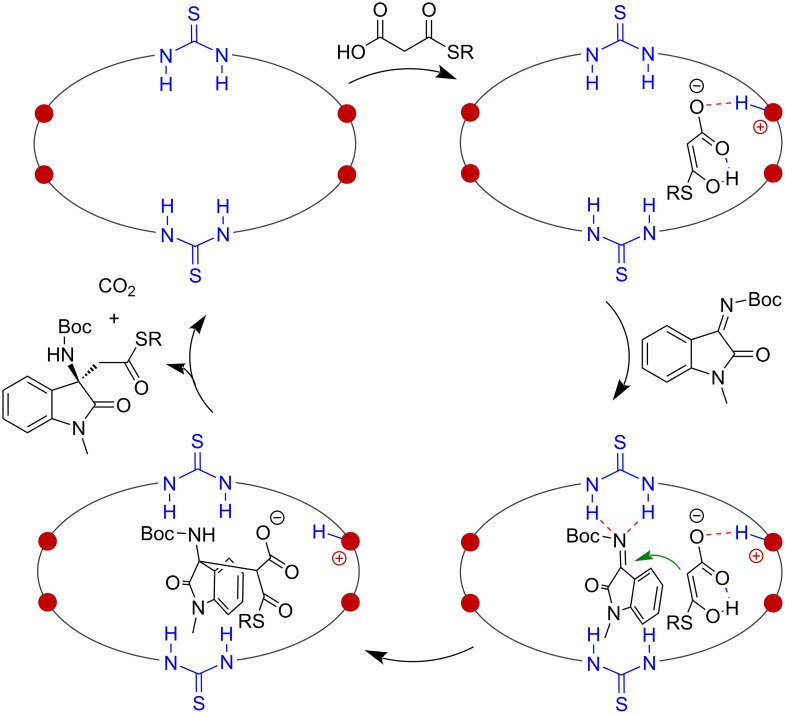
Proposed catalytic mechanism.

With the reaction outcomes and pronounced macrocyclic effect observed, a plausible catalytic mechanism is represented in Figure 2. The MAHT substrate is deprotonated by one of the tertiary amine sites, and the formed enolate intermediate can be stabilized by hydrogen bonding-mediated ion-pair interaction within the macrocyclic cavity. The imine substrate is activated by one or both of the two thiourea sites through hydrogen bonding to accept the enolate attack. The chiral environment provided by the (*S*,*S*)-cyclohexanediamine part governed the face-selective attack and led to the *R*-configurated product. In the last step, the decarboxylation leads to the enolate of the thioester, which is more basic than the MAHT-enolate and can thus be protonated by the ammonium fragment in the macrocycle. This leads to the neutral product, which can easily escape from the macrocyclic cavity, releasing the macrocycle catalyst to enter the next catalytic cycle. As suggested by the above control experiments, the rigid macrocyclic framework is crucial in organizing the multiple functional sites for cooperative binding and activation.

## Conclusion

In conclusion, a series of multifunctional tetraamino-bisthiourea chiral macrocycles were efficiently synthesized. By using a modular fragment-coupling approach, different chiral diamine units, including the homo- and hetero-combination, can be easily incorporated. This provides a very rich structural diversity of the macrocycles and allows for fine tuning of the chiral cavity environments. With the short and high-yielding synthesis, gram-scale of macrocycle products can be readily obtained. This kind of macrocycles can efficiently catalyze the decarboxylative addition of malonic acid half thioesters to isatin-derived ketimines, affording a series of chiral β-amino ester products in excellent yields and good enantioselectivity. In contrast, reactions catalyzed by acyclic analogues containing very similar structural units were non-selective, suggesting the essential role of the rigid macrocyclic framework in realizing efficient stereocontrol. With the easy synthesis, rich structural diversity, cooperative binding and activation sites, we believe this type of biomimetic chiral macrocycles will find more applications as catalysts in other reactions.

## Supporting Information

File 1 Experimental procedures, characterization data, copies of ^1^H and ^13^C NMR spectra.
