# Supplementary material for: Bioinspired tetraamino-bisthiourea chiral macrocycles in catalyzing decarboxylative Mannich reactions
Source: Beilstein J Org Chem. 2022 May 2;18:486–96. doi: 10.3762/bjoc.18.51 (PMC9086498; doi:10.3762/bjoc.18.51)
Supplement: File 1 — Experimental procedures, characterization data, copies of 1H and 13C NMR spectra. [file Beilstein_J_Org_Chem-18-486-s001.pdf]

BEILSTEIN JOURNAL OF ORGANIC CHEMISTRY

Supporting Information

for

Bioinspired tetraamino-bisthiourea chiral macrocycles in catalyzing decarboxylative Mannich reactions

Hao Guo, Yu-Fei Ao, De-Xian Wang and Qi-Qiang Wang

Beilstein J. Org. Chem. **2022,** *18,* 486–496. doi:10.3762/bjoc.18.51

Experimental procedures, characterization data, copies of ^{1}H and ^{13}C NMR spectra

License and Terms: This is a supporting information file under the terms of the Creative Commons Attribution License (https://creativecommons.org/licenses/by/4.0). Please note that the reuse, redistribution and reproduction in particular requires that the author(s) and source are credited and that individual graphics may be subject to special legal provisions.
The license is subject to the *Beilstein Journal of Organic Chemistry* terms and conditions: (https://www.beilstein-journals.org/bjoc/terms)

Table of contents

1. General information ········· S2

2. Synthesis ········· S3

2.1 Synthesis of dinitro compounds **3** ········· S3

2.2 Synthesis of diamine compounds **4** ········· S7

2.3 Synthesis of diisothiocyanate compounds **5** ········· S10

2.4 Synthesis of macrocycles **M** ········· S13

2.5 Synthesis of acyclic control compound **9** ········· S19

3. Catalysis studies ········· S21

3.1 Typical procedure for the decarboxylative Mannich reactions ········· S21

3.2 Characterization data for products **8** ········· S22

4. Copies of ^{1}H and ^{13}C NMR spectra ········· S39

5. HPLC analysis of products ········· S93

6. References ········· S126

1. General information

All chemicals were obtained from commercial sources and used without further purification unless stated otherwise. Anhydrous solvents such as THF, CH_2Cl_2, $CHCl_3$, CH_3CN, Et_2O, 1,4-dioxane, methyl *tert*-butyl ether (TBME), 1,2-dimethoxyethane (DME), ethyl vinyl ether (EVE), and cyclopentyl methyl ether (CPME) were obtained by conventional methods through distilling with suitable drying agents (Na for THF, Et_2O, 1,4-dioxane, MTBE, and toluene; CaH_2 for CH_2Cl_2, $CHCl_3$, CH_3CN, and CPME; 4 Å molecular sieves for EVE and DME). NMR spectra were recorded on Bruker 300, 400 or 500 MHz NMR spectrometers. Chemical shifts are reported in ppm and referenced to tetramethylsilane or the residual solvent resonance. Mass spectra were obtained on a Thermo Fisher Exactive Mass Spectrometer. Infrared spectra were recorded on Nicolet-6700 FT-IR spectrometer. Elemental analysis was recorded on Carlo Erba 1106. Optical rotations were performed on Rudolph Autopl VI. High performance liquid chromatography (HPLC) was performed on Shimadzu SCL-20AVP. Melting points are uncorrected.

2. Synthesis

Compounds **3a**, **3e**, **4a**, **4e**, **5a**, **5e**, **M1**, **M5**, **M7**, and **M8** were synthesized according to our previous reported method.[1]

2.1 Synthesis of dinitro compounds 3

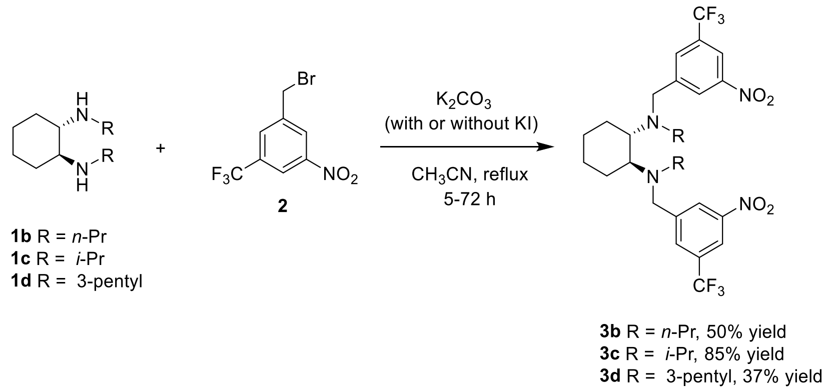

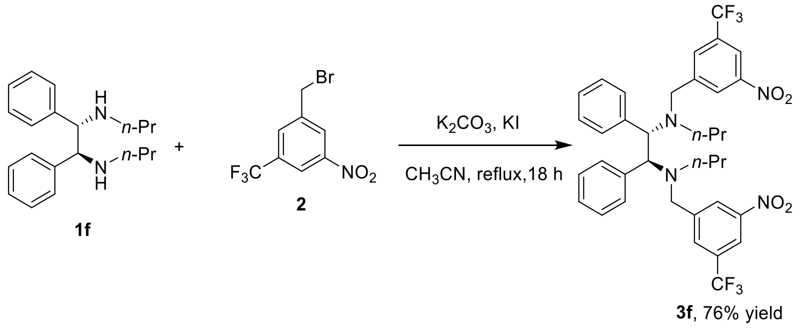

General procedure: To a solution of chiral 1,2-diamine **1** (1.0 equiv) and 1-(bromomethyl)-3-nitro-5-(trifluoromethyl)benzene (**2**, 2.2 equiv) in acetonitrile, K_2CO_3 (4 equiv) and KI (if applicable) were added. The resulting mixture was heated at reflux for a given period. After work-up by removal of the solvent under reduced pressure, extraction (if applicable), the crude product was subjected to column chromatography on silica gel (petroleum ether/ethyl acetate as eluent) to give the corresponding dinitro compound **3**.

(1*S*,2*S*)-*N*1,*N*2-Bis(3-nitro-5-(trifluoromethyl)benzyl)-*N*1,*N*2-dipropylcyclohexane-1,2-diamine (3b)

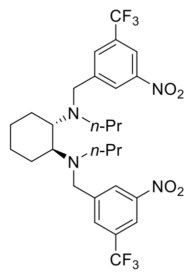

3b

Dinitro compound **3b** was synthesized according to the general procedure by heating the mixture of (1*S*,2*S*)-*N*1,*N*2-dipropylcyclohexane-1,2-diamine (**1b**[2], 3.97 g, 20 mmol), 1-(bromomethyl)-3-nitro-5-(trifluoromethyl)benzene (**2**, 12.50 g, 44 mmol) and K_2CO_3 (11.06g, 80 mmol) in acetonitrile (300 mL) at reflux for 10 h. After work-up by removal of solvent, addition of water (100 mL) and extraction with ethyl acetate (100 mL × 3), column chromatography (petroleum ether/ethyl acetate 10:1) gave **3b** as a yellow oil (6.08 g, yield: 50%). ^{1}H NMR ($CDCl_3$, 500 MHz) δ (ppm) 8.48 (s, 2H), 8.31 (s, 2H), 7.96 (s, 2H), 3.86 (d, *J* = 14.4 Hz, 1H), 3.53 (d, *J* = 14.4 Hz, 1H), 2.81-2.64 (m, 2H), 2.48 (t, *J* = 7.6 Hz, 4H), 2.22-1.98 (m, 2H), 1.91-1.68 (m, 2H), 1.65-1.51 (m, 2H), 1.51-1.37 (m, 2H), 1.30-1.08 (m, 4H), 0.81 (t, *J* = 7.3 Hz, 6H); ^{13}C NMR ($CDCl_3$, 125 MHz) δ (ppm) 148.4, 145.7, 131.9 (q, *J* = 33.8 Hz, *C*-CF_3), 131.3 (q, *J* = 3.3 Hz, *C*-C-CF_3), 127.0, 123.1 (q, *J* = 271.3 Hz, CF_3), 119.3 (q, *J* = 3.8 Hz, *C*-C- CF_3), 60.9, 53.8, 52.9, 26.0, 25.6, 22.0, 12.0; IR (KBr) ν 3098, 2934, 2858, 1542, 1471, 1323, 1174, 1136, 1107 cm^{-1}; HRMS (APCI$^+$) calc. for $[M+H]^+$ ($C_{28}H_{35}F_6N_4O_4^+$), 605.2557, found 605.2546; $[\alpha]_D^{25}$ = +41.4 (c = 0.50, $CHCl_3$).

(1*S*,2*S*)-*N*1,*N*2-Diisopropyl-*N*1,*N*2-bis(3-nitro-(trifluoromethyl)benzyl)cyclo-hexane-1,2-diamine (3c)

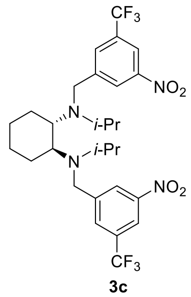

3c

Dinitro compound **3c** was synthesized according to the general procedure by heating the mixture of (1*S*,2*S*)-*N*1,*N*2-diisopropylcyclohexane-1,2-diamine (**1c**[3], 5.95 g, 30 mmol), 1-(bromomethyl)-3-nitro-5-(trifluoromethyl)benzene (**2**, 18.69 g, 66 mmol), K_2CO_3 (16.60 g, 120 mmol) and KI (300 mg, 1.8 mmol) in acetonitrile (360 mL) at reflux for 40 h. After work-up by removal of solvent, column chromatography (petroleum ether/ethyl acetate 20:1) gave **3c** as a yellow solid (15.40 g, yield: 85%). Mp 98-99 ^{o}C; ^{1}H NMR ($CDCl_3$, 400 MHz) δ (ppm)

8.47 (s, 2H), 8.32 (s, 2H), 7.95 (s, 2H), 3.77 (d, *J* = 14.4 Hz, 2H), 3.65 (d, *J* = 14.4 Hz, 2H), 3.03-2.80 (m, 2H), 2.73-2.54 (m, 2H), 2.24-2.07 (m, 2H), 1.85-1.68 (m, 2H), 1.17 (d, *J* = 6.6 Hz, 6H), 1.14-1.01 (m, 4H), 0.97 (d, *J* = 6.5 Hz, 6H); ^{13}C NMR ($CDCl_3$, 100 MHz) δ (ppm) 148.4, 146.0, 131.9 (q, *J* = 33.7 Hz, *C*-CF_3), 131.5 (q, *J* = 3.4 Hz, *C*-C-CF_3), 127.2, 123.1 (q, *J* = 271.2 Hz, *C*F_3), 119.3 (q, *J* = 3.1 Hz, *C*-C-CF_3), 60.1, 48.5, 48.4, 28.5, 26.4, 23.2, 20.3; IR (KBr) ν 3098, 2936, 2859, 1542, 1352, 1322, 1171, 1134, 1108 cm^{-1}; HRMS ($APCI^+$) calc. for $[M+H]^+$ ($C_{28}H_{35}F_6N_4O_4^+$), 605.2557, found 605.2547; $[\alpha]_D^{25}$ = +24.6 (c = 0.50, $CHCl_3$).

(1*S*,2*S*)-*N*¹,*N*²-Bis(3-nitro-5-(trifluoromethyl)benzyl)-*N*¹,*N*²-di(pentan-3-yl)cyclohexane-1,2-diamine (3d)

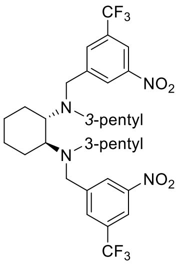

3d

Dinitro compound **3d** was synthesized according to the general procedure by heating the mixture of (1*S*,2*S*)-*N*¹,*N*²-di(pentan-3-yl)cyclohexane-1,2-diamine (**1d**[4], 4.58 g, 18 mmol), 1-(bromomethyl)-3-nitro-5-(trifluoromethyl)benzene (**2**, 15.34 g, 54 mmol) and K_2CO_3 (9.95 g, 72 mmol), KI (180 mg, 1.1 mmol) in acetonitrile (200 mL) at reflux for 72 h. After work-up by removal of solvent, column chromatography (petroleum ether/ethyl acetate 20:1) gave **3d** as a yellow oil (4.37 g, yield: 37%). ^{1}H NMR ($CDCl_3$, 300 MHz) δ (ppm) 8.37 (s, 2H), 8.29 (s, 2H), 7.88 (s, 2H), 3.82-3.62 (m, 4H), 2.78-2.63 (m, 2H), 2.61-2.46 (m, 2H), 2.17-1.99 (m, 2H), 1.77-1.66 (m, 2H), 1.64-1.43 (m, 6H), 1.36-1.11 (m, 6H), 0.94 (t, *J* = 7.4 Hz, 6H), 0.85(t, *J* = 7.4 Hz, 6H); ^{13}C NMR ($CDCl_3$, 125 MHz) δ (ppm) 148.3, 146.4, 131.9 (q, *J* = 33.7 Hz, *C*-CF_3), 131.0 (q, *J* = 3.3 Hz, *C*-C-CF_3), 126.6, 123.0 (q, *J* = 272.9 Hz, *C*F_3), 119.1(q, *J* = 3.7 Hz, *C*-C-CF_3), 61.8, 60.8, 49.9, 28.5, 27.2, 25.3, 24.5, 12.7, 11.9 ; IR (KBr) ν 3098, 2935, 2876, 1543, 1323, 1174, 1136 cm^{-1}; HRMS ($APCI^+$) calc. for $[M+H]^+$ ($C_{32}H_{43}F_6N_4O_4^+$), 661.3183, found 661.3178; $[\alpha]_D^{25}$ = +6.4 (c = 0.45, $CHCl_3$).

(1*S*,2*S*)-*N*[1],*N*[2]-Bis(3-nitro-5-(trifluoromethyl)benzyl)-1,2-diphenyl-*N*[1],*N*[2]-dipropylethane-1,2-diamine (3f)

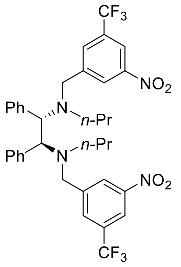

3f

Dinitro compound **3f** was synthesized according to the general procedure by heating the mixture of (1*S*,2*S*)-1,2-diphenyl-*N*[1],*N*[2]-dipropylethane-1,2-diamine **1f**[5] (3.55 g, 12 mmol), 1-(bromomethyl)-3-nitro-5-(trifluoromethyl)benzene (**2**, 7.50 g, 26.4 mmol), K_2CO_3 (6.63 g, 48 mmol) and KI (120 mg, 0.7 mmol) in acetonitrile (180 mL) at reflux for 18 h. After work-up by removal of solvent, column chromatography (petroleum ether/ethyl acetate 10:1) gave **3f** as a yellow solid (6.42 g, yield: 76%). Mp 128-129 °C; ^{1}H NMR ($CDCl_3$, 500 MHz) δ (ppm) 8.50 (s, 2H), 8.37 (s, 2H), 7.98 (s, 2H), 7.20-7.08 (m, 6H), 6.97 (d, *J* = 7.2 Hz, 4H), 4.50 (s, 2H), 4.03 (d, *J* = 14.3 Hz, 2H), 3.23 (d, *J* = 14.3 Hz, 2H), 2.72-2.60 (m, 2H), 2.37-2.22 (m, 2H), 1.81-1.60 (m, 4H), 0.92 (t, *J* = 7.3 Hz, 6H); ^{13}C NMR ($CDCl_3$, 100 MHz) δ (ppm) 148.6, 144.9, 135.5, 132.2 (q, *J* = 33.6 Hz, *C*-CF_3), 131.3, 129.5, 128.1, 127.5, 126.9, 123.0 (q, *J* = 271.4 Hz, *C*F_3), 119.6 (q, *J* = 3.8 Hz, *C*-C-CF_3), 64.4, 53.8, 52.6, 21.7, 12.1; IR (KBr) ν 2962, 2875, 1541, 1351, 1174, 1134 cm^{-1}; HRMS (ESI$^+$) calc. for $[M+H]^+$ ($C_{36}H_{37}F_6N_4O_4^+$), 703.2714, found 703.2705; $[\alpha]_D^{25}$ = +89.6 (*c* = 0.50, $CHCl_3$).

2.2 Synthesis of diamine compounds 4

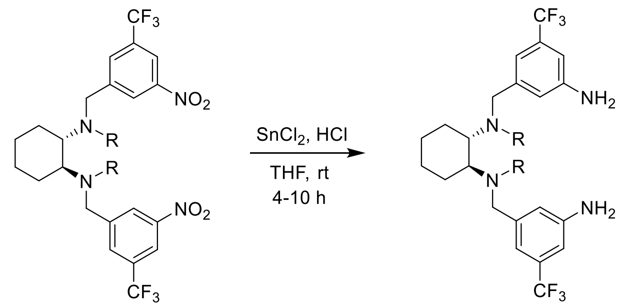

3b R = *n*-Pr
3c R = *i*-Pr
3d R = 3-pentyl

4b, R = *n*-Pr, 83% yield
4c, R = *i*-Pr, 83% yield
4d, R = 3-pentyl, 89% yield

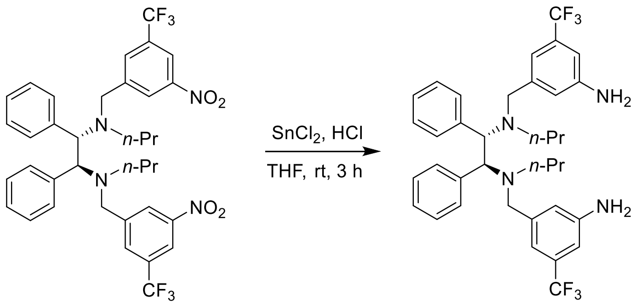

3f

4f, 98% yield

General procedure: To a solution of **3** (1.0 equiv) in THF was added a solution of $SnCl_2 \cdot 2H_2O$ (6.4 equiv) in a given volume of *conc.* hydrochloric acid. The resulting mixture was stirred at room temperature for a given period. Afterwards, the mixture was treated with a solution of 40% aq. NaOH to adjust pH > 10. The organic layer was separated, and the aqueous layer was extracted with ethyl acetate. The combined organic layers were dried over Na_2SO_4, and then evaporated under reduced pressure. The residue was subjected to column chromatography on silica gel (dichloromethane/methanol as eluent) to give the corresponding diamine compound **4**.

(1*S*,2*S*)-*N*[1],*N*[2]-Bis(3-amino-5-(trifluoromethyl)benzyl)-*N*[1],*N*[2]-dipropylcyclohexane-1,2-diamine (4b)

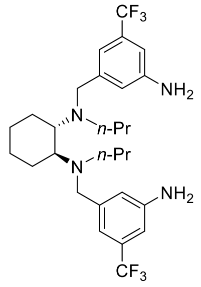

4b

Diamine compound **4b** was synthesized according to the general procedure by reaction of **3b** (6.04 g, 10 mmol) with $SnCl_2\cdot 2H_2O$ (11.03 g, 64 mmol), *conc.* hydrochloric acid (25 mL) in THF (500 mL) for 4 h. After work-up, column chromatography (dichloromethane/methanol 10:1) gave **4b** as a yellow oil (4.53 g, yield: 83%). ^{1}H NMR ($CDCl_3$, 400 MHz) δ (ppm) 7.03 (s, 2H), 6.94 (s, 2H), 6.73 (s, 2H), 3.92-3.47 (m, 6H), 3.30 (d, *J* = 14.1 Hz, 2H), 2.70-2.56 (m, 2H), 2.45 (s, 2H), 2.39-2.27 (m, 2H), 2.09-1.93 (m, 2H), 1.79-1.64 (m, 2H), 1.63-1.36 (m, 4H), 1.17-0.98 (m, 4H), 0.85 (t, *J* = 7.1 Hz, 6H); ^{13}C NMR ($CDCl_3$, 100 MHz) δ (ppm) 146.7, 144.3, 131.1 (q, *J* = 31.6 Hz, *C*-CF_3), 124.5 (q, *J* = 272.4 Hz, *C*F3), 118.6, 115.6, 109.7, 60.1 53.9, 52.1, 26.2, 25.2, 22.0, 12.0; IR (KBr) ν 3385, 3321, 2933, 2858, 2808, 1625, 1464, 1356, 1257, 1161, 1122 cm^{-1}; HRMS (ESI$^-$) calc. for $[M-H]^-$ ($C_{28}H_{39}F_6N_4^-$), 545.3073, found 545.3072; $[\alpha]_D^{25}$ = +74.6 (*c* = 0.70, $CHCl_3$).

(1*S*,2*S*)-*N*[1],*N*[2]-Bis(3-amino-5-(trifluoromethyl)benzyl)-*N*[1],*N*[2]-diisopropylcyclohexane-1,2-diamine (4c)

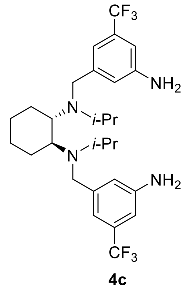

4c

Diamine compound **4c** was synthesized according to the general procedure by reaction of **3c** (15.11 g, 25 mmol) with $SnCl_2\cdot 2H_2O$ (27.57 g, 160 mmol), *conc.* hydrochloric acid (62.5 mL) in THF (1250 mL) for 10 h. After work-up, column chromatography (dichloromethane/methanol 10:1) gave **4c** as a yellow oil (11.30 g, yield: 83%). Mp 167-168 °C; ^{1}H NMR (DMSO-d_6, 400 MHz) δ (ppm) 6.81 (s, 2H), 6.71 (s, 2H), 6.68 (s, 2H), 5.39 (s, br, 4H), 3.56 (d, *J* = 13.4 Hz, 2H), 3.31 (d, *J* = 13.0 Hz, 2H), 2.82-2.69 (m, 2H), 2.46-2.36 (m, 2H), 2.15-2.03 (m, 2H), 1.71-1.55 (m, 2H), 1.12 (d, *J* = 6.4 Hz, 6H), 0.99-0.84 (m, 4H), 0.81 (d, *J* = 6.4 Hz, 6H); ^{13}C NMR (DMSO-d_6, 100 MHz) δ (ppm) 148.9, 142.8, 129.3 (q, *J* = 30.6 Hz, *C*-CF_3), 124.7 (q, *J* = 272.2 Hz, *C*F_3), 118.11, 112.5 (q, *J* = 3.9 Hz, *C*-C-CF_3), 107.7 (q, *J* = 3.7 Hz, *C*-C-CF_3), 59.2, 47.2, 45.5, 27.4, 26.1, 23.2, 19.6; IR (KBr) ν 3474, 3392, 2986, 2922, 2849,

1460, 1352, 1322, 1171, 1134, 1107 cm^{-1}; HRMS (ESI^+) calc. for $[M+H]^+$ ($C_{28}H_{39}F_6N_4^+$), 545.3073, found 545.3068; $[\alpha]_D^{25}$ = +54.4 (*c* = 0.50, $CHCl_3$).

(1*S*,2*S*)-*N*¹,*N*²-Bis(3-amino-5-(trifluoromethyl)benzyl)-*N*¹,*N*²-di(pentan-3-yl)cyclohexane-1,2-diamine (4d)

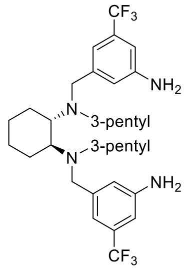

4d

Diamine compound **4d** was synthesized according to the general procedure by reaction of **3d** (1.65 g, 2.5 mmol) with $SnCl_2·2H_2O$ (2.76 g, 16 mmol), *conc.* hydrochloric acid (6.25 mL) in THF (125 mL) for 10 h. After work-up, column chromatography (dichloromethane/methanol 10:1) gave **4d** as a yellow oil (1.34 g, yield: 89%). ^{1}H NMR ($CDCl_3$, 400 MHz) δ (ppm) 6.98 (s, 2H), 6.81 (s, 2H), 6.69 (s, 2H), 3.88-3.30 (m, 8H), 2.65 (s, 2H), 2.51 (s, 2H), 2.15-1.97 (m, 2H), 1.76-1.47 (m, 9H), 1.36-1.20 (m, 3H), 1.20-1.03 (m, 4H), 0.94 (t, *J* = 7.1 Hz ,7H), 0.83 (t, *J* = 7.1Hz , 7H); ^{13}C NMR ($CDCl_3$, 100 MHz) δ (ppm) 146.5, 144.9, 131.0 (q, *J* = 31.4 Hz, *C*-CF_3), 124.5 (q, *J* = 270.3 Hz, *C*F_3), 118.6, 115.8 (q, *J* = 3.7 Hz, *C*-C-CF_3), 109.6 (q, *J* = 3.0 Hz, *C*-C-CF_3), 60.8, 60.4, 49.9, 28.3, 27.4, 25.8, 24.4, 12.8, 12.0; IR (KBr) ν 3388, 3213, 2933, 2874, 1624, 1464, 1366, 1258, 1161, 1122 cm^{-1}; HRMS (ESI^+) calc. for $[M+H]^+$ ($C_{32}H_{47}F_6N_4^+$), 601.3699, found 601.3701; $[\alpha]_D^{25}$ = +27.8 (*c* = 0.50, $CHCl_3$).

(1*S*,2*S*)-*N*¹,*N*²-Bis(3-amino-5-(trifluoromethyl)benzyl)-1,2-diphenyl-*N*¹,*N*²-di-propylethane-1,2-diamine (4f)

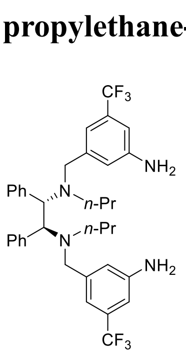

4f

Diamine compound **4f** was synthesized according to the general procedure by reaction of **3f** (1.40 g, 2.0 mmol) with $SnCl_2·2H_2O$ (2.21 g, 12.8 mmol), *conc.* hydrochloric acid (5 mL) in THF (100 mL) for 3 h. After work-up, column chromatography (dichloromethane/methanol 10:1) gave **4f** as a brown solid (1.26 g, yield: 98%). Mp 48-49 °C; ^{1}H NMR ($CDCl_3$, 500 MHz) δ (ppm) 7.16-7.09 (m, 4H), 7.09-7.02 (m, 4H), 7.01-6.90 (m, 6H), 6.77 (s, 2H), 4.45 (s, 2H), 3.87 (d, *J* = 14.1 Hz, 2H), 3.56 (s, br, 4H), 2.96 (d, *J* = 14.1 Hz, 2H), 2.74-2.62 (m, 2H), 2.16 -2.08 (m, 2H), 1.78 -1.65 (m, 4H), 0.94 (t, *J* = 7.4 Hz, 6H); ^{13}C NMR ($CDCl_3$, 125 MHz) δ (ppm) 146.8, 143.7, 136.1,

131.4 (q, J = 31.6 Hz, C-CF_3), 129.8, 127.7, 126.9, 124.5 (q, J = 270.8 Hz, CF_3), 118.4, 115.7 (q, J = 3.7 Hz, C-C-CF_3), 110.0 (q, J = 3.8 Hz, C-C-CF_3), 63.6, 53.8, 51.8, 21.7, 12.2; IR (KBr) ν 3465, 3390, 2961, 2932, 2873, 1625, 1465, 1375, 1356, 1261, 1121 cm^{-1}; HRMS (ESI^+) calc. for $[M+H]^+$ ($C_{36}H_{41}F_6N_4^+$), 643.3230, found 643.3203; $[\alpha]_D^{25}$ = +107.4 (c = 0.50, $CHCl_3$).

Enantiomers *ent*-**4a**, *ent*-**4b** and *ent*-**4c** were synthesized according to the same methods as described for **4a**, **4b** and **4c** starting from (1*R*,2*R*)-N^1,N^2-disubstituted cyclohexane-1,2-diamines, respectively. The characterization data were consistent.

2.3 Synthesis of diisothiocyanate compounds 5

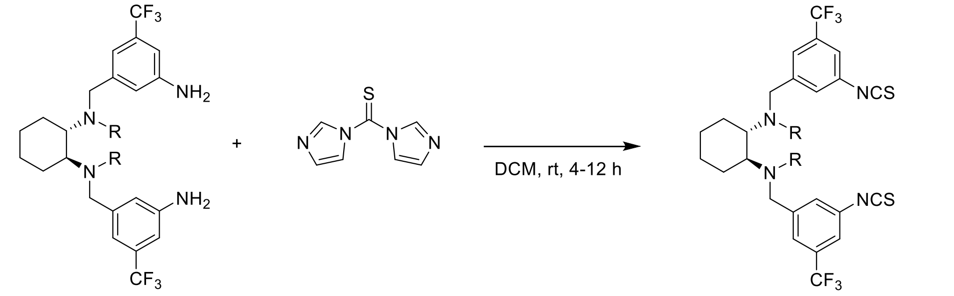

4b R = *n*-Pr
4c R = *i*-Pr
4d R = 3-pentyl

5b R = *n*-Pr, 86% yield
5c R = *i*-Pr, 89% yield
5d R = 3-pentyl, 68% yield

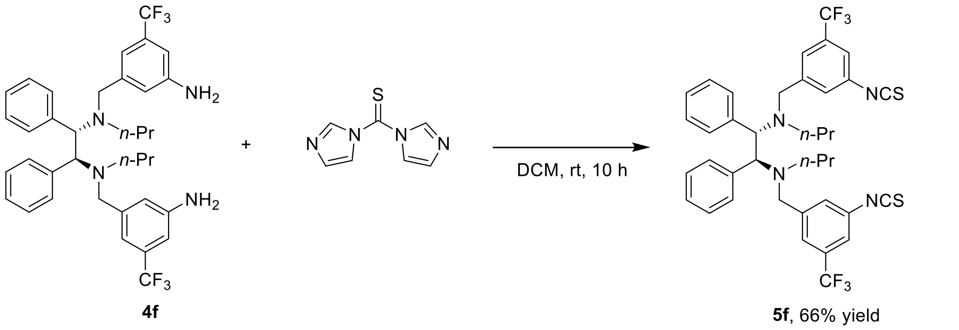

4f

5f, 66% yield

General procedure: To a solution of **4** (1.0 equiv) in CH_2Cl_2 was added 1,1'-thiocarbonyldiimidazole (4.0 equiv). The mixture was stirred at room temperature for a given period. The solvent was evaporated under reduced pressure and the residue was subjected to column chromatography on silica gel to give compounds **5**.

(1*S*,2*S*)-*N*[1],*N*[2]-Bis(3-isothiocyanato-5-(trifluoromethyl)benzyl)-*N*[1],*N*[2]-dipropyl-cyclohexane-1,2-diamine (5b)

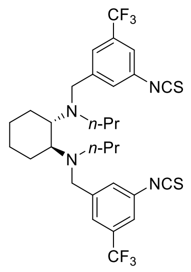

5b

Diisothiocyanate compound **5b** was synthesized according to the general procedure by reaction of **4b** (2.72 g, 5.0 mmol) with 1,1'-thiocarbonyldiimidazole (3.56 g, 20 mmol) in DCM (200 mL) for 12 h. After work-up, column chromatography (petroleum ether/ethyl acetate 5:1) gave **5b** as a brown oil (2.70 g, yield: 86%). ^{1}H NMR ($CDCl_3$, 500 MHz) δ (ppm) 7.53 (s, 2H), 7.43 (s, 2H), 7.30 (s, 2H), 3.71 (d, *J* = 14.3 Hz, 2H), 3.36 (d, *J* = 14.3 Hz, 2H), 2.68-2.57 (m, 2H), 2.54-2.34 (m, 4H), 2.08-1.96 (m, 2H), 1.83-1.70 (m, 2H), 1.56-1.35 (m, 4H), 1.20-1.06 (m, 4H), 0.82 (t, *J* = 7.3 Hz, 6H); ^{13}C NMR ($CDCl_3$, 125 MHz) δ (ppm) 145.2, 137.6, 132.1, 131.9 (q, *J* = 32.8 Hz, *C*-CF_3), 129.4, 124.2 (q, *J* = 3.4 Hz, *C*-C-CF_3), 123.5 (q, *J* = 271.2 Hz, *C*F$_3$), 121.0 (q, *J* = 3.6 Hz, *C*-C-CF_3), 60.7, 53.8, 52.7, 26.1, 25.5, 22.1, 12.1; IR (KBr) ν 2933, 2857, 2062, 1603, 1456, 1347, 1241, 1169, 1130 cm^{-1}; HRMS (APCI$^+$) calc. for $[M+H]^+$ ($C_{30}H_{35}F_6N_4S_2^+$), 629.2202, found 629.2191; $[\alpha]_D^{25}$ = +20.8 (*c* = 0.50, $CHCl_3$).

(1*S*,2*S*)-*N*[1],*N*[2]-Diisopropyl-*N*[1],*N*[2]-bis(3-isothiocyanato-5-(trifluoromethyl)ben-zyl)cyclohexane-1,2-diamine (5c)

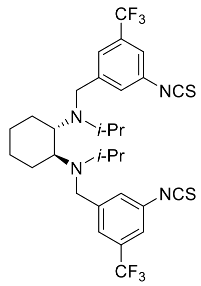

5c

Diisothiocyanate compound **5c** was synthesized according to the general procedure by reaction of **4c** (272 mg, 0.5 mmol) with 1,1'-thiocarbonyldiimidazole (356 mg, 2 mmol) in DCM (20 mL) for 4 h. After work-up, column chromatography (petroleum ether/ethyl acetate 5:1) gave **5c** as a brown oil (282 mg, yield: 89%). ^{1}H NMR ($CDCl_3$, 400 MHz) δ (ppm) 7.52 (s, 2H), 7.40 (s, 2H), 7.31 (s, 2H), 3.64 (d, *J* = 14.2 Hz, 2H), 3.48 (d, *J* = 14.2 Hz, 2H), 2.92- 2.75 (m, 2H), 2.61 -2.40 (m, 2H), 2.20-2.04 (m, 2H), 1.81-1.63 (m, 2H), 1.17 (d, *J* = 6.6 Hz, 6H), 1.13-0.99 (m, 4H), 0.93 (d, *J* = 6.5 Hz, 6H); ^{13}C NMR ($CDCl_3$, 100 MHz) δ (ppm) 145.3, 137.5, 132.0, 131.9 (q, *J* = 32.7 Hz, *C*-CF_3), 129.5, 124.5 (q, *J* = 3.4 Hz, *C*-C-CF_3), 123.5 (q, *J* = 271.0 Hz, *C*F$_3$), 121.1 (q, *J* = 3.8 Hz, *C*-C-CF_3), 60.1, 48.2, 47.6, 28.3, 26.50, 23.5, 20.2; IR (KBr) ν 2966, 2934, 2857, 2074, 1602, 1452, 1344, 1241, 1169, 1130 cm^{-1}; HRMS

(APCI$^+$) calc. for $[M+H]^+$ ($C_{30}H_{35}F_6N_4S_2^+$), 629.2201, found 629.2193; $[\alpha]_D^{25}$ = +26.8 (c = 0.50, $CHCl_3$).

(1*S*,2*S*)-*N*¹,*N*²-Bis(3-isothiocyanato-5-(trifluoromethyl)benzyl)-*N*¹,*N*²-di(pentan-3-yl)cyclohexane-1,2-diamine (5d)

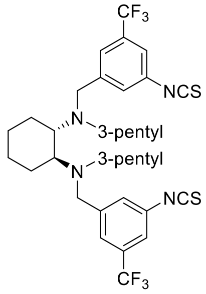

5d

Diisothiocyanate compound **5d** was synthesized according to the general procedure by reaction of **4d** (1.80 g, 3.0 mmol) with 1,1'-thiocarbonyldiimidazole (2.14 g, 12 mmol) in DCM (120 mL) for 12 h. After work-up, column chromatography (petroleum ether/ethyl acetate 10:1) gave **5d** as a brown oil (1.40 g, yield: 68%). ^{1}H NMR ($CDCl_3$, 400 MHz) δ (ppm) 7.46 (s, 2H), 7.31 (s, 2H), 7.28 (s, 2H), 3.60 (d, J = 14.8 Hz , 2H), 3.52 (d, J = 14.8 Hz , 2H), 2.71-2.56 (m, 2H), 2.53-2.40 (m, 2H), 2.13-1.98 (m, 2H), 1.76-1.65 (m, 2H), 1.65-1.46 (m, 6H), 1.31-1.20 (m, 3H), 1.60-1.09 (m, 3H), 0.94 (t, J = 7.4 Hz , 6H), 0.84 (t, J = 7.4 Hz , 6H); ^{13}C NMR ($CDCl_3$, 100 MHz) δ (ppm) 145.7, 137.5, 131.8 (q, J = 32.7 Hz, *C*-CF_3), 129.0, 124.2 (q, J = 3.8 Hz, *C*-C-CF_3), 123.4 (q, J = 271.2 Hz, *C*F_3), 121.0 (q, J = 3.8 Hz, *C*-C-CF_3), 61.3, 60.6, 49.7, 28.3, 27.4, 25.5, 24.5, 12.7, 11.9; IR (KBr) ν 2962, 2933, 2875, 2070, 1602, 1460, 1345, 1241, 1171 cm^{-1}; HRMS (APCI$^+$) calc. for $[M+H]^+$ ($C_{34}H_{43}F_6N_4S_2^+$), 685.2828, found 685.2833; $[\alpha]_D^{25}$ = +0.6 (c = 0.50, $CHCl_3$).

(1*S*,2*S*)-*N*¹,*N*²-Bis(3-isothiocyanato-5-(trifluoromethyl)benzyl)-1,2-diphenyl-*N*¹,*N*²-dipropylethane-1,2-diamine (5f)

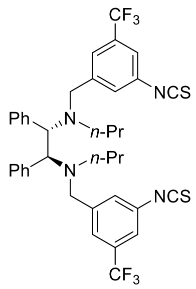

5f

Diisothiocyanate compound **5f** was synthesized according to the general procedure by reaction of **4f** (3.85 g, 6.0 mmol) with 1,1'-thiocarbonyldiimidazole (4.28 g, 24 mmol) in DCM (240 mL) for 10 h. After work-up, column chromatography (petroleum ether/ethyl acetate 5:1) gave **5f** as a white solid (2.86 g, yield: 66%). Mp 119-120 °C; ^{1}H NMR ($CDCl_3$, 500 MHz) δ (ppm) 7.60 (s, 2H), 7.46 (s, 2H), 7.37 (s, 2H), 7.20-7.13 (m, 4H), 7.12-7.06 (m, 2H), 6.96 (d, J = 7.2 Hz, 4H), 4.44 (s, 2H), 3.91 (d, J = 14.2 Hz, 2H), 3.09 (d, J = 14.2 Hz, 2H), 2.71-2.59 (m, 2H), 2.29-2.15 (m, 2H),

1.79-1.60 (m, 4H), 0.94 (t, J = 7.3 Hz, 6H); ^{13}C NMR ($CDCl_3$, 125 MHz) δ (ppm) 144.4, 137.8, 135.7, 132.4, 132.1 (q, J = 32.9 Hz, C-CF_3), 129.6, 129.3, 128.0, 127.3, 124.3 (q, J = 3.4 Hz, C-C-CF_3), 123.4 (q, J = 271.2 Hz, CF_3), 121.4 (q, J = 3.7 Hz, C-C-CF_3), 64.2, 53.7, 52.3, 21.4, 12.1; IR (KBr) ν 3028, 2962, 2932, 2848, 2104, 1602, 1454, 1345, 1241, 1172, 1131 cm^{-1}; HRMS (APCI$^-$) calc. for $[M+H]^+$ ($C_{38}H_{37}F_6N_4S_2^-$), 727.2358, found 727.2347; $[\alpha]_D^{25}$ = +109.6 (c = 0.50, $CHCl_3$).

2.4 Synthesis of macrocycles M

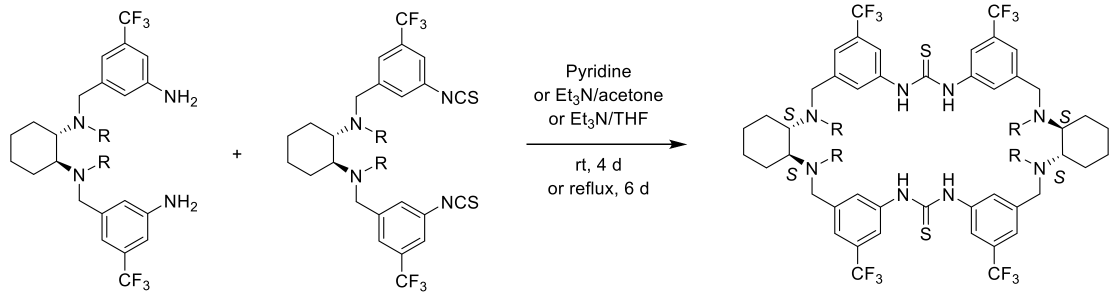

4a R = *n*-Pr
4b R = *i*-Pr
4c R = 3-pentyl

5a R = *n*-Pr
5b R = *i*-Pr
5c R = 3-pentyl

M2 R = *n*-Pr, 52% yield
M3 R = *i*-Pr, 35% yield
M4 R = 3-pentyl, 42% yield

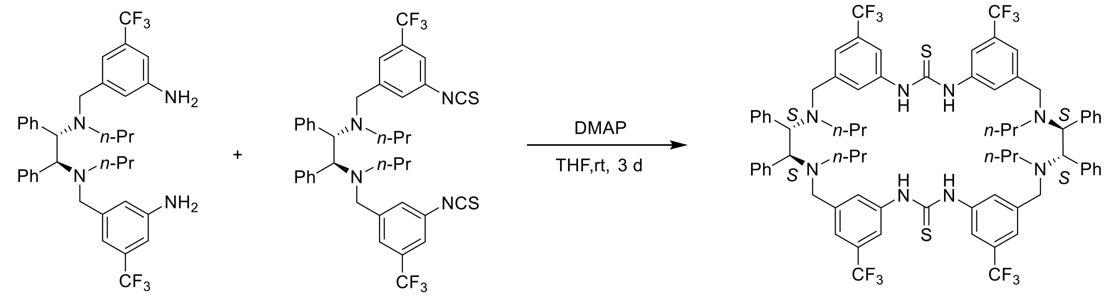

4f

5f

M6, 44% yield

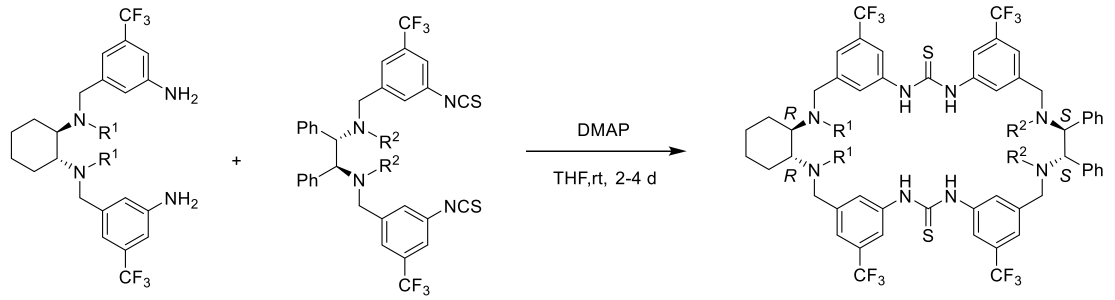

ent-**4a** R = Me
ent-**4b** R = *n*-Pr
ent-**4c** R = *i*-Pr

5e R = Me
5f R = *n*-Pr

M9 R^1 = Me; R^2 = *n*-Pr, 30% yield
M10 R^1 = *n*-Pr; R^2 = Me, 30% yield
M11 R^1 = *n*-Pr; R^2 = *n*-Pr, 32% yield
M12 R^1 = *i*-Pr; R^2 = *n*-Pr, 47% yield

General procedure: To a solution of **4** (1.0 equiv) in corresponding solvent (THF, acetone or pyridine) was added **5** (1.0 equiv) and corresponding base (DMAP or Et_3N). The mixture was stirred at room temperature or heated at reflux for a given period. The solvent was evaporated under reduced pressure, and the residue was subjected to column chromatography on silica gel. The crude product was washed with ethyl acetate and *n*-hexane to give the corresponding macrocycle **M**.

Macrocycle M2

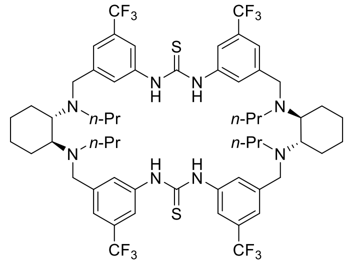

M2

Macrocycle **M2** was synthesized according to the general procedure by reaction of **4b** (1.36 g, 2.5 mmol), **5b** (1.57 g, 2.5 mmol) and Et_3N (875 μL, 6.3 mmol) in THF (50 mL) at room temperature for 96 h. Column chromatography eluent: dichloromethane/methanol 10:1. Macrocycle **M2** was obtained as a light yellow solid (1.54 g, yield: 52%). Mp 141-142 °C; ^{1}H NMR (DMSO-d_6, 400 MHz) δ (ppm) 9.75 (s, 4H), 7.81-7.31 (m, 12H), 3.63 (d, J = 13.4 Hz, 4H), 3.41 (d, J = 13.4 Hz, 4H), 2.75-257 (m, 4H), 2.43-2.28 (m, 4H), 2.00-1.84 (m, 4H), 1.73-1.58 (m, 4H), 1.58-1.30 (m, 8H), 1.18-0.94 (s, 8H), 0.78 (t, J = 7.1 Hz, 12H); ^{13}C NMR(DMSO-d_6, 125 MHz, 343 K) δ (ppm) 179.3, 143.1, 139.3, 128.5 (q, J = 31.3 Hz, *C*-CF_3), 126.9, 123.2 (q, J = 270.6 Hz, *C*F_3), 120.6, 117.6, 59.9, 53.2, 52.0, 26.1, 25.2, 21.1, 11.3; IR (KBr) ν 2933, 2858, 1546, 1464, 1346,1319, 1224, 1169, 1127 cm^{-1}; HRMS (ESI^-) calc. for $[M-H]^-$ ($C_{58}H_{71}F_{12}N_8S_2^-$), 1171.5057, found 1171.5058; Anal. Calcd. for $C_{58}H_{72}F_{12}N_8S_2$: C, 59.37; H, 6.19; N, 9.55. Found: C, 59.41; H, 6.18; N, 9.50; $[\alpha]_D^{25}$ = + 73.6 (c = 0.50, $CHCl_3$).

Macrocycle M3

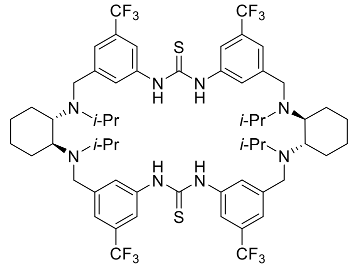

M3

Macrocycle **M3** was synthesized according to the follow procedure: **4c** (3.26 g, 6 mmol) and **5c** (3.77 g, 6 mmol) were dissolved in acetone (150 mL), and then Et_3N (1.9 mL, 15 mmol) was added. The mixture was heated at reflux for 6 days. The resulting mixture was cooled to room temperature and stand for 2 h. The precipitate was collected by filtration and redissolved in ethyl ether (40 mL), then sat. $NaHCO_3$ aq. (40 mL) was added and the resulting mixture was stirred at room temperature for 8 h. The organic layer was separated and the aqueous layer was extracted with ethyl ether (100 mL × 3). The combined organic layers were dried over Na_2SO_4, then concentrated to 4 mL under reduced pressure, and *n*-hexane (40 mL) was added. The precipitate was collected by filtration to give macrocycle **M3** as a yellow solid (2.44 g, yield: 35%). Mp 149-150 °C; ^1H NMR (DMSO-d_6, 500 MHz, 328 K) δ (ppm) 9.67 (s, 4H), 7.62 (s, 4H), 7.51 (s, 4H), 7.48 (s, 4H), 3.61 (d, J = 14.2 Hz, 4H), 3.51 (d, J = 14.2 Hz, 4H), 3.02-2.84 (m, 4H), 2.67-2.53 (m, 4H), 2.11-1.95 (m, 4H), 1.70-1.57 (m, 4H), 1.12-0.97 (m, 21H), 0.92 (d, J = 5.3 Hz, 12H); ^{13}C NMR (DMSO-d_6, 125 MHz, 328 K) δ (ppm) 179.9, 143.6, 139.4, 128.4 (q, J = 31.8 Hz, *C*-CF_3), 123.9 (q, J = 270.1 Hz, *C*F_3), 121.4, 118.2, 59.9, 47.6, 46.8, 28.4, 25.7, 22.9, 19.9; IR (KBr) ν 2928, 2855, 1659, 1608, 1462, 1343, 1225, 1167, 1126 cm^{-1}; HRMS (ESI^-) calc. for $[M-H]^-$ ($C_{58}H_{71}F_{12}N_8S_2^-$), 1171.5057, found 1171.5062; Anal. Calcd. for $C_{58}H_{72}F_{12}N_8S_2$: C, 59.37; H, 6.19; N, 9.55. Found: C, 59.38; H, 6.15; N, 9.53; $[\alpha]_D^{25}$ = + 60.8 (*c* = 0.50, CH_3OH).

Macrocycle M4

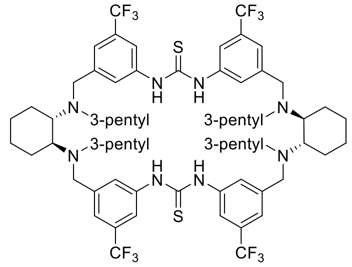

M4

Macrocycle **M4** was synthesized according to the general procedure by reaction of **4d** (601 mg, 1 mmol) and **5d** (685 mg, 0.5 mmol) in pyridine (400 mL) at room temperature for 96 h. Column chromatography eluent: petroleum ether/ethyl acetate 2:1. Macrocycle **M4** was obtained as a yellow solid (561 mg, yield: 42%). Mp

141.4-141.9 ºC; ^{1}H NMR (DMSO-d_6, 300 MHz) δ (ppm) 9.85 (s, 4H), 7.62 (s, 4H), 7.54 (s, 4H), 7.37 (s, 4H), 3.50 (s, 8H), 2.76-2.57 (m, 4H), 2.09-1.91 (m, 4H), 1.66-1.36 (m, 18H), 1.28-1.00 (m, 14H), 0.88 (t, *J* = 7.2 Hz, 12H), 0.80 (t, *J* = 7.2 Hz, 12H); ^{13}C NMR (DMSO-d_6, 125 MHz, 328K) δ (ppm) 179.3, 144.0, 139.4, 128.4 (q, *J* = 31.3 Hz, *C*-CF_3), 126.8, 123.9 (q, *J* = 270.6 Hz, *C*F_3), 120.7, 117.1, 60.5, 59.9, 49.0, 28.0, 26.2, 24.4, 23.8, 12.0, 11.1; IR (KBr) ν 3231, 2961, 2934, 2875, 1559, 1544, 1464, 1344, 1169, 1127 cm^{-1}; HRMS (ESI$^-$) calc. for [M-H]$^-$ ($C_{66}H_{87}F_{12}N_8S_2^-$), 1283.6309, found 1283.6300; Anal Calcd. for $C_{66}H_{88}F_{12}N_8S_2$: C, 61.66; H, 6.90; N, 8.72. Found: C, 61.56; H, 6.93; N, 8.47; $[\alpha]_D^{25}$ = +6.0 (*c* = 0.50, $CHCl_3$).

Macrocycle M6

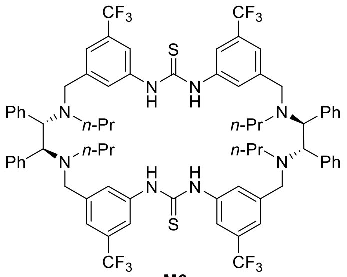

M6

Macrocycle **M6** was synthesized according to the general procedure by reaction of **4f** (1.61 g, 2.5 mmol), **5f** (1.81 g, 2.5 mmol) and DMAP (764 mg, 6.25 mmol) in THF (50 mL) at room temperature for 72 h. Column chromatography eluent: petroleum ether/ethyl acetate 5:1. Macrocycle **M6** was obtained as a white solid (1.51 g, yield: 44%). Mp 146-147 ºC; ^{1}H NMR (DMSO-d_6, 500 MHz, 343 K) δ (ppm) 9.45 (s, 4H), 7.67 (s, 8H), 7.52 (s, 4H), 7.21-6.92 (m, 20H), 4.59 (s, 4H), 4.06 (d, *J* = 14.4 Hz, 4H), 3.16 (d, *J* = 14.4 Hz, 4H), 2.84-2.61 (m, 4H), 2.29-2.09 (m, 4H), 1.77-1.52 (m, 8H), 0.87 (t, *J* = 6.4 Hz, 12H); ^{13}C NMR (DMSO-d_6, 125 MHz, 343 K) δ (ppm) 179.4, 142.4, 139.3, 136.1, 129.1, 128.6 (q, *J* = 31.6 Hz, *C*-CF_3), 127.6, 127.0, 126.2, 123.8 (q, *J* = 271.0 Hz, *C*F_3), 121.1, 118.2, 63.30, 53.4, 51.8, 20.5, 11.4; IR (KBr) ν 3220, 3029, 2961, 2932, 2874, 1534, 1463, 1344, 1225, 1171, 1128 cm^{-1}; HRMS (ESI$^-$) calc. for [M-H]$^-$ ($C_{74}H_{75}F_{12}N_8S_2^-$), 1367.5370, found 1367.5366; Anal. Calcd. for $C_{74}H_{76}F_{12}N_8S_2$: C, 64.60; H, 5.59; N, 8.18. Found: C, 64.76; H, 5.68; N, 8.05; $[\alpha]_D^{25}$ = +89.4 (*c* = 0.50, $CHCl_3$).

Macrocycle M9

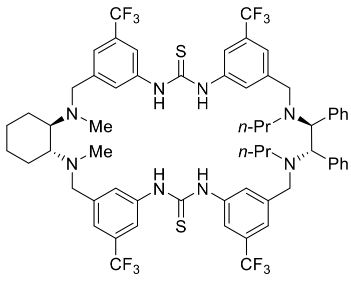

M9

Macrocycle **M9** was synthesized according to the general procedure by reaction of *ent*-**4a** (244 mg, 0.5 mmol), **5f** (363 mg, 0.5 mmol) and DMAP (153 mg, 1.25 mmol) in THF (10 mL) at room temperature for 48 h. Column chromatography eluent: dichloromethane/methanol 20:1. Macrocycle **M9** was obtained as a white solid (180 mg, yield: 30%). Mp 143-144 ºC; ^{1}H NMR (DMSO-d_6, 300 MHz) δ (ppm) 9.78 (d, 4H), 8.00-7.28 (m, 12H), 7.23-6.86 (m, 10H), 4.58 (s, 2H), 3.91 (d, J = 14.3 Hz, 2H), 3.82-3.51 (m, 4H), 3.13 (d, J = 14.4 Hz, 2H), 2.75-2.54 (m, 3H), 2.34-2.02 (m, 8H), 1.97-1.82 (m, 2H), 1.78-1.49 (m, 6H), 1.37-1.03 (m, 5H), 0.82 (t, J = 7.3 Hz, 6H); ^{13}C NMR (DMSO-d_6, 125 MHz) δ (ppm) 179.4, 142.7, 139.7, 139.6, 136.3, 129.4, 128.7 (q, J = 253.8 Hz, CF$_3$), 127.8, 127.3, 126.6, 125.22, 125.17, 123.1, 123.0, 121.3, 120.8, 120.7, 118.5, 118.0, 63.3, 62.0, 53.4, 51.0, 31.0, 25.3, 22.1, 20.8, 14.0, 11.8; IR (KBr) ν 3214, 2931, 1534, 1461, 1344, 1225, 1169, 1127 cm^{-1}; HRMS (ESI$^+$) calc. for [M+H]$^+$ ($C_{62}H_{67}F_{12}N_8S_2^+$), 1215.4733, found 1215.4717; Anal. Calcd. for $C_{62}H_{66}F_{12}N_8S_2$: C, 61.27; H, 5.47; N, 9.22. Found: C, 61.20; H, 5.46; N, 9.10; $[\alpha]_D^{25}$ = +44.8 (c = 0.50, $CHCl_3$).

Macrocycle M10

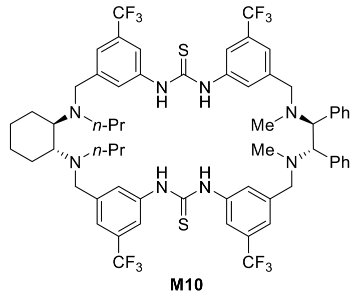

M10

Macrocycle **M10** was synthesized according to the general procedure by reaction of *ent*-**4b** (272 mg, 0.5 mmol), **5e** (353 mg, 0.5 mmol) and DMAP (153 mg, 1.25 mmol) in THF (10 mL) at room temperature for 48 h. Column chromatography eluent: dichloromethane/methanol 20:1. Macrocycle **M10** was obtained as a white solid (182 mg, yield: 30%). Mp 145 ºC; ^{1}H NMR (DMSO-d_6, 400 MHz, 333 K) δ (ppm) 9.66 (s, 4H), 7.82 (s, 2H), 7.74 (s, 2H), 7.63 (s, 2H), 7.53 (s, 2H), 7.49 (s, 2H), 7.29 (s, 2H), 7.24 (d, J = 7.6 Hz, 4H), 7.19 (t, 4H), 7.08 (d, J = 7.2 Hz, 2H), 4.63 (s, 2H), 3.74-3.58 (m, 4H), 3.51 (d, J = 13.7 Hz, 2H), 3.41 (d, J = 14.4 Hz, 2H), 3.17 (s, 3H), 2.67-2.58 (m, 2H),

2.57-2.50 (m, 2H), 2.43-2.31 (m, 2H), 2.20 (s, 6H), 2.02-1.90 (m, 2H), 1.77-1.63 (m, 2H), 1.55-1.34 (m, 4H), 1.09 (s, 4H), 0.79 (t, 6H); ^{13}C NMR (DMSO-*d*$_6$, 125 MHz, 328K) δ (ppm) 178.9, 143.1, 141.8, 139.5, 139.3, 135.8, 129.1, 128.4 (q, *J* = 253.8 Hz, *C*F$_3$), 127.2, 125.0 124.9, 122.87, 122.77, 120.8, 117.8, 117.6, 66.6, 59.7, 56.9, 53.0, 51.8, 36.4, 25.5, 25.3, 21.2, 11.5; IR (KBr) ν 3030, 2932, 2853, 1544, 1463, 1344, 1224, 1127 cm^{-1}; HRMS (ESI$^+$) calc. for [M+H]$^+$ ($C_{62}H_{67}F_{12}N_8S_2^+$), 1215.4733, found 1215.4718. Anal. Calcd. for $C_{62}H_{66}F_{12}N_8S_2$: C, 61.27; H, 5.47; N, 9.22. Found: C, 60.91; H, 5.53; N, 9.25; $[\alpha]_D^{25}$ = -22.4 (*c* = 0.50, CHCl$_3$).

Macrocycle M11

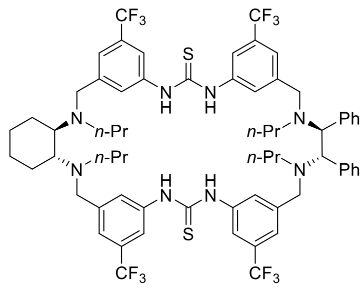

M11

Macrocycle **M11** was synthesized according to the general procedure by reaction of *ent*-**4b** (272 mg, 0.5 mmol), **5f** (363 mg, 0.5 mmol) and DMAP (153 mg, 1.25 mmol) in THF (10 mL) at room temperature for 96 h. Column chromatography eluent: dichloro-methane/methanol 20:1. Macrocycle **M11** was obtained as a white solid (203 mg, yield: 32%). Mp 136-137 °C; ^{1}H NMR (DMSO-*d*$_6$, 400 MHz, 333 K) δ (ppm) 9.62 (d, *J* = 22.7 Hz, 4H), 7.77 (s, 2H), 7.72 (s, 2H), 7.63 (s, 2H), 7.50 (s, 4H), 7.43 (s, 2H), 7.16-7.07 (m, 8H), 7.07-7.01 (m, 2H), 4.58 (s, 2H), 3.98 (d, *J* = 14.6 Hz, 2H), 3.65 (d, *J* = 14.3 Hz, 2H), 3.41 (d, *J* = 14.3 Hz, 2H), 3.23-3.17 (m, 2H), 2.76-2.60 (m, 4H), 2.45-2.30 (m, 2H), 2.22-2.09 (m, 2H), 2.02-1.91 (m, 2H), 1.72-1.55 (m, 6H), 1.52-1.37 (m, 4H), 1.19-0.99 (m, 4H), 0.95-0.60 (m, 14H); ^{13}C NMR (DMSO-*d*$_6$, 125 MHz, 328K) δ (ppm) 179.2, 143.1, 142.5, 139.5, 139.2, 136.3, 129.2, 128.5, 128.4, 127.2, 126.3, 125.1, 125.0, 122.8, 121.0, 120.9, 118.3, 117.7, 63.3, 59.7, 53.5, 53.0, 51.8, 51.7, 25.3, 21.2, 20.6, 11.53, 11.51; IR (KBr) ν 3214, 2960, 2933, 1539, 1463, 1345, 1225, 1170, 1127 cm^{-1}; HRMS (ESI$^+$) calc. for [M+H]$^+$ ($C_{66}H_{75}F_{12}N_8S_2^+$), 1271.5359, found 1271.5348; Anal. Calcd. for $C_{66}H_{74}F_{12}N_8S_2$: C, 62.35; H, 5.87; N, 8.81. Found: C, 61.95; H, 5.78; N, 8.93; $[\alpha]_D^{25}$ = +22.6 (*c* = 0.50, CHCl$_3$).

Macrocycle M12

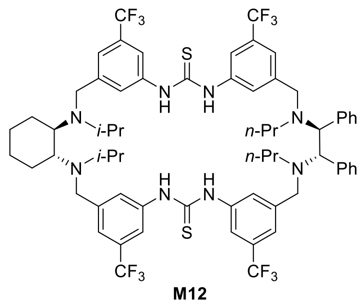

M12

Macrocycle **M12** was synthesized according to the general procedure by reaction of *ent*-**4c** (272 mg, 0.5 mmol), **5f** (363 mg, 0.5 mmol) and DMAP (153 mg, 1.25 mmol) in THF (10 mL) at room temperature for 48 h. Column chromatography eluent: dichloromethane/methanol 20:1. Macrocycle **M12** was obtained as a white solid (300 mg, yield: 47%). Mp 144-145 ºC; ^{1}H NMR (DMSO-d_6, 400 MHz, 328 K) δ (ppm) 9.54 (s, 4H), 7.75-7.42 (m, 12 H), 7.15-7.05 (m, 10 H), 4.59 (s, 2H), 3.99-3.97 (m, 2H), 3.51-3.48 (m , 4H), 3.20-3.07 (m, 4H), 2.78-2.65 (m, 4H), 2.15-2.03 (m, 4H), 1.64-1.62 (m, 6H), 1.14-1.07 (m, 8H), 0.97-0.96 (m, 6H), 0.85-0.82 (m, 8H); ^{13}C NMR (DMSO-d_6, 125 MHz, 328K) δ (ppm) 179.0, 143.7, 142.5, 141.7, 139.5, 138.2, 136.3, 129.3, 129.2, 128.5, 128.2, 127.2, 126.3, 125.1, 125.0, 122.9, 122.8, 121.3, 121.0, 63.3, 59.2, 53.5, 53.1, 51.8, 47.6, 28.6, 25.7, 22.5, 20.7, 19.9, 11.5; IR (KBr) ν 3202, 2962, 2873, 1549, 1464, 1344, 1318, 1224, 1169, 1127 cm^{-1}; HRMS (ESI$^+$) calc. for $[M+H]^+$ ($C_{66}H_{75}F_{12}N_8S_2^+$), 1271.5359, found 1271.5349; Anal. Calcd. for $C_{66}H_{74}F_{12}N_8S_2$: C,62.35; H, 5.87; N, 8.81. Found: C, 62.27; H, 5.75; N, 8.76. $[\alpha]_D^{25}$ = +8.8 (c = 0.50, CH_3OH).

2.5 Synthesis of acyclic control compound 9

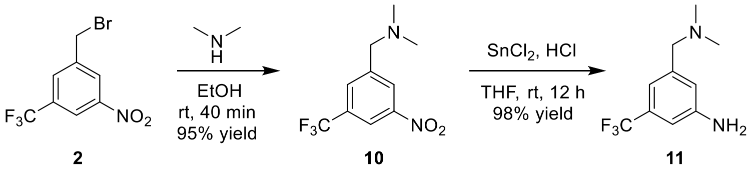

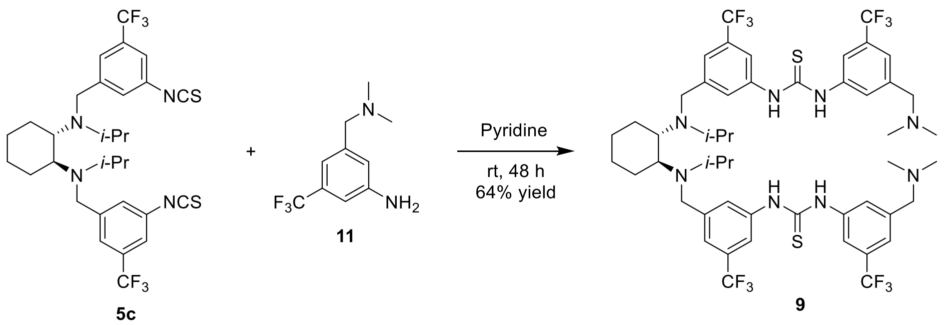

***N,N*-Dimethyl-1-(3-nitro-5-(trifluoromethyl)phenyl)methanamine (10):** To a solution of **2** (5.66 g, 20 mmol) in EtOH (30 mL) was added 30 wt % dimethylamine aqueous solution (13.60 g, 100 mmol). The mixture was stirred at room temperature for 40 min. The solvent was evaporated under reduced pressure, and the residue was subjected to column chromatography on silica gel (petroleum ether/ethyl acetate 1:1) to give **10**[6] as a yellow oil (4.75 g, yield: 95%). ^{1}H NMR ($CDCl_3$, 400 MHz) δ (ppm) 8.39 (s, 1H), 8.37 (s, 1H), 7.95 (s, 1H), 3.58 (s, 2H), 2.28 (s, 6H).

3-((Dimethylamino)methyl)-5-(trifluoromethyl)aniline (11): To a solution of **10** (9.18 g, 37 mmol) in THF (500 mL) was added a solution of $SnCl_2·2H_2O$ (26.71 g, 118 mmol) in *conc.* hydrochloric acid (44 mL). The mixture was stirred at room temperature for 12 h, and then was treated with a solution of 40% aq. NaOH to adjust pH > 10. The organic layer was separated and the aqueous layer was extracted with ethyl acetate (100 mL × 3). The combined organic layers were dried over Na_2SO_4, and then evaporated under reduced pressure. The residue was subjected to column chromatography on silica gel (dichloromethane/methanol 5:1) to give **11**[6] as a yellow solid (8.08 g, yield: 98%). ^{1}H NMR ($CDCl_3$, 400 MHz) δ (ppm) 6.91 (s, 1H), 6.80 (s, 1H), 6.77 (s, 1H), 3.81 (s, br, 2H), 3.34 (s, 2H), 2.23 (s, 6H).

Acyclic control compound 9: To a solution of **5c** (628 mg, 1 mmol) in pyridine (10 mL) was added **11** (436 mg, 2 mmol). The mixture was stirred at room temperature for 48 h. The solvent was evaporated under reduced pressure, and the residue was subjected to column chromatography on silica gel (dichloromethane/methanol 5:1) to give **9** as a white solid (680 mg, yield: 64%). Mp 104-105 °C; ^{1}H NMR (DMSO-d_6, 400 MHz) δ (ppm) 10.25 (s, 4H), 7.83 (s, 2H), 7.76-7.60 (m, 6H), 7.40 (d, J = 11.4 Hz, 4H), 3.63 (d, J = 13.0 Hz, 2H), 3.51 (s, 4H), 3.45 (d, J = 14.1 Hz, 2H), 2.77-2.62 (m, 2H), 2.20 (s, 12H), 2.12-2.02 (m, 2H), 1.66-1.47 (m, 2H), 1.18-0.68 (m, 18H); 10.15 (s, 4H), 7.82 (s, 2H), 7.70 (s, 2H), 7.68 (s, 2H), 7.64 (s, 2H), 7.54 (s, 2H), 7.36 (s, 2H), 7.25-7.19 (m, 4H), 7.16 (t, J = 7.5 Hz, 4H), 7.08 (t, J = 7.2 Hz, 2H), 4.62 (s, 2H), 3.62 (d, J = 13.7 Hz, 2H), 3.57-3.41 (m, 6H), 2.30-2.10 (m, 18H); ^{13}C NMR (DMSO-d_6, 125 MHz) δ

(ppm) 180.0, 149.4, 140.2, 139.6, 129.1, 128.9, 128.4, 127.3, 124.1 (q, J = 270.8 Hz, CF_3), 124.0 (q, J = 270.8 Hz, CF_3), 124.5, 120.8, 118.5, 117.9, 82.2, 59.3, 47.0, 44.7, 27.6, 26.0, 23.3, 19.8; HRMS (ESI$^-$) calc. for $[M-H]^-$ ($C_{50}H_{59}F_{12}N_8S_2^-$), 1063.4118, found 1063.4126; IR (KBr) ν 2940, 2860, 2780, 1545, 1465, 1344, 1170 cm^{-1}; $[\alpha]_D^{25}$ = +14.6 (c = 0.50, $CHCl_3$).

3. Catalysis studies

All the ketimines **6**[7] and malonic acid half thioesters (MAHTs) **7**[8-9] are known compounds and were prepared according to literature procedures.

3.1 Typical procedure for the decarboxylative Mannich reactions

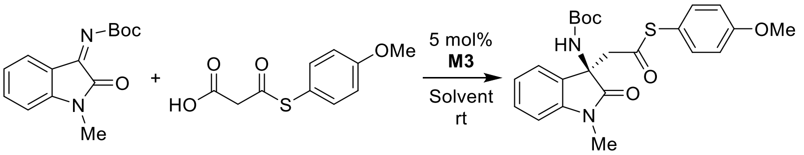

6a **7a** **8a**

Typical procedure: The ketimine **6a** (52.1 mg, 0.2 mmol) and the macrocycle catalyst **M3** (11.8 mg, 0.01 mmol) was added into a 10 mL Schlenk flask equipped with a stirring bar, then dried cyclopentyl methyl ether (CPME, 2.0 mL) and malonic acid half thioester **7a** (67.9 mg, 0.3 mmol) were added. The resulting reaction mixture was stirred at room temperature until full consumption of the starting ketimine **6a** as indicated by TLC. The reaction mixture was concentrated under reduced pressure and the residue was subjected to column chromatography on silica gel (petroleum ether/ethyl acetate 3:1) to give the desired product **8a** as a white solid.

3.2 Characterization data for products 8

S-(4-Methoxyphenyl) (R)-2-(3-((tert-butoxycarbonyl)amino)-1-methyl-2-oxoindolin-3-yl)ethanethioate (8a)[9]

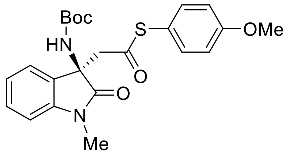

8a

88% yield; ^{1}H NMR ($CDCl_3$, 500 MHz) δ (ppm) 7.37-7.31 (m, 1H), 7.30-7.22 (m, 3H), 7.07 (t, *J* = 7.5 Hz, 1H), 6.95 (d, *J* = 8.9 Hz, 2H), 6.87 (d, *J* = 7.6 Hz, 1H), 6.31 (s, 1H), 3.83 (s, 3H), 3.27 (s, 3H), 3.19 (d, *J* = 15.0 Hz, 1H), 2.78 (d, *J* = 15.1 Hz, 1H), 1.24 (s, 9H); ^{13}C NMR ($CDCl_3$, 125 MHz) δ (ppm) 196.8, 175.2, 161.2, 153.8, 143.3, 136.1, 129.5, 129.0, 123.7, 122.8, 117.5, 115.2, 108.6, 80.4, 60.3, 55.6, 47.6, 28.2, 26.9; $[\alpha]_D^{25}$ = +99.6 (*c* = 0.50, $CHCl_3$); *ee*: 72%; HPLC analysis CHIRALPAK OD-H, *n*-hexane:^{i}PrOH = 4:1, 25 °C, 1 mL/min flow rate, detection at 254 nm, t_1 = 9.7 min (major), t_2 = 12.1 min (minor).

S-(4-Methoxyphenyl) (R)-2-(3-((tert-butoxycarbonyl)amino)-2-oxoindolin-3-yl)ethanethioate (8b)

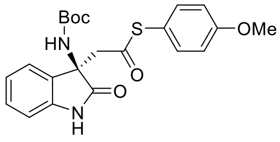

8b

Yellow solid, 48% yield; Mp 158-159 ºC; ^{1}H NMR ($CDCl_3$, 400 MHz) δ (ppm) 7.77 (s, 1H), 7.28-7.23 (m, 4H), 7.05 (t, *J* = 7.5 Hz, 1H), 6.95 (d, *J* = 8.7 Hz, 2H), 6.87 (d, *J* = 7.7 Hz, 1H), 6.42 (s, 1H), 3.84 (s, 3H), 3.16 (d, *J* = 15.0 Hz, 1H), 2.84 (d, *J* = 15.2 Hz, 1H), 1.28 (s, 9H); ^{13}C NMR ($CDCl_3$, 100 MHz) δ (ppm) 176.5, 161.3, 154.0, 140.3, 136.2, 129.5, 124.0, 122.9, 117.5, 115.2, 110.5, 80.7, 60.5, 55.6, 47.4, 28.2; IR (KBr) ν 3278, 2976, 2929, 1690, 1623, 1593, 1497, 1253, 1161, 1026, 827, 751 cm^{-1}; HRMS (ESI^+) calc. for $[M+Na]^+$ ($C_{22}H_{24}N_2O_5SNa^+$), 451.1298, found 451.1294; $[\alpha]_D^{25}$ = +47.2 (*c* = 0.50, $CHCl_3$); *ee*: 40%; HPLC analysis CHIRALPAK AD-H, *n*-hexane:^{i}PrOH = 9:1, 25 °C, 1 mL/min flow rate, detection at 254 nm, t_1 = 10.8 min (major), t_2 = 13.3 min (minor).

S-(4-Methoxyphenyl) (R)-2-(3-((tert-butoxycarbonyl)amino)-1-ethyl-2-oxoindolin-3-yl)ethanethioate (8c)

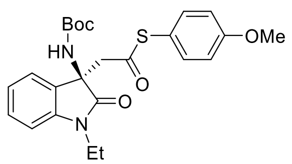

8c

Yellow solid, 73% yield; Mp 164-165 °C; ^{1}H NMR ($CDCl_3$, 300 MHz) δ (ppm) 7.35-7.22 (m, 4H), 7.06 (t, *J* = 7.4 Hz, 1H), 6.95-6.87 (m, 3H), 6.24 (s, 1H), 3.94-3.59 (m, 5H), 3.18 (d, *J* = 15.1 Hz, 1H), 2.76 (d, *J* = 15.0 Hz, 1H), 1.33-1.25 (m, 12H); ^{13}C NMR ($CDCl_3$, 75 MHz) δ (ppm) 196.6, 174.8, 161.2, 153.8, 142.4, 136.1, 129.4, 129.3, 124.0, 122.6, 117.7, 115.2, 108.7, 80.3, 60.3, 55.5, 47.7, 35.3, 28.2, 12.7; IR (KBr) ν 3251, 2977, 2931, 1727, 1615, 1592, 1496, 1368, 1251, 1160, 1024, 751 cm^{-1}; HRMS (ESI^+) calc. for $[M+Na]^+$ ($C_{24}H_{28}N_2O_5S\ Na^+$), 479.1611, found 479.1610; $[\alpha]_D^{25}$ = +58.8 (*c* = 0.50, $CHCl_3$); *ee*: 64%; HPLC analysis CHIRALPAK IA, *n*-hexane:^{i}PrOH = 4:1, 25 °C, 1 mL/min flow rate, detection at 254 nm, t_1 = 8.7 min (major), t_2 = 17.8 min (minor).

S-(4-Methoxyphenyl) (R)-2-(3-((tert-butoxycarbonyl)amino)-1-isopropyl-2-oxoindolin-3-yl)ethanethioate (8d)

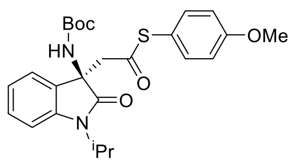

8d

Yellow solid, 43% yield; Mp 129-130 °C; ^{1}H NMR ($CDCl_3$, 300 MHz) δ (ppm) 7.33-7.20 (m, 4H), 7.03 (t, *J* = 8.3 Hz, 2H), 6.93 (d, *J* = 8.9 Hz, 2H), 6.19 (s, 1H), 4.60 (m, 1H), 3.82 (s, 3H), 3.17 (d, *J* = 15.0 Hz, 1H), 2.76 (d, *J* = 14.9 Hz, 1H), 1.51 (d, *J* = 7.0 Hz, 6H), 1.27 (s, 9H); ^{13}C NMR ($CDCl_3$, 75 MHz) δ (ppm) 196.4, 174.9, 161.2, 153.8, 142.2, 136.1, 129.5, 129.1, 124.1, 122.2, 117.8, 115.2, 110.2, 80.2, 60.1, 55.5, 47.8, 44.6, 28.3, 19.6, 19.3; IR (KBr) ν 3262, 2978, 2931, 1718, 1611, 1591, 1496, 1367, 1250, 1161, 1025, 827, 753 cm^{-1}; HRMS (ESI^+) calc. for $[M+Na]^+$ ($C_{25}H_{30}N_2O_5SNa^+$), 493.1768, found 493.1763; $[\alpha]_D^{25}$ = +37.9 (*c* = 0.48, $CHCl_3$); *ee*: 53%; HPLC analysis CHIRALPAK IA, *n*-hexane:^{i}PrOH = 4:1, 25 °C, 1 mL/min flow rate, detection at 254 nm, t_1 = 8.1 min (major), t_2 = 17.7 min (minor).

S-(4-Methoxyphenyl) (R)-2-(1-benzyl-3-((tert-butoxycarbonyl)amino)-2-oxoindolin-3-yl)ethanethioate (8e)

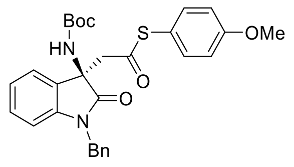

8e

White solid, 46% yield; Mp 142-143 ºC; ^{1}H NMR ($CDCl_3$, 500 MHz) δ (ppm) 7.38 (d, J = 7.3 Hz, 2H), 7.34-7.24 (m, 6H), 7.20 (t, J = 7.1 Hz, 1H), 7.03 (t, J = 7.5 Hz, 1H), 6.96-6.93 (m, 2H), 6.72 (d, J = 7.8 Hz, 1H), 5.10-4.83 (m, 2H), 3.83 (s, 3H), 3.21 (d, J = 14.8 Hz, 1H), 2.82 (d, J = 15.0 Hz, 1H), 1.28 (d, J = 12.8 Hz, 9H); ^{13}C NMR ($CDCl_3$, 125 MHz) δ (ppm) 196.7, 175.3, 161.2, 153.8, 142.4, 136.2, 135.9, 129.4, 128.9, 127.7, 127.5, 123.7, 122.9, 117.6, 115.2, 109.6, 80.5, 60.4, 55.6, 47.7, 44.4, 28.3; IR (KBr) ν 3330, 2976, 2928, 1711, 1614, 1593, 1497, 1366, 1251, 1172, 1024, 828, 734 cm^{-1}; HRMS (ESI$^+$) calc. for $[M+Na]^+$ ($C_{29}H_{30}N_2O_5SNa^+$), 541.1768, found 541.1765; $[\alpha]_D^{25}$ = +24.2 (c = 0.50, $CHCl_3$); *ee*: 37%; HPLC analysis CHIRALPAK OD-H, *n*-hexane:^{i}PrOH = 7:3, 25 °C, 1 mL/min flow rate, detection at 254 nm, t_1 = 8.9 min (minor), t_2 = 10.5 min (major).

S-(4-Methoxyphenyl) (R)-2-(3-((tert-butoxycarbonyl)amino)-1-(methoxymethyl)-2-oxoindolin-3-yl)ethanethioate (8f)

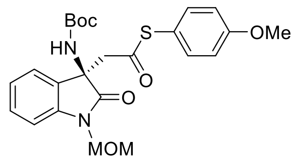

8f

Yellow solid, 40% yield; Mp 114-115 ºC; ^{1}H NMR ($CDCl_3$, 400 MHz) δ (ppm) 7.35-7.27 (m, 2H), 7.24 (s, 1H), 7.08 (t, J = 7.5 Hz, 2H), 6.95 (d, J = 8.6 Hz, 2H), 6.37 (s, 1H), 5.21 (d, J = 11.0 Hz, 1H), 5.14 (d, J = 11.3 Hz, 1H), 3.83 (d, J = 1.1 Hz, 3H), 3.41 (s, 3H), 3.15 (d, J = 15.0 Hz, 1H), 2.81 (d, J = 15.0 Hz, 1H), 1.26 (s, 9H); ^{13}C NMR ($CDCl_3$, 100 MHz) δ (ppm) 196.6, 175.8, 161.3, 153.8, 141.6, 136.1, 129.6, 128.6, 123.7, 123.3, 117.6, 115.2, 110.0, 80.5, 72.1, 60.6, 56.7, 55.5, 47.8, 28.2; IR (KBr) ν 3251, 2977, 2931, 1727, 1615, 1592, 1496, 1368, 1251, 1160, 1024, 834, 751 cm^{-1}; HRMS (ESI$^+$) calc. for $[M+Na]^+$ ($C_{24}H_{28}N_2O_6SNa^+$), 495.1560, found 495.1559; $[\alpha]_D^{25}$ = +33.8 (c = 0.50, $CHCl_3$); *ee*: 45%; HPLC analysis CHIRALPAK IA, *n*-hexane:^{i}PrOH = 4:1, 25 °C, 1 mL/min flow rate, detection at 254 nm, t_1 = 8.1 min (major), t_2 = 17.7 min (minor).

S-(4-Methoxyphenyl) (R)-2-(1-acetyl-3-((tert-butoxycarbonyl)amino)-2-oxoindolin-3-yl)ethanethioate (8g)

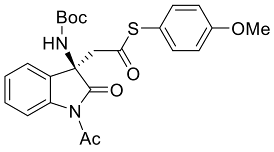

8g

Yellow solid, 35% yield; Mp 157-158 ºC; ^{1}H NMR ($CDCl_3$, 400 MHz) δ (ppm) 8.27 (d, *J* = 8.1 Hz, 1H), 7.39 (t, *J* = 7.8 Hz, 1H), 7.30-7.28 (m, 1H), 7.24-7.20 (m, 3H), 6.94 (d, *J* = 8.9 Hz, 2H), 6.35 (s, 1H), 3.83 (s, 3H), 3.13 (d, *J* = 14.9 Hz, 1H), 2.92 (d, *J* = 14.8 Hz, 1H), 2.71 (s, 3H), 1.23 (s, 9H); ^{13}C NMR ($CDCl_3$, 100 MHz) δ (ppm) 195.8, 175.8, 170.9, 161.3, 153.8, 139.8, 136.1, 129.9, 128.3, 125.4, 123.0, 117.2, 116.9, 115.3, 81.2, 60.7, 55.5, 48.3, 28.1, 26.8; IR (KBr) ν 3377, 2960, 2929, 1751, 1715, 1592, 1497, 1375, 1250, 1018, 771 cm^{-1}; HRMS (ESI$^+$) calc. for $[M+Na]^+$ ($C_{24}H_{26}N_2O_6SNa^+$), 493.1404, found 193.1399; $[\alpha]_D^{25}$ = -1.2 (*c* = 0.42, $CHCl_3$); *ee*: 16%; HPLC analysis CHIRALPAK IA, *n*-hexane:^{i}PrOH = 4:1, 25 °C, 1 mL/min flow rate, detection at 254 nm, t_1 = 22.1 min (major), t_2 = 29.5 min (minor).

S-(4-Methoxyphenyl) (R)-2-(3-(((benzyloxy)carbonyl)amino)-1-methyl-2-oxoindolin-3-yl)ethanethioate (8h)

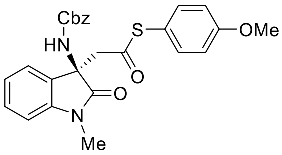

8h

White solid, 73% yield; Mp 56-57 ºC; ^{1}H NMR ($CDCl_3$, 500 MHz) δ (ppm) 7.35 (t, *J* = 7.7 Hz, 1H), 7.30-7.23 (m, 8H), 7.08 (t, *J* = 7.5 Hz, 1H), 6.94 (d, *J* = 8.8 Hz, 2H), 6.87 (s, 1H), 6.66 (s, 1H), 4.92 (s, 2H), 3.82 (s, 3H), 3.24 (d, *J* = 15.1 Hz, 4H), 2.82 (d, *J* = 15.4 Hz, 1H); ^{13}C NMR ($CDCl_3$, 125 MHz) δ (ppm) 196.9, 174.7, 161.2, 154.5, 143.4, 136.1, 135.8, 129.8, 128.5, 128.2, 128.3, 128.1, 123.9, 123.0, 117.3, 115.2, 108.7, 67.2, 60.3, 55.5, 47.3, 26.8; IR (KBr) ν 3263, 3029, 2958, 1729, 1697, 1615, 1591, 1252, 1024, 829, 751 cm^{-1}; HRMS (ESI$^+$) calc. for $[M+Na]^+$ ($C_{26}H_{24}N_2O_5SNa^+$), 499.1298, found 499.1293; $[\alpha]_D^{25}$ = +30.0 (*c* = 0.46, $CHCl_3$); *ee*: 26%; HPLC analysis CHIRALPAK OD-H, *n*-hexane:^{i}PrOH = 4:1, 25 °C, 1 mL/min flow rate, detection at 254 nm, t_1 = 20.9 min (major), t_2 = 28.0 min (minor).

S-(4-Methoxyphenyl) (R)-2-(3-((tert-butoxycarbonyl)amino)-1,5-dimethyl-2-oxoindolin-3-yl)ethanethioate (8i)

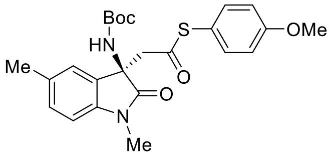

8i

White solid, 76% yield; Mp 165-166 °C; ^1H NMR ($CDCl_3$, 300 MHz) δ (ppm) 7.27-7.22 (m, 2H), 7.14-7.09 (m, 2H), 6.98-6.93 (m, 2H), 6.75 (d, J = 7.8 Hz, 1H), 3.83 (s, 3H), 3.24 (s, 3H), 3.16 (d, J = 14.9 Hz, 1H), 2.75 (d, J = 14.9 Hz, 1H), 2.34 (s, 3H), 1.24 (s, 9H); ^{13}C NMR ($CDCl_3$, 750 MHz) δ (ppm) 196.8, 175.0, 161.2, 153.7, 140.9, 136.1, 132.3, 129.7, 128.9, 124.4, 117.6, 115.2, 108.3, 80.3, 60.3, 55.5, 47.6, 28.2, 26.8, 21.3; IR (KBr) ν 3276, 2977, 2932, 1706, 1625, 1592, 1496, 1369, 1250, 1173, 1029, 832, 754 cm^{-1}; HRMS (ESI^+) calc. for $[M+Na]^+$ ($C_{24}H_{28}N_2O_5SNa^+$), 479.1611, found 479.1606; $[\alpha]_D^{25}$ = +102.0 (c = 0.50, $CHCl_3$); *ee*: 74%; HPLC analysis CHIRALPAK OD-H, *n*-hexane:^{i}PrOH = 9:1, 25 °C, 1 mL/min flow rate, detection at 254 nm, t_1 = 18.7 min (major), t_2 = 21.9 min (minor).

S-(4-Methoxyphenyl) (R)-2-(3-((tert-butoxycarbonyl)amino)-5-methoxy-1-methyl-2-oxoindolin-3-yl)ethanethioate (8k)

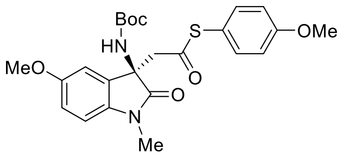

8k

White solid, 82% yield; Mp 143-144 °C; ^1H NMR ($CDCl_3$, 400 MHz) δ (ppm) 7.27-7.25 (m, 2H), 6.97-6.91 (m, 3H), 6.87-6.84 (m, 1H), 6.77 (d, J = 8.4 Hz, 1H), 6.30 (s, 1H), 3.83 (s, 3H), 3.78 (s, 3H), 3.24 (s, 3H), 3.18 (d, J = 15.3 Hz, 1H), 2.77 (d, J = 15.2 Hz, 1H), 1.26 (s, 9H); ^{13}C NMR ($CDCl_3$, 100 MHz) δ (ppm) 196.7, 174.8, 161.2, 156.2, 153.7, 136.7, 136.1, 130.2, 117.5, 115.2, 113.9, 111.1, 108.9, 80.4, 56.1, 55.5, 47.6, 28.2, 26.9; IR (KBr) ν 3324, 2975, 2935, 2837, 1720, 1593, 1497, 1367, 1251, 1173, 1030, 828 cm^{-1}; HRMS (ESI^+) calc. for $[M+Na]^+$ ($C_{24}H_{28}N_2O_6SNa^+$), 495.1560, found 495.1556; $[\alpha]_D^{25}$ = +101.8 (c = 0.50, $CHCl_3$); *ee*: 72%; HPLC analysis CHIRALPAK OD-H, *n*-hexane:^{i}PrOH = 4:1, 25 °C, 1 mL/min flow rate, detection at 254 nm, t_1 = 11.4 min (major), t_2 = 14.0 min (minor).

S-(4-Methoxyphenyl) (R)-2-(3-((tert-butoxycarbonyl)amino)-6-fluoro-1-methyl-2-oxoindolin-3-yl)ethanethioate (8l)

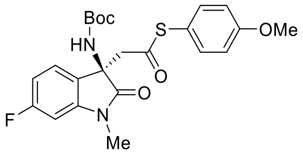

8l

White solid, 88% yield; Mp 198-199 °C; ^{1}H NMR ($CDCl_3$, 400 MHz) δ (ppm) 7.26-7.22 (m, 3H), 6.95 (d, J = 8.5 Hz, 2H), 6.74 (t, J = 8.6 Hz, 1H), 6.60 (d, J = 7.5 Hz, 1H), 6.24 (s, 1H), 3.83 (s, 3H), 3.24-3.19 (m, 4H), 2.78 (d, J = 15.3 Hz, 1H), 1.26 (s, 9H); ^{13}C NMR ($CDCl_3$, 100 MHz) δ (ppm) 196.4, 175.4, 163.9 (d, J = 246.5 Hz, *C*-F), 161.3, 153.7, 145.1 (d, J = 11.7 Hz, *C*-C-C-F), 136.1, 124.3, 125.0 (d, J = 9.9 Hz, *C*-C-C-F), 117.4, 115.2, 108.8 (d, J = 22.4 Hz, *C*-C-F), 97.5 (d, J = 27.5 Hz, *C*-C-F), 80.6, 59.8, 55.5, 47.7, 28.2, 27.0; IR (KBr) ν 3327, 2977, 2914, 1729, 1616, 1497, 1383, 1250, 1173, 1086, 830 cm^{-1}; HRMS (ESI$^+$) calc. for $[M+Na]^+$ ($C_{23}H_{25}FN_2O_5SNa^+$), 483.1360, found 483.1356; $[\alpha]_D^{25}$ = +105.6 (c = 0.50, $CHCl_3$); *ee*: 70%; HPLC analysis CHIRALPAK OD-H, *n*-hexane:^{i}PrOH = 4:1, 25 °C, 1 mL/min flow rate, detection at 254 nm, t_1 = 9.2 min (major), t_2 = 12.6 min (minor).

S-(4-Methoxyphenyl) (R)-2-(3-((tert-butoxycarbonyl)amino)-7-fluoro-1-methyl-2-oxoindolin-3-yl)ethanethioate (8m)

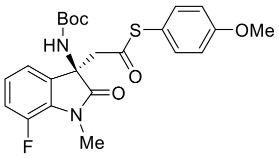

8m

White solid, 85% yield; Mp 177-178 °C; ^{1}H NMR ($CDCl_3$, 400 MHz) δ (ppm) 7.25 (d, J = 9.7 Hz, 2H), 7.08-6.98 (m, 3H), 6.95 (d, J = 8.9 Hz, 2H), 6.33 (s, 1H), 3.83 (s, 3H), 3.47 (d, J = 2.8 Hz, 3H), 3.17 (d, J = 15.2 Hz, 1H), 2.78 (d, J = 15.2 Hz, 1H), 1.26 (s, 9H); ^{13}C NMR($CDCl_3$, 100 MHz) δ (ppm) 196.5, 174.9, 161.3, 153.7, 148.0 (d, J = 244.3 Hz, *C*-F), 136.1, 131.9, 130.0 (d, J = 8.4 Hz, *C*-C-C-F), 123.3 (d, J = 6.2 Hz, *C*-C-C-F), 119.4 (d, J = 3.3 Hz, *C*-C-C-C-F), 117.5 (d, J = 19.4 Hz, *C*-C-F), 117.3, 115.2, 80.6, 60.3, 55.5, 47.5, 29.4, 29.4, 28.2; IR (KBr) ν 3334, 2976, 2940, 1728, 1632, 1593, 1496, 1249, 1026, 828 cm^{-1}; HRMS (ESI$^+$) calc. for $[M+Na]^+$ ($C_{23}H_{25}FN_2O_5SNa^+$), 483.1860, found 483.1355; $[\alpha]_D^{25}$ = +102.4 (c = 0.50, $CHCl_3$); *ee*: 74%; HPLC analysis CHIRALPAK IA, *n*-hexane:^{i}PrOH = 4:1, 25 °C, 1 mL/min flow rate, detection at 254 nm, t_1 = 8.8 min (major), t_2 = 17.0 min (minor).

***S*-(4-Methoxyphenyl) (*R*)-2-(3-((*tert*-butoxycarbonyl)amino)-5,6-difluoro-1-methyl-2-oxoindolin-3-yl)ethanethioate (8n)**

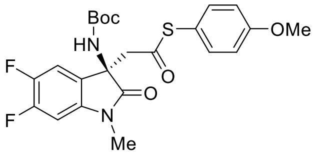

8n

white solid, 99% yield; Mp 149-150 °C; ^{1}H NMR ($CDCl_3$, 400 MHz) δ (ppm) 7.28-7.25 (m, 2H), 7.19-7.15 (m, 1H), 6.98-6.95 (m, 2H), 6.72-6.68 (m, 1H), 6.19 (s, 1H), 3.84 (s, 3H), 3.25-321 (m, 4H), 2.78 (d, *J* = 15.4 Hz, 1H), 1.28 (s, 9H); ^{13}C NMR ($CDCl_3$, 100 MHz) δ (ppm) 196.4, 175.0, 161.3, 153.7, 151.2 (dd, *J* = 247.1, 13.9 Hz, *C*-F), 146.7 (dd, *J* = 241.6, 13.1 Hz, *C*-F), 139.8 (d, *J* = 10.1 Hz, *C*-C-C-F), 136.2, 124.2, 117.1, 115.3, 113.9 (d, *J* = 20.1 Hz, *C*-C-F), 98.9 (d, *J* = 23.0 Hz, *C*-C-F), 80.8, 59.9, 55.6, 47.5, 28.2, 27.1; IR (KBr) ν 3337, 2978, 2935, 1718, 1626, 1593, 1510, 1396, 1252, 1173, 1029, 829, 783 cm^{-1}; HRMS (ESI^+) calc. for $[M+Na]^+$ ($C_{23}H_{24}F_2N_2O_5SNa^+$), 501.1266, found 501.1263; $[\alpha]_D^{25}$ = +99.6 (*c* = 0.50, $CHCl_3$); *ee*: 67%; HPLC analysis CHIRALPAK IA, *n*-hexane:^{i}PrOH = 4:1, 25 °C, 1 mL/min flow rate, detection at 254 nm, t_1 = 8.4 min (major), t_2 = 10.6 min (minor).

***S*-(4-Methoxyphenyl) (*R*)-2-(3-((*tert*-butoxycarbonyl)amino)-5-chloro-1-methyl-2-oxoindolin-3-yl)ethanethioate (8p)**

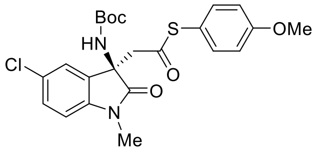

8p

White solid, 97% yield; Mp 181-182 °C; ^{1}H NMR ($CDCl_3$, 400 MHz) δ (ppm) 7.33-7.25 (m, 4H), 6.97 (d, *J* = 9.0 Hz, 2H), 6.80 (d, *J* = 8.2 Hz, 1H), 6.29 (s, 1H), 3.84 (s, 3H), 3.25 (s, 3H), 3.17 (d, *J* = 15.2 Hz, 1H), 2.75 (d, *J* = 15.0 Hz, 1H), 1.27 (s, 9H); ^{13}C NMR ($CDCl_3$, 100 MHz) δ (ppm) 196.6, 174.7, 161.3, 153.7, 141.9, 136.2, 130.4, 129.4, 128.2, 124.2, 117.2, 115.3, 109.6, 80.7, 60.1, 55.5, 47.3, 28.2, 27.0; IR (KBr) ν 3281, 2978, 2938, 1709, 1611, 1592, 1495, 1392, 1250, 1172, 1028, 831 cm^{-1}; HRMS (ESI^+) calc. for $[M+Na]^+$ ($C_{23}H_{25}ClN_2O_5SNa^+$), 499.1065, found 499.1061; $[\alpha]_D^{25}$ = +97.6 (*c* = 0.50, $CHCl_3$); *ee*: 72%; HPLC analysis CHIRALPAK OD-H, *n*-hexane:^{i}PrOH = 4:1, 25 °C, 1 mL/min flow rate, detection at 254 nm, t_1 = 9.5 min (major), t_2 = 11.9 min (minor).

S-(4-Methoxyphenyl) (R)-2-(3-((tert-butoxycarbonyl)amino)-6-chloro-1-methyl-2-oxoindolin-3-yl)ethanethioate (8q)

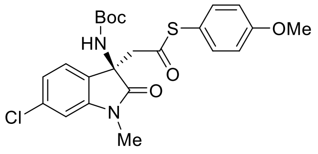

8q

White solid, 94% yield; Mp 196-197 ºC; ^{1}H NMR ($CDCl_3$, 500 MHz) δ (ppm) 7.26-7.24 (m, 2H), 7.20 (d, *J* = 7.9 Hz, 1H), 7.05-7.03 (m, 1H), 6.97-6.94 (m, 2H), 6.88-6.84 (m, 1H), 6.26 (s, 1H), 3.83 (s, 3H), 3.25 (s, 3H), 3.20 (d, *J* = 15.3 Hz, 1H), 2.77 (d, *J* = 15.3 Hz, 1H), 1.26 (s, 9H); ^{13}C NMR ($CDCl_3$, 125 MHz) δ (ppm) 196.5, 175.1, 161.3, 153.7, 144.6, 136.1, 135.3, 127.3, 124.7, 122.6, 117.2, 115.2, 109.4, 80.6, 59.8, 55.5, 47.5, 28.2, 27.0; IR (KBr) ν 3324, 2977, 2936,1729, 1612, 1497, 1369, 1250, 1173, 1031, 828 cm^{-1}; HRMS (ESI^{+}) calc. for $[M+Na]^+$ ($C_{23}H_{25}ClN_2O_5SNa^+$), 499.1065, found 499.1061; $[\alpha]_D^{25}$ = +115.0 (*c* = 0.50, $CHCl_3$); *ee*: 73%; HPLC analysis CHIRALPAK OD-H, *n*-hexane:^{i}PrOH = 4:1, 25 °C, 1 mL/min flow rate, detection at 254 nm, t_1 = 9.0 min (major), t_2 = 12.7 min (minor).

S-(4-Methoxyphenyl) (R)-2-(3-((tert-butoxycarbonyl)amino)-7-chloro-1-methyl-2-oxoindolin-3-yl)ethanethioate (8r)

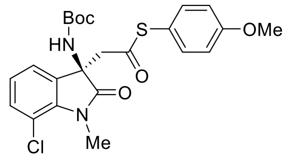

8r

White solid, 87% yield; Mp 169-170 ºC; ^{1}H NMR ($CDCl_3$, 400 MHz) δ (ppm) 7.26-7.23 (m, 3H), 7.16 (d, *J* = 7.3 Hz, 1H), 7.00-6.94 (m, 3H), 6.34 (s, 1H), 3.83 (s, 3H), 3.63 (s, 3H), 3.14 (d, *J* = 15.2 Hz, 1H), 2.76 (d, *J* = 15.2 Hz, 1H), 1.26 (s, 9H); ^{13}C NMR ($CDCl_3$, 100 MHz) δ (ppm) 196.6, 175.5, 161.3, 153.7, 139.2, 136.1, 131.9, 131.8, 123.5, 122.0, 117.3, 116.1, 115.2, 80.7, 59.9, 55.5, 47.5, 30.3, 28.2; IR (KBr) ν 3322, 2977, 2838, 1729, 1610, 1593, 1496, 1466, 1367, 1251, 1173, 1112, 1028, 828, 734 cm^{-1}; HRMS (ESI^{+}) calc. for $[M+Na]^+$ ($C_{23}H_{25}ClN_2O_5SNa^+$), 499.1065, found 499.1060; $[\alpha]_D^{25}$ = +104.0 (*c* = 0.50, $CHCl_3$); *ee*: 70%; HPLC analysis CHIRALPAK IA, *n*-hexane:^{i}PrOH = 4:1, 25 °C, 1 mL/min flow rate, detection at 254 nm, t_1 = 9.3 min (major), t_2 = 19.4 min (minor).

_S_-(4-Methoxyphenyl) (_R_)-2-(5-bromo-3-((_tert_-butoxycarbonyl)amino)-1-methyl-2-oxoindolin-3-yl)ethanethioate (8t)

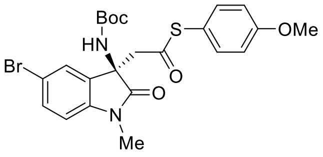

8t

White solid, 99% yield; Mp 187-188 ºC; ^{1}H NMR ($CDCl_3$, 400 MHz) δ (ppm) 748-7.45 (m, 1H), 7.42-7.35 (m, 1H), 7.30 (d, *J* = 8.9 Hz, 2H), 6.98 (d, *J* = 8.9 Hz, 2H), 6.76 (d, *J* = 8.3 Hz, 1H), 6.30 (s, 1H), 3.84 (s, 3H), 3.25 (s, 3H), 3.16 (d, *J* = 14.8 Hz, 1H), 2.73 (d, *J* = 15.0 Hz, 1H), 1.27 (s, 9H); ^{13}C NMR ($CDCl_3$, 100 MHz) δ (ppm) 196.7, 174.6, 161.3, 153.7, 142.4, 136.3, 132.3, 130.8, 126.9, 117.2, 115.5, 115.3, 110.1, 80.7, 60.1, 55.6, 47.3, 28.2, 27.0; IR (KBr) ν 3280, 2977, 2937, 1709, 1609, 1592, 1494, 1391, 1250, 1172, 1028, 832 cm^{-1}; HRMS (ESI^{+}) calc. for $[M+Na]^{+}$ ($C_{23}H_{25}BrN_2O_5SNa^{+}$), 543.0560, found 543.0554; $[\alpha]_D^{25}$ = +106.0 (*c* = 0.50, $CHCl_3$); *ee*: 75%; HPLC analysis CHIRALPAK OD-H, *n*-hexane:^{i}PrOH = 4:1, 25 °C, 1 mL/min flow rate, detection at 254 nm, t_1 = 10.5 min (major), t_2 = 12.6 min (minor).

_S_-(4-Methoxyphenyl) (_R_)-2-(6-bromo-3-((_tert_-butoxycarbonyl)amino)-1-methyl-2-oxoindolin-3-yl)ethanethioate (8u)

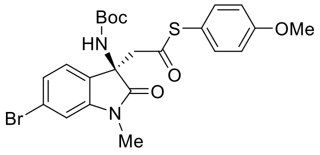

8u

White solid, 98% yield; Mp 186-187 ºC; ^{1}H NMR ($CDCl_3$, 500 MHz) δ (ppm) 7.26-7.20 (m, 3H), 7.14 (d, *J* = 7.9 Hz, 1H), 7.03-7.00 (m, 1H), 6.97-6.94 (m, 2H), 6.27 (s, 1H), 3.83 (s, 3H), 3.24 (s, 3H), 3.19 (d, *J* = 15.7 Hz, 1H), 2.77 (d, *J* = 15.3 Hz, 1H), 1.27 (s, 9H); ^{13}C NMR ($CDCl_3$, 125 MHz) δ (ppm) 196.5, 175.0, 161.3, 153.7, 144.7, 136.1, 127.9, 125.6, 125.0, 123.2, 117.2, 115.2, 112.2, 80.7, 59.9, 55.5, 47.4, 28.2, 27.0; IR (KBr) ν 3326,2976, 2931, 1728, 1608, 1592, 1895, 1368, 1250, 1173, 1050, 829 cm^{-1}; HRMS (ESI^{+}) calc. for $[M+Na]^{+}$ ($C_{23}H_{25}BrN_2O_5SNa^{+}$), 543.0560, found 543.0554; $[\alpha]_D^{25}$ = +105.6 (*c* = 0.50, $CHCl_3$); *ee*: 71%; HPLC analysis CHIRALPAK OD-H, *n*-hexane:^{i}PrOH = 4:1, 25 °C, 1 mL/min flow rate, detection at 254 nm, t_1 = 9.3 min (major), t_2 = 13.2 min (minor).

S-(4-Methoxyphenyl) (R)-2-(7-bromo-3-((tert-butoxycarbonyl)amino)-1-methyl-2-oxoindolin-3-yl)ethanethioate (8v)

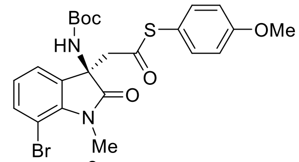
8v

White solid, 85% yield; Mp 156-157 ºC; ^{1}H NMR ($CDCl_3$, 400 MHz) δ (ppm) 7.44 (d, J = 8.1 Hz, 1H), 7.26-7.18 (m, 3H), 6.96-6.89 (m, 3H), 3.83 (s, 3H), 3.64 (s, 3H), 3.13 (d, J = 15.0 Hz, 1H), 2.76 (d, J = 15.0 Hz, 1H), 1.26 (s, 9H); ^{13}C NMR ($CDCl_3$, 100 MHz) δ (ppm) 196.8, 175.7, 161.3, 153.7, 140.6, 136.1, 135.1, 132.3, 123.9, 122.5, 117.3, 115.2, 103.0, 80.7, 59.9, 55.6, 47.5, 30.5, 28.2; IR (KBr) ν 3325, 2977, 2933, 1729, 1609, 1592, 1496, 1463, 1367, 1250, 1173, 1029, 828, 734 cm^{-1}; HRMS (ESI^+) calc. for $[M+Na]^+$ ($C_{23}H_{25}BrN_2O_5SNa^+$), 543.0560, found 543.0557; $[\alpha]_D^{25}$ = +84.8 (c = 0.50, $CHCl_3$); *ee*: 66%; HPLC analysis CHIRALPAK IA, *n*-hexane:^{i}PrOH = 4:1, 25 °C, 1 mL/min flow rate, detection at 254 nm, t_1 = 8.4 min (major), t_2 = 10.6 min (minor).

S-(4-Methoxyphenyl) (R)-2-(3-((tert-butoxycarbonyl)amino)-1-methyl-2-oxo-7-(trifluoromethyl)indolin-3-yl)ethanethioate (8w)

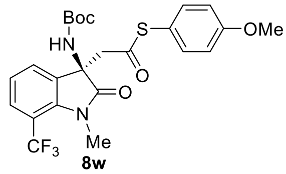
8w

White solid, 74% yield; Mp 151-152 ºC; ^{1}H NMR ($CDCl_3$, 500 MHz) δ (ppm) δ 7.63 (d, J = 8.0 Hz, 1H), 7.46 (d, J = 7.3 Hz, 1H), 7.22 (d, J = 8.7 Hz, 2H), 7.15 (t, J = 7.7 Hz, 1H), 6.95 (d, J = 8.9 Hz, 2H), 6.33 (s, 1H), 3.83 (s, 3H), 3.46 (d, J = 2.4 Hz, 3H), 3.15 (d, J = 15.1 Hz, 1H), 2.77 (d, J = 15.1 Hz, 1H), 1.23 (s, 9H); ^{13}C NMR ($CDCl_3$, 125 MHz) δ (ppm) 196.4, 176.1, 161.3, 153.5, 141.2, 136.1, 131.8, 127.4 (q, J = 6.3 Hz, *C*-C-CF_3), 126.9, 123.6 (q, J = 269.9 Hz, *C*F_3), 117.2, 115.2, 112.9 (q, J = 32.5 Hz, *C*-CF_3), 80.8, 58.8, 55.5, 47.5, 29.6, 28.1; IR (KBr) ν 3333, 2982, 2840, 1733, 1706, 1599, 1497, 1464, 1250, 1176, 1122, 828, 751 cm^{-1}; HRMS (ESI^+) calc. for $[M+Na]^+$ ($C_{24}H_{25}F_3N_2O_5SNa^+$), 533.1329, found 533.1324; $[\alpha]_D^{25}$ = +60.6 (c = 0.50, $CHCl_3$); *ee*: 60%; HPLC analysis CHIRALPAK IA, *n*-hexane:^{i}PrOH = 4:1, 25 °C, 1 mL/min flow rate, detection at 254 nm, t_1 = 7.5 min (major), t_2 = 16.2 min (minor).

***S*-(2-Methoxyphenyl) (*R*)-2-(3-((*tert*-butoxycarbonyl)amino)-1-methyl-2-oxoindolin-3-yl)ethanethioate (8ab)**

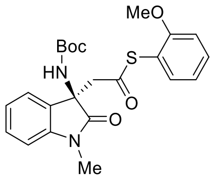

8ab

Yellow solid, 51% yield; Mp 186-187 ºC; ^{1}H NMR ($CDCl_3$, 500 MHz) δ (ppm) 7.47-7.43 (m, 1H), 7.35-7.30 (m, 3H), 7.07 (t, *J* = 7.5 Hz, 1H), 7.00 (t, *J* = 7.6 Hz, 2H), 6.87 (d, *J* = 7.8 Hz, 1H), 6.40 (s, 1H), 3.86 (s, 3H), 3.27 (s, 3H), 3.22 (d, *J* = 15.0 Hz, 1H), 2.76 (d, *J* = 15.1 Hz, 1H), 1.24 (s, 9H); ^{13}C NMR ($CDCl_3$, 125 MHz) δ (ppm) 195.0, 175.2, 159.3, 153.7, 143.3, 136.6, 132.4, 129.4, 129.0, 123.9, 122.7, 121.3, 115.1, 111.8, 108.5, 80.3, 60.2, 56.1, 47.7, 28.2, 26.8; IR (KBr) ν 3329, 2975, 2933, 1709, 1614, 1495, 1366, 1276, 1164, 1023, 753 cm^{-1}; HRMS (ESI^+) calc. for $[M+Na]^+$ ($C_{23}H_{26}N_2O_5SNa^+$), 465.1455, found 465.1454; $[\alpha]_D^{25}$ = +80.0 (*c* = 0.46, $CHCl_3$); *ee*: 63%; HPLC analysis CHIRALPAK IA, *n*-hexane:^{i}PrOH = 4:1, 25 °C, 1 mL/min flow rate, detection at 254 nm, t_1 = 10.3 min (major), t_2 = 18.4 min (minor).

***S*-Phenyl (*R*)-2-(3-((*tert*-butoxycarbonyl)amino)-1-methyl-2-oxoindolin-3-yl)ethanethioate (8ac)**[9]

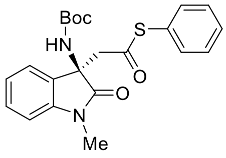

8ac

64% yield; ^{1}H NMR ($CDCl_3$, 300 MHz) δ (ppm) 7.46-7.39 (m, 3H), 7.37-7.29 (m, 4H), 7.08 (t, *J* = 7.3 Hz, 1H), 6.87 (d, *J* = 7.8 Hz, 1H), 3.27-3.20 (m, 4H), 2.81 (d, *J* = 15.0 Hz, 1H), 1.24 (s, 9H); ^{13}C NMR ($CDCl_3$, 100 MHz) δ (ppm) 195.5, 175.1, 153.8, 143.3, 134.5, 130.1, 129.5, 129.5, 129.0, 126.8, 123.7, 122.9, 108.6, 80.4, 60.3, 47.9, 28.2, 26.8; $[\alpha]_D^{25}$ = +93.4 (*c* = 0.50, $CHCl_3$); *ee*: 69%; HPLC analysis CHIRALPAK OD-H, *n*-hexane:^{i}PrOH = 4:1, 25 °C, 1 mL/min flow rate, detection at 254 nm, t_1 = 7.5 min (major), t_2 = 8.6 min (minor).

***S*-(*p*-Tolyl) (*R*)-2-(3-((*tert*-butoxycarbonyl)amino)-1-methyl-2-oxoindolin-3-yl)ethanethioate (8ad)**[9]

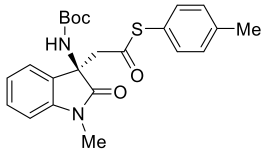

8ad

79% yield; ^{1}H NMR ($CDCl_3$, 500 MHz) δ (ppm) 7.37-7.31 (m, 1H), 7.31-7.19 (m, 5H), 7.07 (t, *J* = 7.6 Hz, 1H), 6.87 (d, *J* = 7.8 Hz, 1H), 6.31 (s, 1H), 3.26 (s, 3H), 3.21 (d, *J* = 15.1 Hz, 1H), 2.78 (d, *J* = 15.1 Hz, 1H), 2.38 (s, 3H), 1.24 (s, 9H); ^{13}C NMR ($CDCl_3$, 125 MHz) δ (ppm) 196.2, 175.1, 153.6, 143.3, 140.6, 134.5, 130.4, 129.5, 128.9, 123.7, 123.3, 122.8, 108.6, 80.4, 60.3, 47.8, 28.2, 26.8, 21.5; $[\alpha]_D^{25}$ = +99.4 (*c* = 0.50, $CHCl_3$); *ee*: 74%; HPLC analysis CHIRALPAK IA, *n*-hexane:^{i}PrOH = 4:1, 25 °C, 1 mL/min flow rate, detection at 254 nm, t_1 = 8.3 min (major), t_2 = 13.8 min (minor).

***S*-(4-Isopropylphenyl) (*R*)-2-(3-((*tert*-butoxycarbonyl)amino)-1-methyl-2-oxoindolin-3-yl)ethanethioate (8ae)**

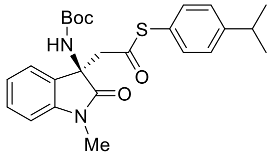

8ae

Yellow solid, 86% yield; Mp 126-127 ºC;^{1}H NMR ($CDCl_3$, 400 MHz) δ (ppm) 7.36-7.24 (m, 6H), 7.08 (t, *J* = 7.6 Hz, 1H), 6.87 (d, *J* = 7.8 Hz, 1H), 6.32 (s, 1H), 3.27 (s, 3H), 3.21 (d, *J* = 14.8 Hz, 1H), 2.97-2.90 (m, 1H), 2.78 (d, *J* = 15.0 Hz, 1H), 1.27-1.23 (m, 15H); ^{13}C NMR ($CDCl_3$, 125 MHz) δ 196.2, 175.2, 153.8, 151.3, 143.3, 134.5, 129.5, 128.9, 127.79, 123.7, 123.6, 122.8, 108.6, 80.4, 60.3, 47.8, 34.1, 28.2, 26.9, 23.9; IR (KBr) ν 3337, 2963, 2931, 1722, 1614, 1495, 1472, 1367, 1163, 826, 751 cm^{-1}; HRMS (ESI^{+}) calc. for $[M+Na]^{+}$ ($C_{25}H_{30}N_2O_4SNa^{+}$), 477.1819, found 477.1818; $[\alpha]_D^{25}$ = +101.8 (*c* = 0.50, $CHCl_3$); *ee*: 76%; HPLC analysis CHIRALPAK IA, *n*-hexane:^{i}PrOH = 4:1, 25 °C, 1 mL/min flow rate, detection at 190 nm, t_1 = 7.6 min (major), t_2 = 12.6 min (minor).

***S*-(4-Fluorophenyl) (*R*)-2-(3-((*tert*-butoxycarbonyl)amino)-1-methyl-2-oxoindolin-3-yl)ethanethioate (8af)**

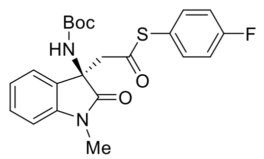

8af

Yellow solid, 76% yield; Mp 187-188 ºC; ^{1}H NMR (CDCl$_3$, 500 MHz) δ (ppm) 7.36-7.29 (m, 4H), 7.14-7.06 (m, 3H), 6.87 (d, *J* = 7.8 Hz, 1H), 6.18 (s, 1H), 3.27 (s, 3H), 3.23 (d, *J* = 14.3 Hz, 1H), 2.83 (d, *J* = 15.1 Hz, 1H), 1.25 (s, 9H); ^{13}C NMR (CDCl$_3$, 125 MHz) δ (ppm) 195.4, 175.1, 163.9 (d, *J* = 251.6 Hz, *C*-F), 153.8, 143.3, 136.6 (d, *J* = 8.6 Hz, *C*-C-C-F), 129.6, 128.9, 123.7, 122.9, 122.2 (d, *J* = 3.2 Hz, *C*-C-C-C-F), 116.9 (d, *J* = 22.3 Hz, *C*-C-F). 108.6, 80.5, 60.2, 47.9, 28.2, 26.8; IR (KBr) ν 3312, 2979, 2931, 1722, 1615, 1492, 1366, 1159, 833, 752 cm^{-1}; HRMS (ESI$^+$) calc. for [M+Na]$^+$ ($C_{22}H_{23}FN_2O_4SNa^+$), 453.1255, found 453.1255; $[\alpha]_D^{25}$ = +80.9 (*c* = 0.45, CHCl$_3$); *ee*: 77%; HPLC analysis CHIRALPAK OD-H, *n*-hexane:^{i}PrOH = 9:1, 25 °C, 1 mL/min flow rate, detection at 254 nm, t_1 = 7.1 min (major), t_2 = 8.3 min (minor).

***S*-(2-Fluorophenyl) (*R*)-2-(3-((*tert*-butoxycarbonyl)amino)-1-methyl-2-oxoindolin-3-yl)ethanethioate (8ag)**

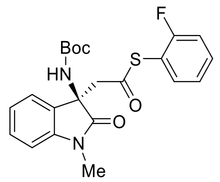

8ag

Yellow solid, 47% yield; Mp 186-187 ºC; ^{1}H NMR (CDCl$_3$, 500 MHz) δ (ppm) 7.50-7.45 (m, 1H), 7.36-7.32 (m, 3H), 7.22-7.18 (m, 2H), 7.08 (t, *J* = 7.5 Hz, 1H), 6.87 (d, *J* = 7.4 Hz, 1H), 6.23 (s, 1H), 3.27-3.25 (m, 4H), 2.83 (d, *J* = 15.1 Hz, 1H), 1.25 (s, 9H); ^{13}C NMR (CDCl$_3$, 125 MHz) δ (ppm) 193.6, 175.0, 162.1 (d, *J* = 250.7 Hz, *C*-F), 153.7, 143.2, 136.5, 132.9 (d, *J* = 8.2 Hz, *C*-C-C-F), 129.6, 128.7, 125.0 (d, *J* = 3.6 Hz, *C*-C-C-F), 123.8, 122.9, 116.5 (d, *J* = 22.3 Hz, *C*-C-F), 114.2 (d, *J* = 18.6 Hz), 108.6, 80.5, 60.2, 47.9, 28.2, 26.8; IR (KBr) ν 3326, 2974, 2910, 1724, 1702, 1615, 1478, 1358, 1125, 754 cm^{-1}; HRMS (ESI$^+$) calc. for [M+Na]$^+$ ($C_{22}H_{23}FN_2O_4SNa^+$), 453.1255, found 453.1254; $[\alpha]_D^{25}$ = +11.2 (*c* = 0.50, CHCl$_3$); *ee*: 12%; HPLC analysis CHIRALPAK OD-H, *n*-hexane:^{i}PrOH = 4:1, 25 °C, 1 mL/min flow rate, detection at 254 nm, t_1 = 7.9 min (major), t_2 = 8.9 min (minor).

S-(4-Chlorophenyl) (R)-2-(3-((tert-butoxycarbonyl)amino)-1-methyl-2-oxoindolin-3-yl)ethanethioate (8ah)[9]

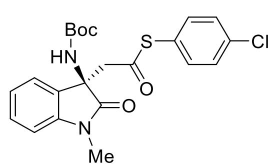

8ah

80% yield; ^{1}H NMR (CDCl$_3$, 500 MHz) δ (ppm) 7.41-7.38 (m, 2H), 7.36-7.30 (m, 2H), 7.27-7.25 (m, 2H), 7.07 (t, *J* = 7.4 Hz, 1H), 6.87 (d, *J* = 7.6 Hz, 1H), 6.15 (s, 1H), 3.26 (s, 3H), 3.24 (d, *J* = 10.5 Hz, 1H), 2.85 (d, *J* = 15.1 Hz, 1H), 1.25 (s, 9H); ^{13}C NMR (CDCl$_3$, 125 MHz) δ (ppm) 194.8, 175.0, 153.8, 143.3, 136.6, 135.7, 129.8, 129.6, 128.8, 125.2, 123.7, 122.9, 108.6, 80.5, 60.2, 48.0, 28.2, 26.8; $[\alpha]_D^{25}$ = +93.0 (*c* = 0.46, CHCl$_3$); *ee*: 80%; HPLC analysis CHIRALPAK OD-H, *n*-hexane:^{i}PrOH = 4:1, 25 °C, 1 mL/min flow rate, detection at 254 nm, t_1 = 7.4 min (major), t_2 = 8.8 min (minor).

S-(3-Chlorophenyl) (R)-2-(3-((tert-butoxycarbonyl)amino)-1-methyl-2-oxoindolin-3-yl)ethanethioate (8ai)

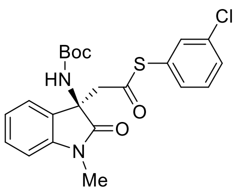

8ai

White solid, 49% yield; Mp 170-171 °C;^{1}H NMR (CDCl$_3$, 400 MHz) δ (ppm) 7.42-7.31 (m, 5H), 7.23 (d, *J* = 7.6 Hz, 1H), 7.09 (t, *J* = 7.5 Hz, 1H), 6.87 (d, *J* = 7.8 Hz, 1H), 6.15 (s, 1H), 3.27-3.24 (m, 4H), 2.85 (d, *J* = 15.2 Hz, 1H), 1.25 (s, 9H); ^{13}C NMR (CDCl$_3$, 100 MHz) δ (ppm) 194.4, 175.0, 153.8, 143.3, 135.1, 134.1, 132.6, 130.5, 130.3, 129.6, 128.8, 128.5, 123.8, 122.9, 108.6, 80.6, 77.5, 77.2, 76.8, 60.2, 48.1, 28.2, 26.8; IR (KBr) ν 3291, 2981, 2928, 1706, 1612, 1532, 1464, 1166, 763 cm^{-1}; HRMS (ESI$^+$) calc. for [M+Na]$^+$ ($C_{22}H_{23}ClN_2O_4SNa^+$), 469.0959, found 469.0957; $[\alpha]_D^{25}$ = +76.8 (*c* = 0.50, CHCl$_3$); *ee*: 79%; HPLC analysis CHIRALPAK IA, *n*-hexane:^{i}PrOH = 4:1, 25 °C, 1 mL/min flow rate, detection at 254 nm, t_1 = 9.1 min (major), t_2 = 20.4 min (minor).

***S*-(4-Bromophenyl) (*R*)-2-(3-((*tert*-butoxycarbonyl)amino)-1-methyl-2-oxoindolin-3-yl)ethanethioate (8aj)**

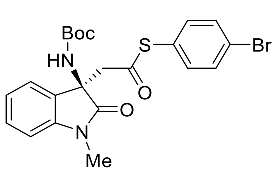

8aj

Yellow solid, 84% yield; Mp 179-180 °C; ^{1}H NMR ($CDCl_3$, 400 MHz) δ (ppm) 7.55 (d, *J* = 8.5 Hz, 2H), 7.36-7.26 (m, 2H), 7.19 (d, *J* = 8.5 Hz, 2H), 7.07 (t, *J* = 7.5 Hz, 1H), 6.87 (d, *J* = 7.7 Hz, 1H), 6.15 (s, 1H), 3.26 (s, 4H), 2.85 (d, *J* = 15.2 Hz, 1H), 1.25 (s, 9H); ^{13}C NMR ($CDCl_3$, 100 MHz) δ (ppm) 194.6, 175.0, 153.8, 143.3, 135.9, 132.7, 129.6, 128.8, 125.9, 124.9, 123.7, 122.9, 108.6, 80.5, 60.2, 48.1, 28.2, 26.8; IR (KBr) ν 3319, 2977, 2930, 1722, 1614, 1495, 1367, 1252, 1165, 814, 751 cm^{-1}; HRMS (ESI^{+}) calc. for $[M+Na]^{+}$ ($C_{22}H_{23}BrN_2O_4SNa^{+}$), 513.0454, found 513.0455; $[\alpha]_D^{25}$ = +86.8 (*c* = 0.50, $CHCl_3$); *ee*: 77%; HPLC analysis CHIRALPAK IA, *n*-hexane:^{i}PrOH = 4:1, 25 °C, 1 mL/min flow rate, detection at 254 nm, t_1 = 10.4 min (major), t_2 = 18.0 min (minor).

***S*-(3-Bromophenyl) (*R*)-2-(3-((*tert*-butoxycarbonyl)amino)-1-methyl-2-oxoindolin-3-yl)ethanethioate (8ak)**

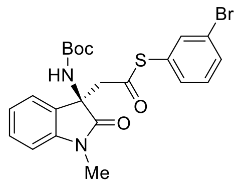

8ak

White solid, 73% yield; Mp 171-172 °C; ^{1}H NMR ($CDCl_3$, 400 MHz) δ (ppm) 7.58-7.47 (m, 1H), 7.47 (s, 1H), 7.37-7.26 (m, 4H), 7.09 (t, *J* = 7.5 Hz, 1H), 6.88 (d, *J* = 7.8 Hz, 1H), 6.14 (s, 1H), 3.27-3.24 (m, 4H), 2.85 (d, *J* = 15.2 Hz, 1H), 1.25 (s, 9H); ^{13}C NMR ($CDCl_3$, 100 MHz) δ (ppm) 194.4, 175.0, 153.8, 143.3, 136.9, 133.2, 133.1, 130.8, 129.6, 128.8, 123.8, 123.0, 122.9, 108.7, 80.6, 60.2, 48.1, 28.2, 26.8; IR (KBr) ν 3294, 2979, 2927, 1707, 1611, 1532, 1332, 1166, 762 cm^{-1}; HRMS (ESI^{+}) calc. for $[M+Na]^{+}$ ($C_{22}H_{23}BrN_2O_4SNa^{+}$), 513.0454, found 513.0454; $[\alpha]_D^{25}$ = +82.0 (*c* = 0.50, $CHCl_3$); *ee*: 78%; HPLC analysis CHIRALPAK IA, *n*-hexane:^{i}PrOH = 4:1, 25 °C, 1 mL/min flow rate, detection at 254 nm, t_1 = 9.4 min (major), t_2 = 22.8 min (minor).

S-(Naphthalen-2-yl) (R)-2-(3-((tert-butoxycarbonyl)amino)-1-methyl-2-oxoindolin-3-yl)ethanethioate (8al)[9]

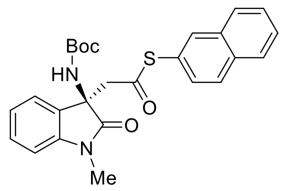

8al

65% yield; ^{1}H NMR ($CDCl_3$, 500 MHz) δ (ppm) δ 7.89-7.82 (m, 4H), 7.57-7.51 (m, 2H), 7.38-7.34 (m, 3H), 7.11 (t, J = 2.0 Hz, 1H), 6.88 (d, J = 7.8 Hz, 1H), 6.27 (s, 1H), 3.23-3.21 (m, 4H), 2.87 (d, J = 15.1 Hz, 1H), 1.25 (s, 9H); ^{13}C NMR ($CDCl_3$, 125 MHz) δ (ppm) δ 195.7, 175.1, 153.8, 143.3, 134.6, 133.7, 133.6, 130.6, 129.5, 129.2, 128.9, 128.2, 128.0, 127.6, 126.9, 124.1, 123.8, 122.9, 108.6, 80.5, 60.3, 48.0, 28.2, 26.8; $[\alpha]_D^{25}$ = +107.4 (c = 0.50, $CHCl_3$); *ee*: 68%; HPLC analysis CHIRALPAK OD-H, *n*-hexane:^{i}PrOH = 4:1, 25 °C, 1 mL/min flow rate, detection at 254 nm, t_1 = 9.6 min (major), t_2 = 13.7 min (minor).

S-Benzyl (R)-2-(3-((tert-butoxycarbonyl)amino)-1-methyl-2-oxoindolin-3-yl)ethanethioate (8am)[9]

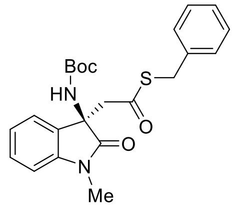

8am

48% yield; ^{1}H NMR ($CDCl_3$, 500 MHz) δ (ppm) 7.31-7.20 (m, 6H), 7.11 (d, J = 8.3 Hz, 1H), 6.94 (t, J = 7.6 Hz, 1H), 6.82 (d, J = 7.8 Hz, 1H), 6.27 (s, 1H), 4.14 (d, J = 14.2 Hz, 1H), 4.07 (d, J = 13.5 Hz, 1H), 3.23 (s, 3H), 3.12 (s, 1H), 2.70 (d, J = 14.9 Hz, 1H), 1.25 (s, 9H); ^{13}C NMR ($CDCl_3$, 125 MHz) δ (ppm) 195.9, 175.2, 153.7, 143.2, 136.8, 129.4, 129.0, 128.8, 127.6, 123.7, 122.8, 108.4, 80.5, 60.2, 48.3, 33.9, 28.2, 26.8; $[\alpha]_D^{25}$ = -7.6 (c = 0.50, $CHCl_3$); *ee*: 9%; HPLC analysis CHIRALPAK IA, *n*-hexane:^{i}PrOH = 4:1, 25 °C, 1 mL/min flow rate, detection at 254 nm, t_1 = 9.5 min (major), t_2 = 15.7 min (minor).

***S*-Ethyl (*R*)-2-(3-((*tert*-butoxycarbonyl)amino)-1-methyl-2-oxoindolin-3-yl)ethanethioate (8an)**

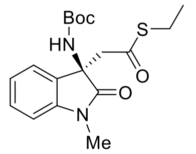

8an

Yellow solid, 34% yield; Mp 90-91 ºC; ^{1}H NMR ($CDCl_3$, 500 MHz) δ (ppm) 7.33-7.23 (m, 2H), 7.03 (t, *J* = 7.5 Hz, 1H), 6.85 (d, *J* = 7.6 Hz, 1H), 6.33 (s, 1H), 3.25 (s, 3H), 3.11 (d, *J* = 14.4 Hz, 1H), 2.92-2.84 (m, 2H), 2.68 (d, *J* = 14.9 Hz, 1H), 1.34-1.21 (m, 12H); ^{13}C ($CDCl_3$, 125 MHz) δ (ppm) 196.8, 175.3, 153.8, 143.2, 129.4, 129.0, 123.6, 122.8, 108.5, 80.5, 48.4, 28.3, 28.2, 26.8, 24.1, 14.5; IR (KBr) ν 3268, 2974, 2932, 1706, 1613, 1537, 1493, 1363, 1173, 759 cm^{-1}; HRMS (ESI^+) calc. for $[M+Na]^+$ ($C_{18}H_{24}N_2O_4SNa^+$), 387.1349, found 387.1348; $[\alpha]_D^{25}$ = +2.9 (*c* = 0.35, $CHCl_3$); *ee*: 9%; HPLC analysis CHIRALPAK OD-H, *n*-hexane:^{i}PrOH = 4:1, 25 °C, 1 mL/min flow rate, detection at 254 nm, t_1 = 5.6 min (major), t_2 = 6.9 min (minor).

4. Copies of ^{1}H and ^{13}C NMR spectra

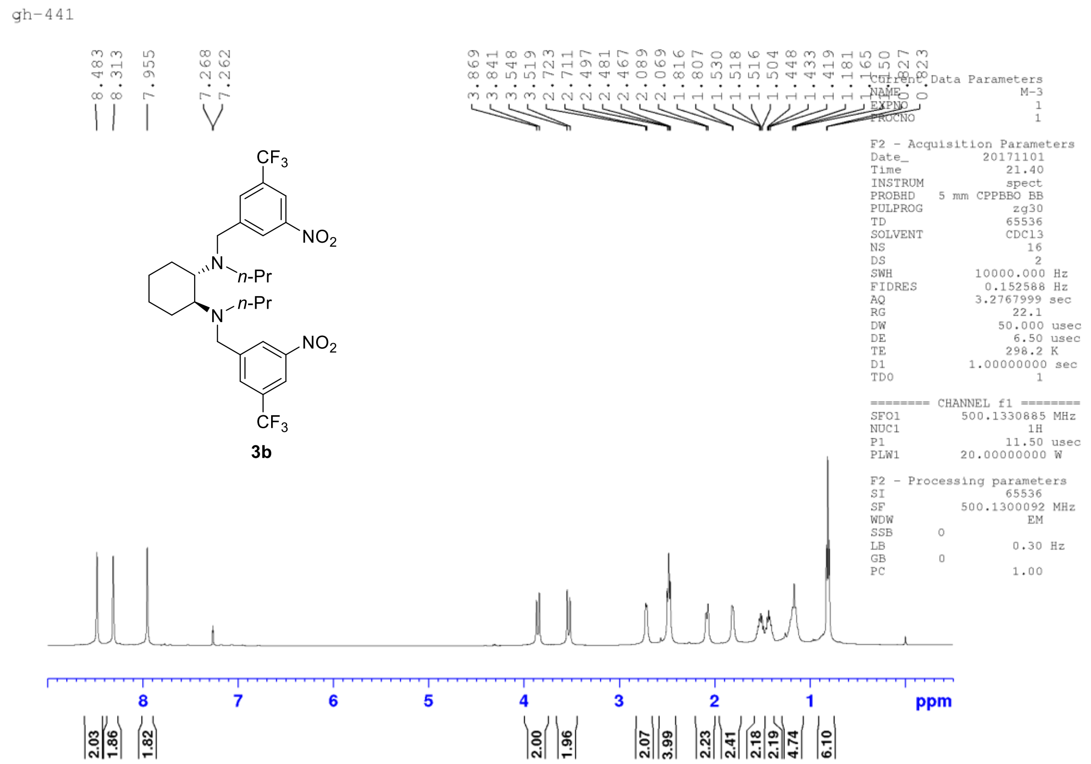

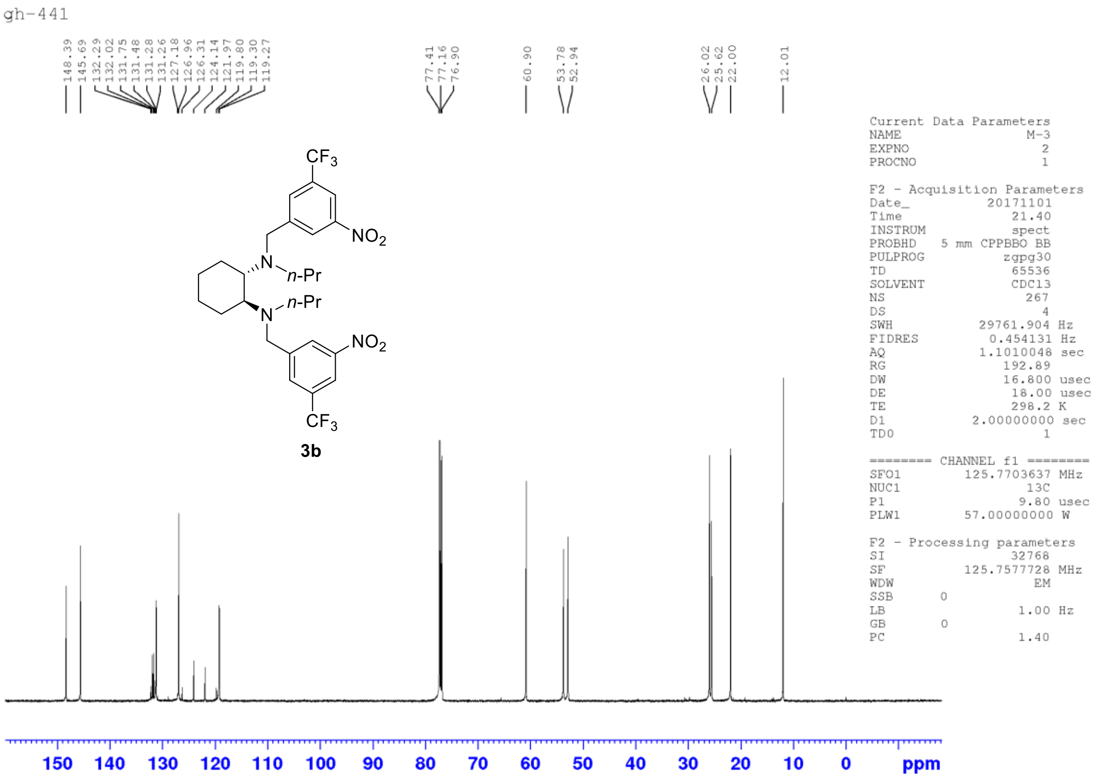

^{1}H and ^{13}C NMR of **3b** in $CDCl_3$.

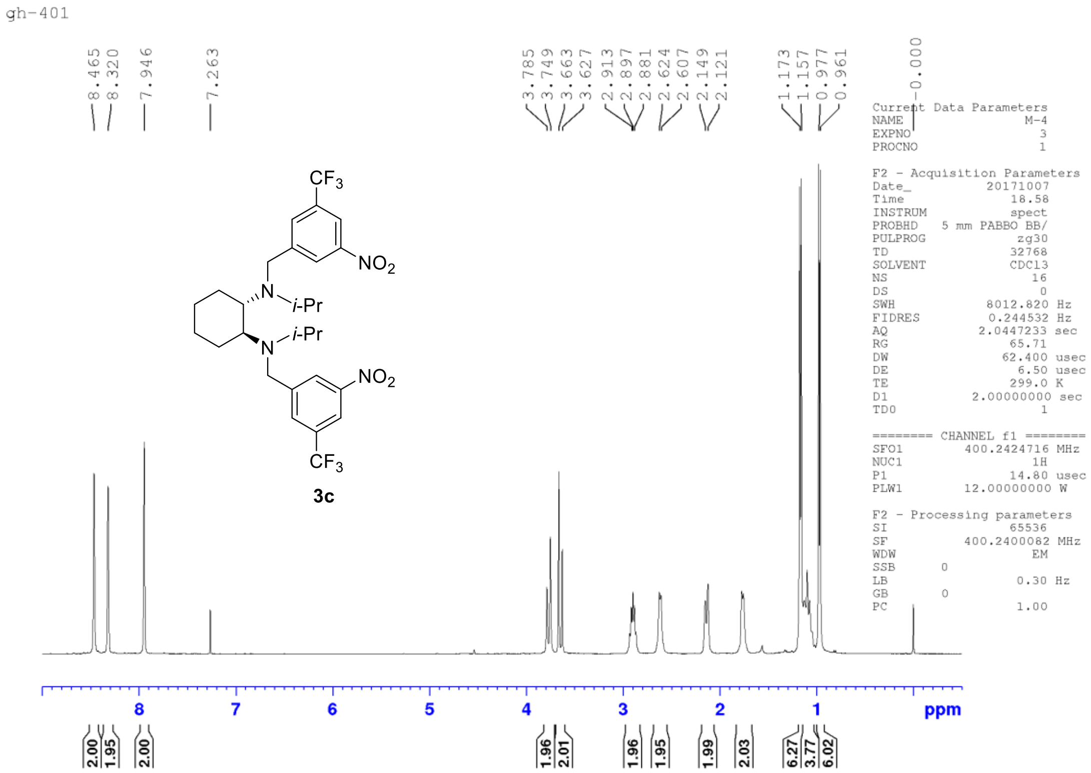

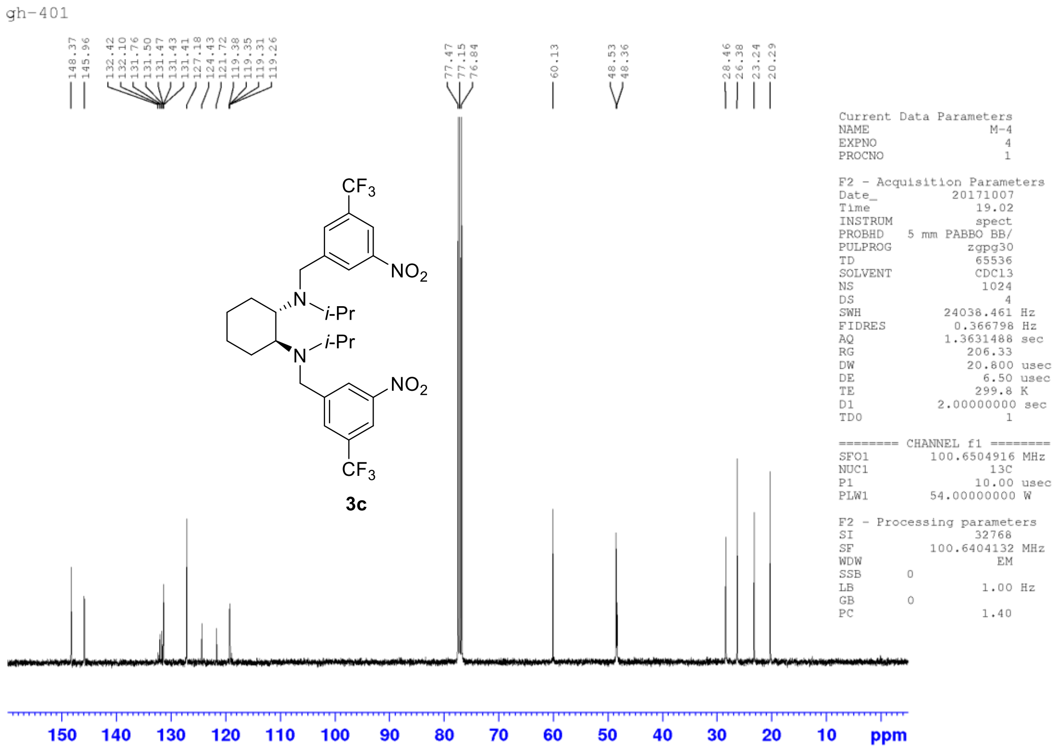

^{1}H and ^{13}C NMR of **3c** in $CDCl_3$.

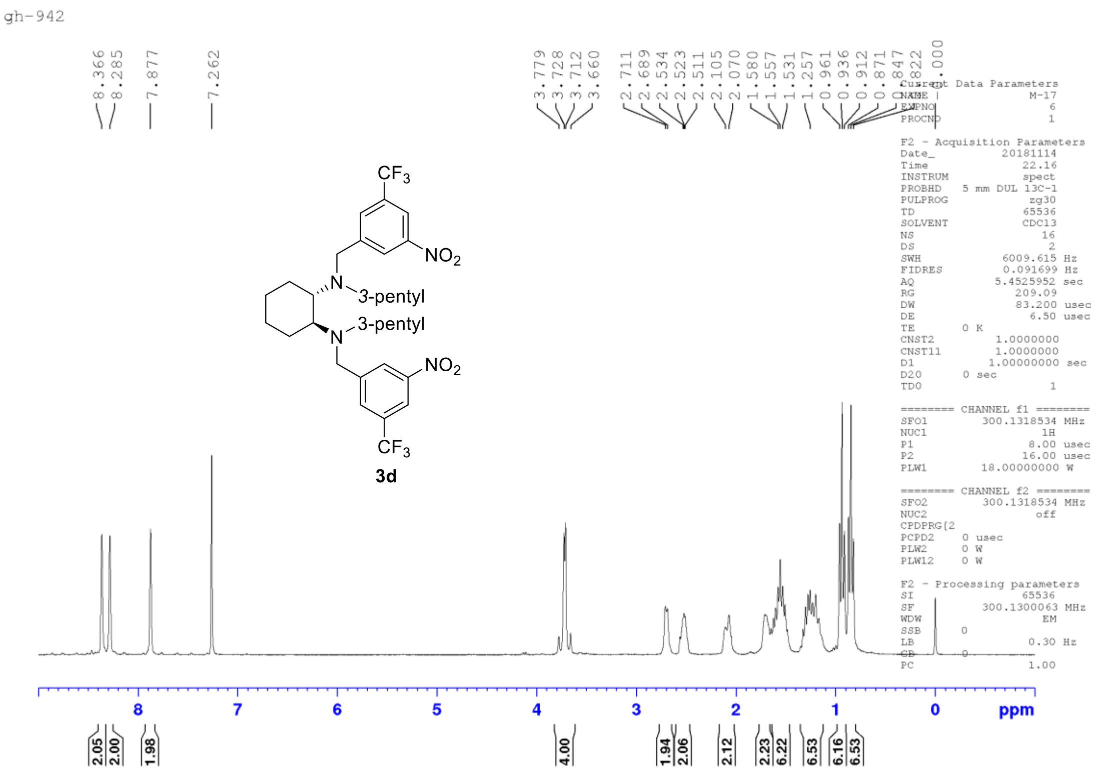

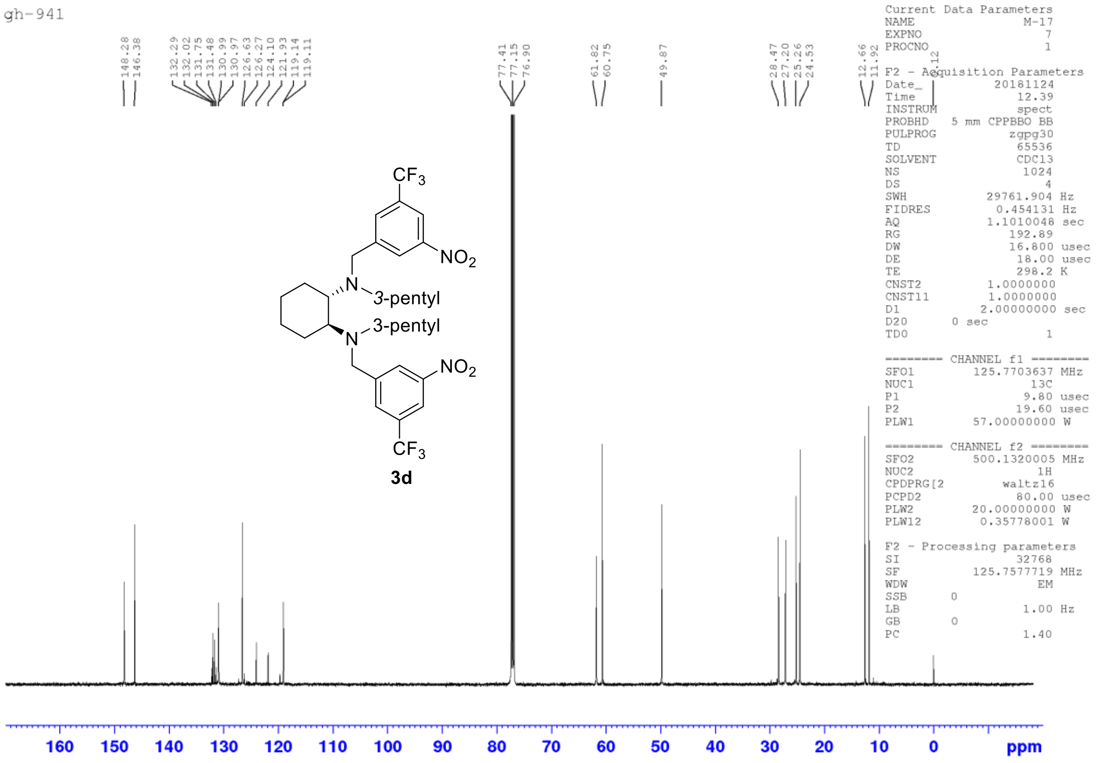

^{1}H and ^{13}C NMR of **3d** in $CDCl_3$.

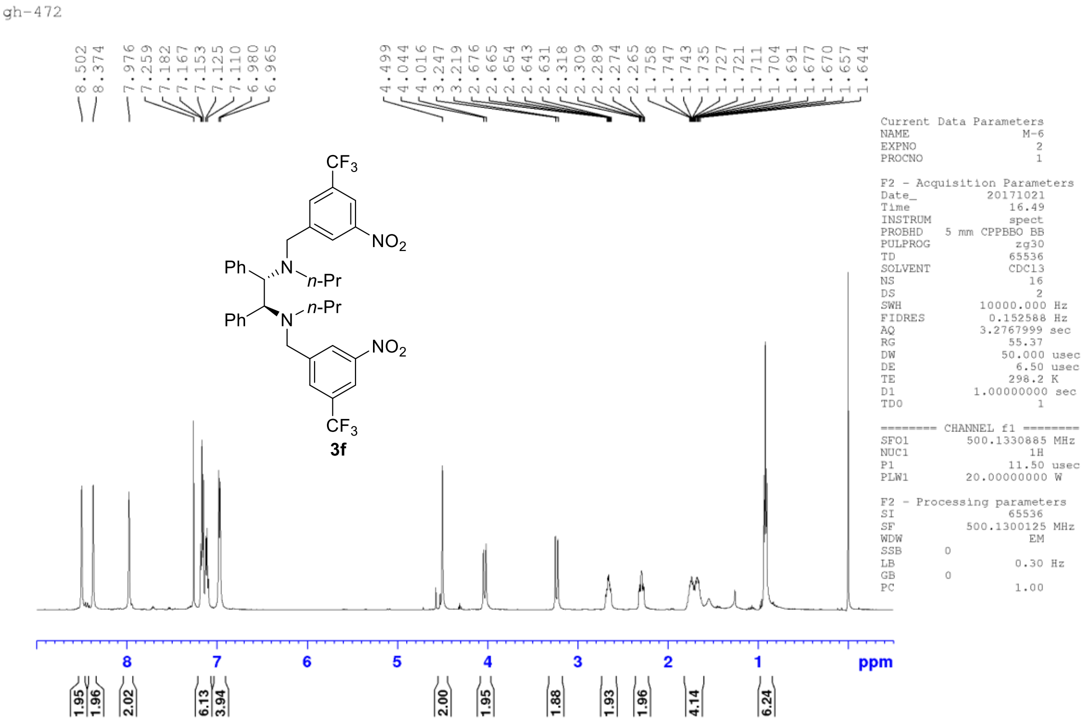

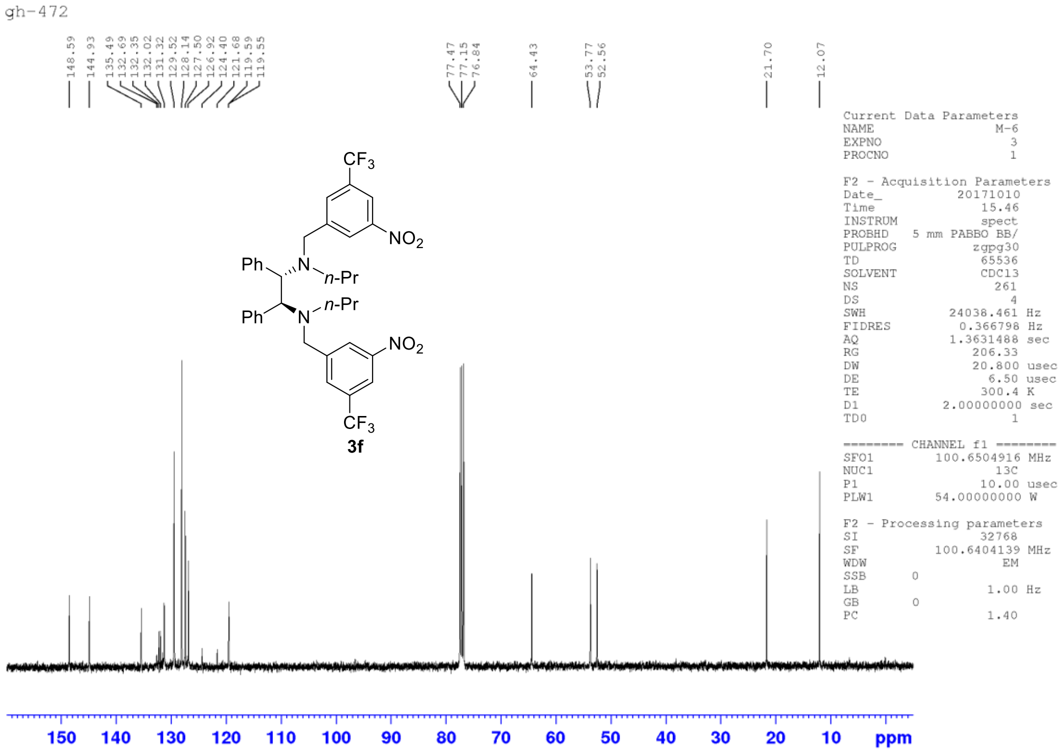

^{1}H and ^{13}C NMR of **3f** in $CDCl_3$.

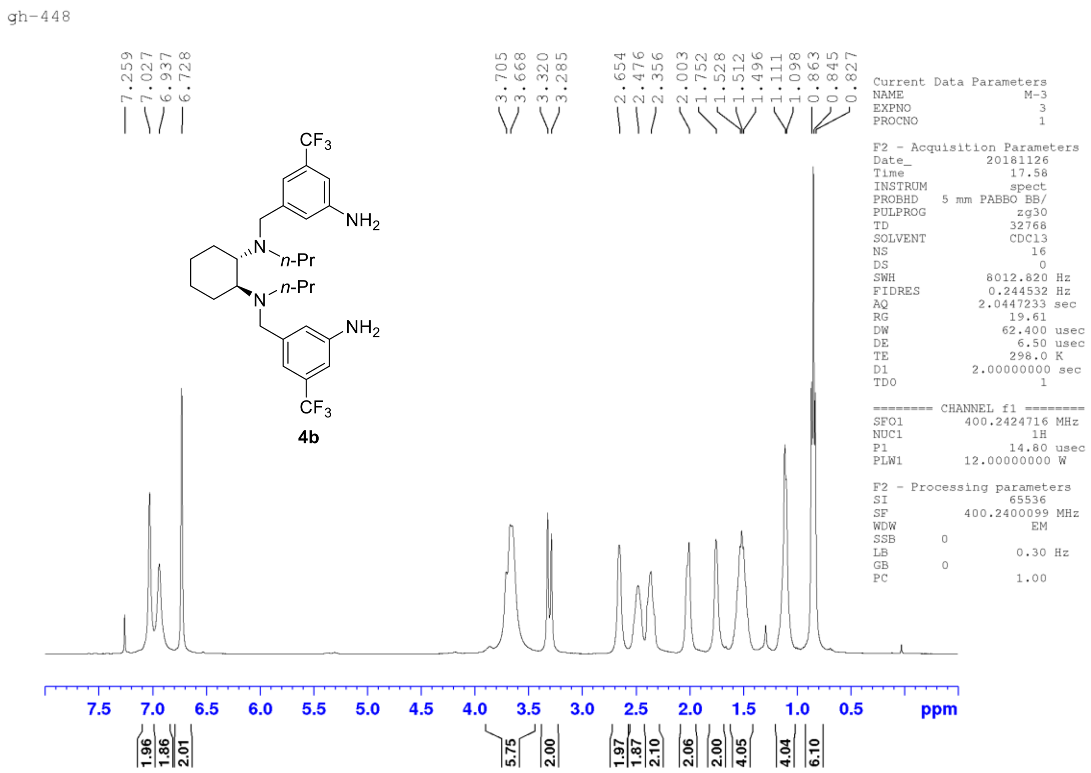

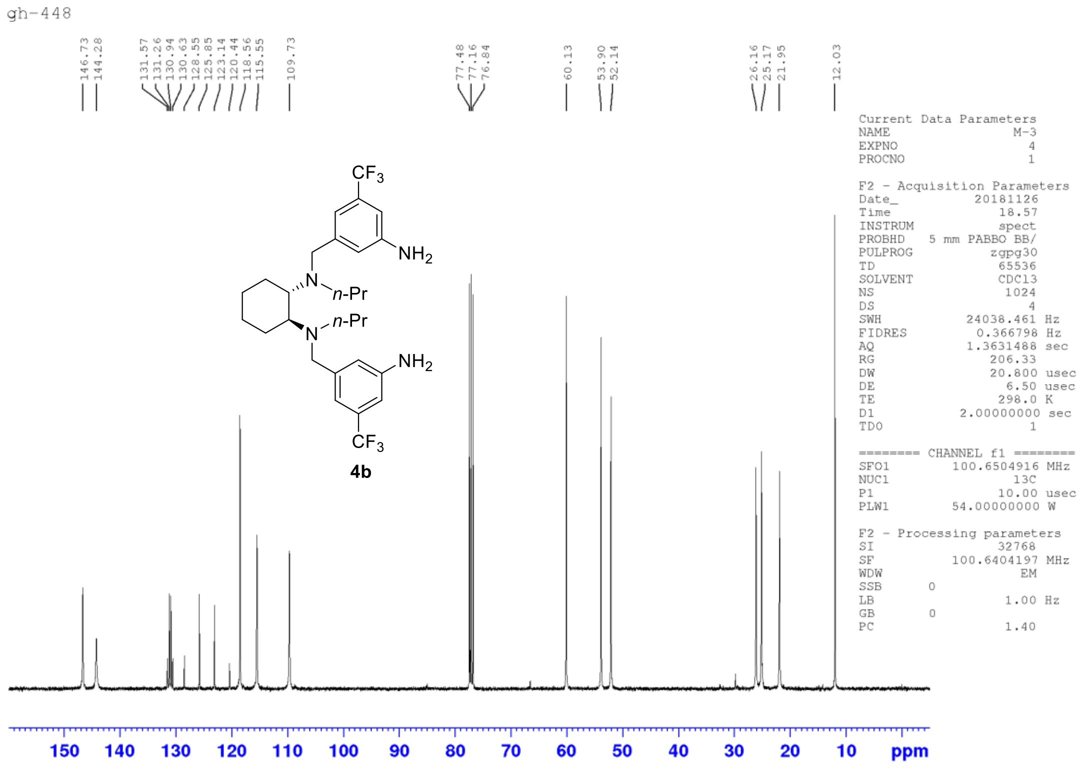

^{1}H and ^{13}C NMR of **4b** in $CDCl_3$.

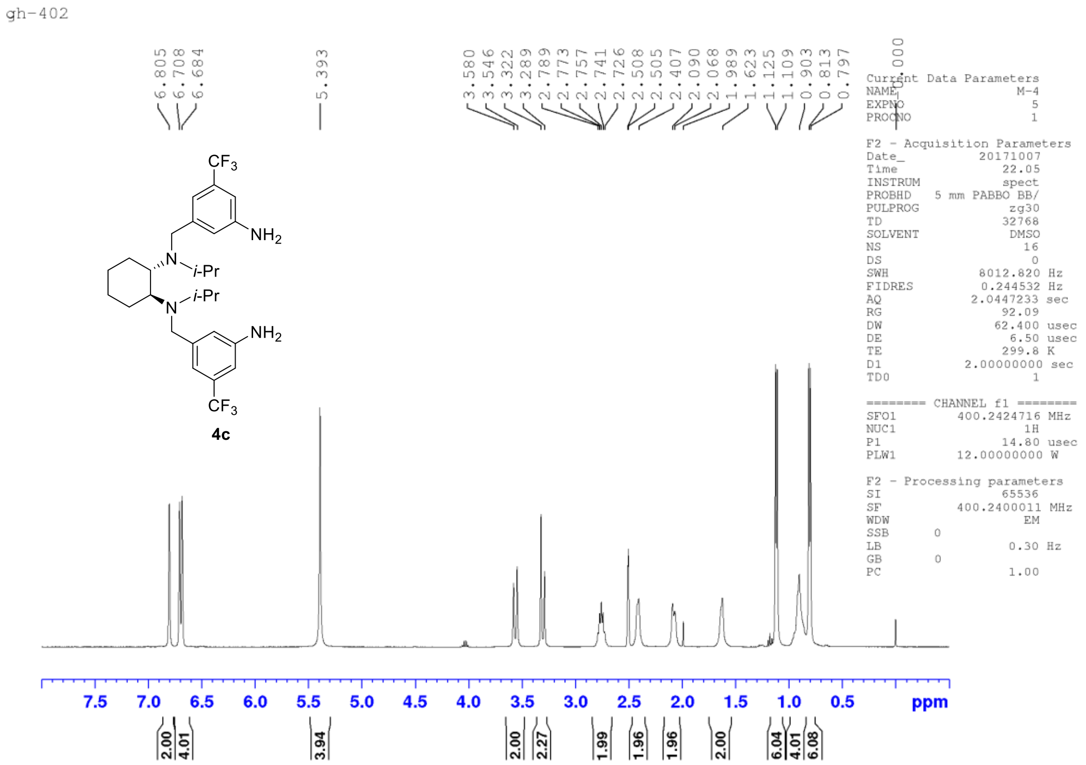

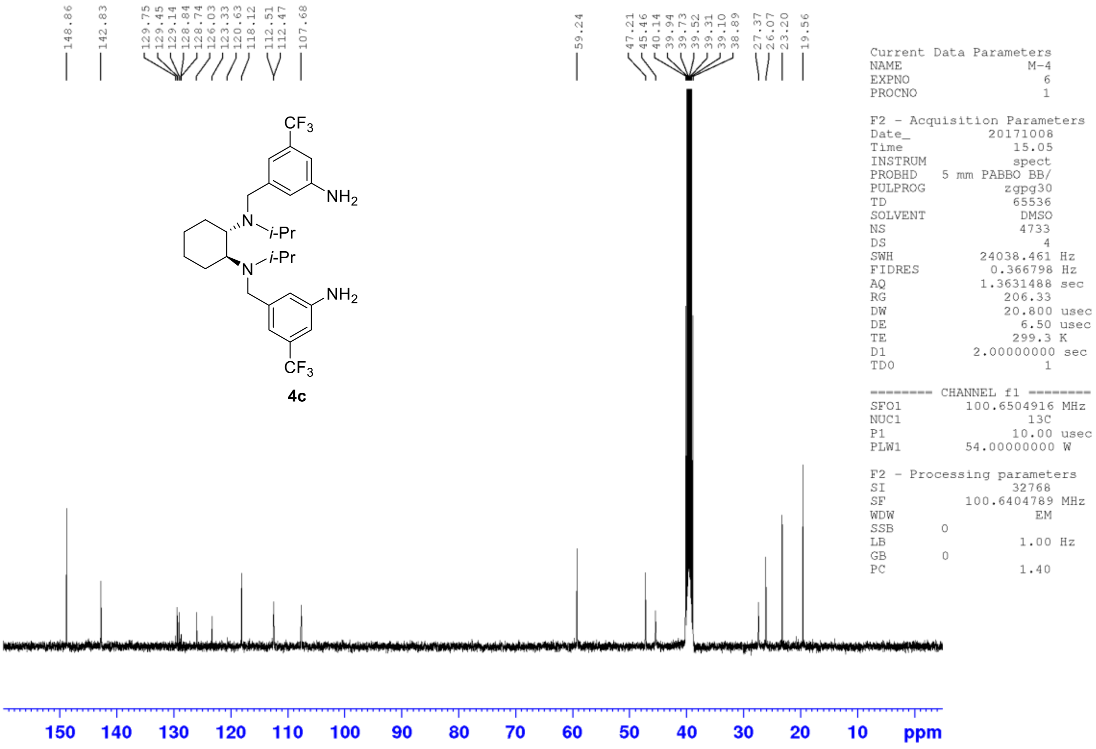

^{1}H and ^{13}C NMR of **4c** in DMSO-d_6.

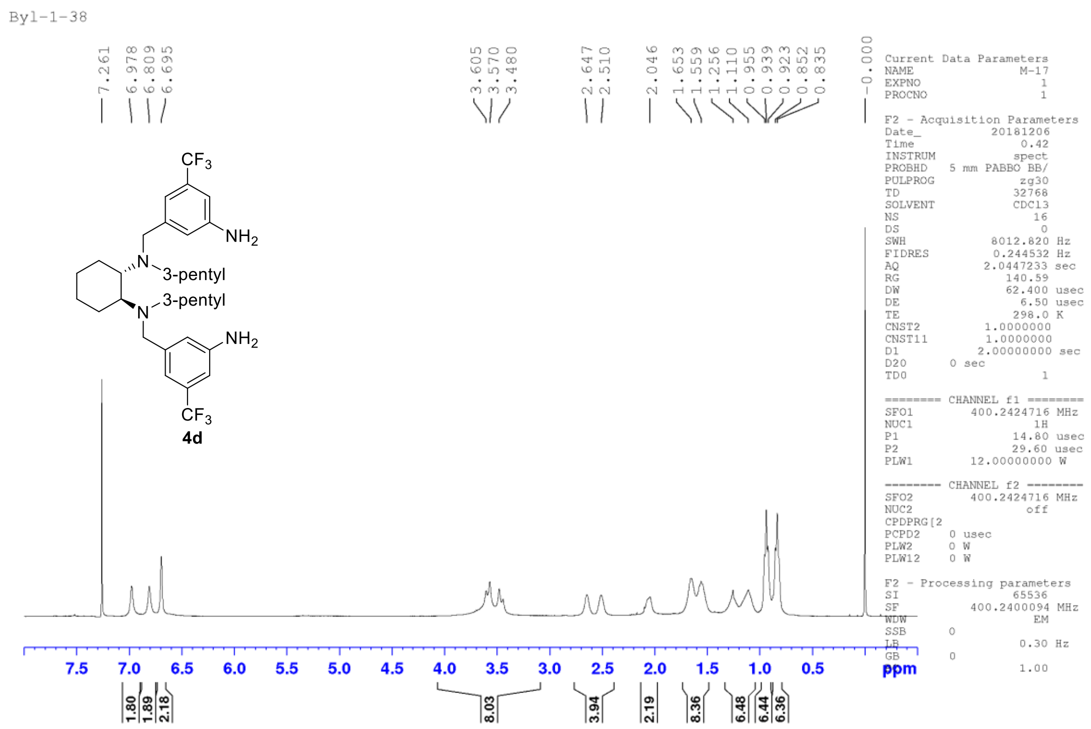

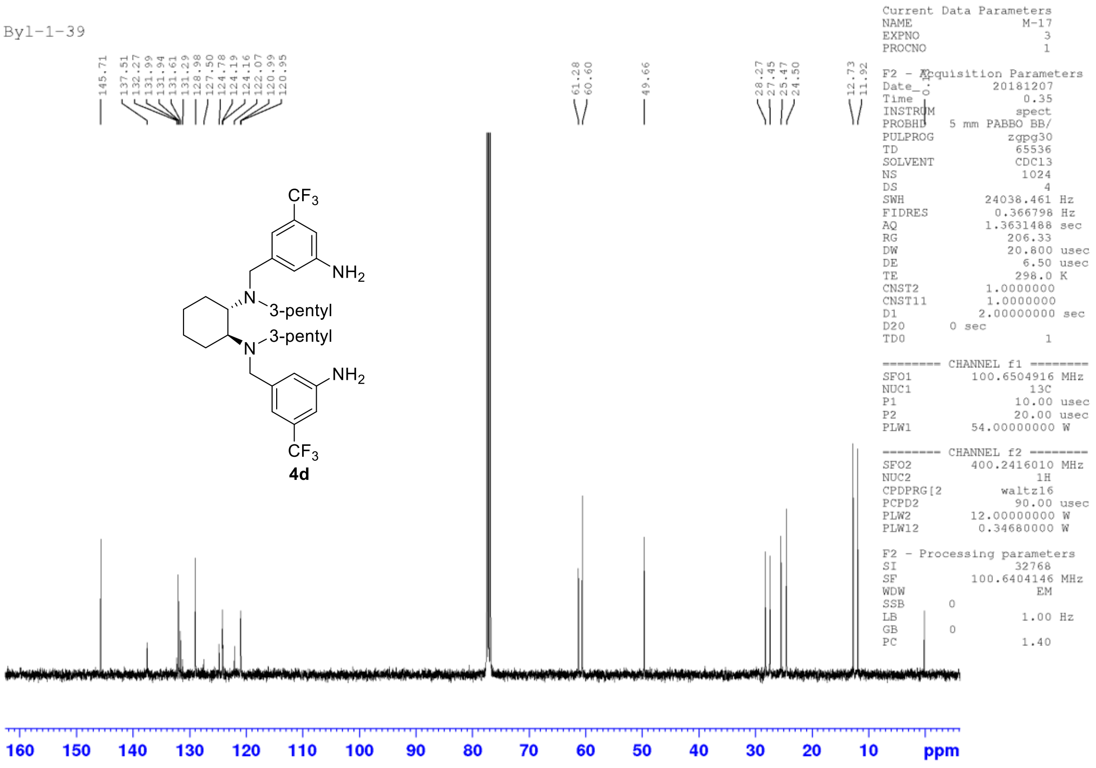

^{1}H and ^{13}C NMR of **4d** in $CDCl_3$.

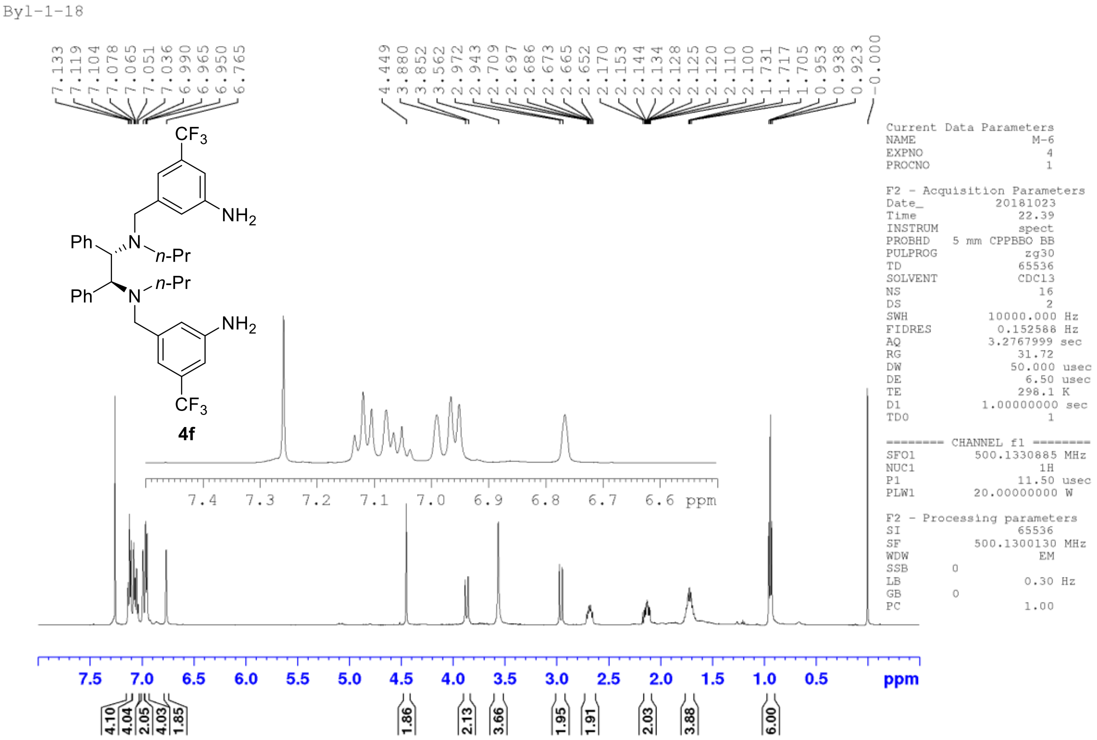

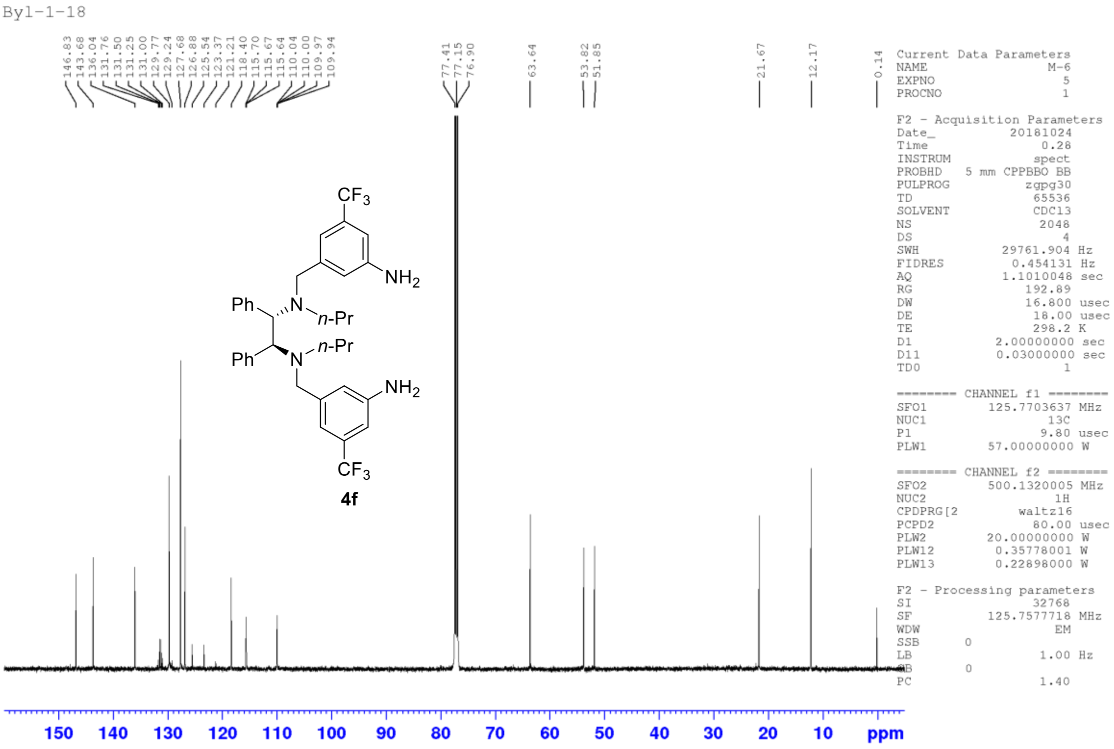

^{1}H and ^{13}C NMR of **4f** in $CDCl_3$.

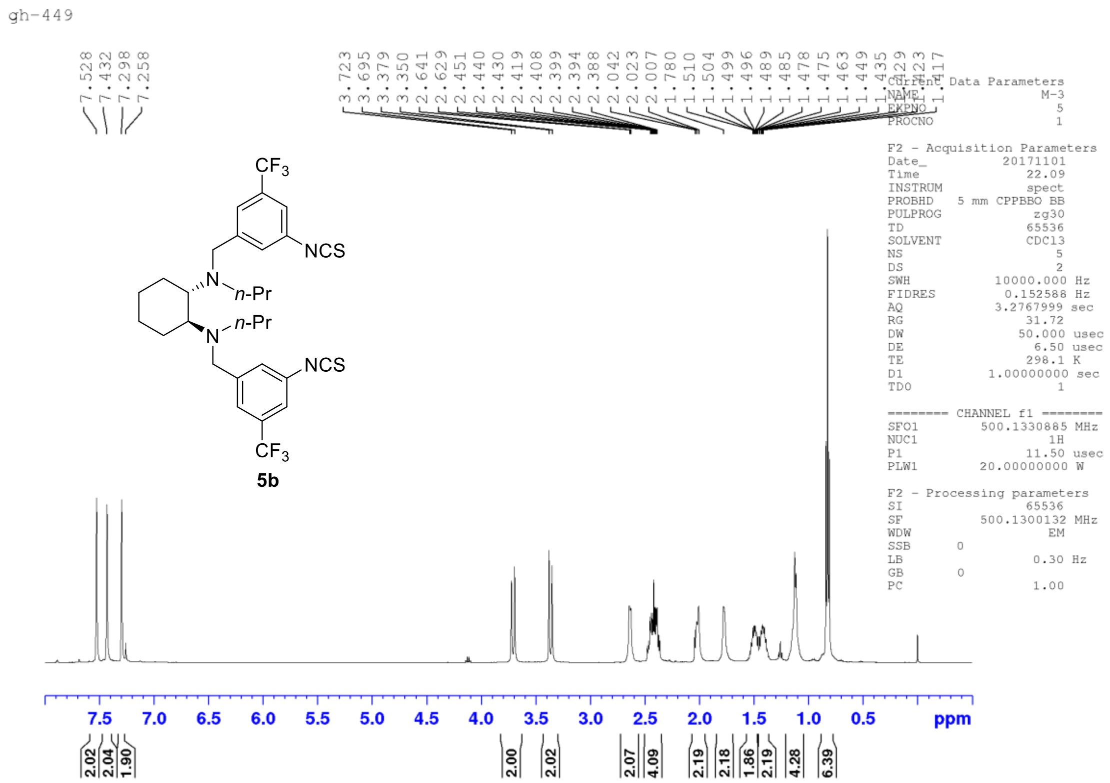

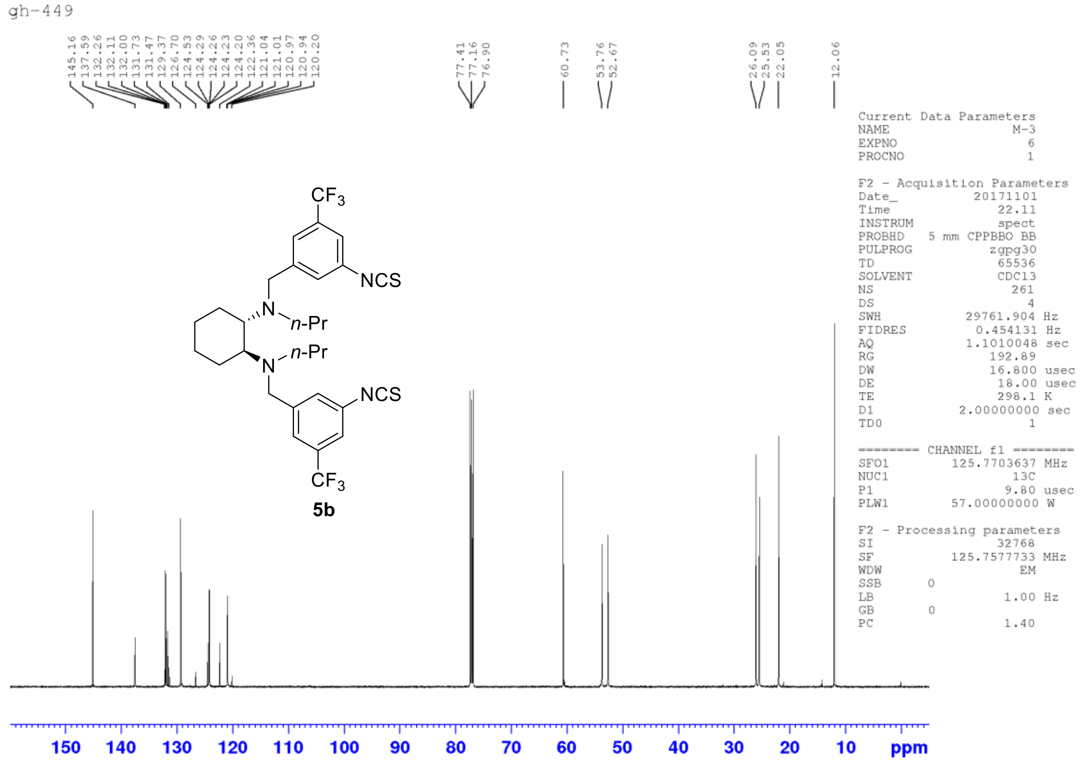

^{1}H and ^{13}C NMR of **5b** in $CDCl_3$.

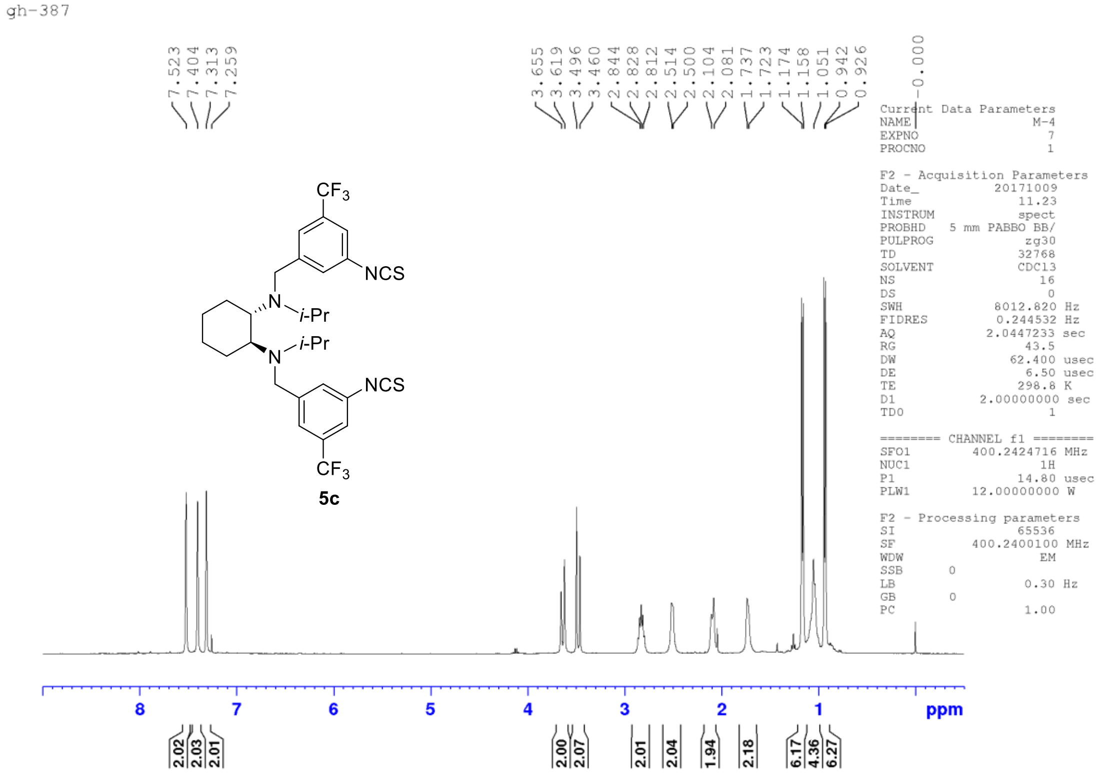

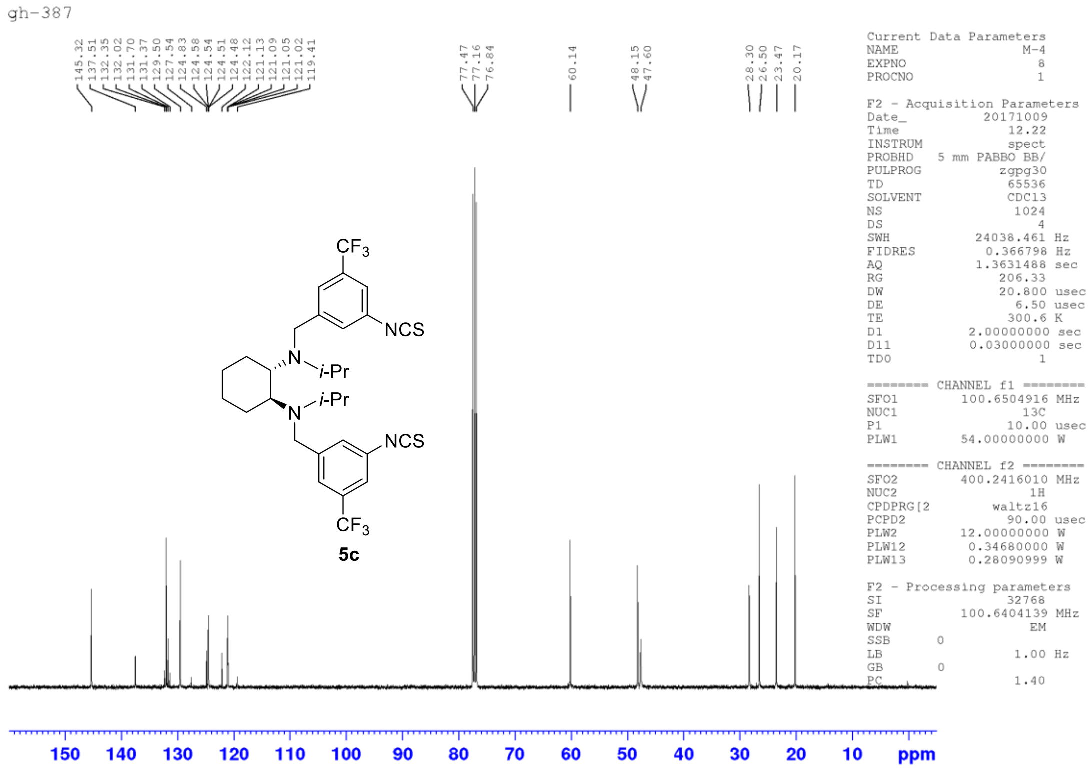

^{1}H and ^{13}C NMR of **5c** in $CDCl_3$.

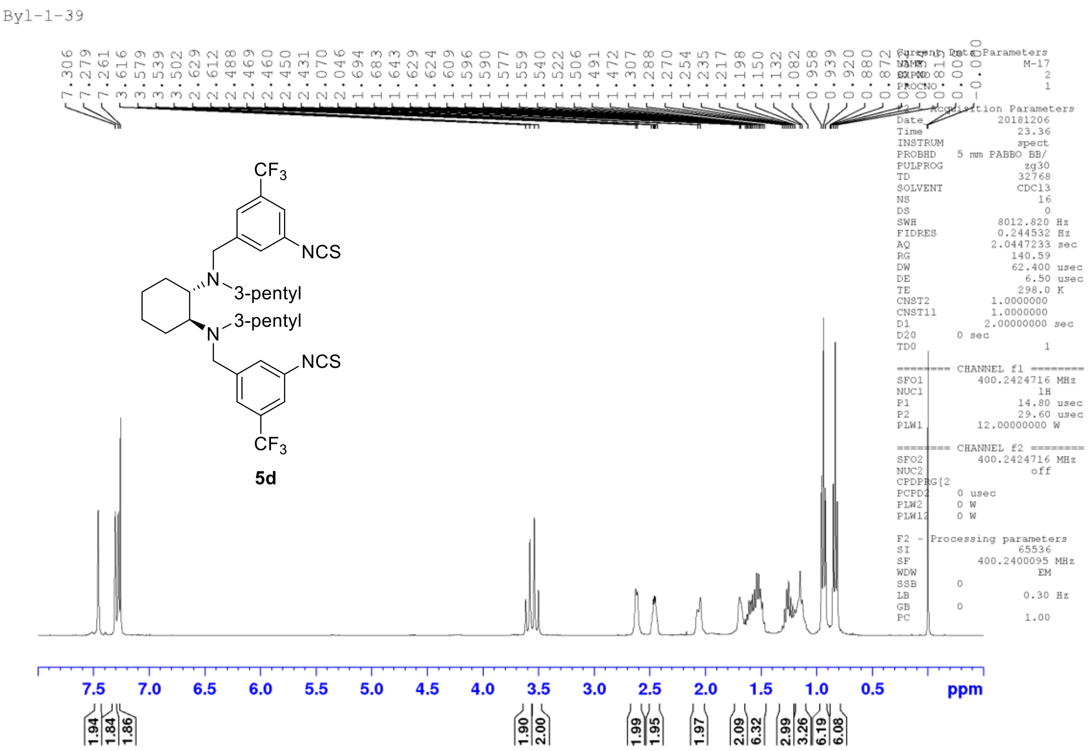

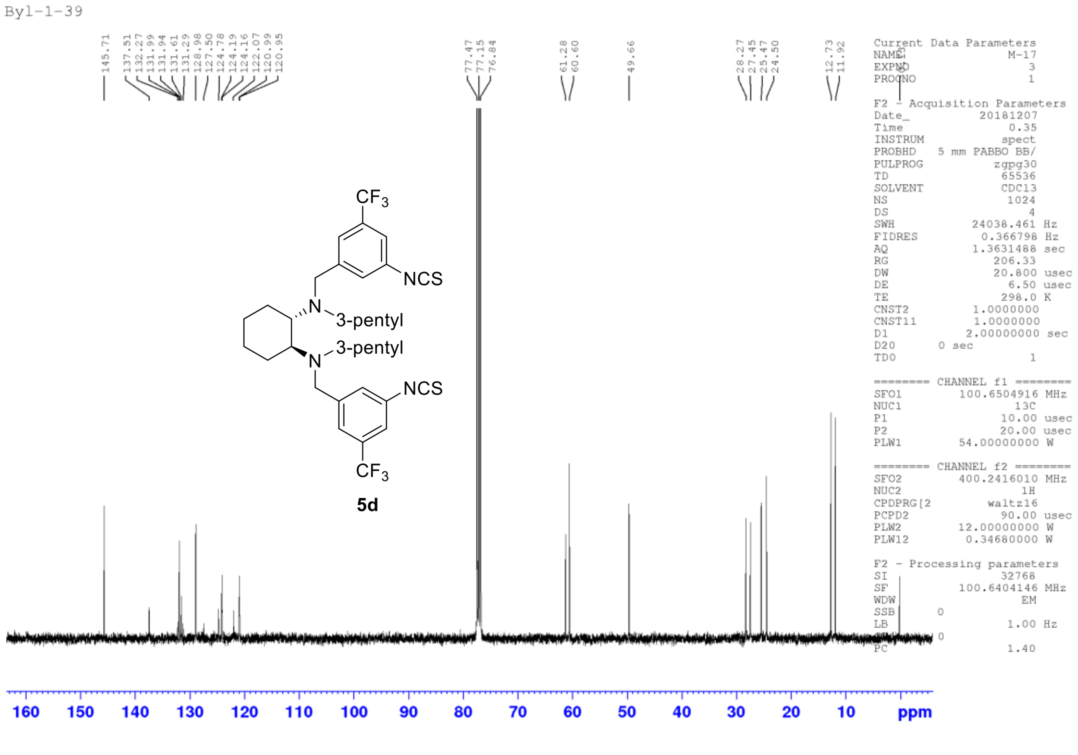

^{1}H and ^{13}C NMR of **5d** in $CDCl_3$.

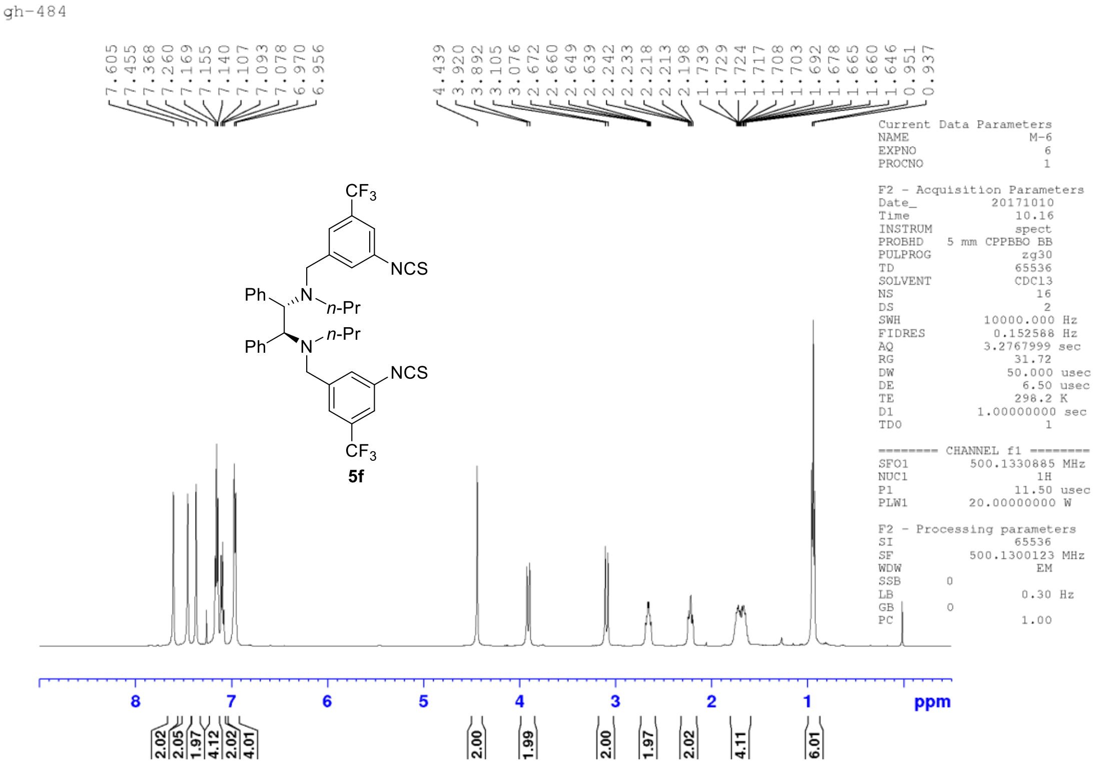

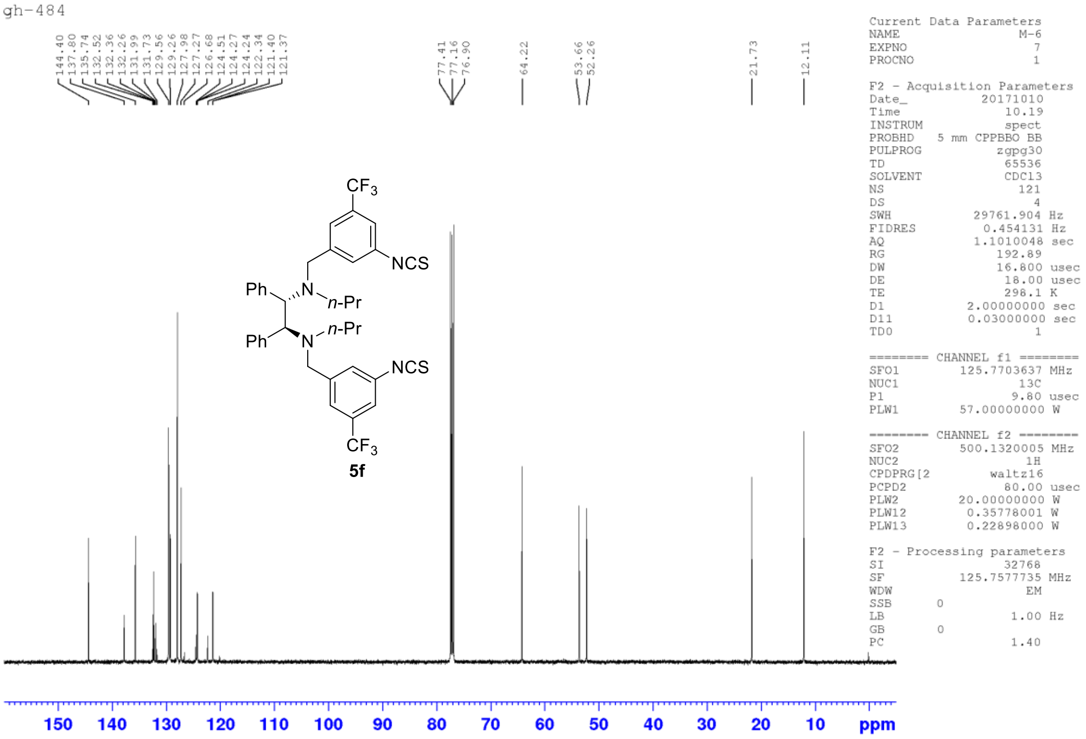

^{1}H and ^{13}C NMR of **5f** in $CDCl_3$.

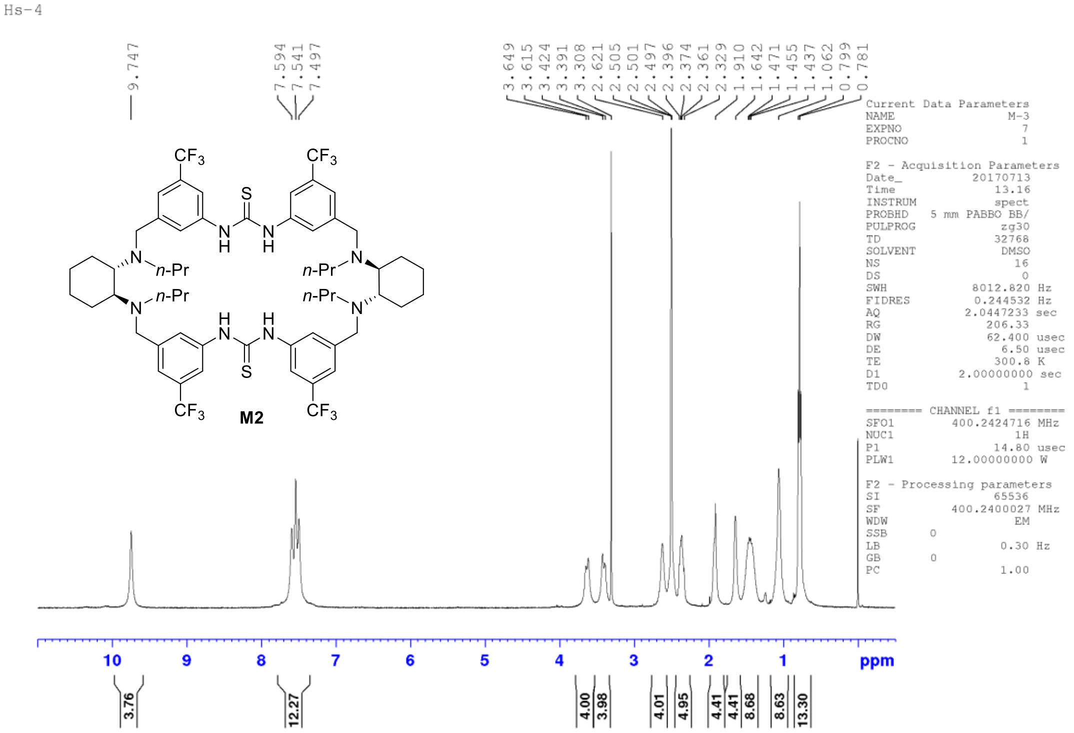

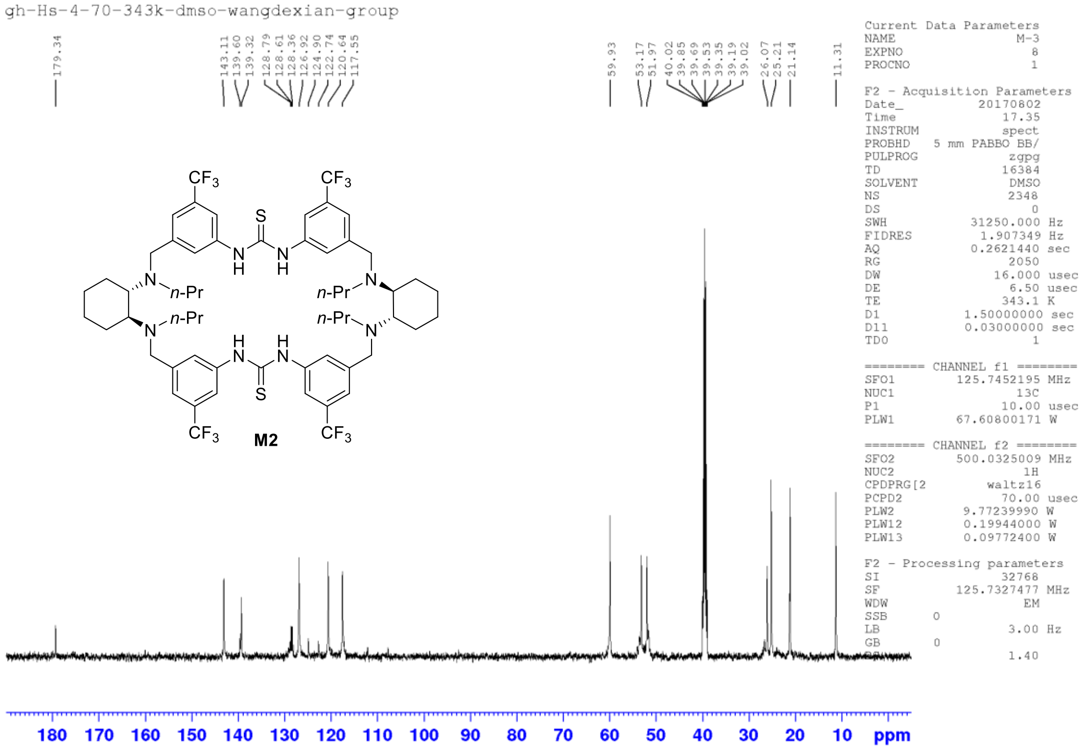

^{1}H and ^{13}C NMR of **M2** in DMSO-*d*$_6$.

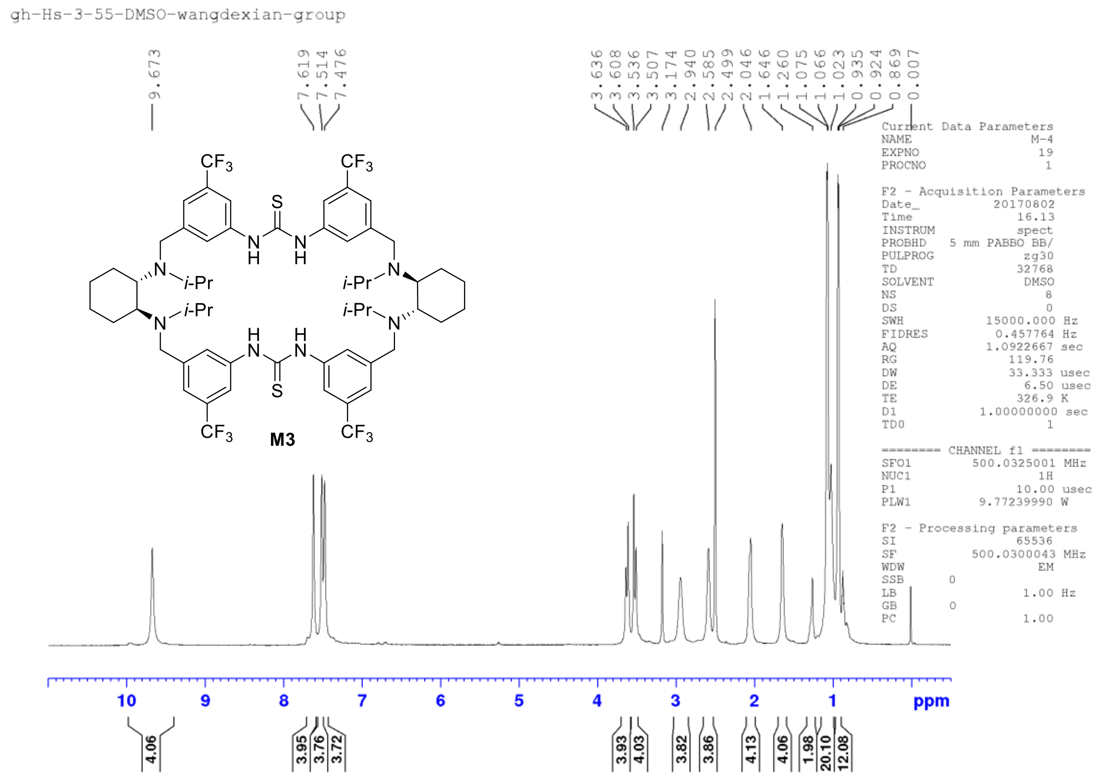

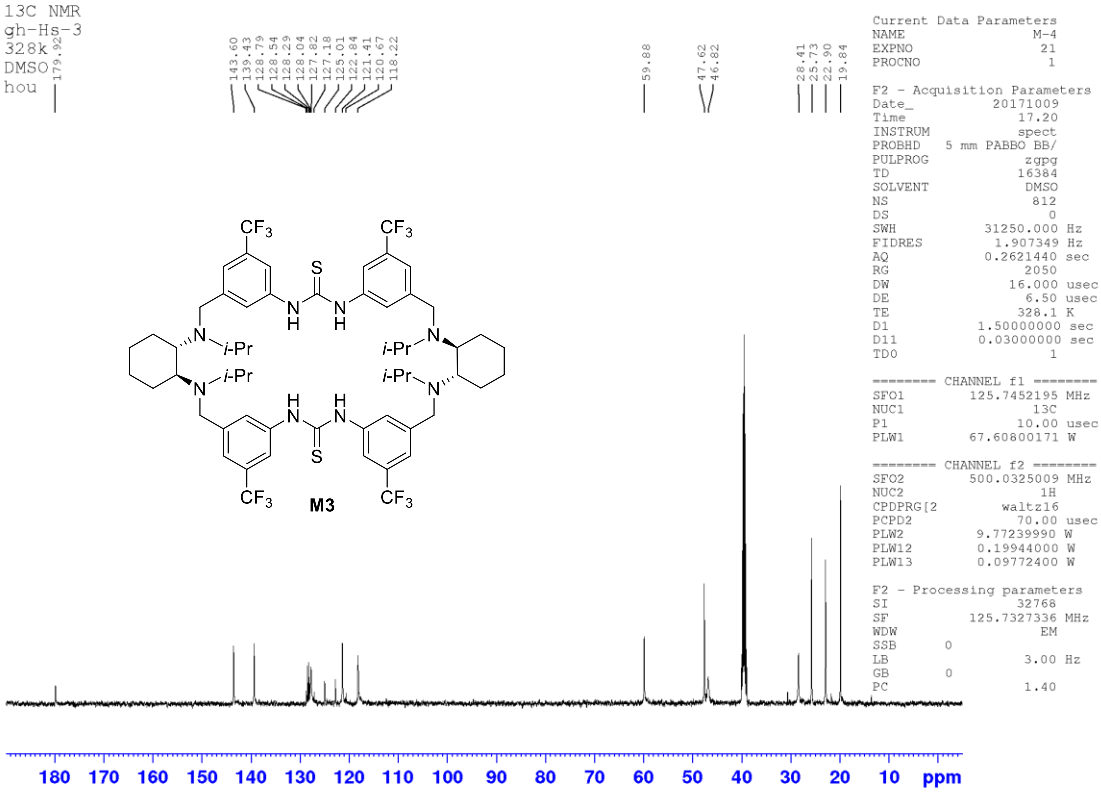

^{1}H and ^{13}C NMR of **M3** in DMSO-*d*$_6$.

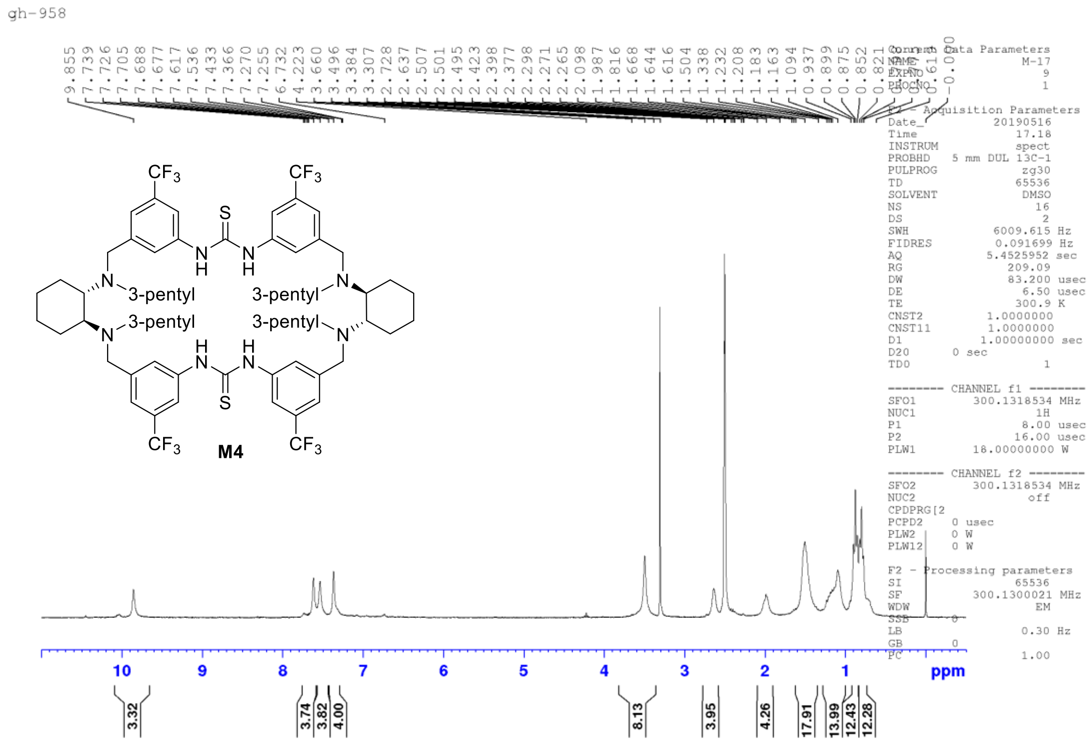

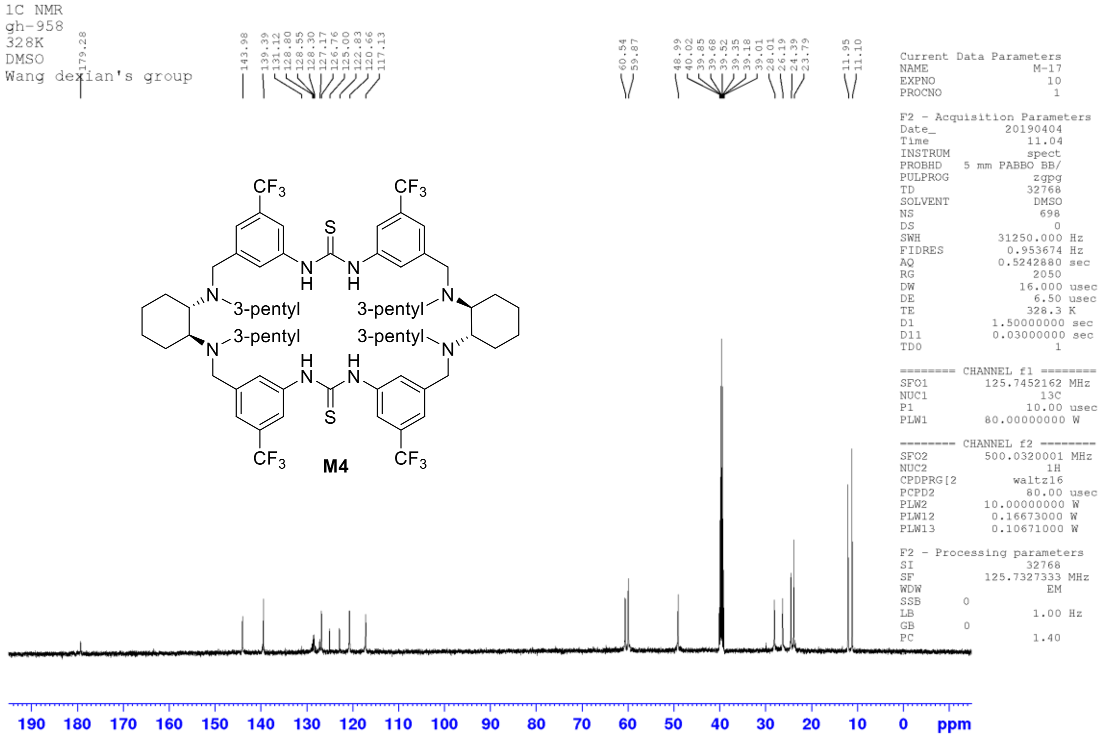

^{1}H and ^{13}C NMR of **M4** in DMSO-d_6.

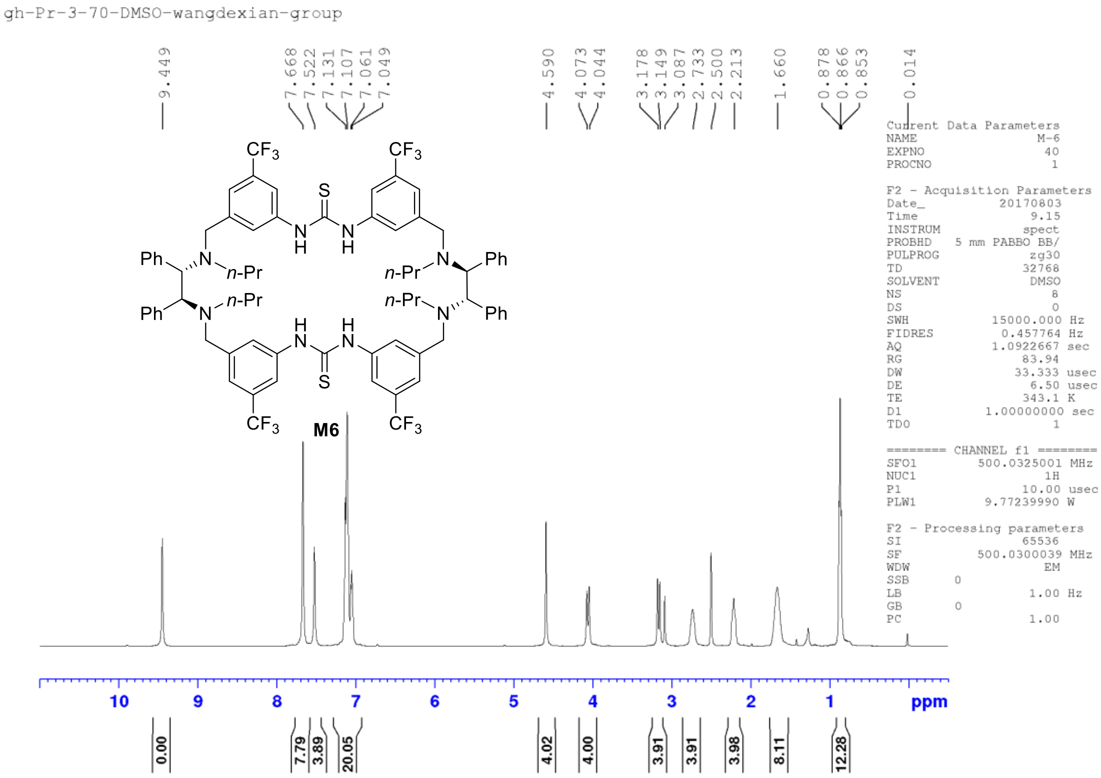

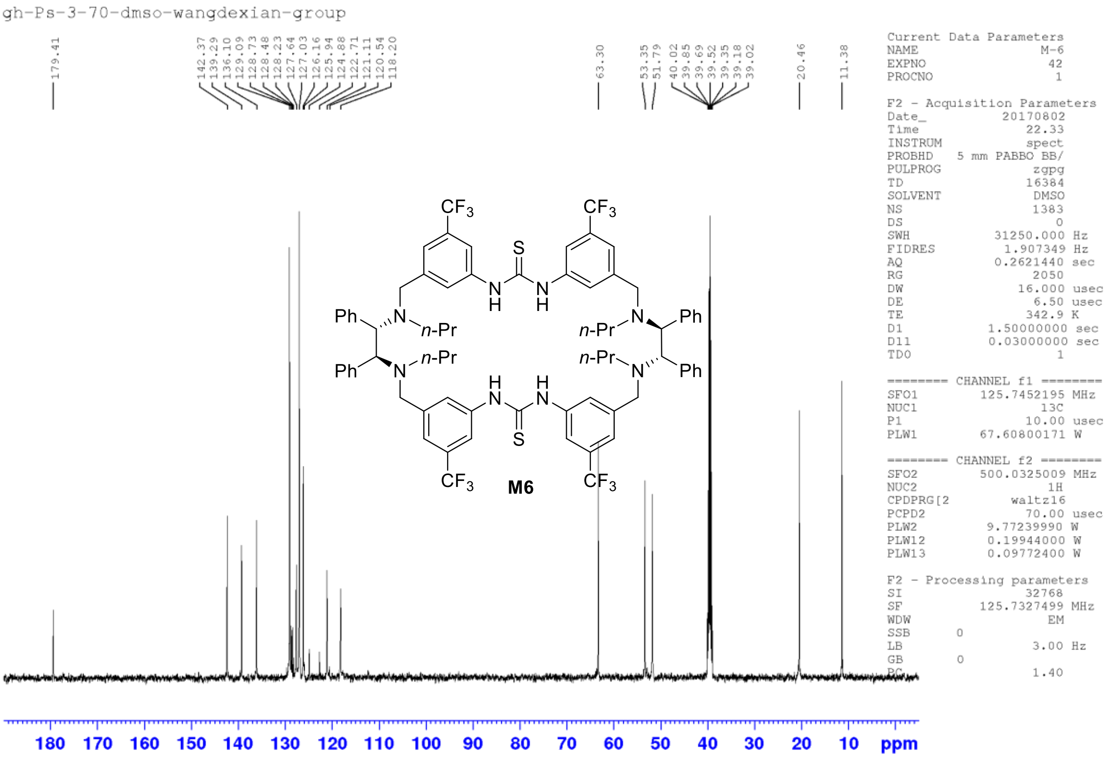

^{1}H and ^{13}C NMR of **M6** in DMSO-d_6.

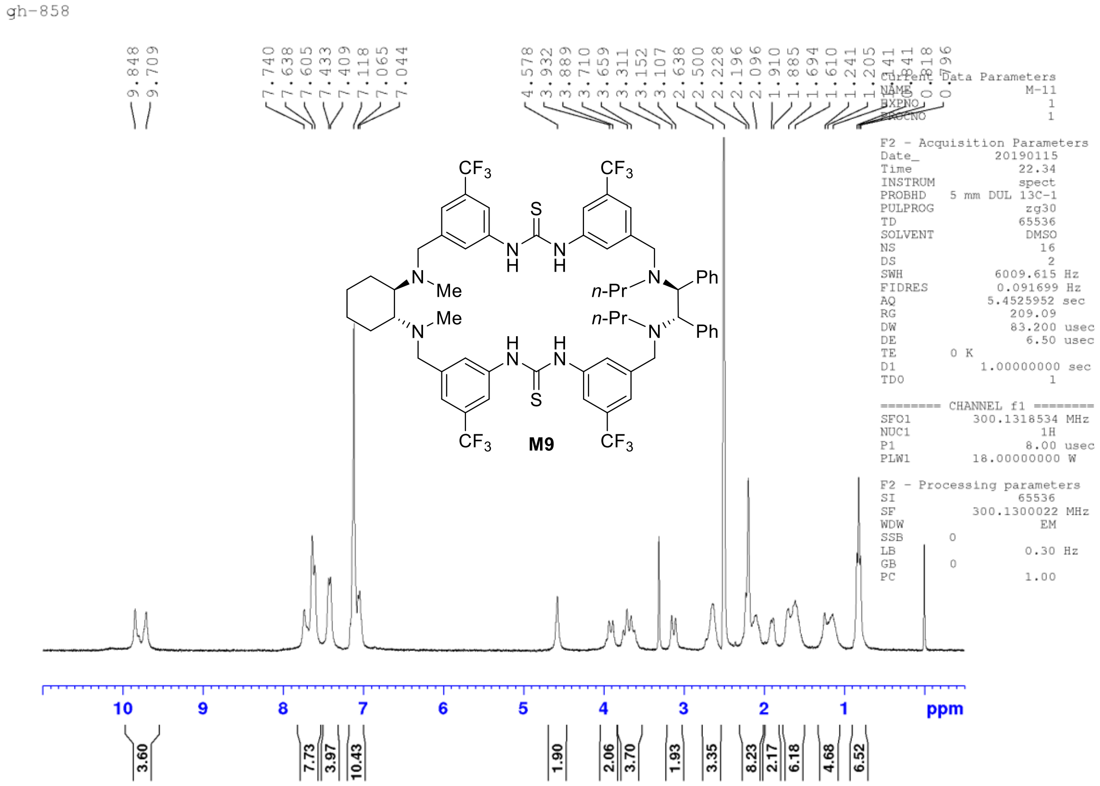

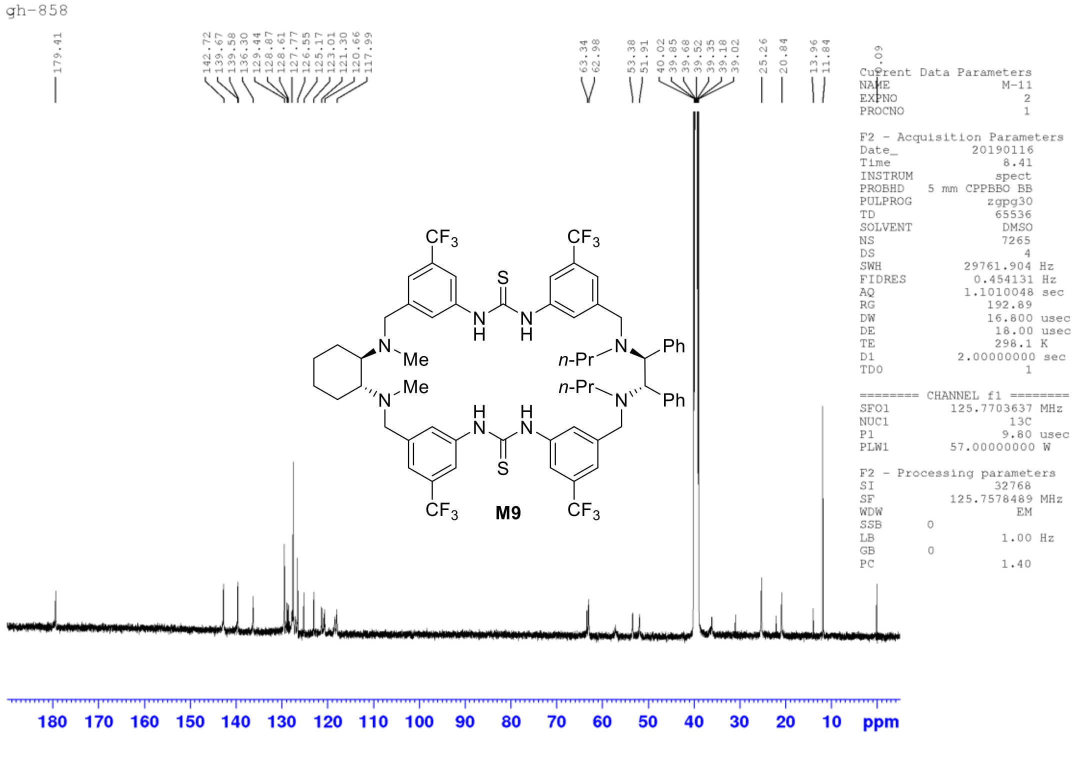

^{1}H and ^{13}C NMR of **M9** in DMSO-d_6.

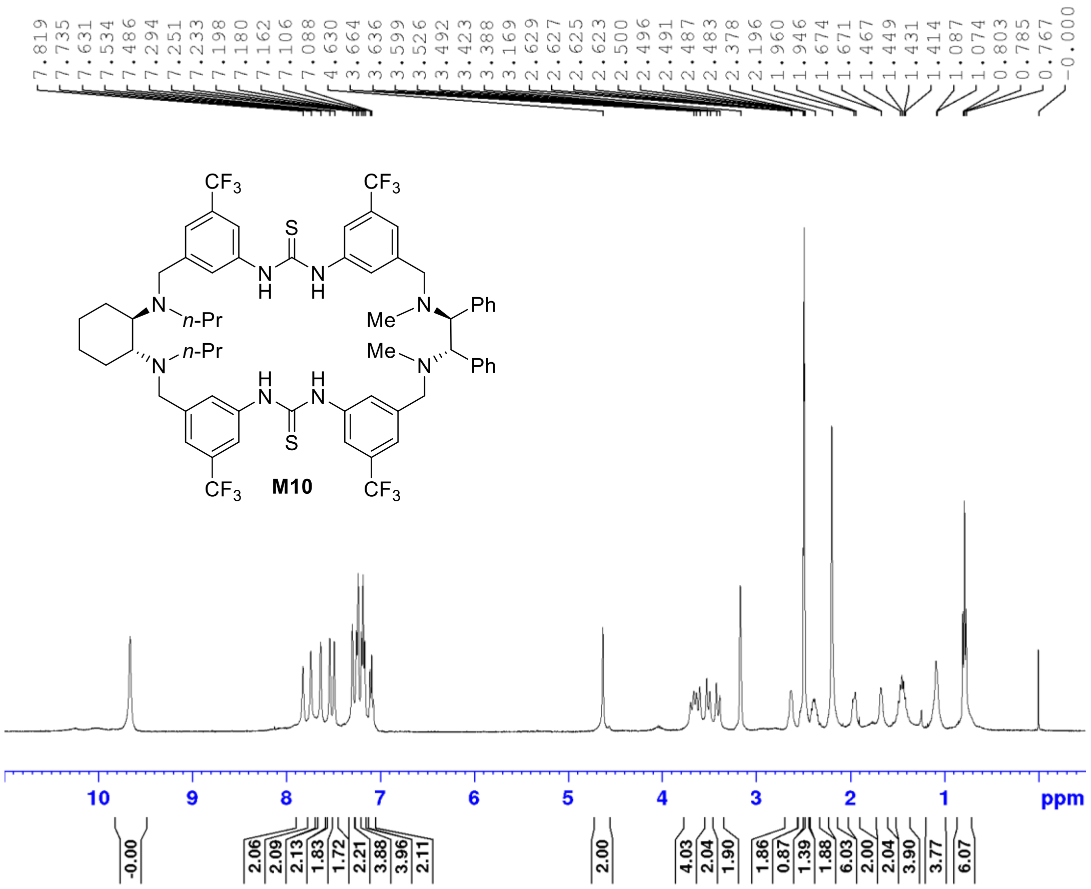

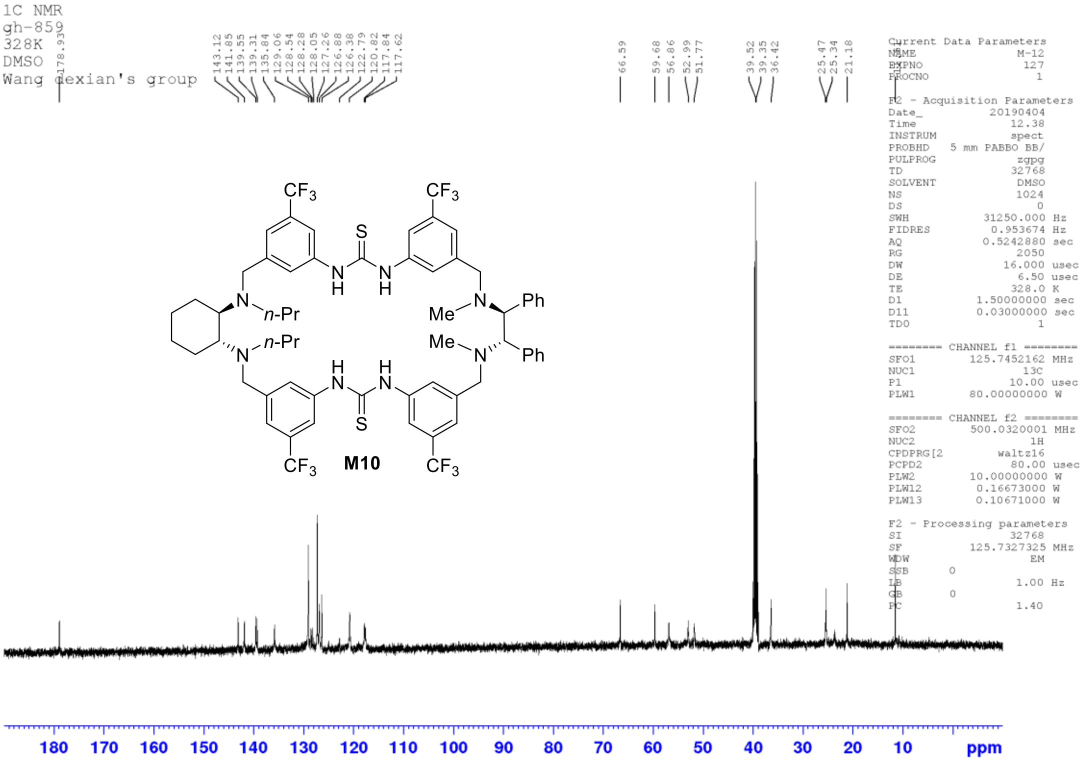

^{1}H and ^{13}C NMR of **M10** in DMSO-d_6.

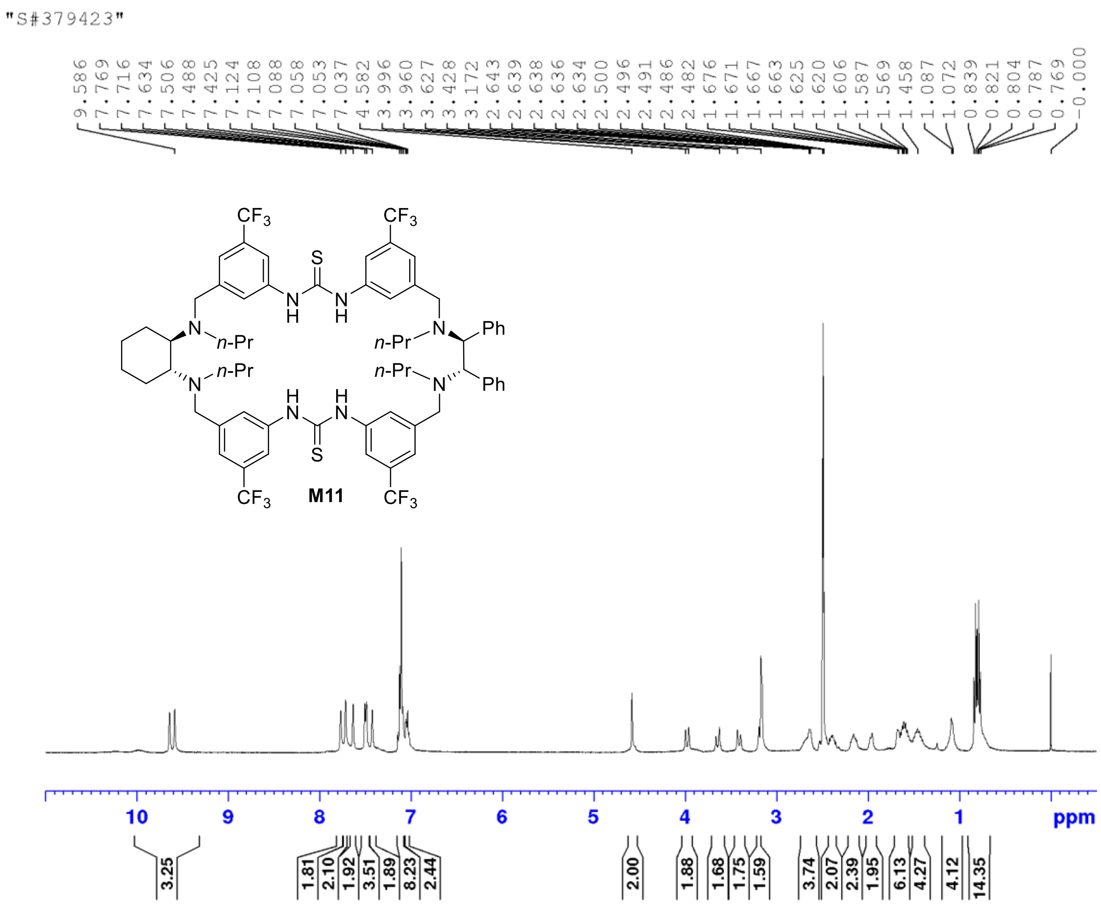

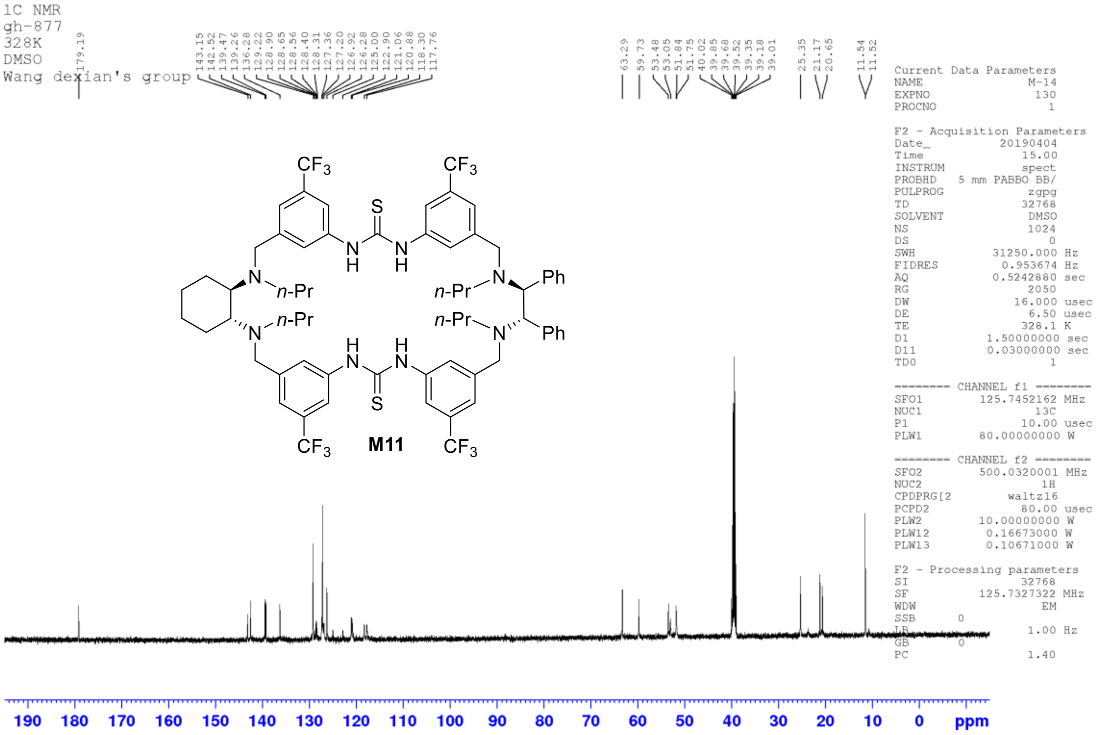

^{1}H and ^{13}C NMR of **M11** in DMSO-d_6.

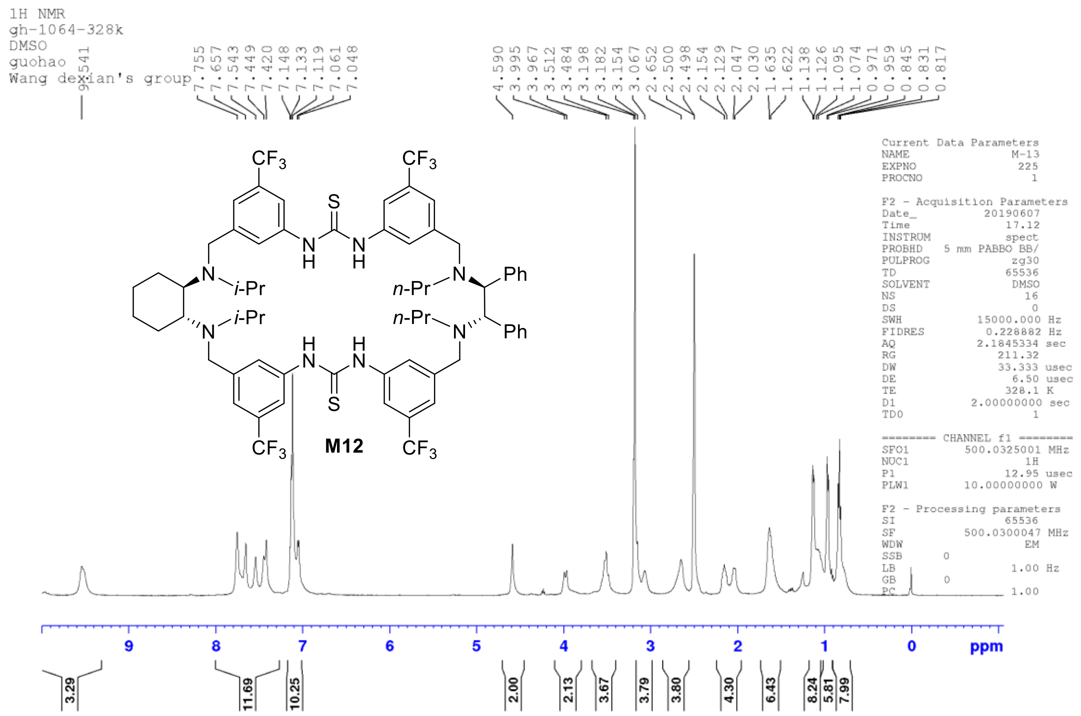

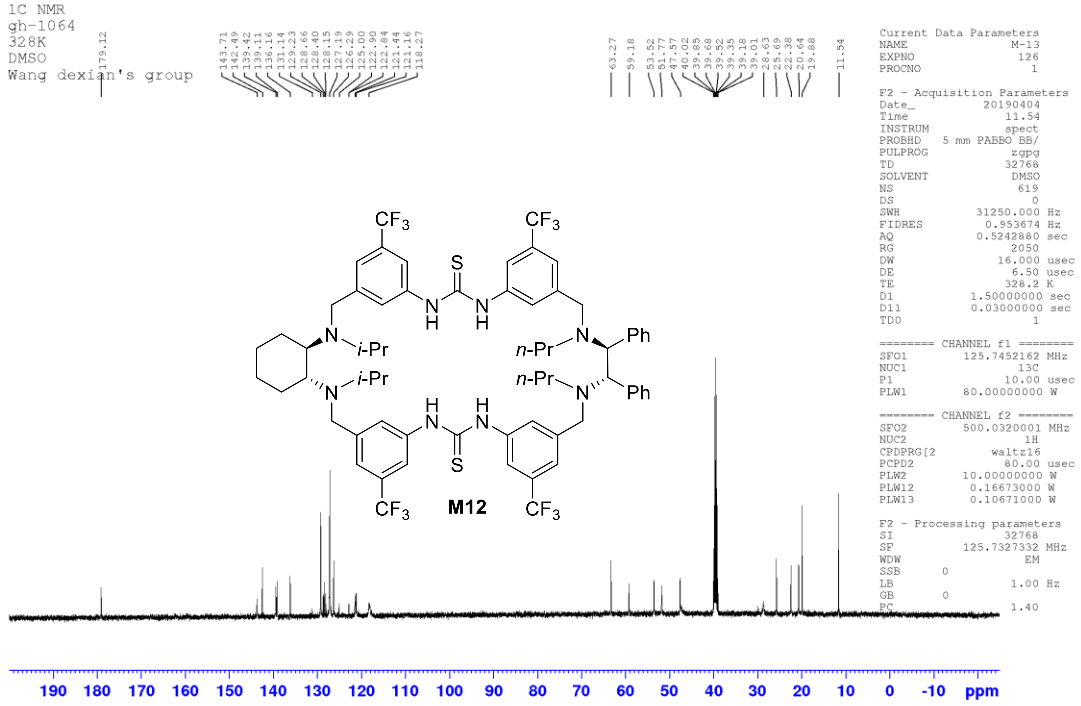

^{1}H and ^{13}C NMR of **M12** in DMSO-d_6.

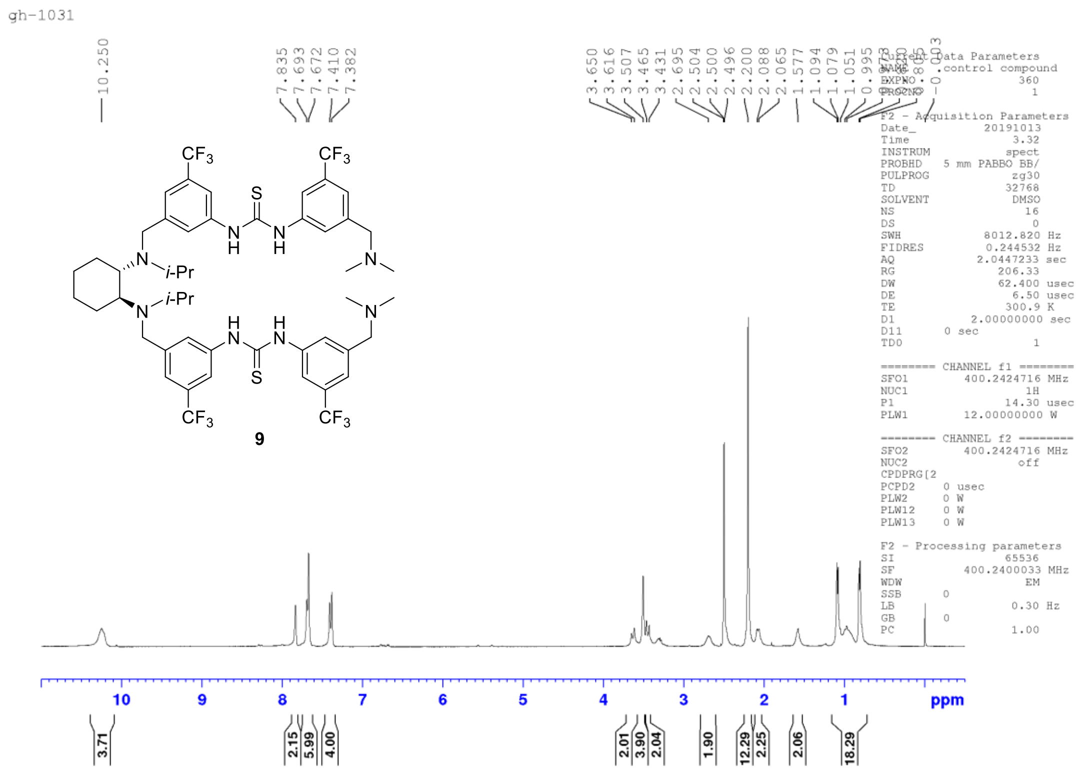

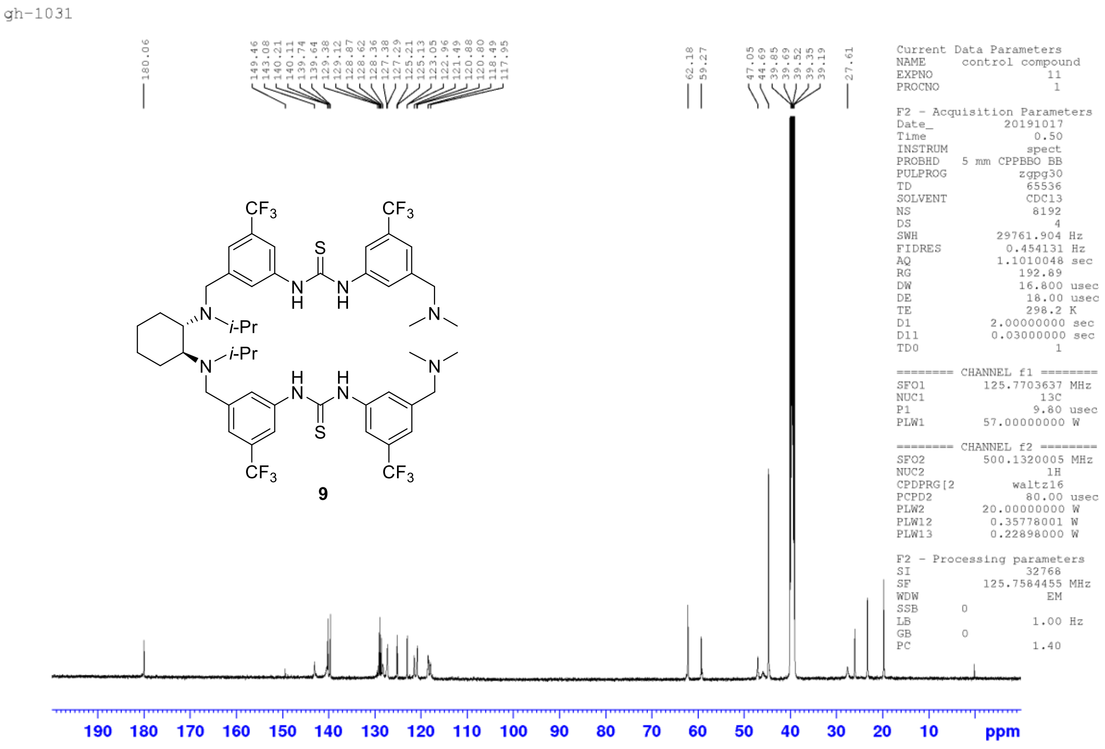

^{1}H and ^{13}C NMR of **9** in DMSO-d_6.

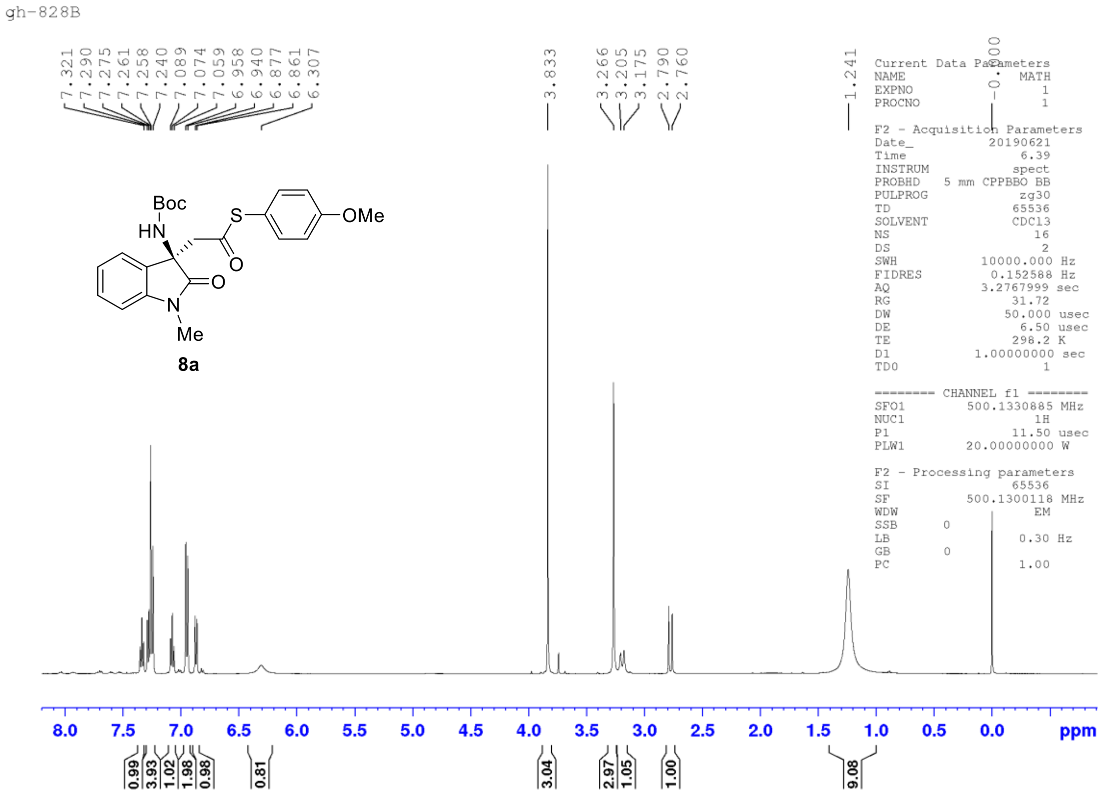

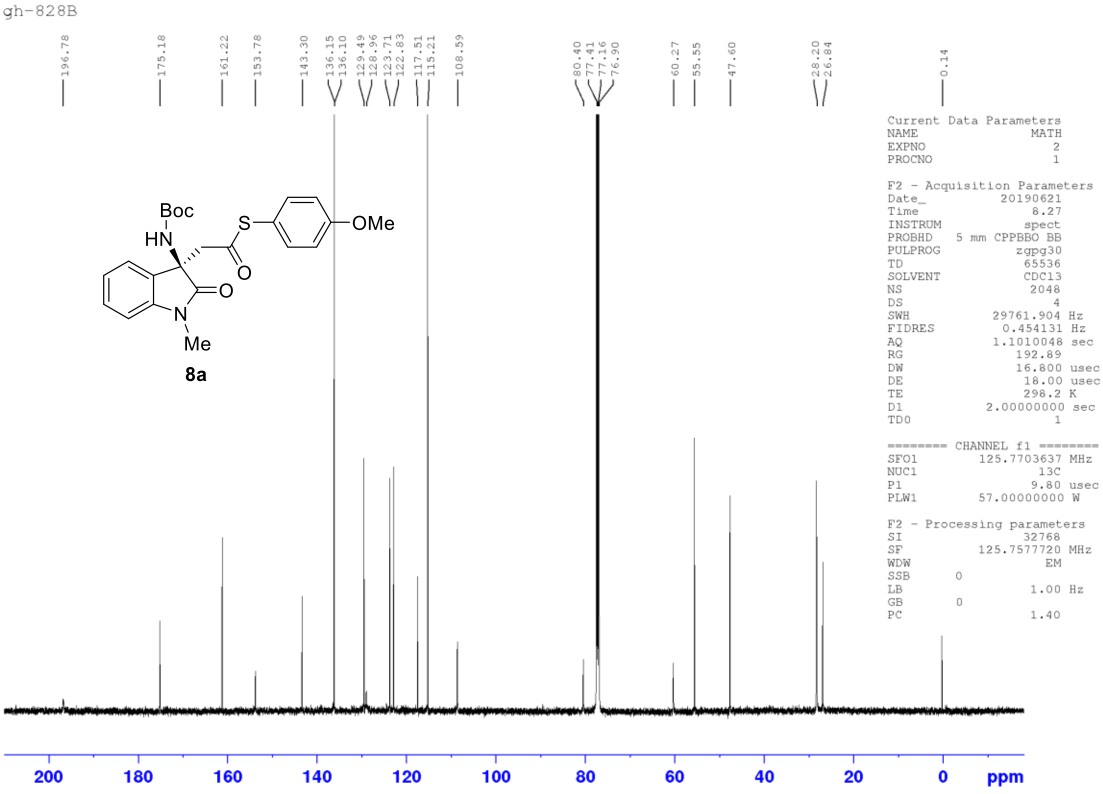

^{1}H and ^{13}C NMR of **8a** in $CDCl_3$.

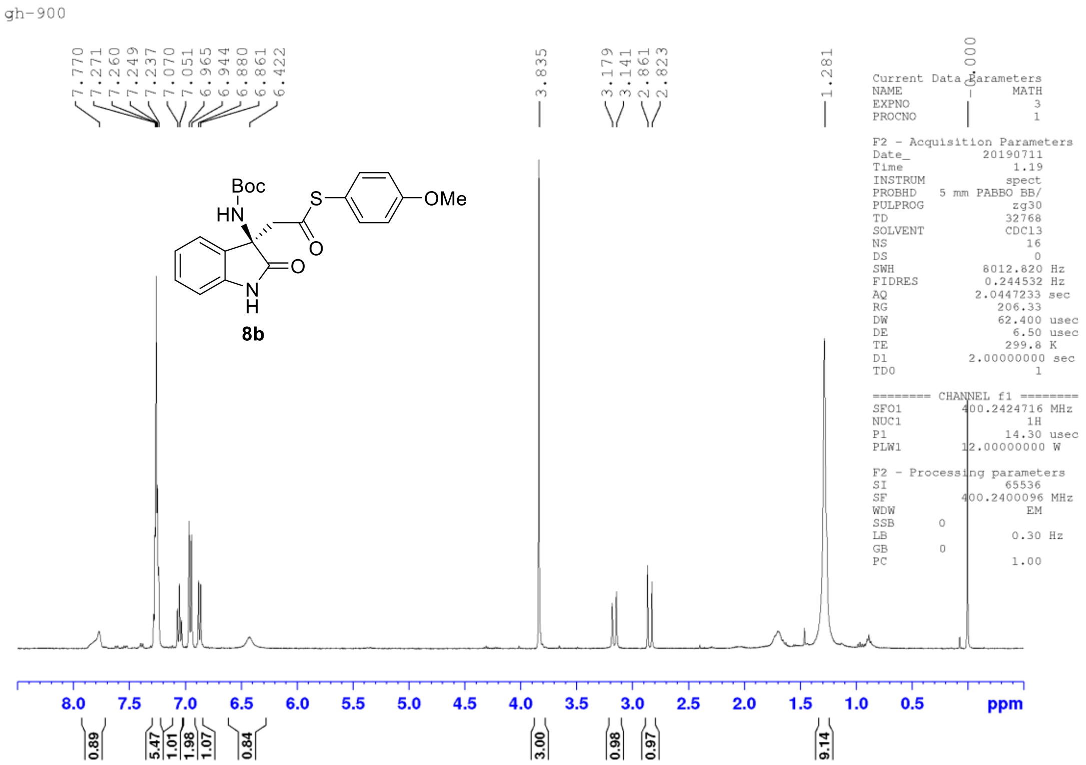

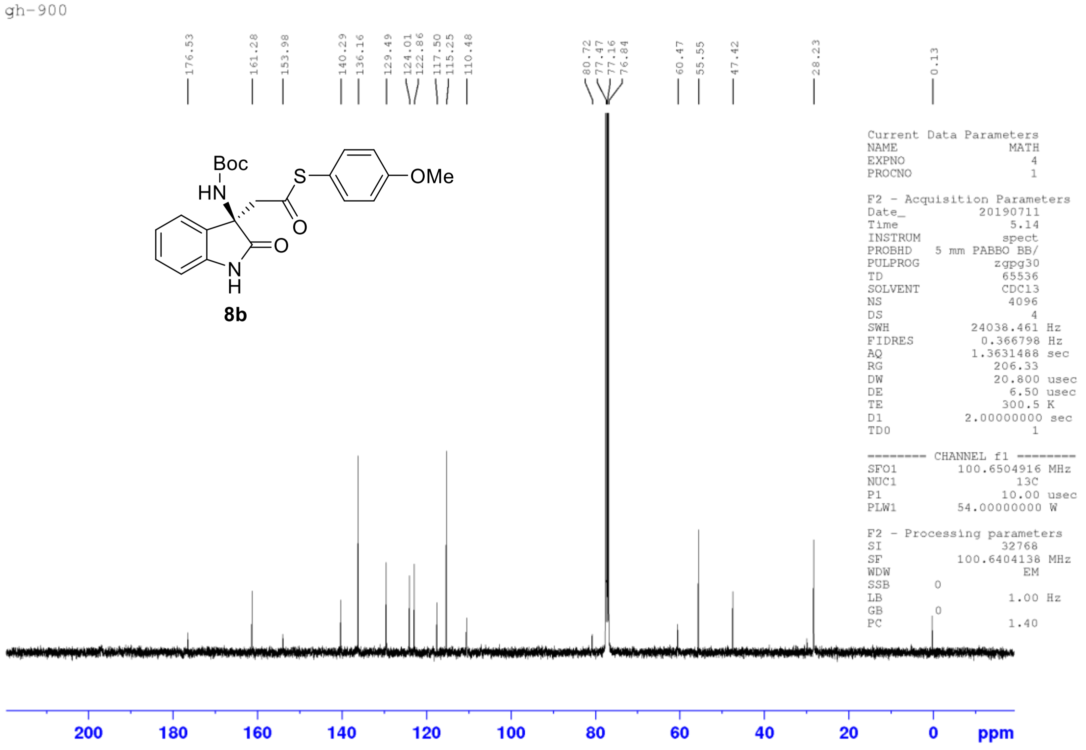

^{1}H and ^{13}C NMR of **8b** in $CDCl_3$.

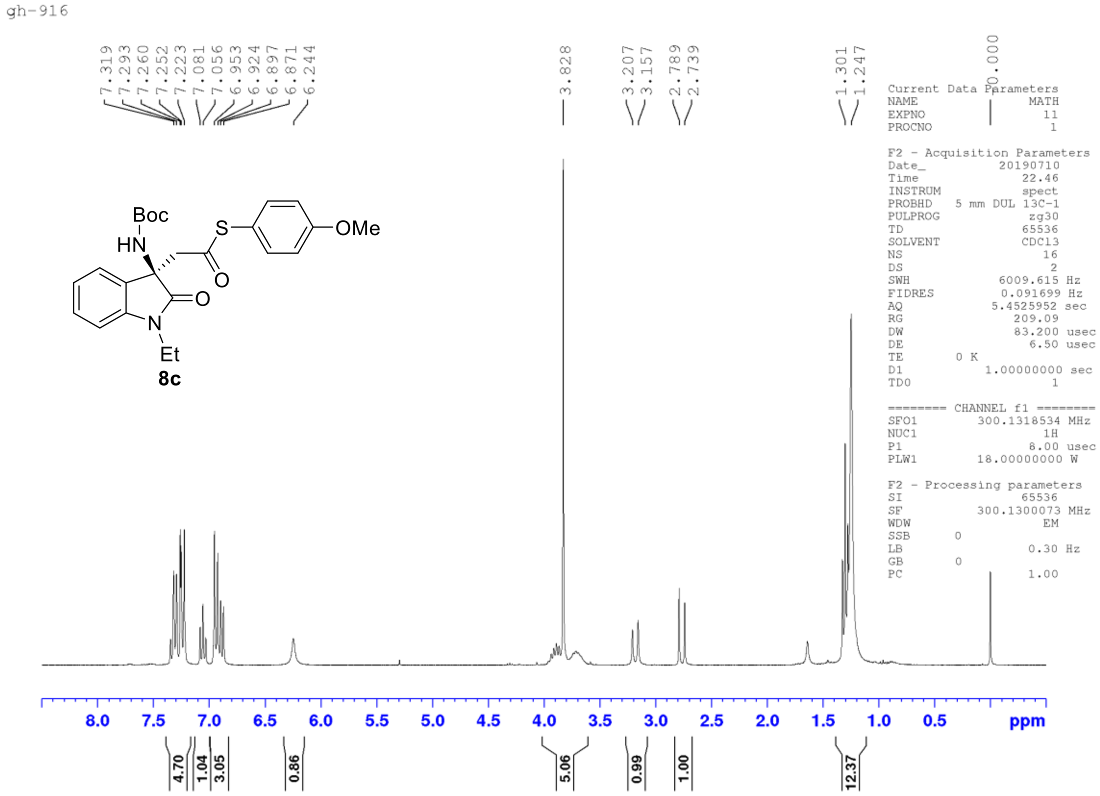

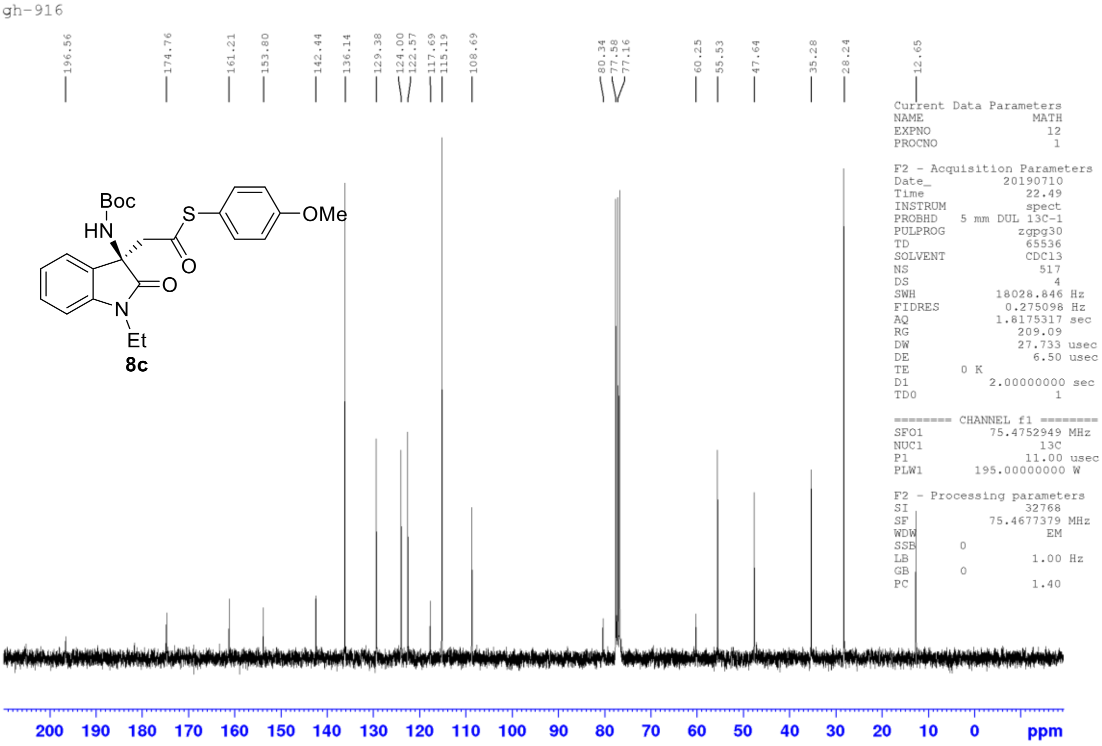

^{1}H and ^{13}C NMR of **8c** in $CDCl_3$.

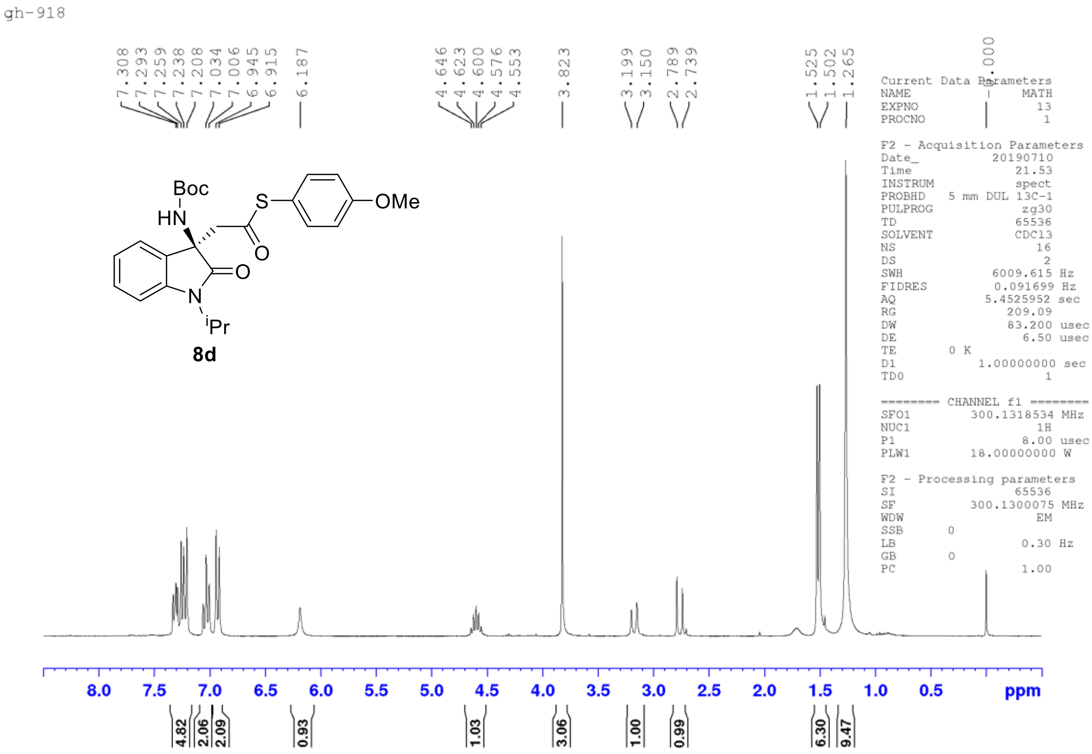

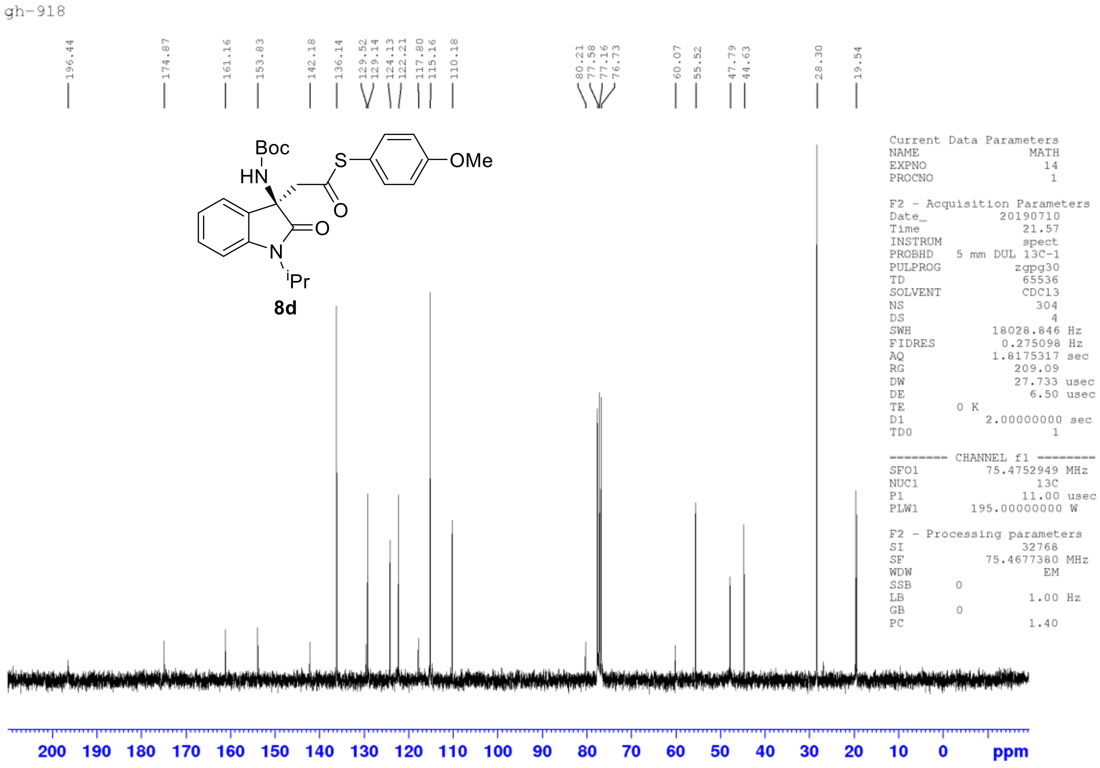

^{1}H and ^{13}C NMR of **8d** in $CDCl_3$.

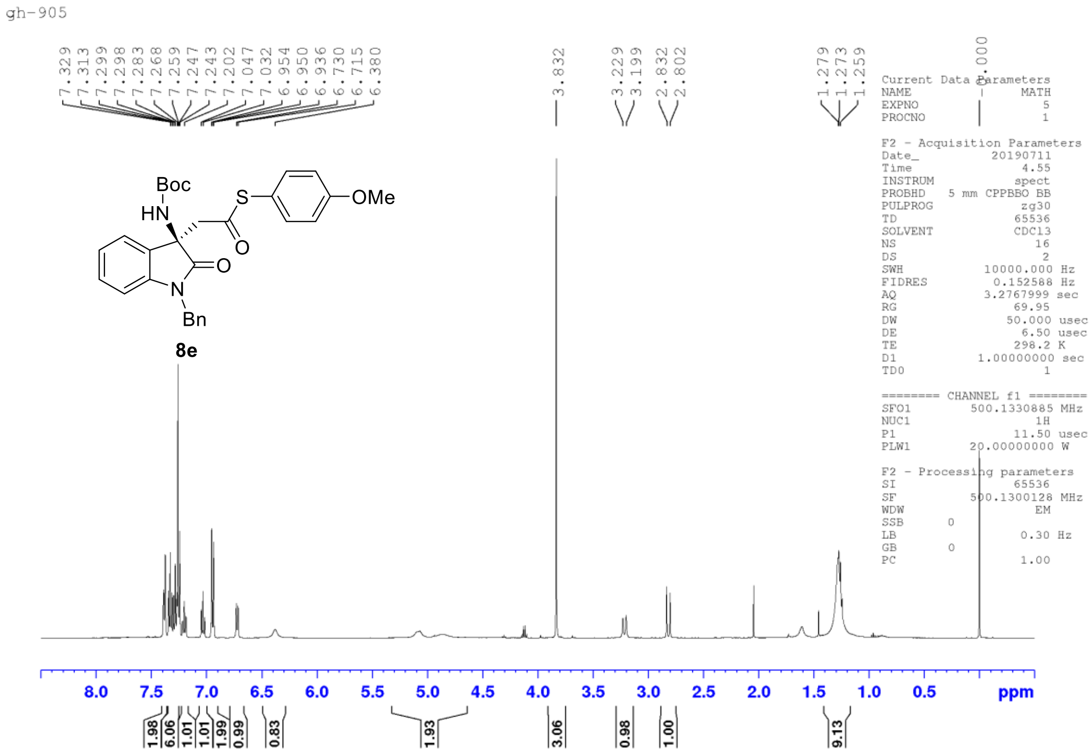

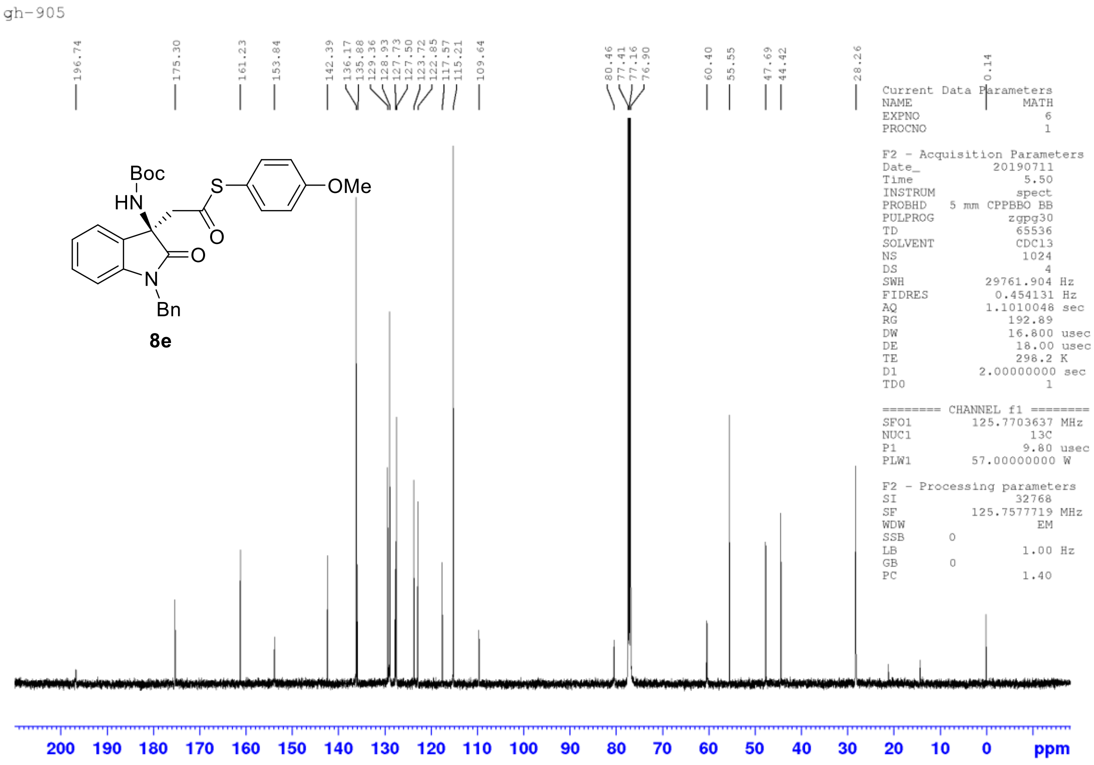

^{1}H and ^{13}C NMR of **8e** in $CDCl_3$.

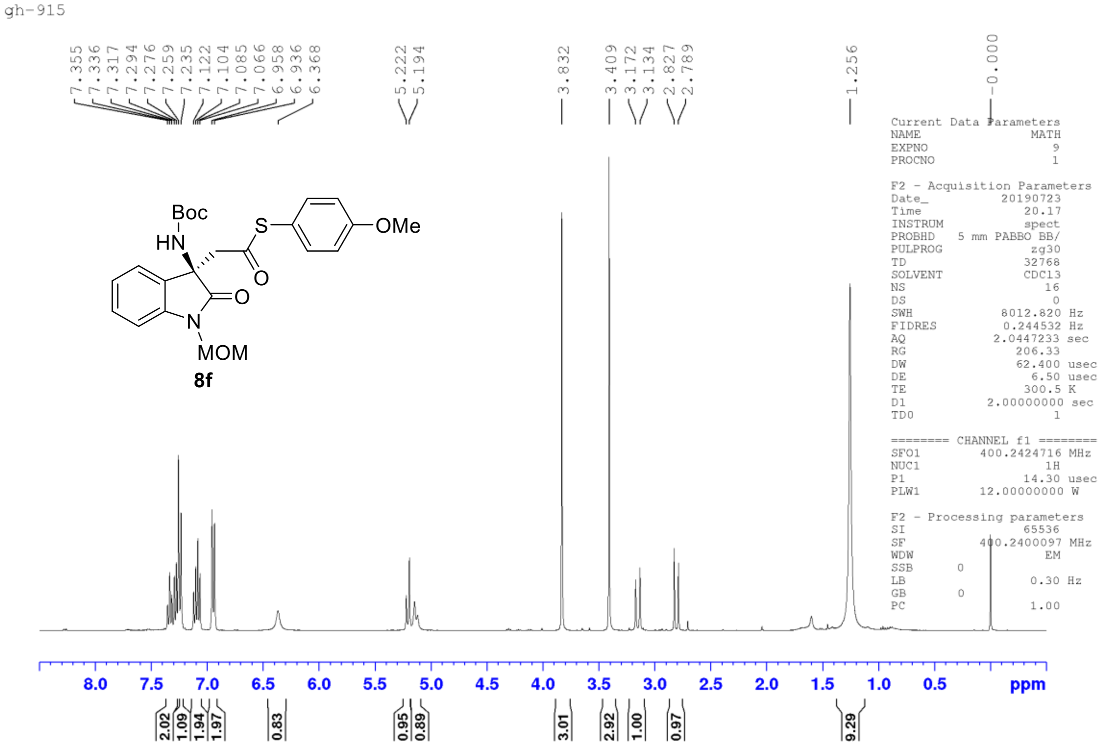

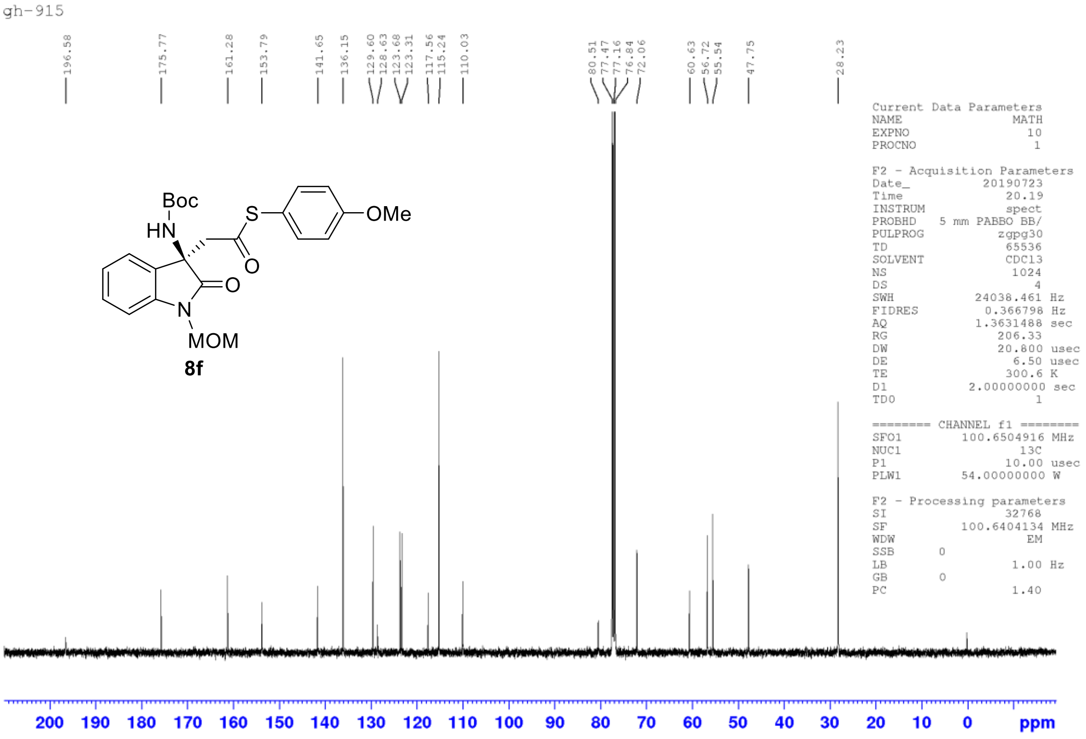

^{1}H and ^{13}C NMR of **8f** in $CDCl_3$.

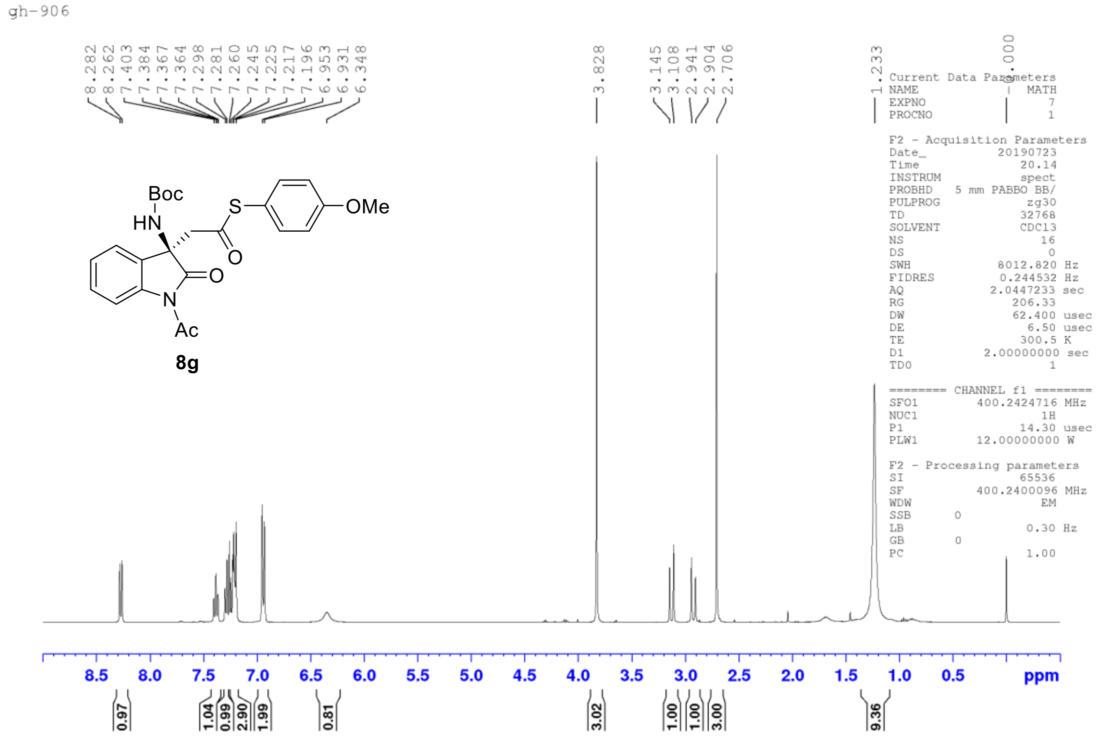

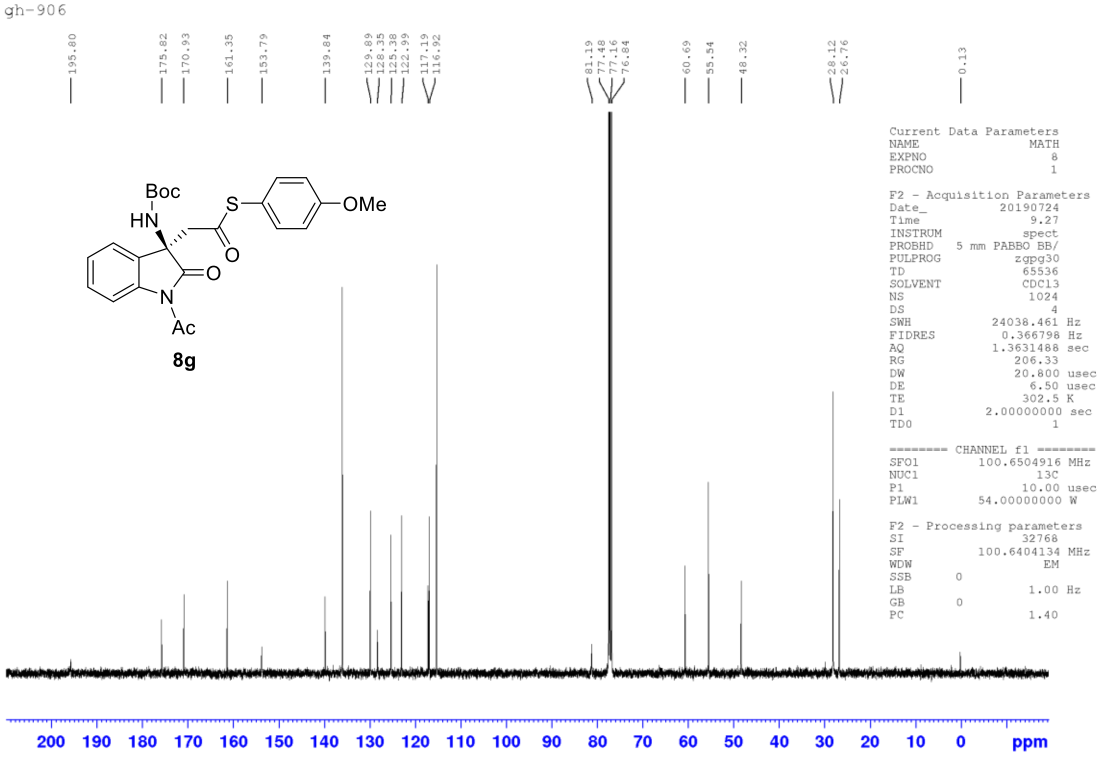

^{1}H and ^{13}C NMR of **8g** in $CDCl_3$.

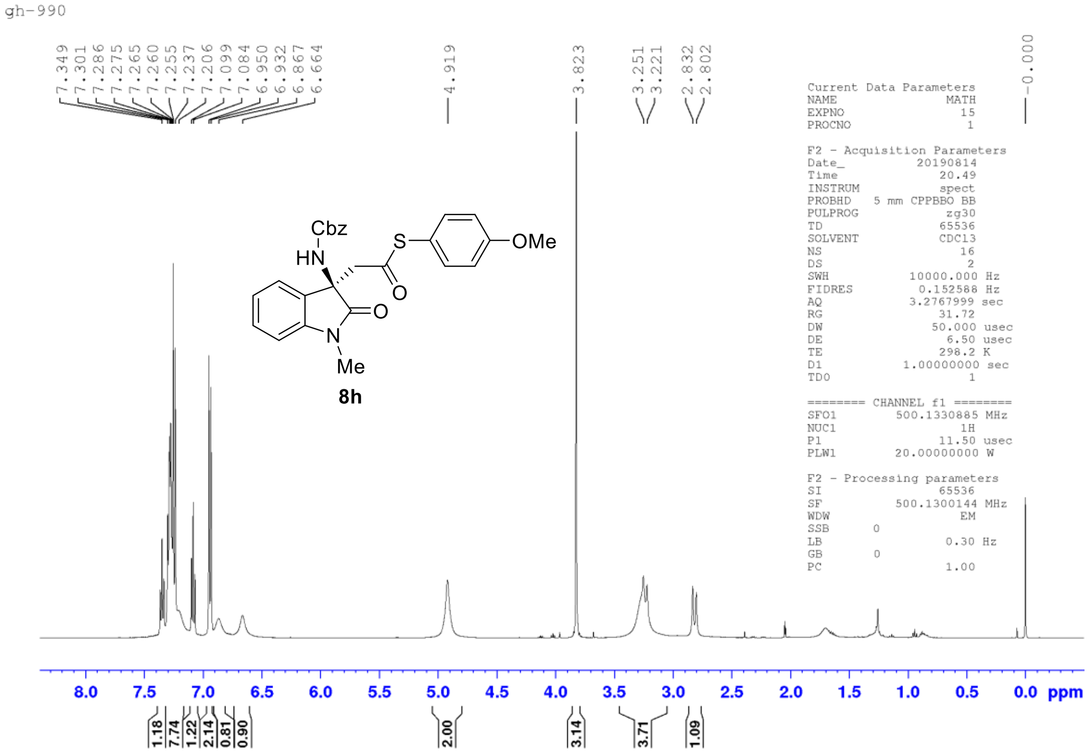

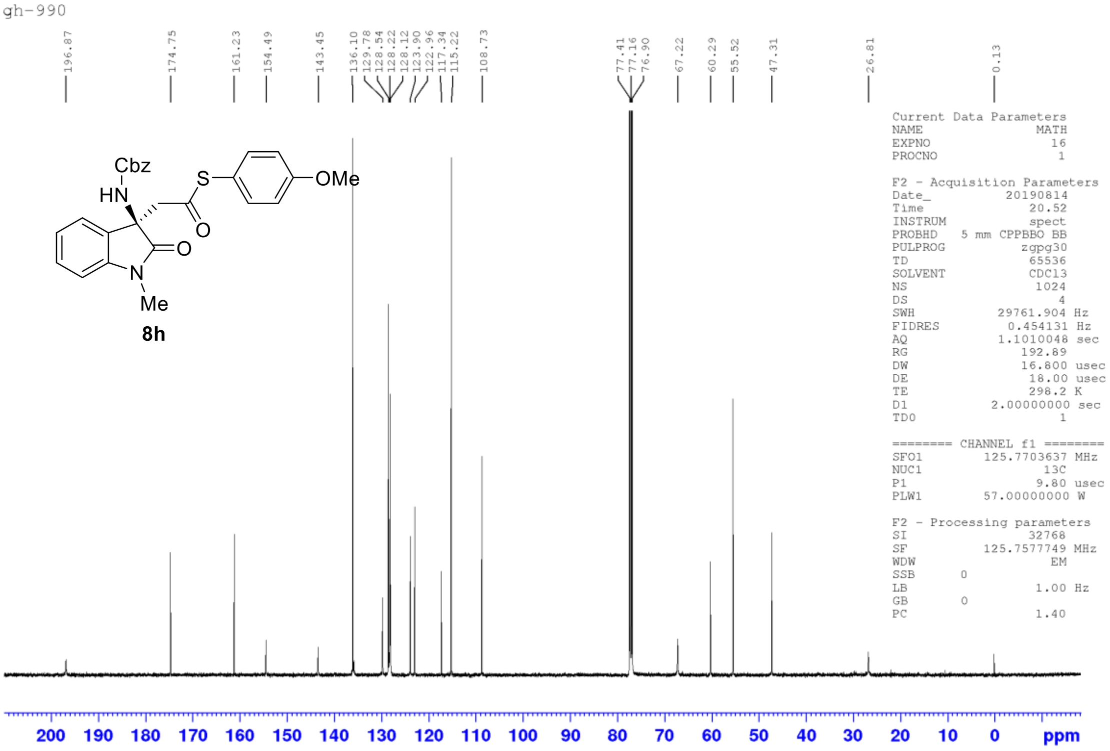

^{1}H and ^{13}C NMR of **8h** in $CDCl_3$.

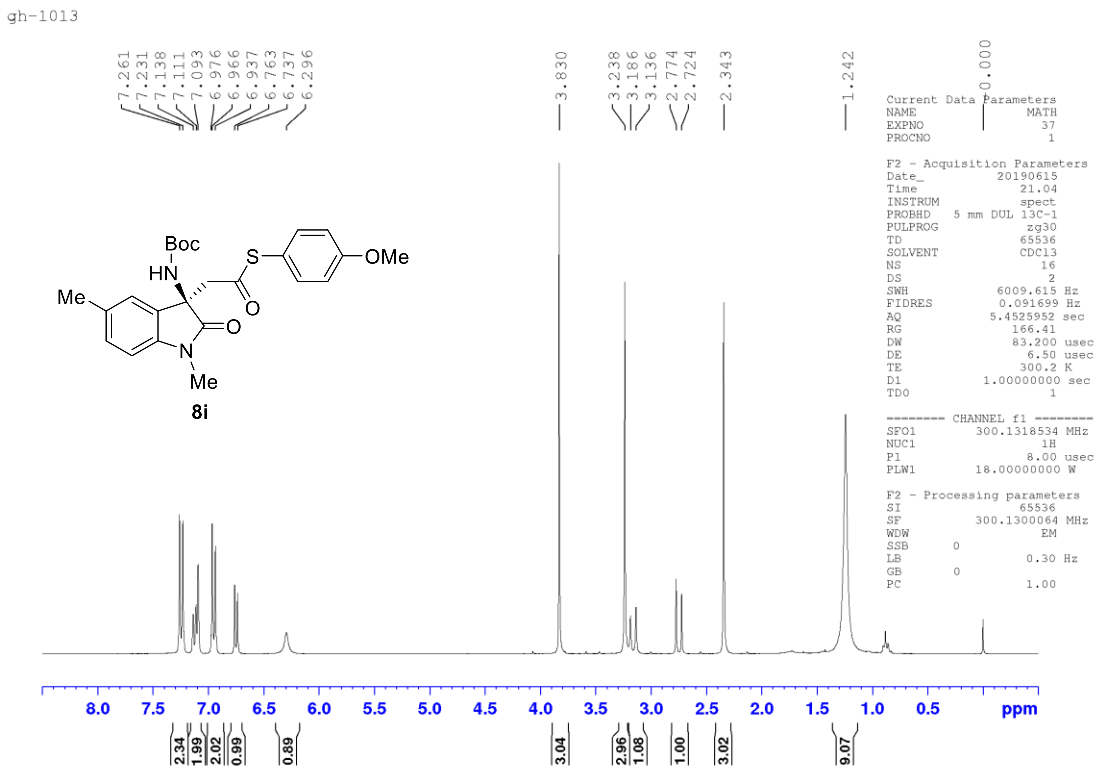

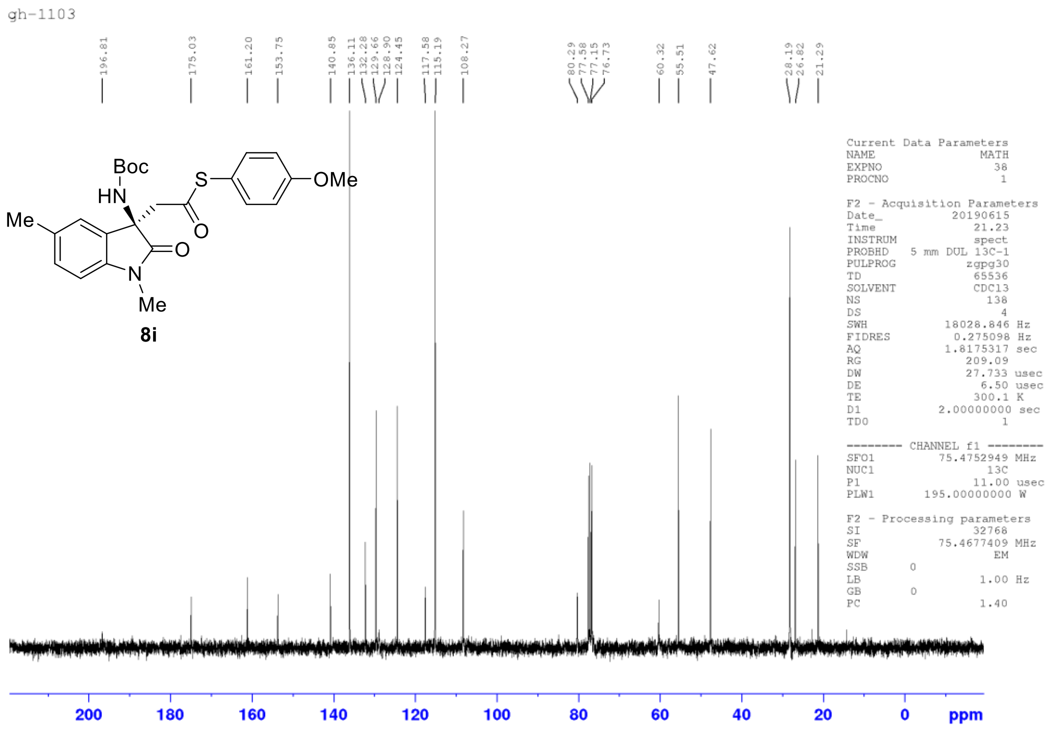

^{1}H and ^{13}C NMR of **8i** in $CDCl_3$.

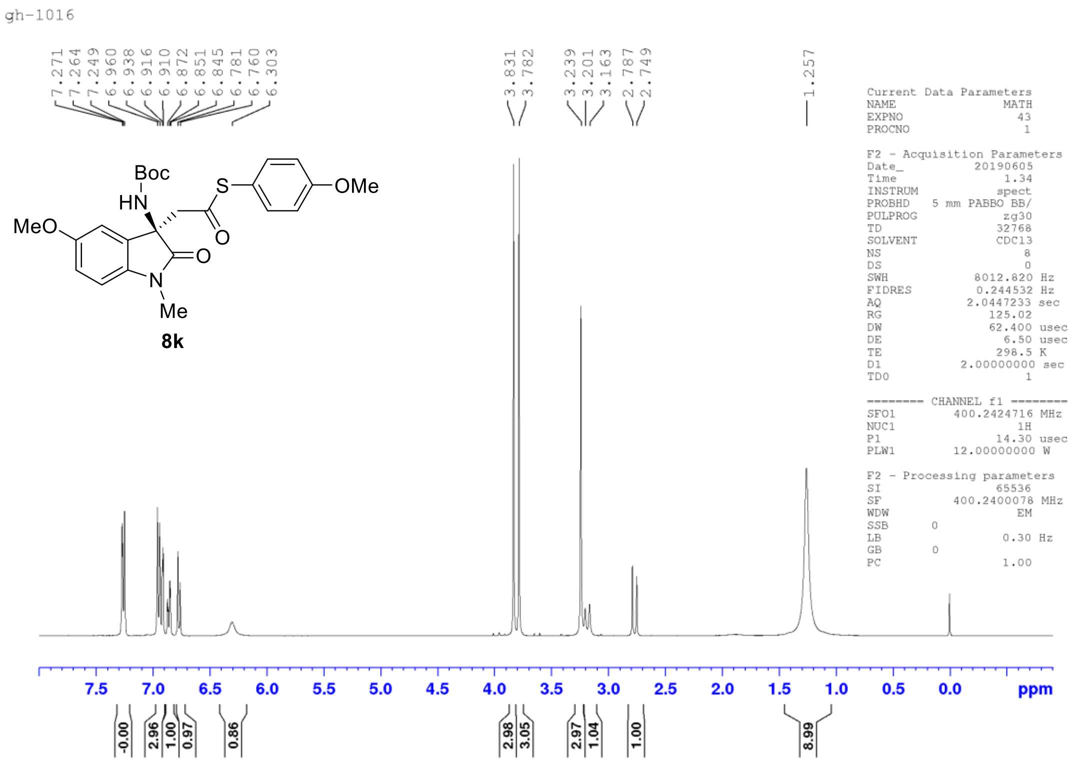

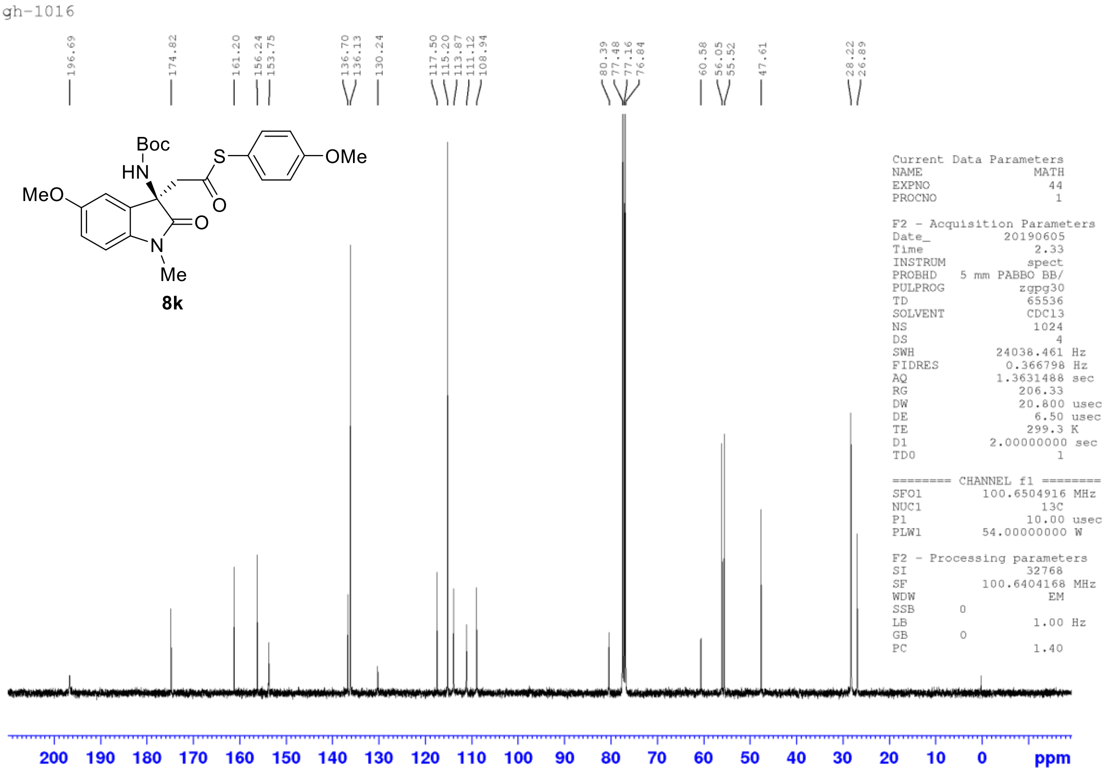

^{1}H and ^{13}C NMR of **8k** in $CDCl_3$.

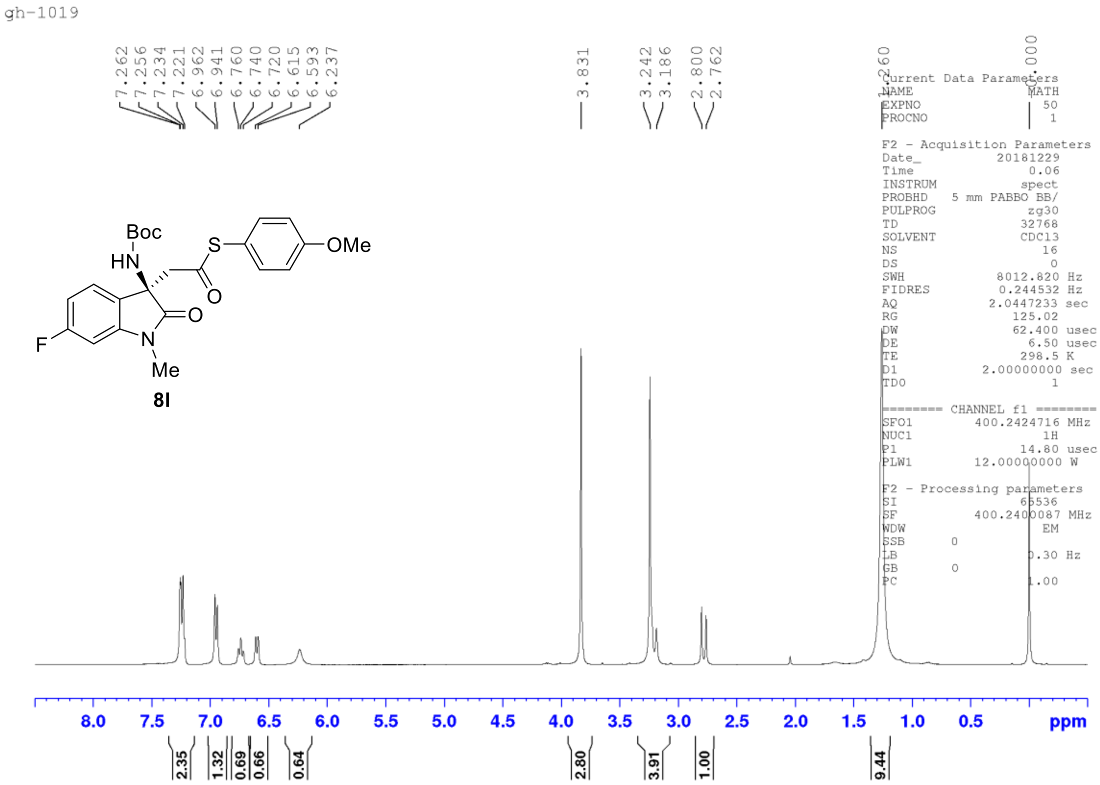

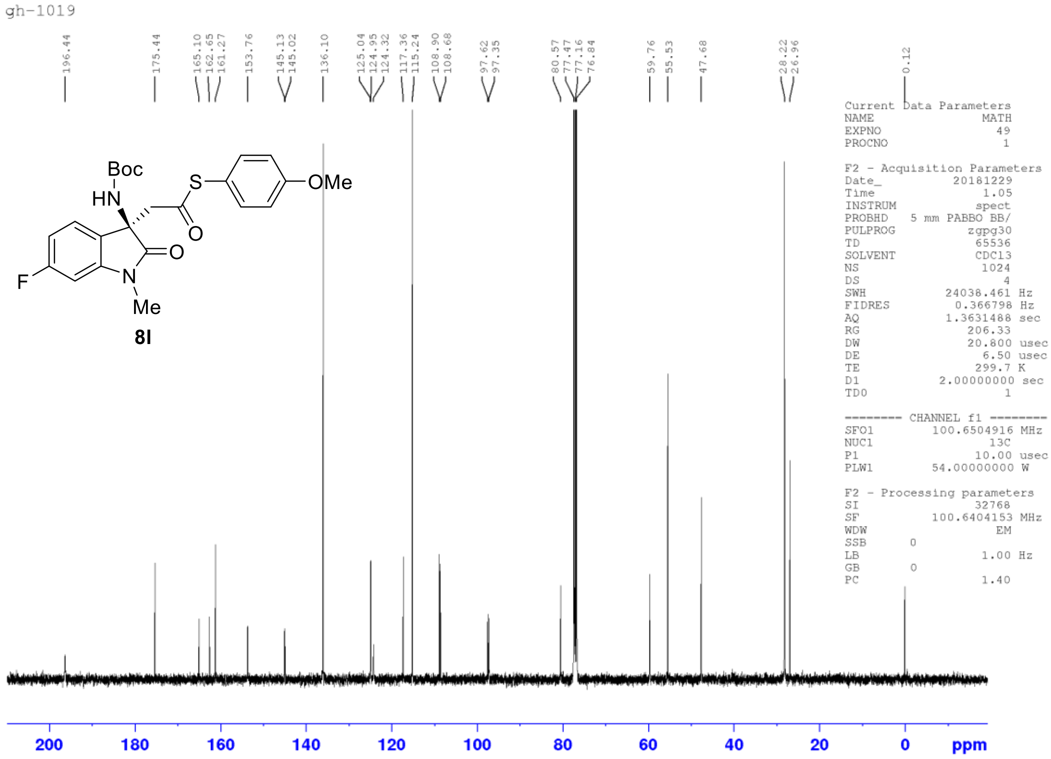

^{1}H and ^{13}C NMR of **8l** in $CDCl_3$.

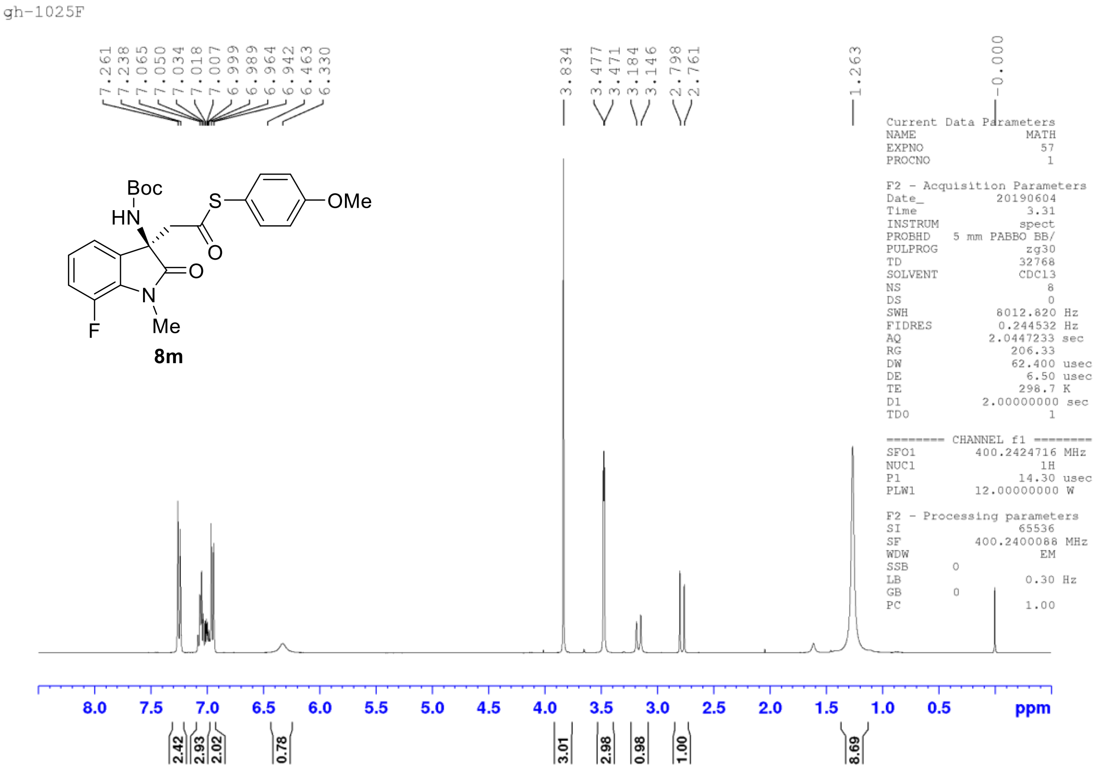

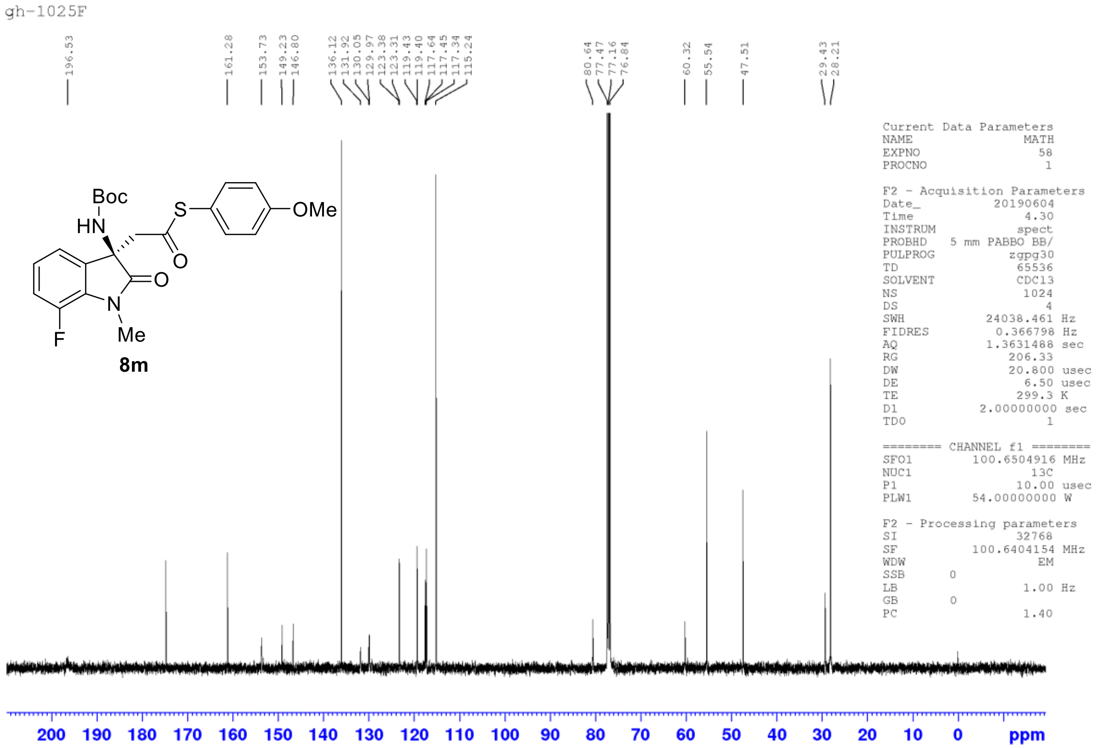

^{1}H and ^{13}C NMR of **8m** in $CDCl_3$.

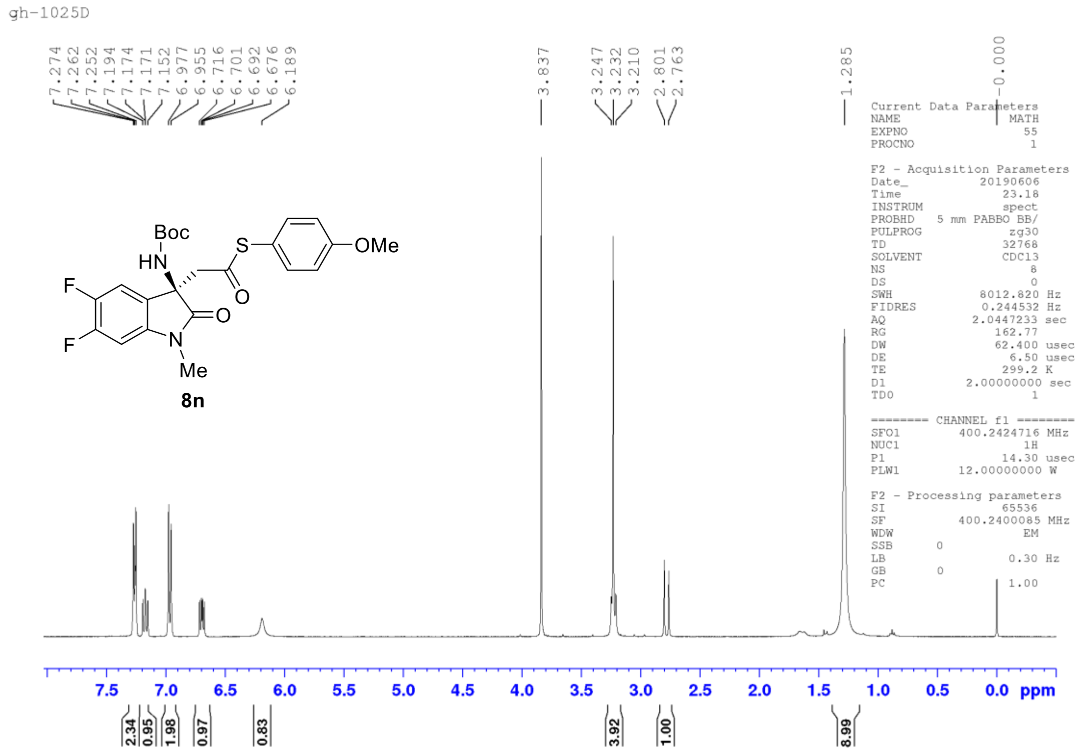

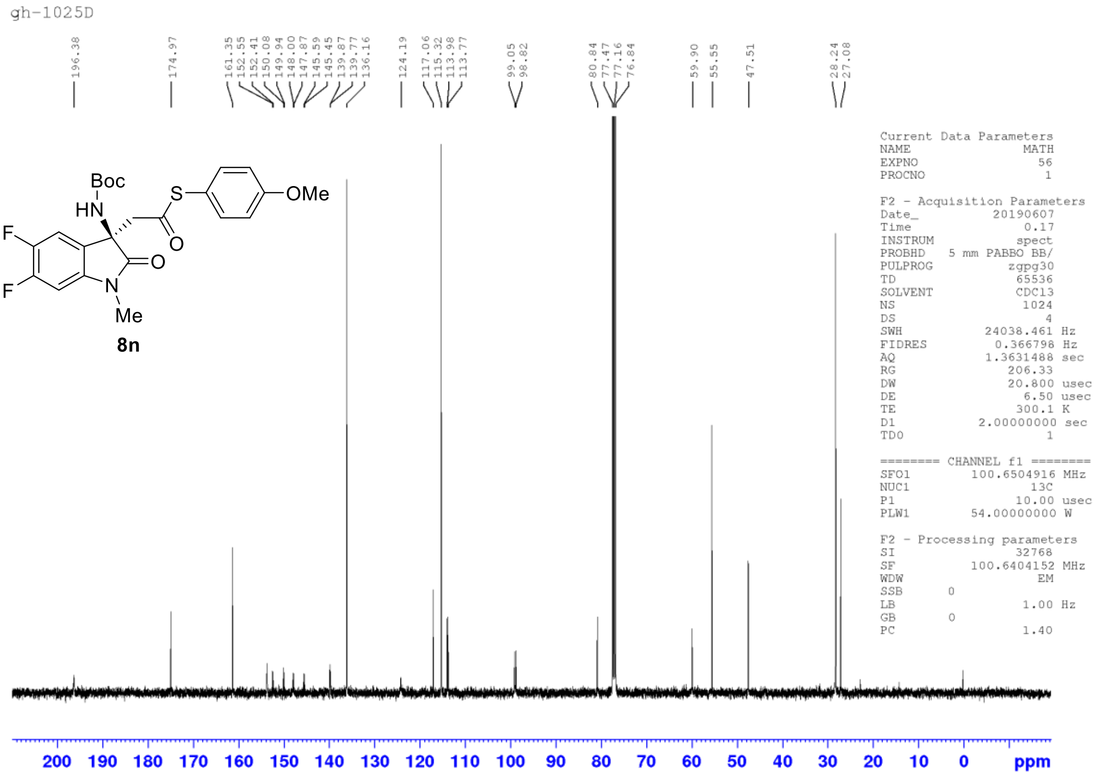

^{1}H and ^{13}C NMR of **8n** in $CDCl_3$.

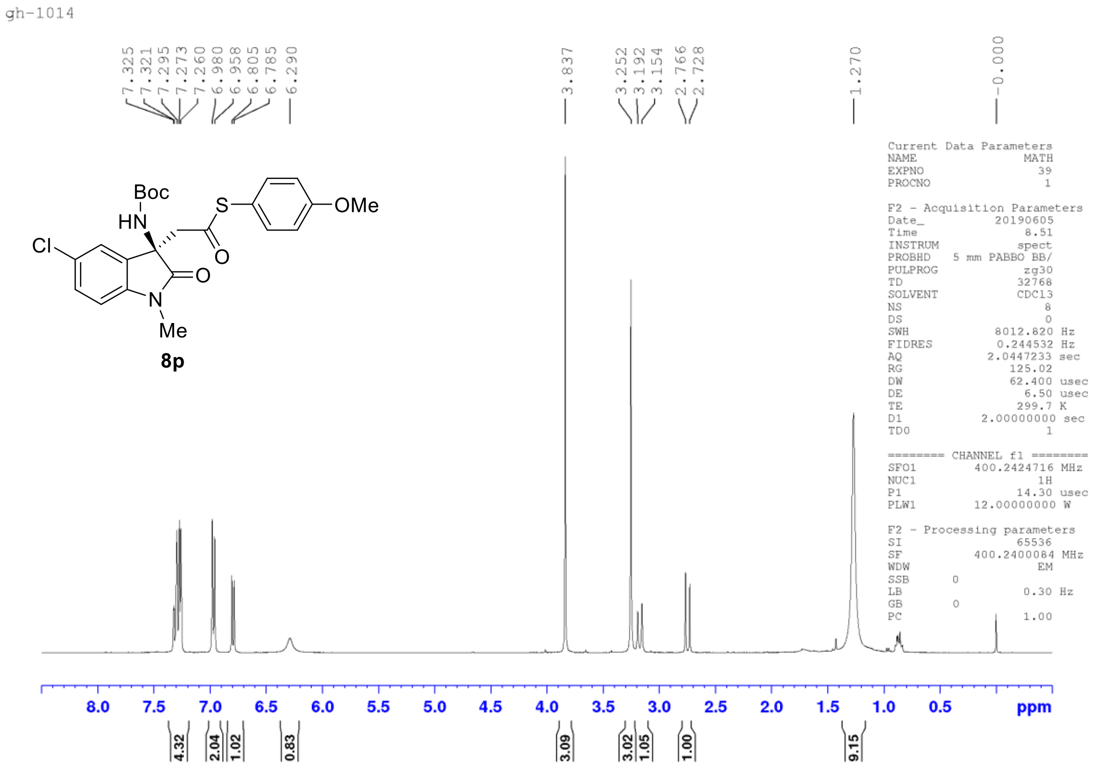

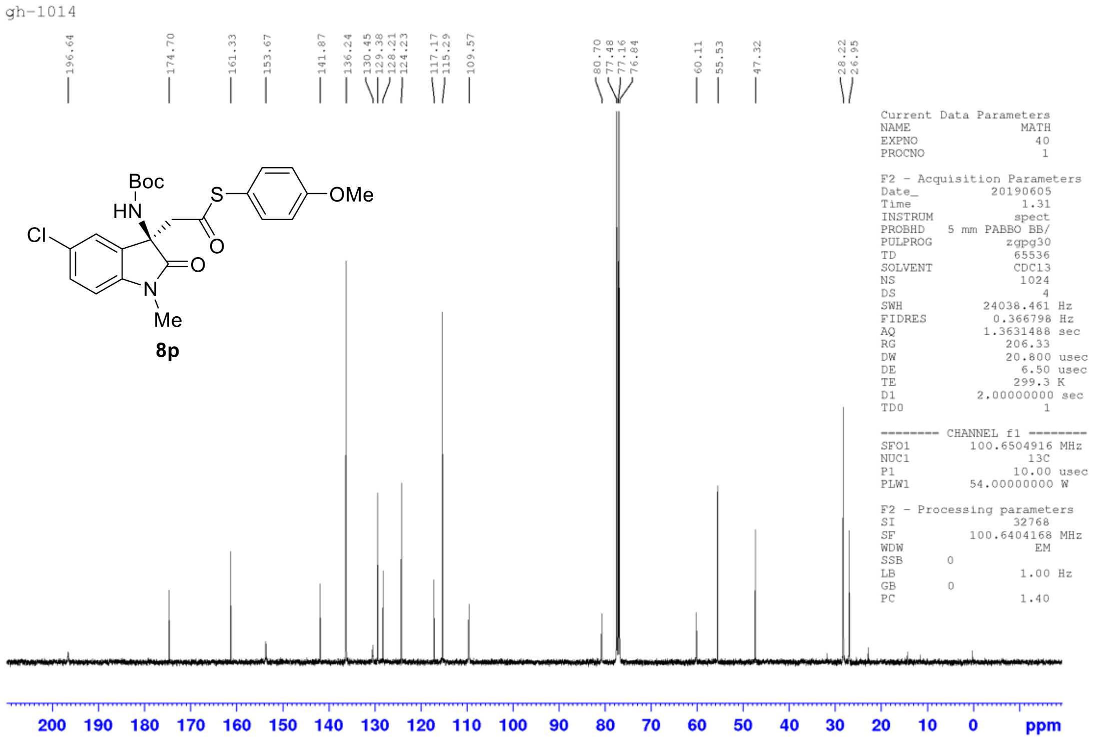

^{1}H and ^{13}C NMR of **8p** in $CDCl_3$.

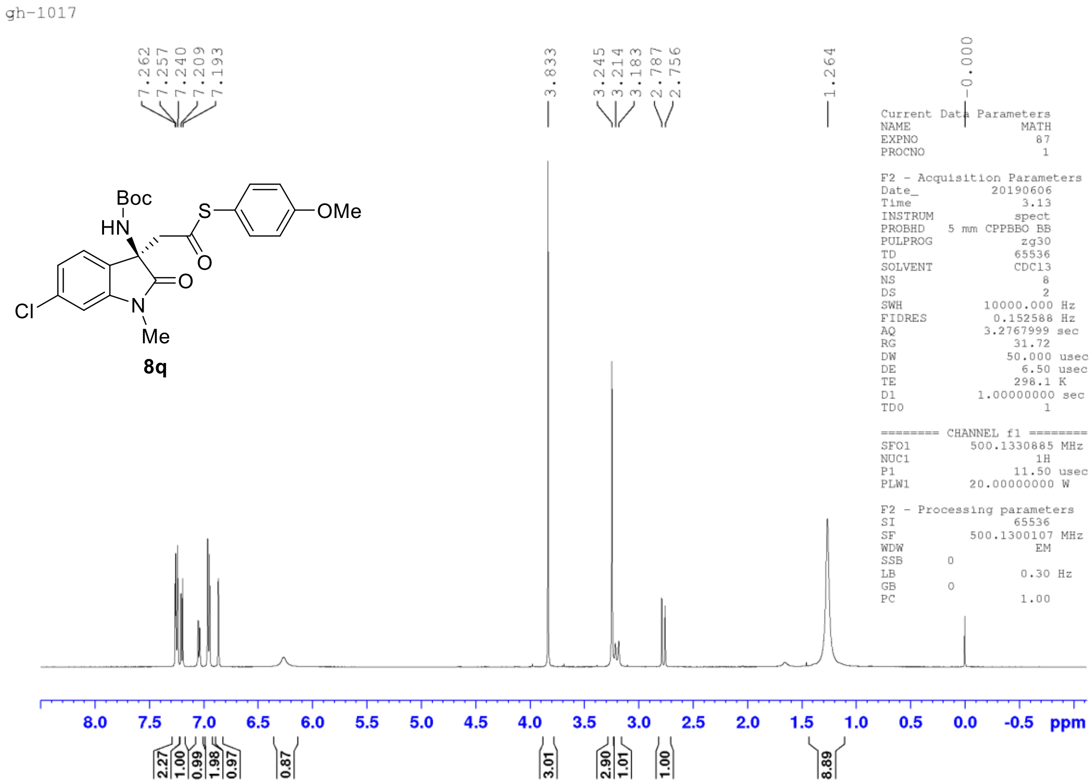

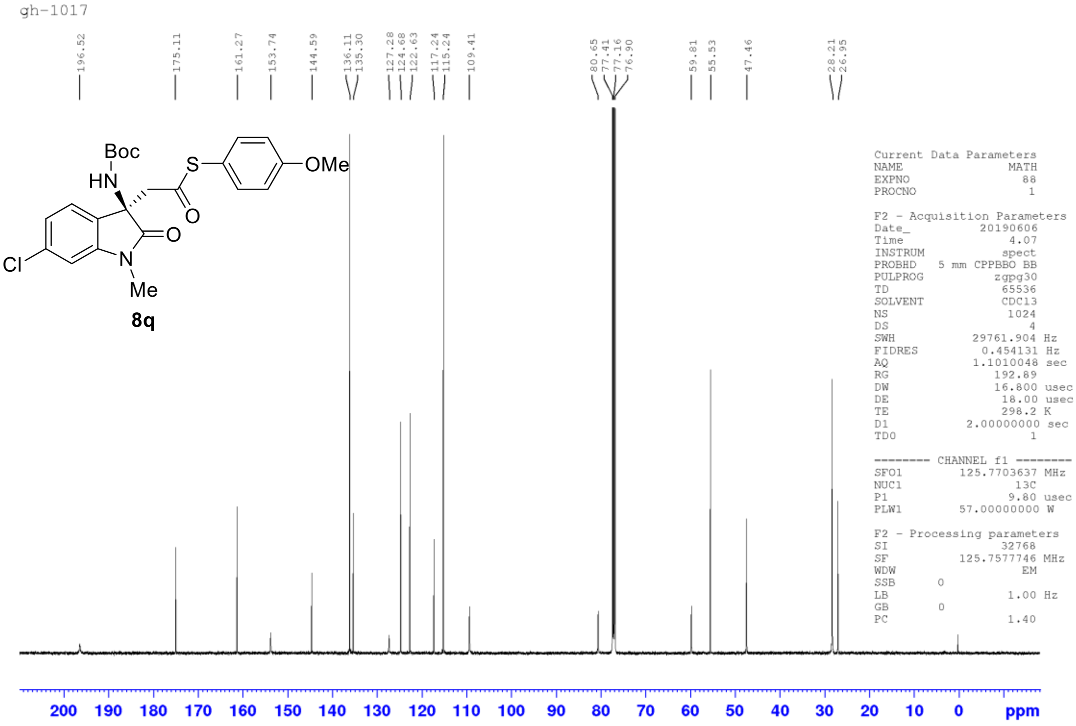

^{1}H and ^{13}C NMR of **8q** in $CDCl_3$.

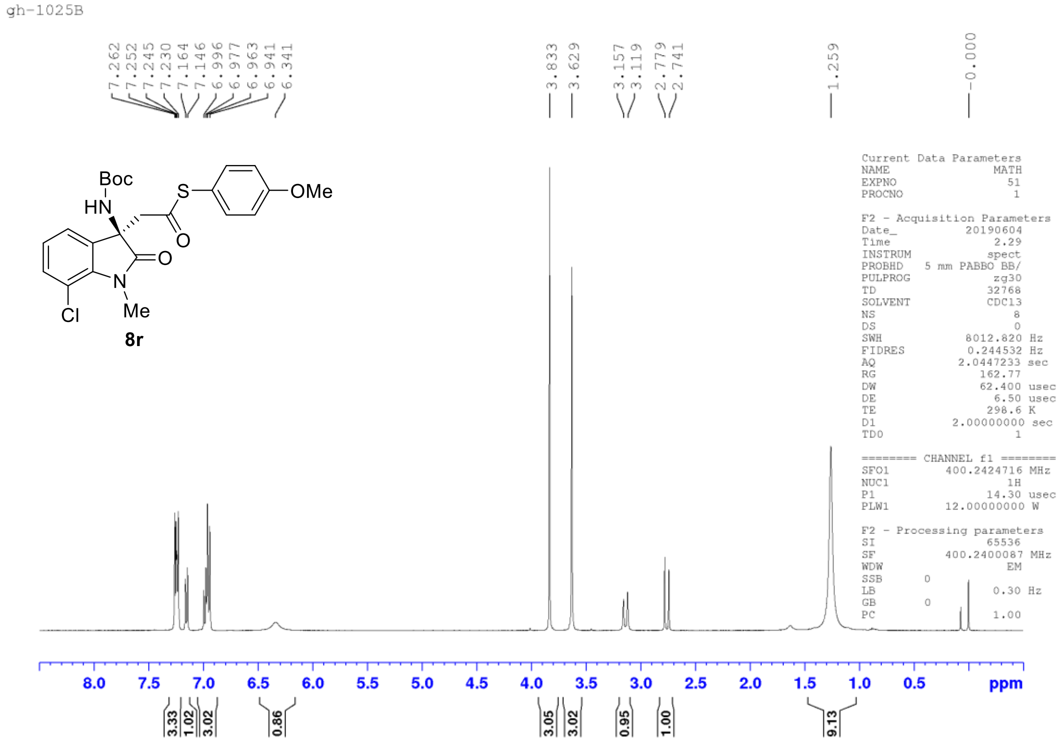

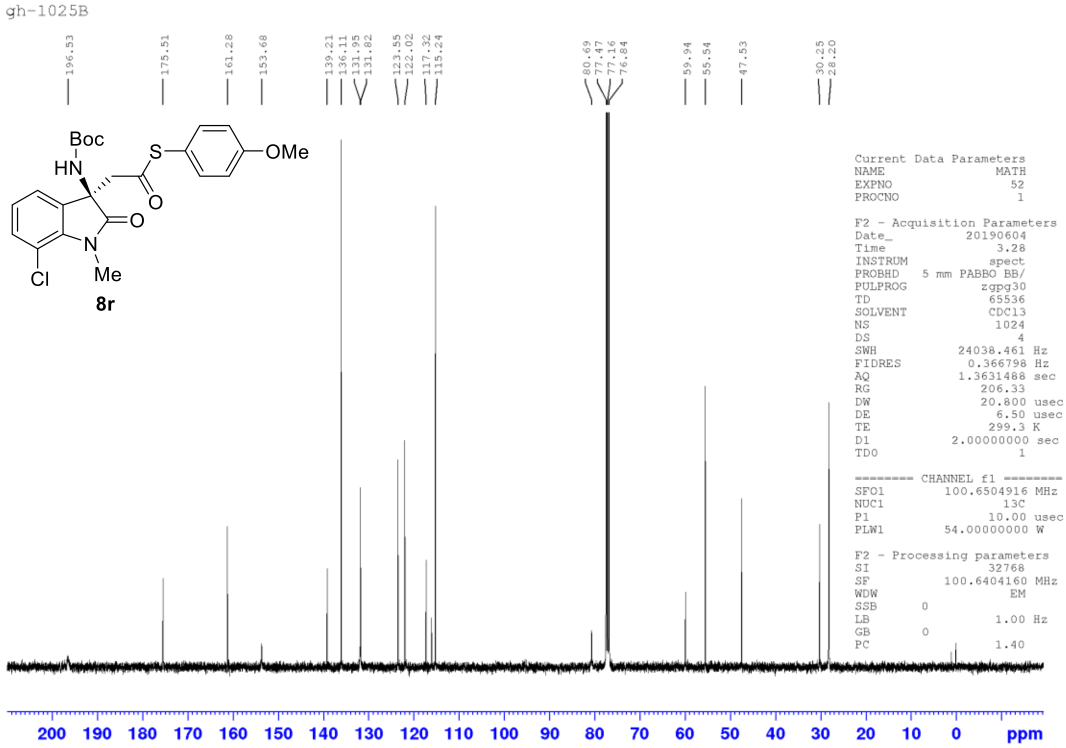

^{1}H and ^{13}C NMR of **8r** in $CDCl_3$.

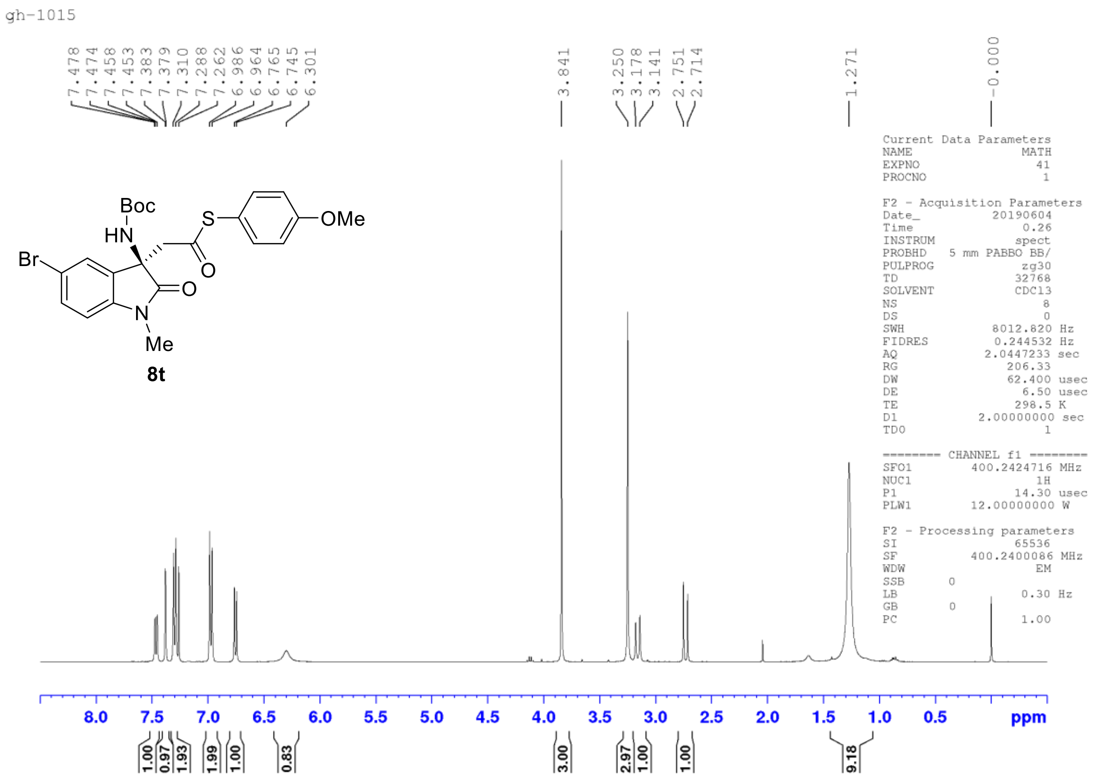

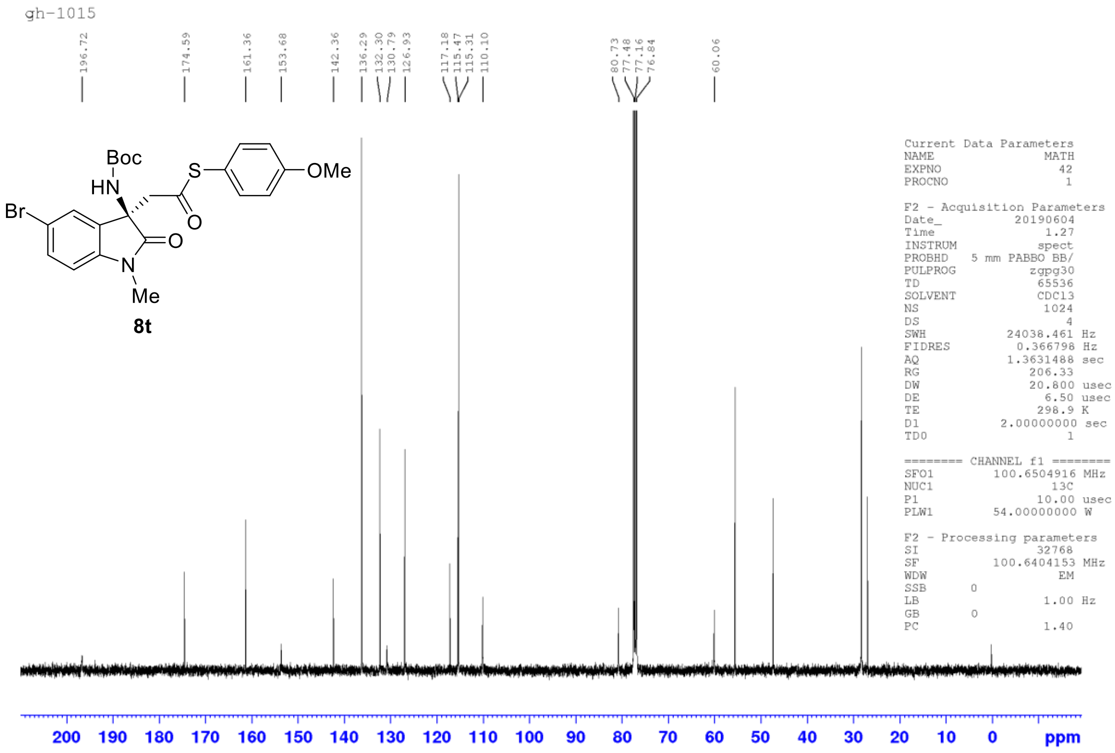

^{1}H and ^{13}C NMR of **8t** in $CDCl_3$.

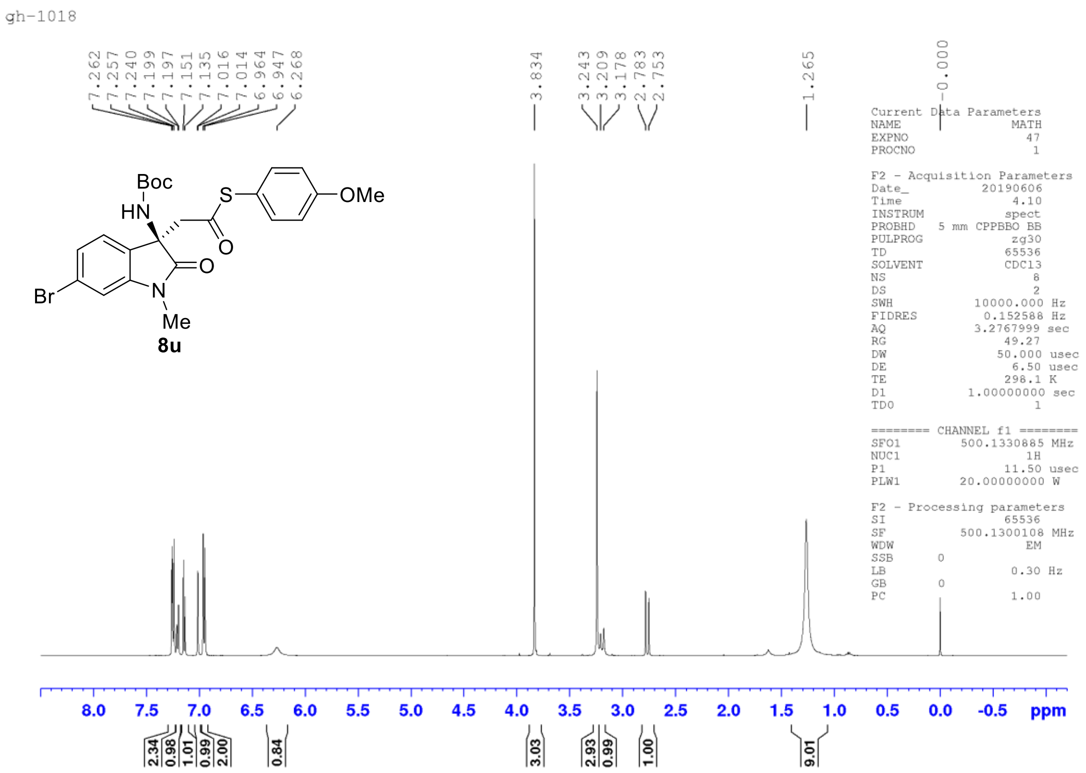

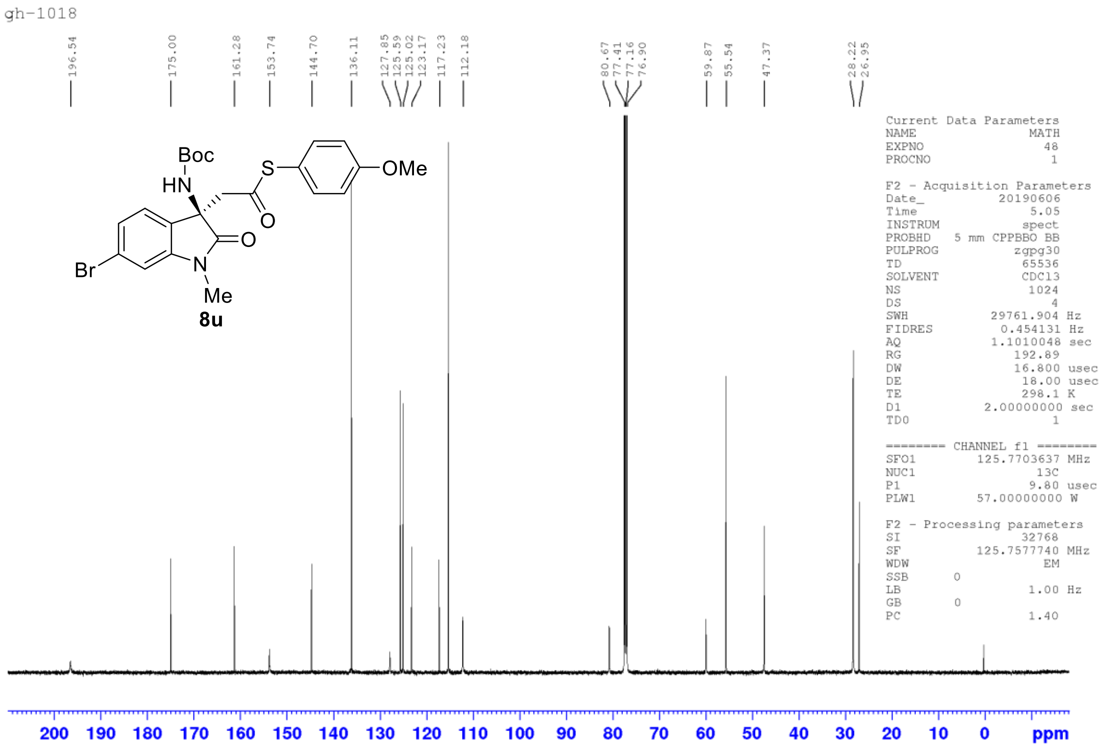

^{1}H and ^{13}C NMR of **8u** in $CDCl_3$.

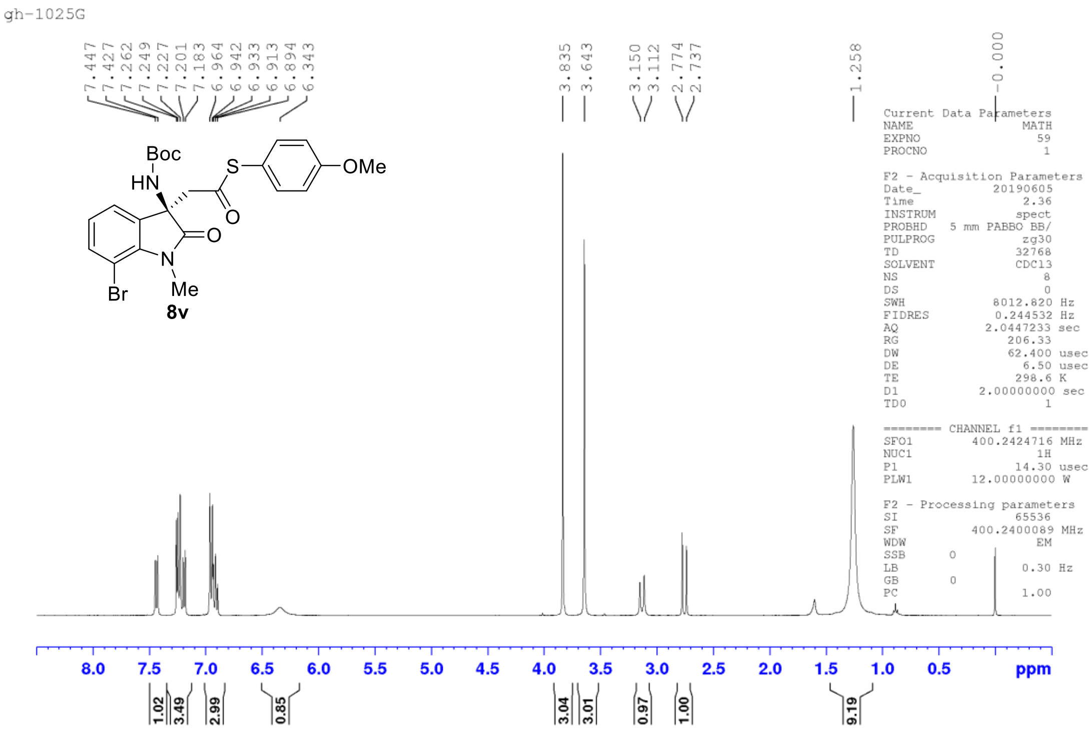

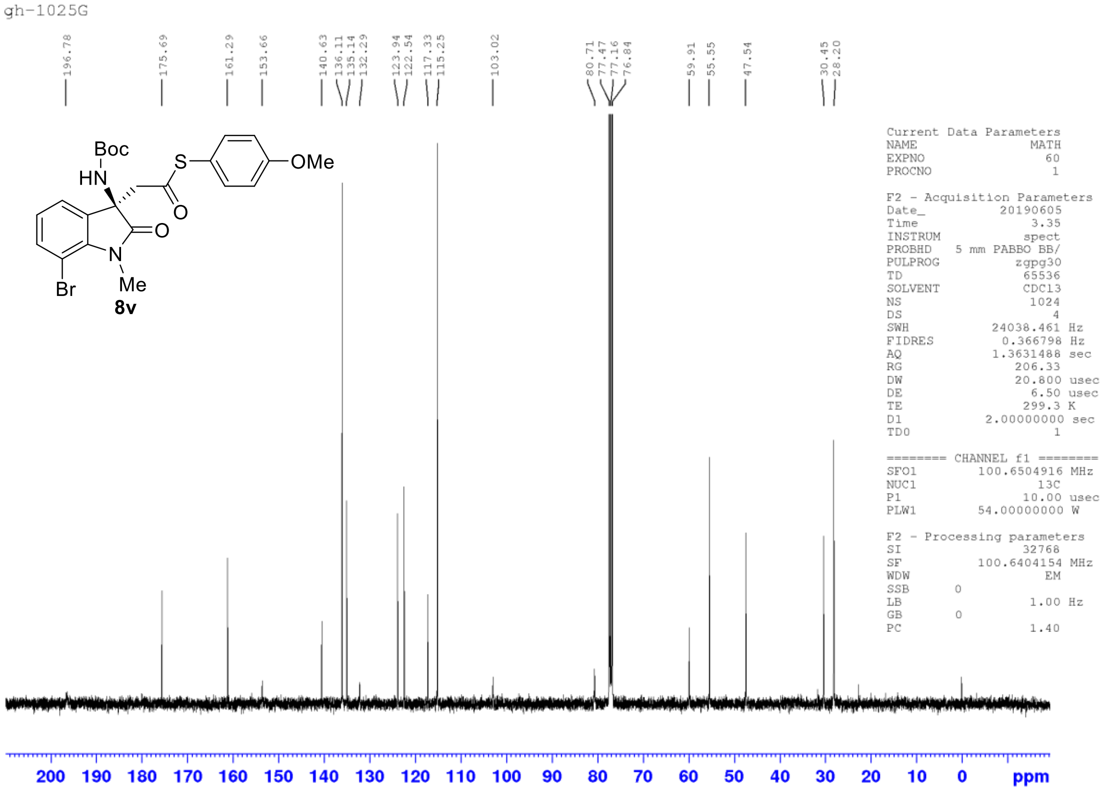

^{1}H and ^{13}C NMR of **8v** in $CDCl_3$.

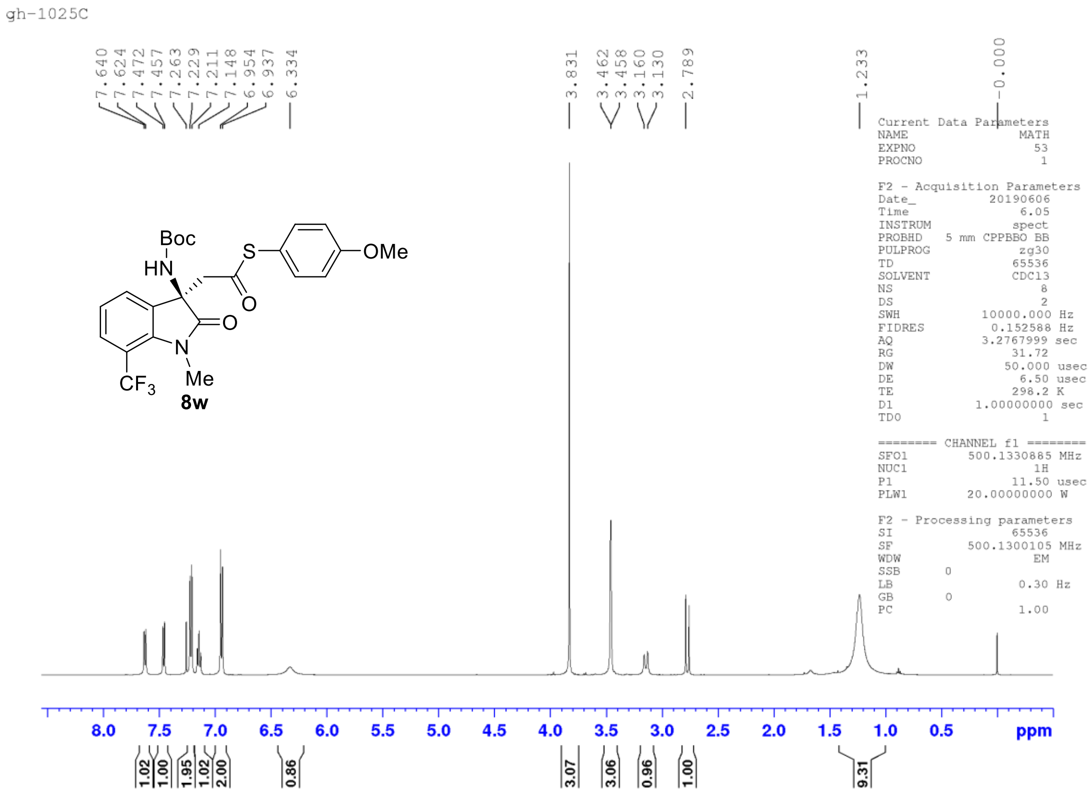

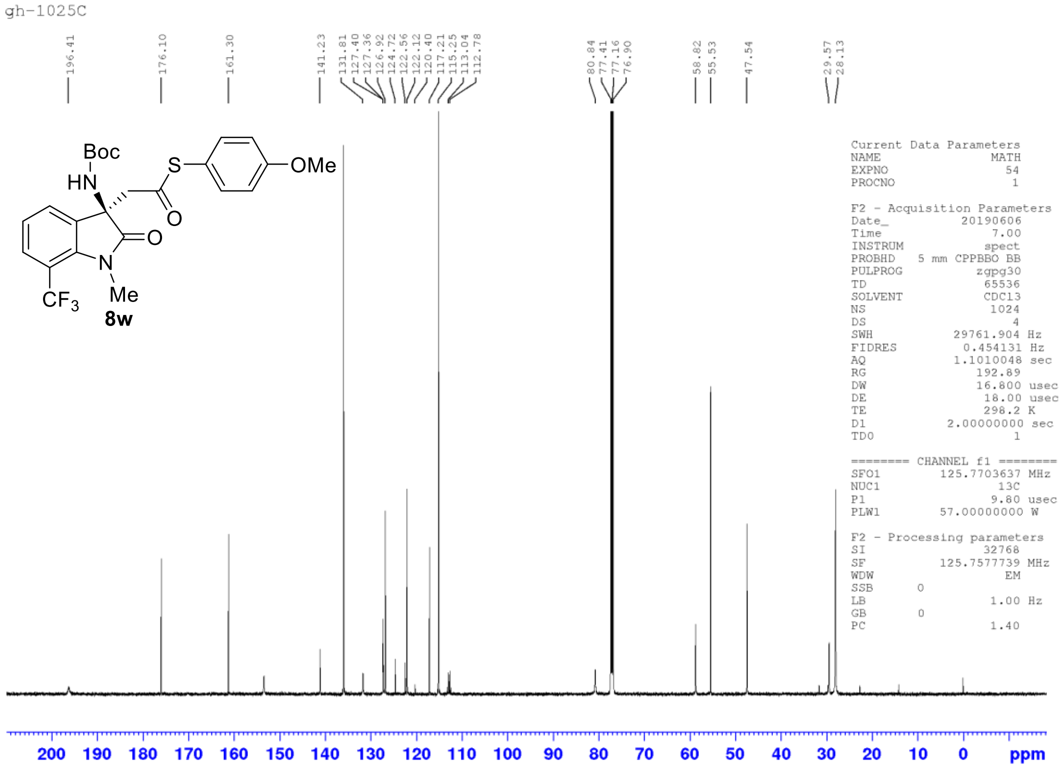

^{1}H and ^{13}C NMR of **8w** in $CDCl_3$.

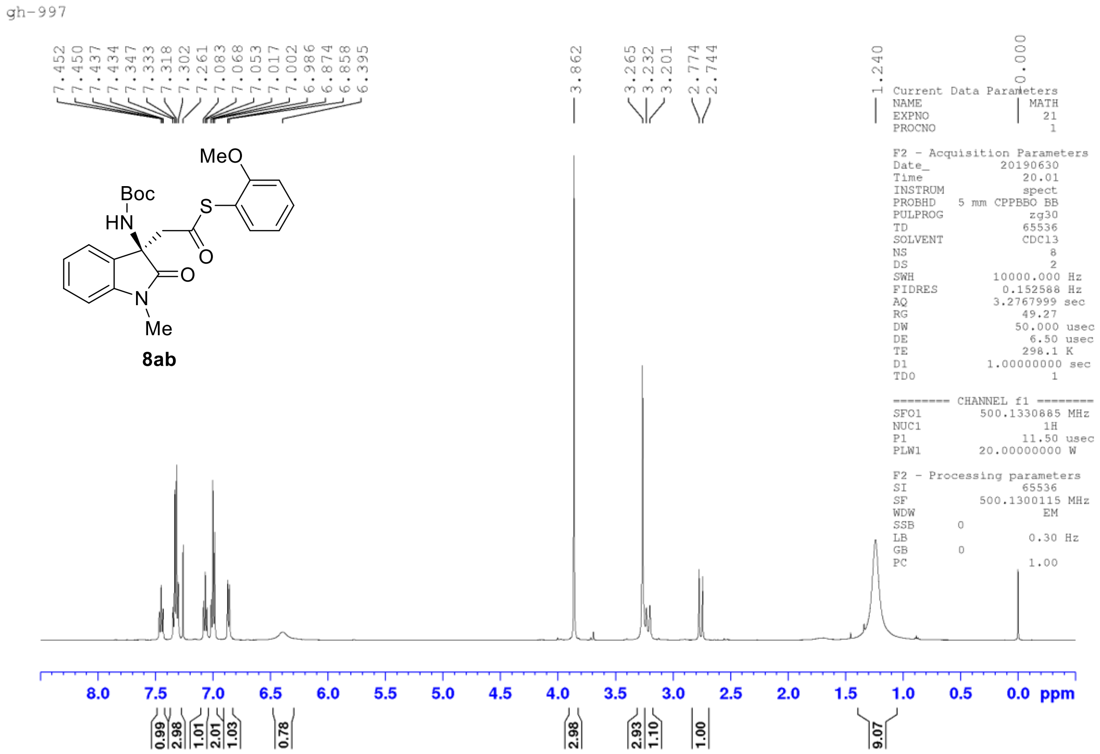

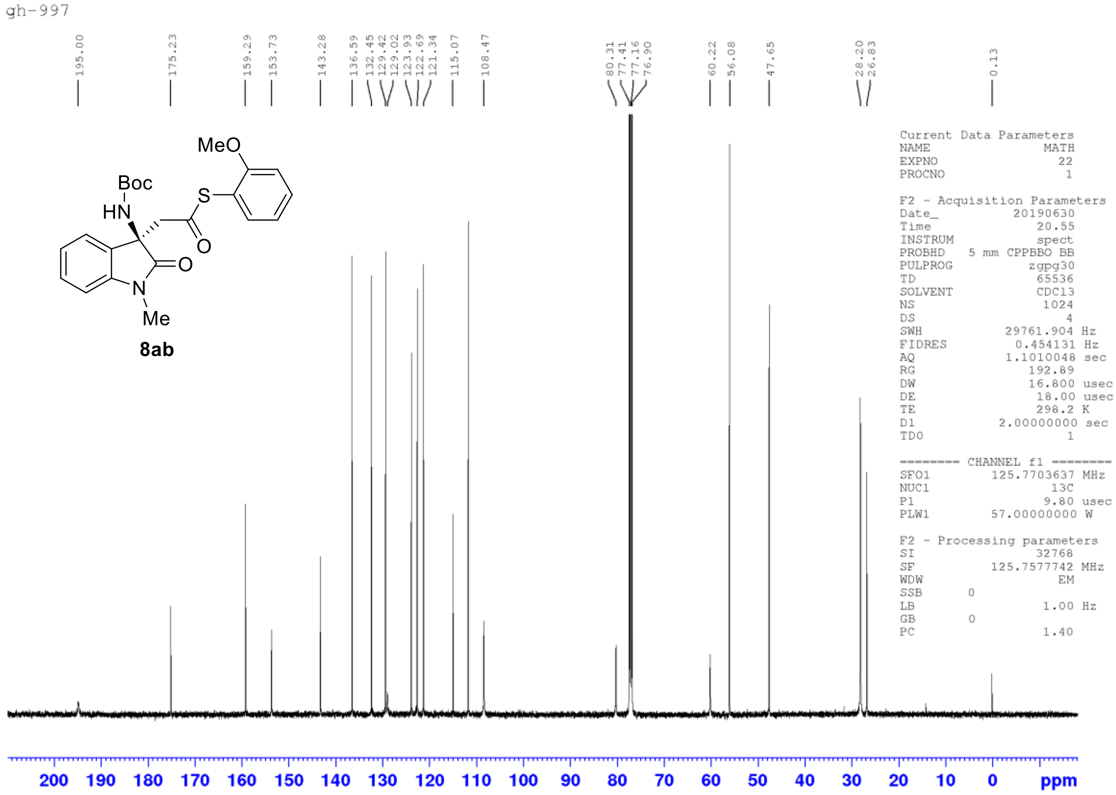

^1H and ^{13}C NMR of **8ab** in $CDCl_3$.

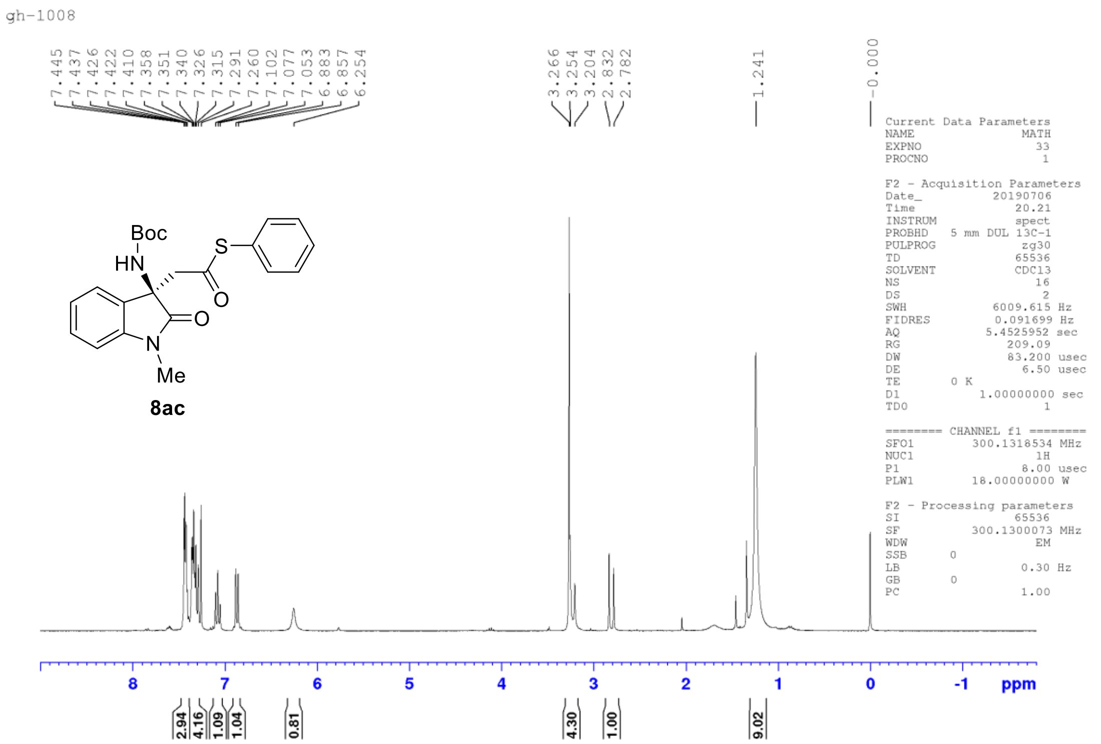

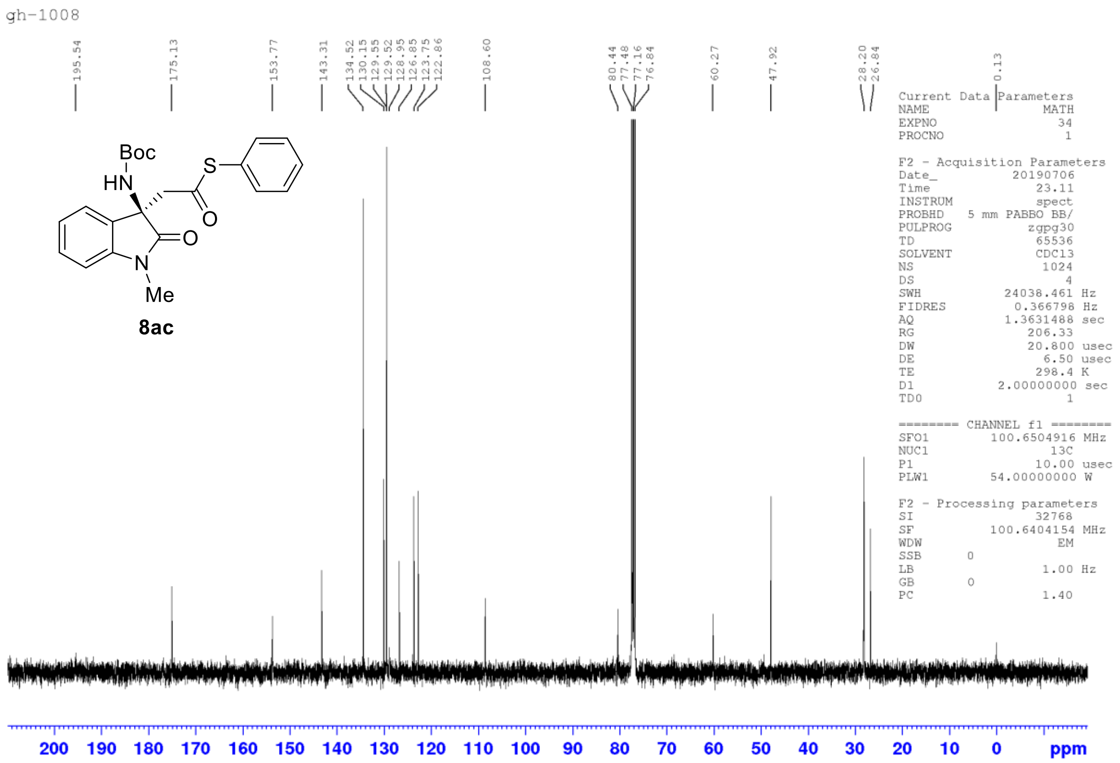

^{1}H and ^{13}C NMR of **8ac** in $CDCl_3$.

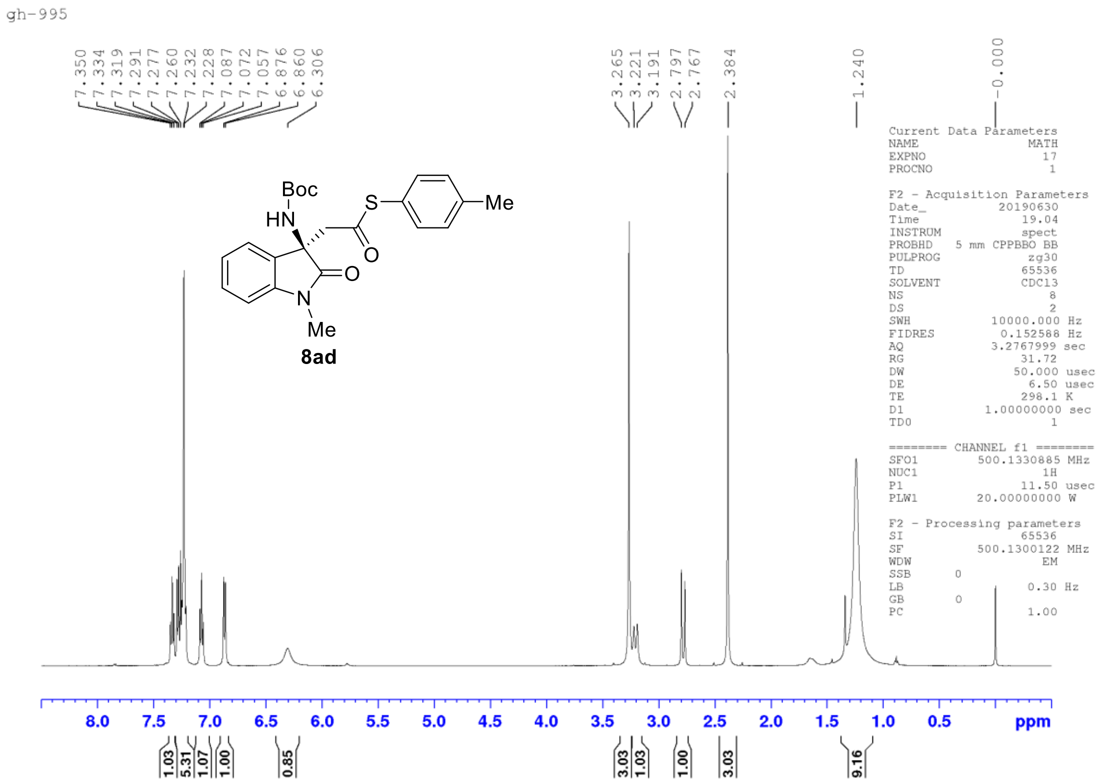

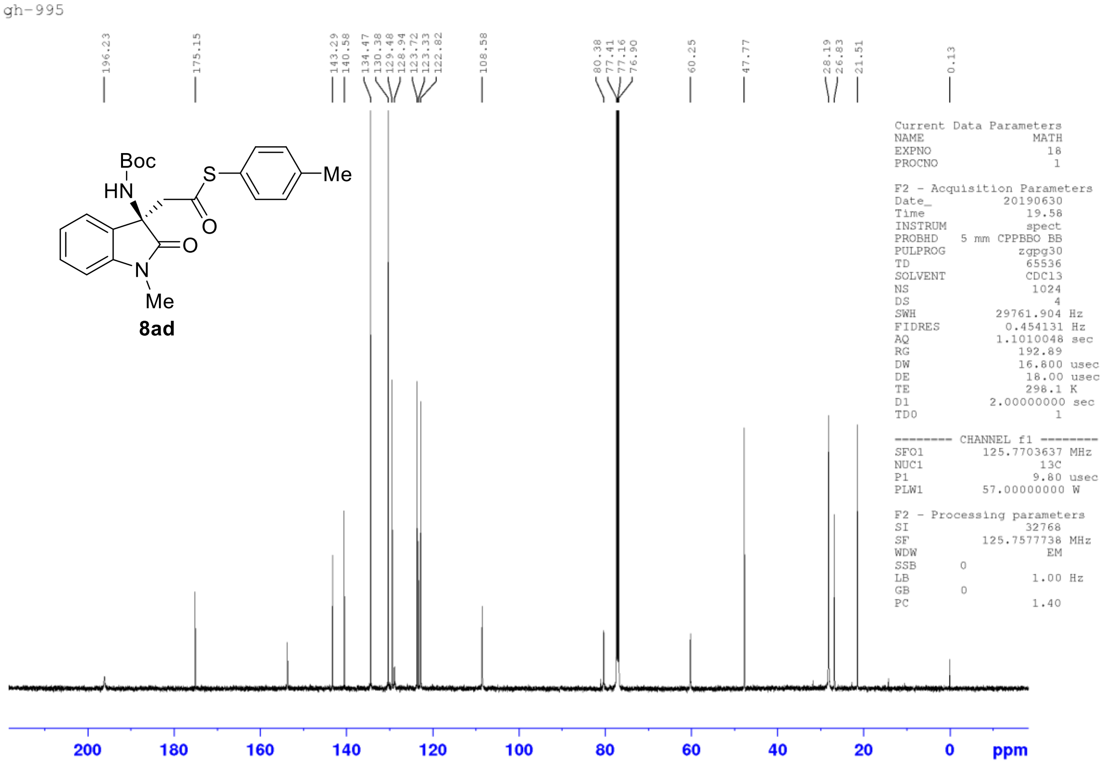

^{1}H and ^{13}C NMR of **8ad** in $CDCl_3$.

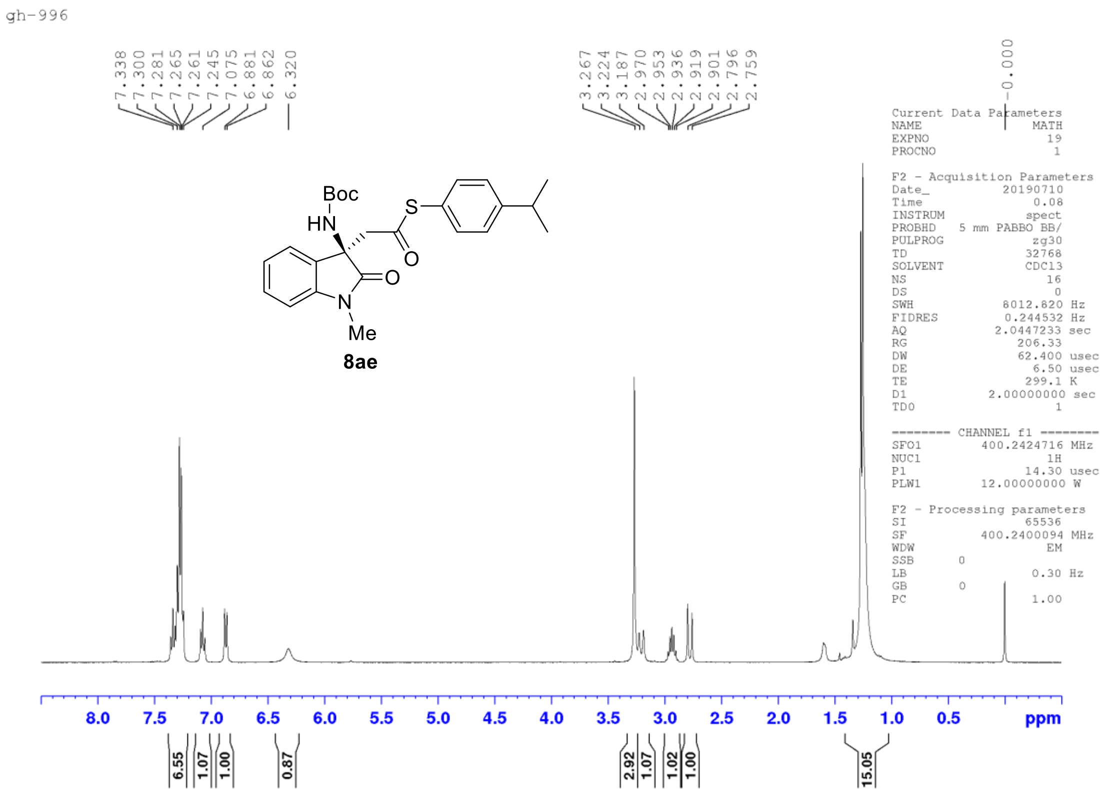

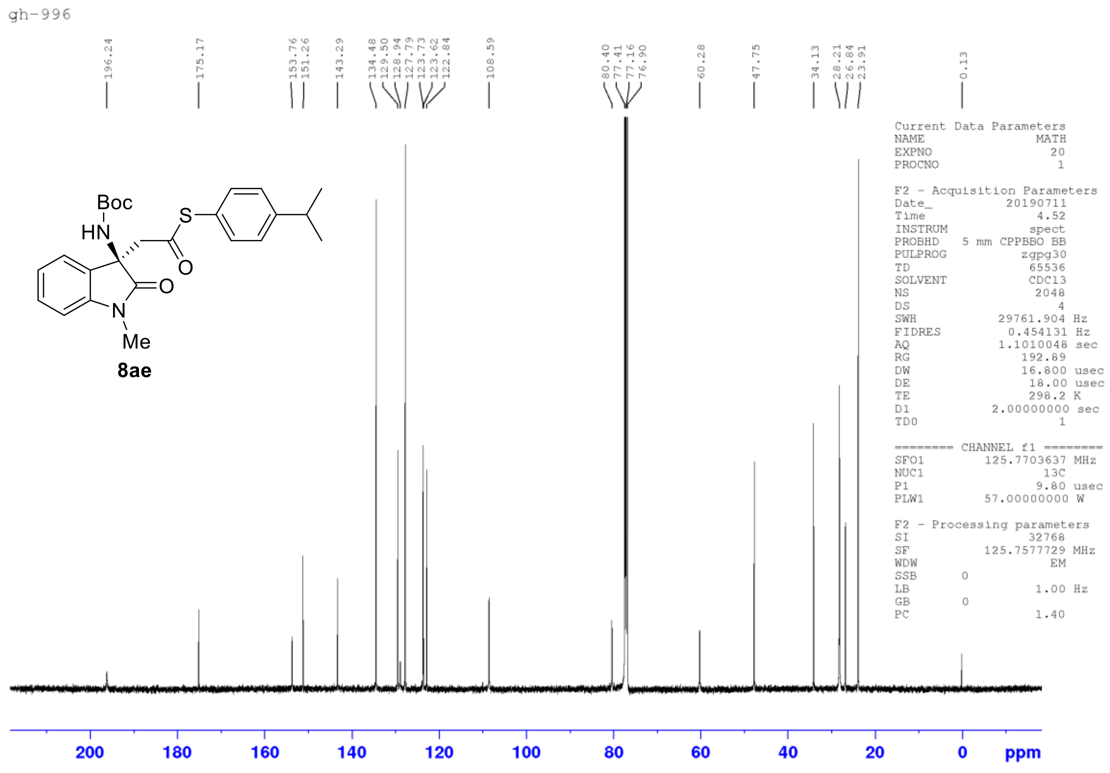

^{1}H and ^{13}C NMR of **8ae** in $CDCl_3$.

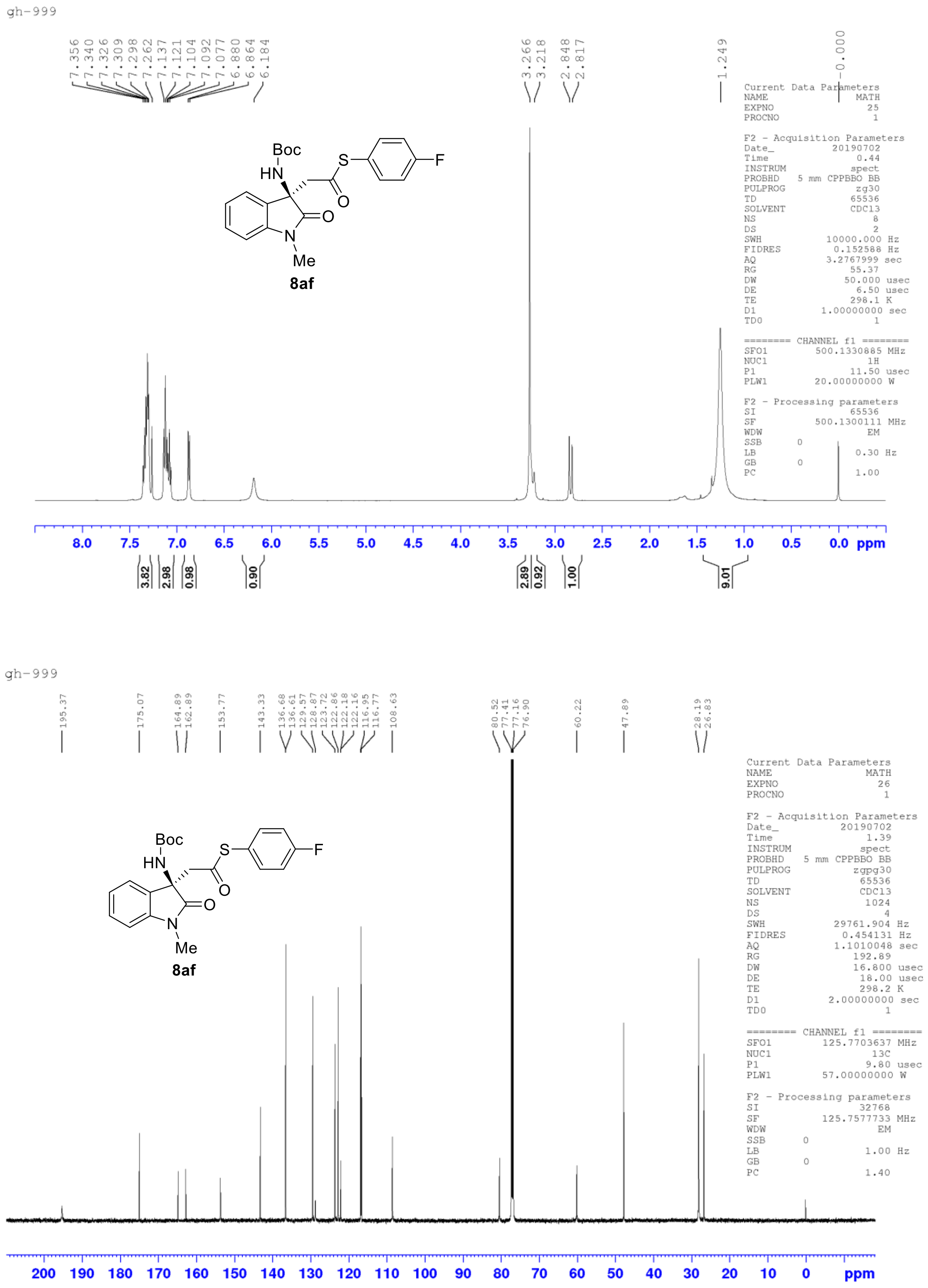

^1H and ^{13}C NMR of **8af** in $CDCl_3$.

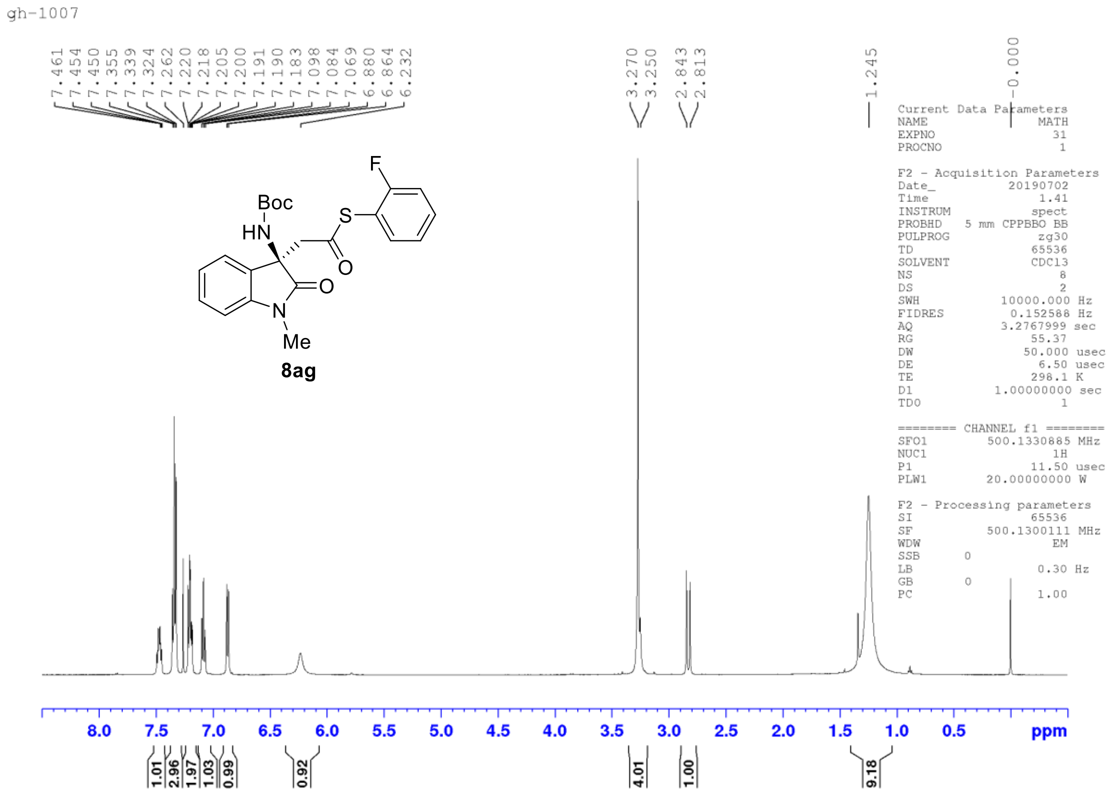

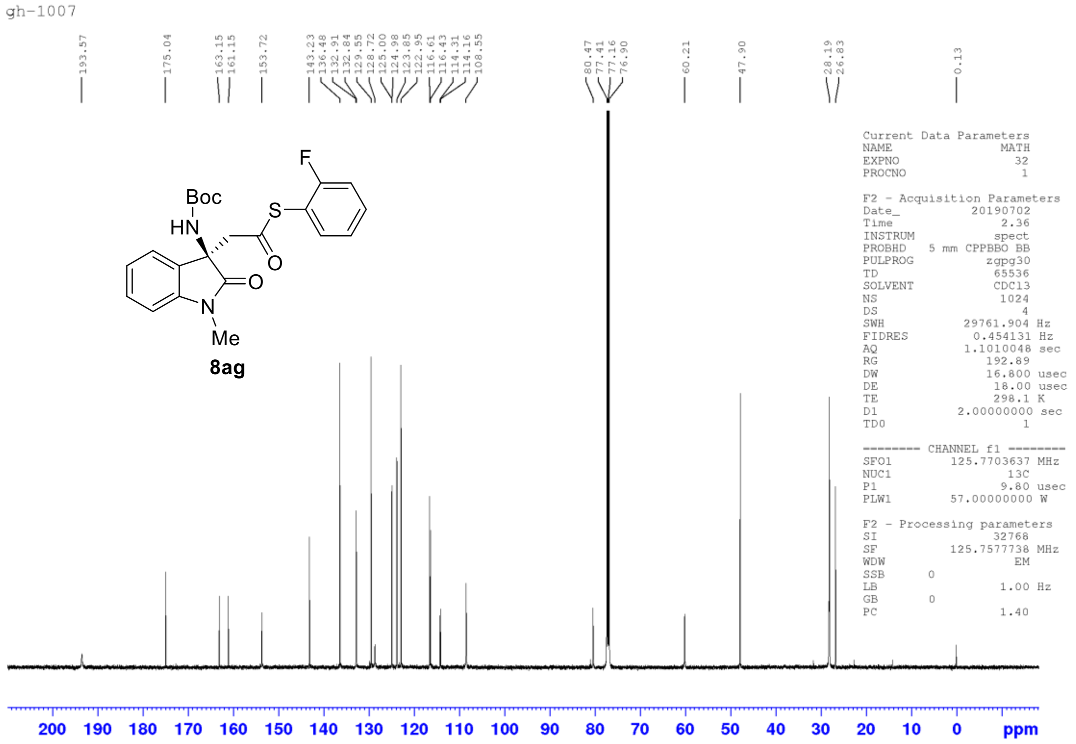

^{1}H and ^{13}C NMR of **8ag** in $CDCl_3$.

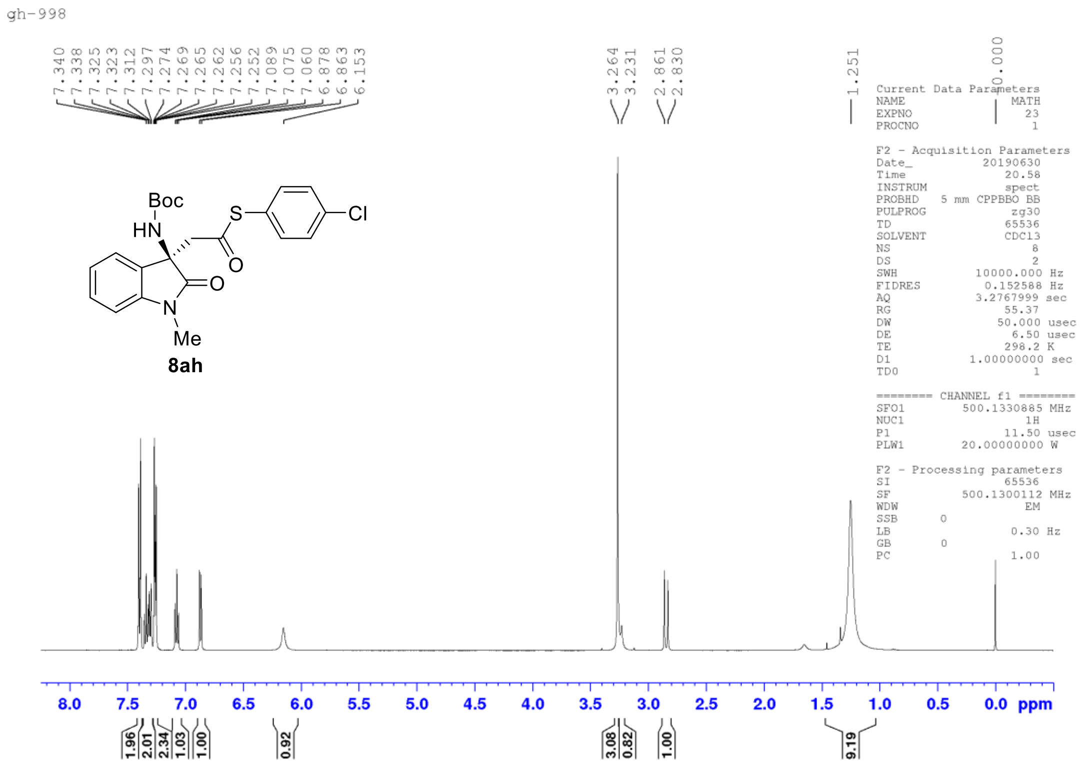

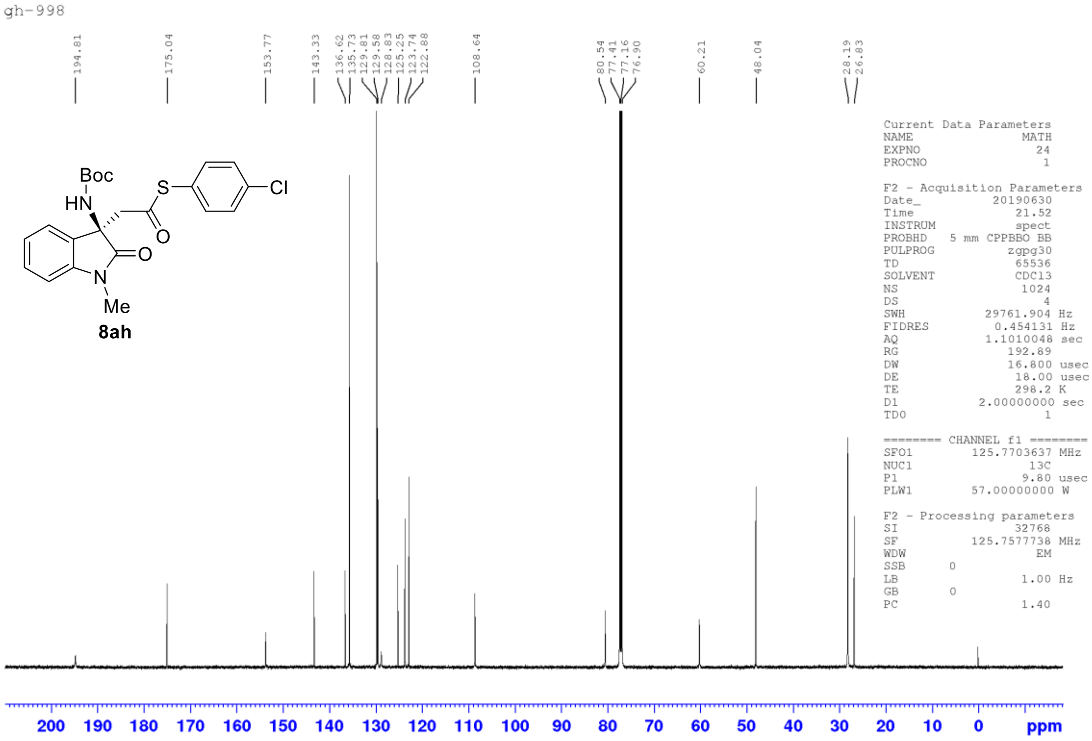

^{1}H and ^{13}C NMR of **8ah** in $CDCl_3$.

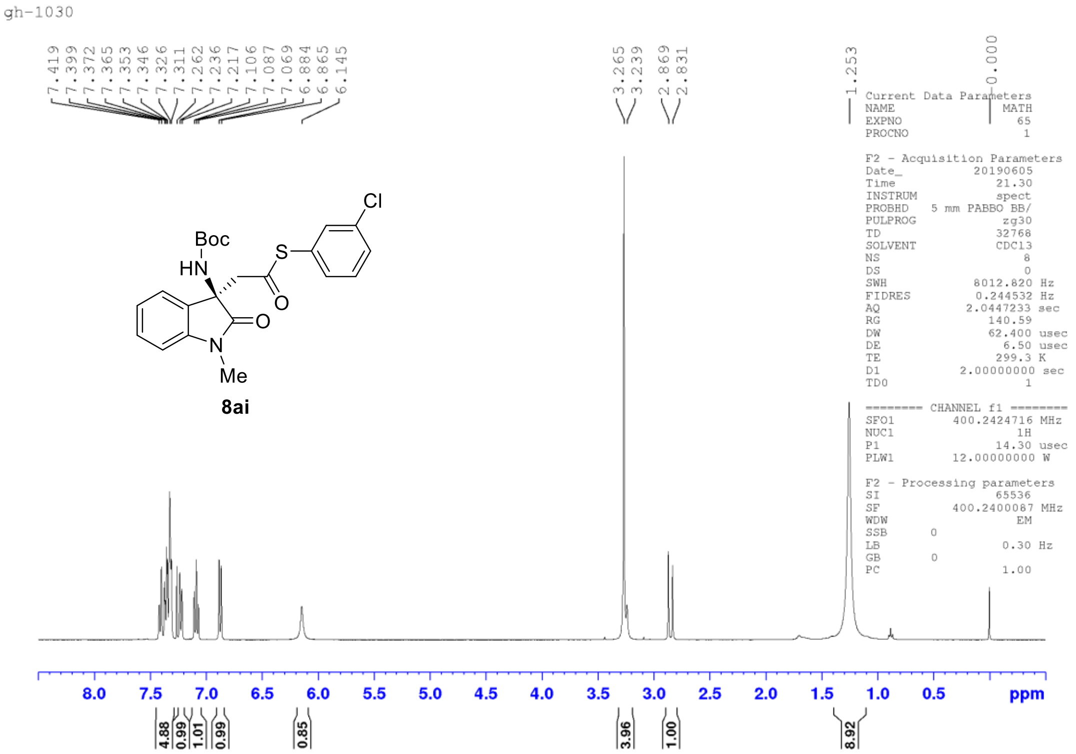

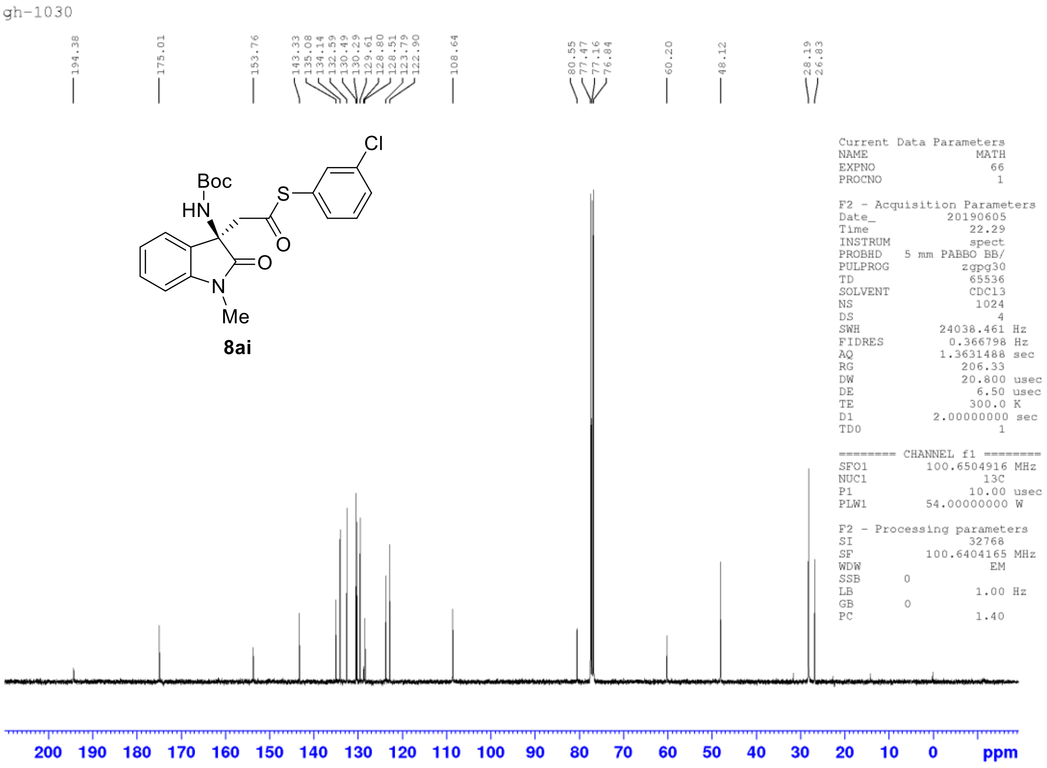

^{1}H and ^{13}C NMR of **8ai** in $CDCl_3$.

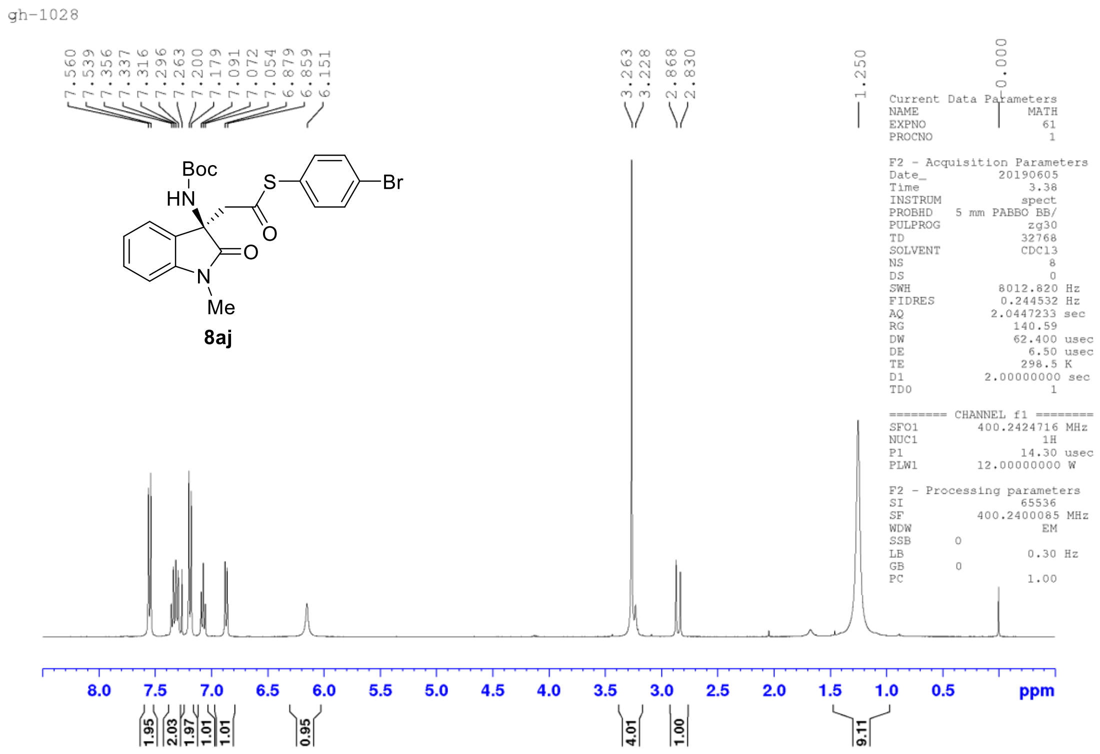

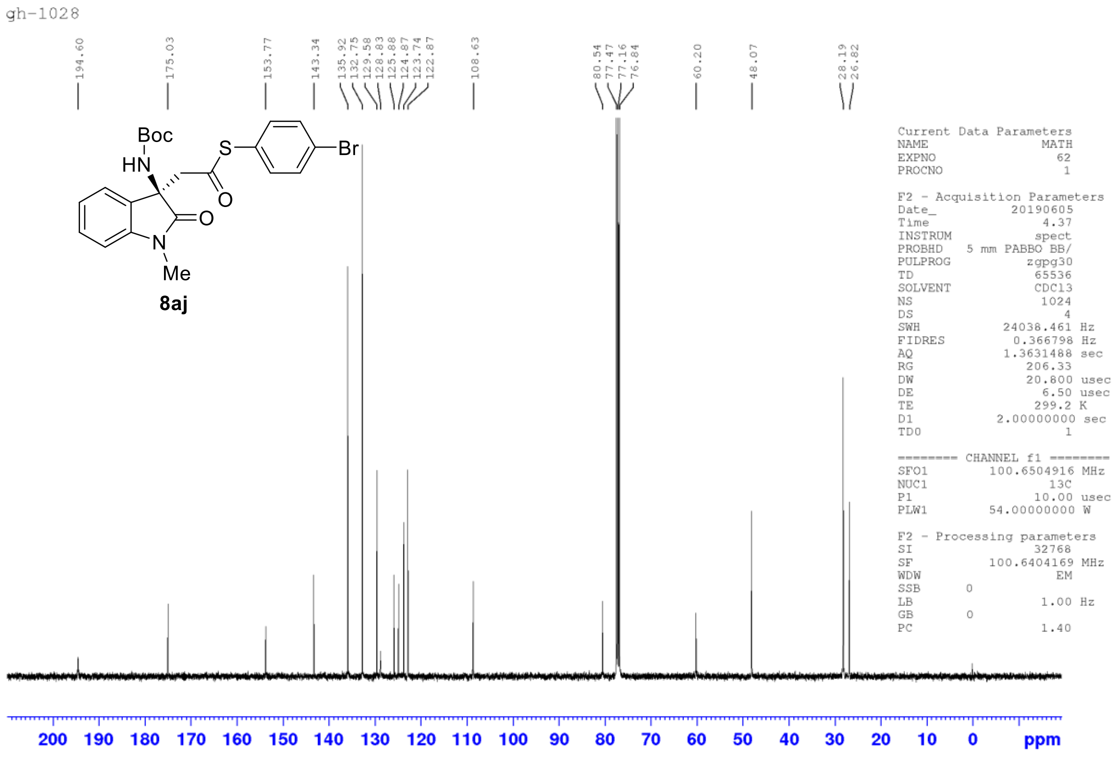

^{1}H and ^{13}C NMR of **8aj** in $CDCl_3$.

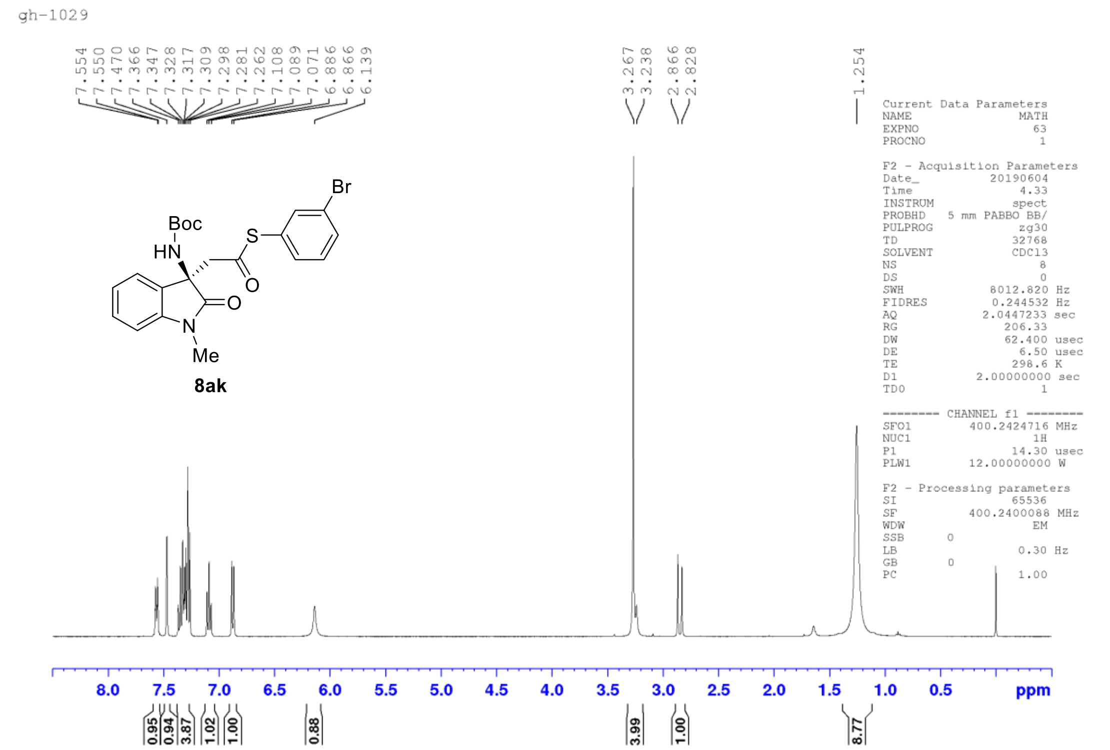

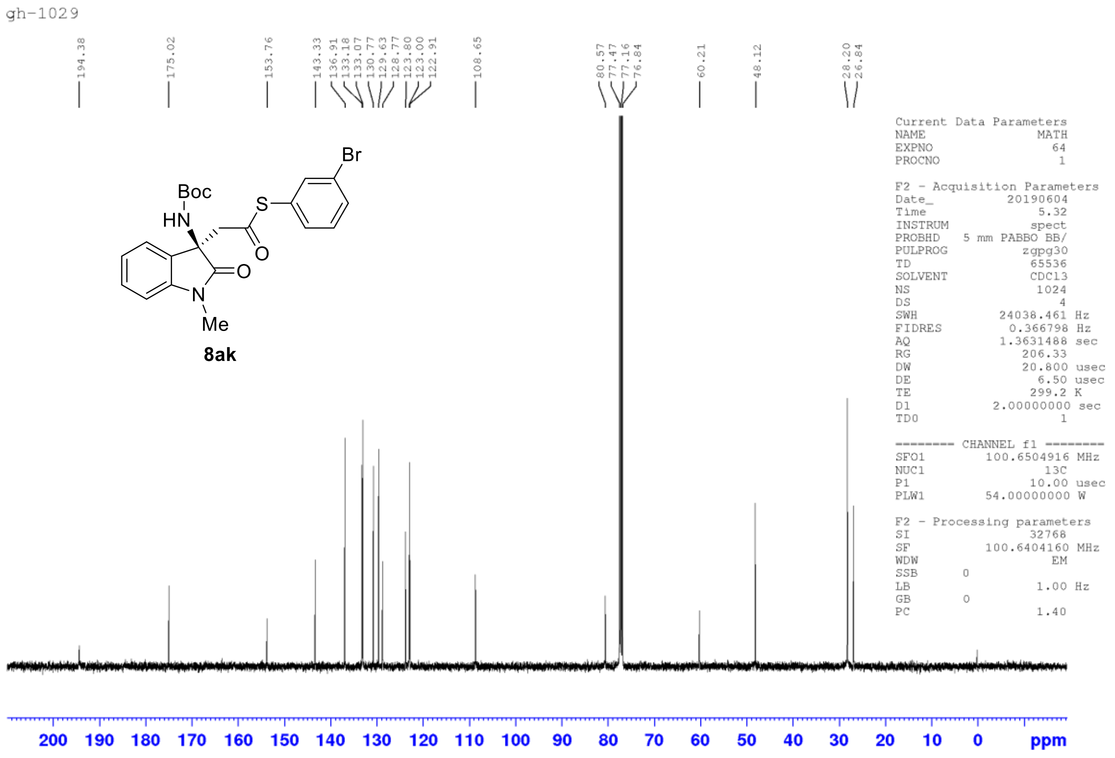

^{1}H and ^{13}C NMR of **8ak** in $CDCl_3$.

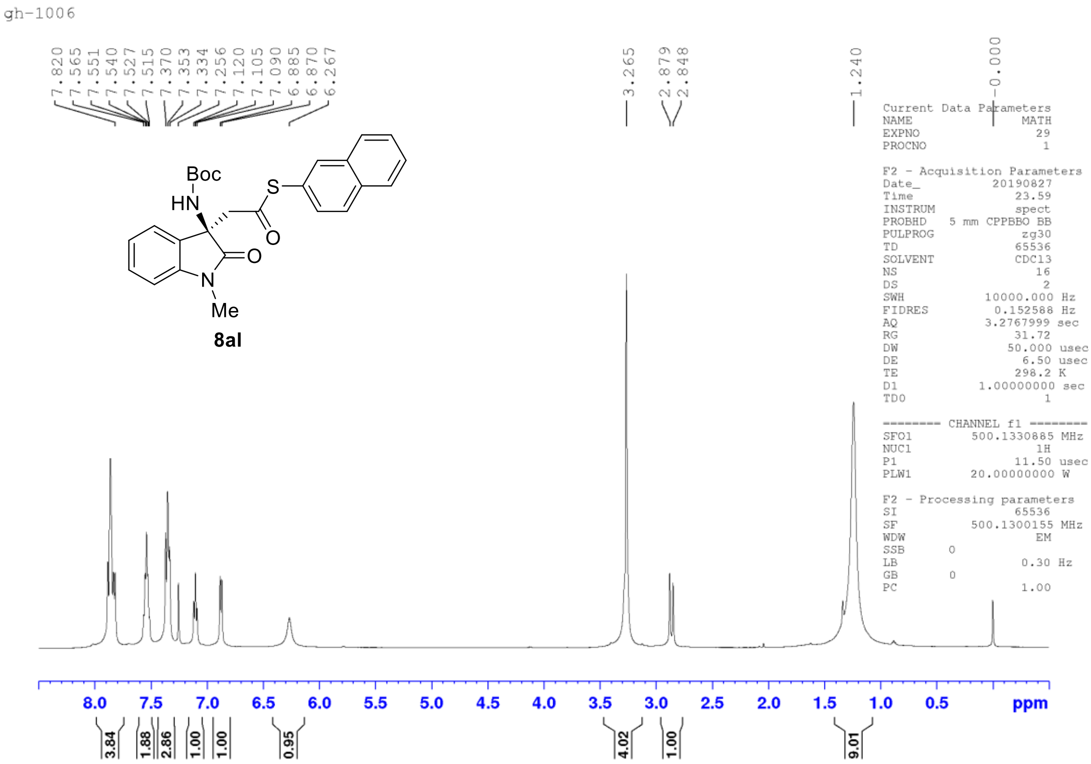

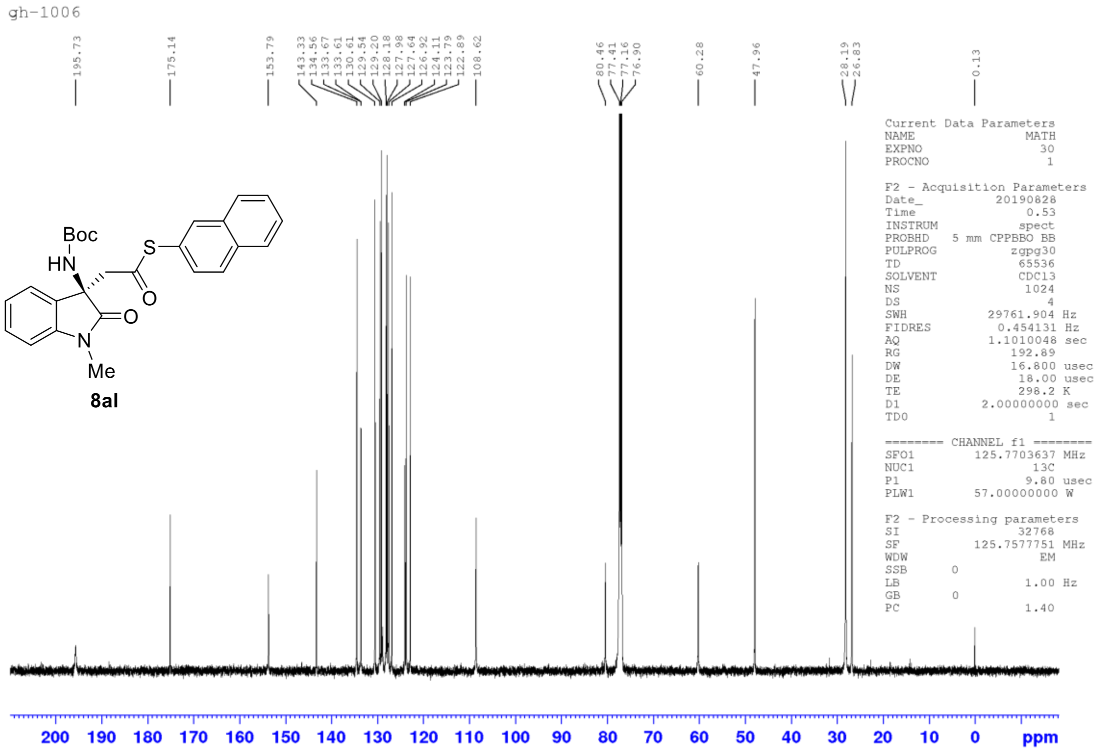

^{1}H and ^{13}C NMR of **8al** in $CDCl_3$.

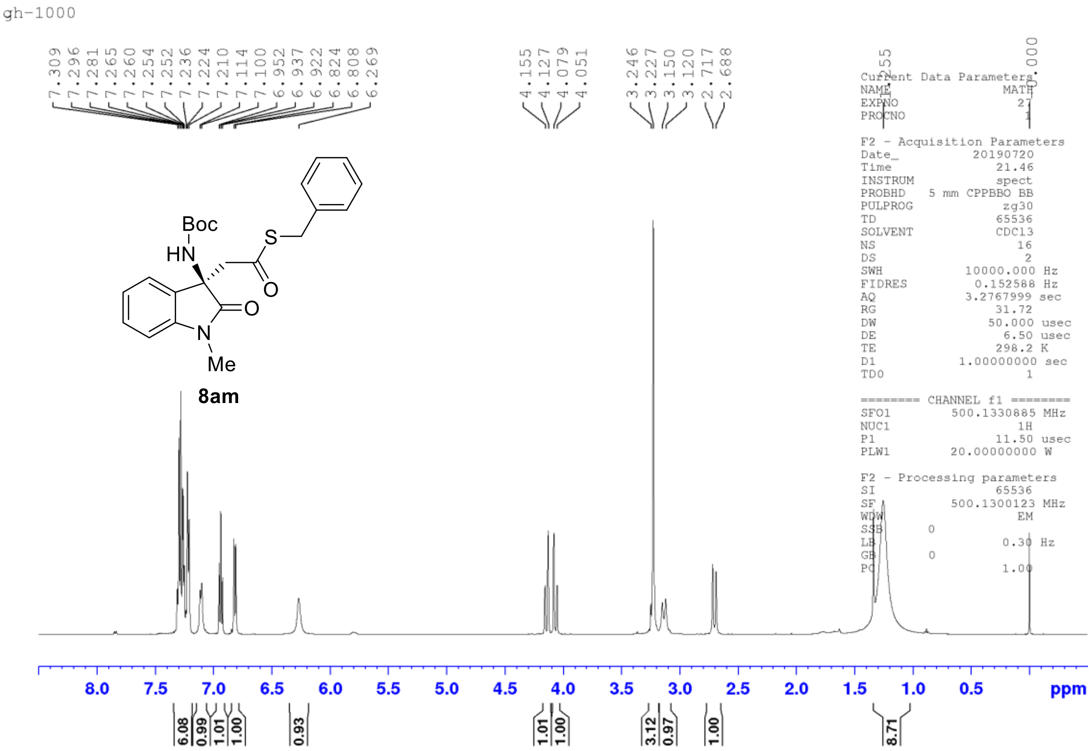

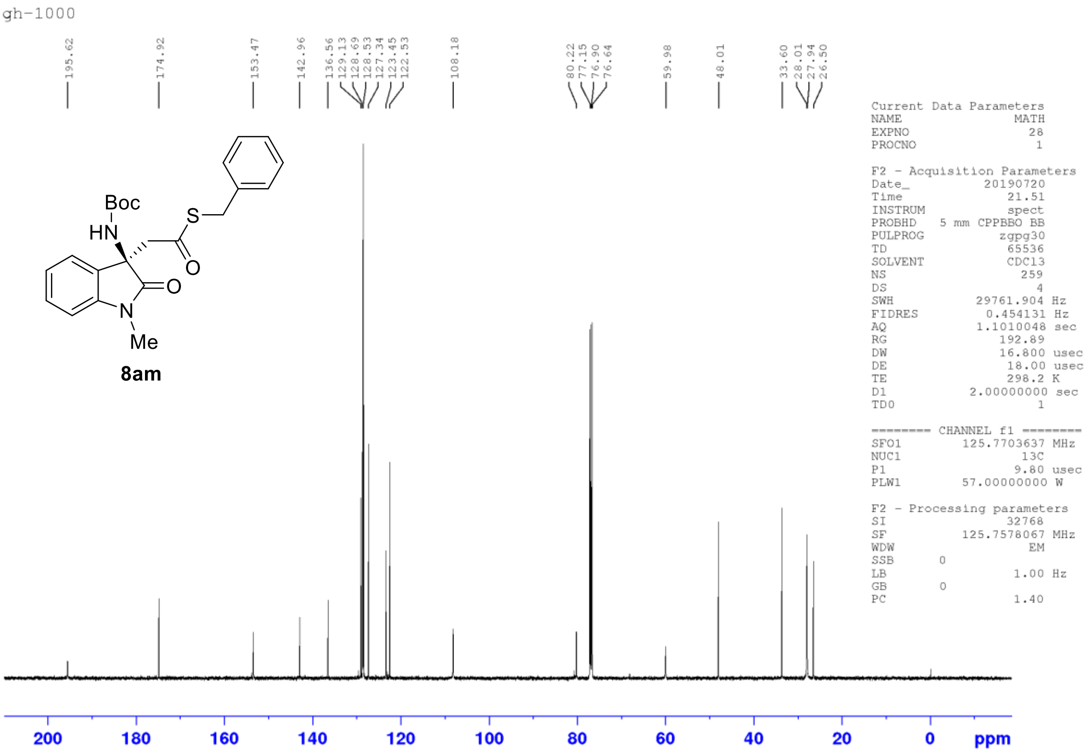

^{1}H and ^{13}C NMR of **8am** in $CDCl_3$.

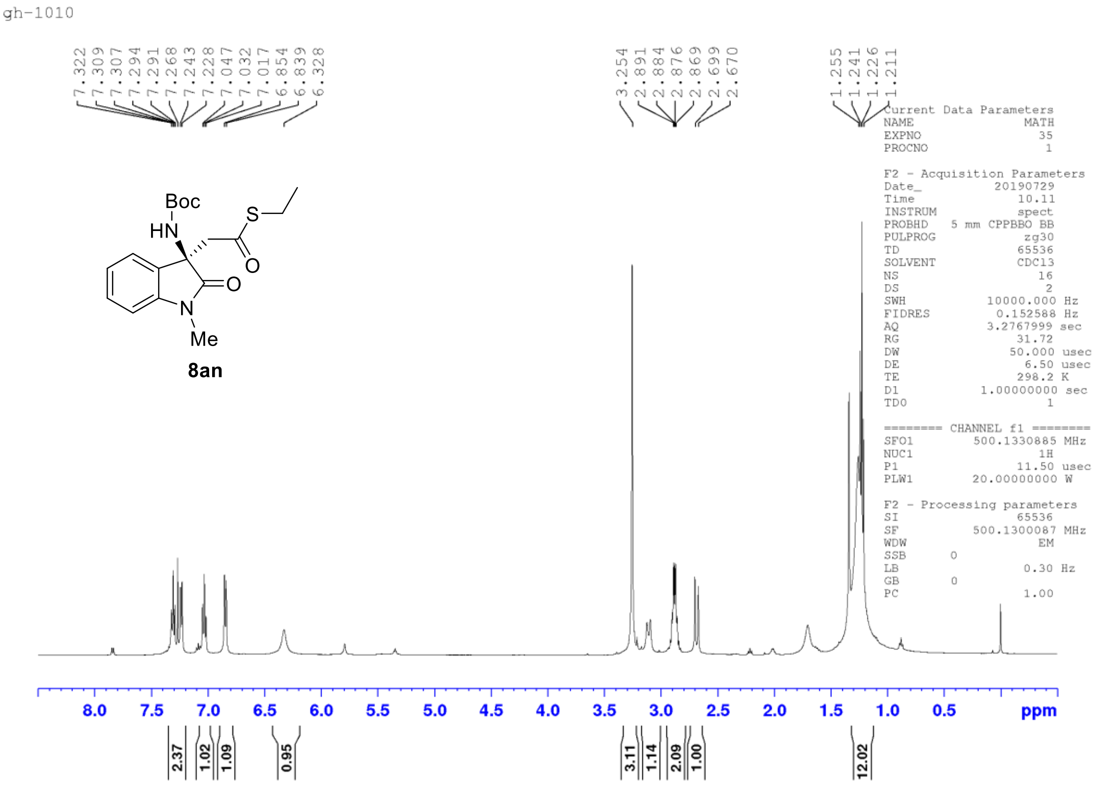

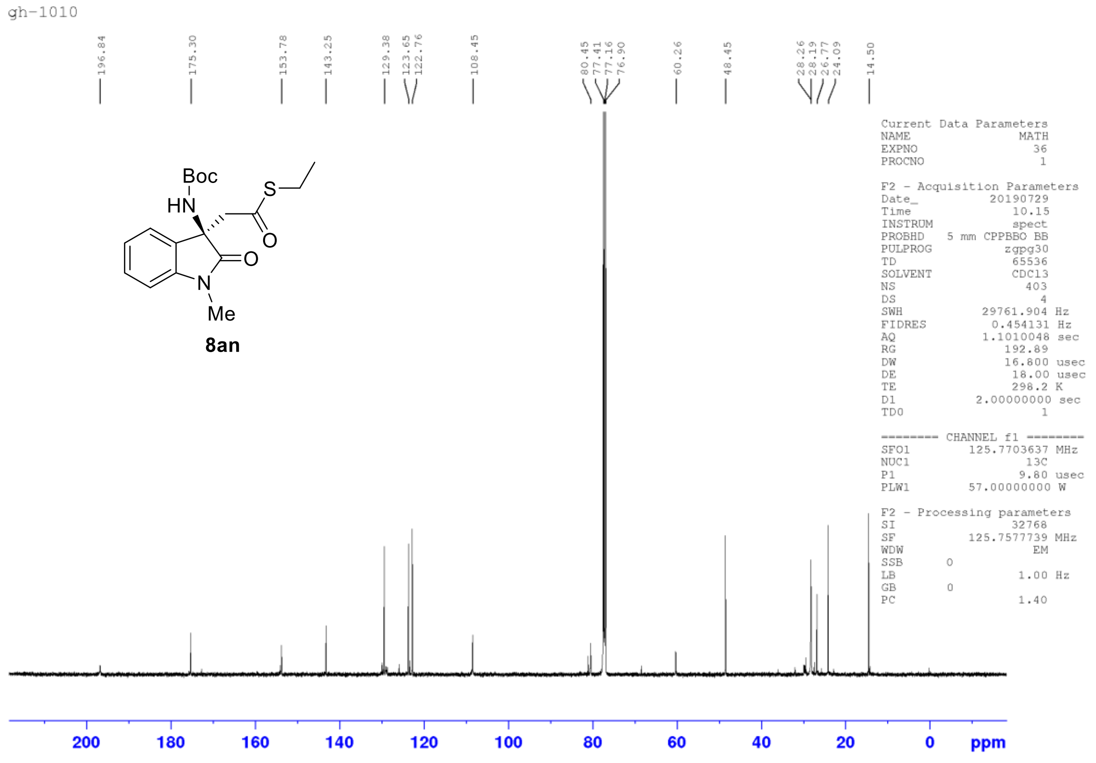

^{1}H and ^{13}C NMR of **8an** in $CDCl_3$.

5. HPLC analysis of products

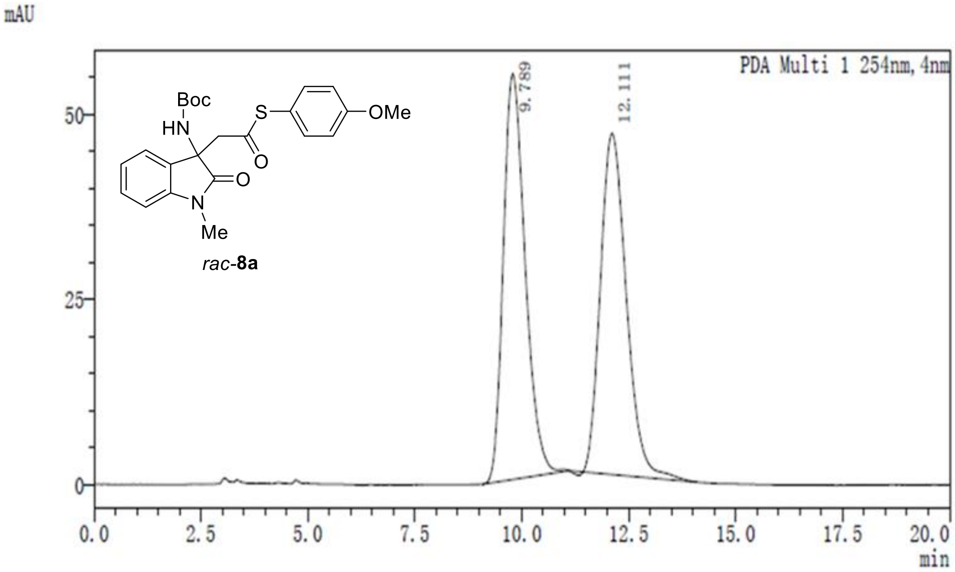

〈峰表〉

PDA Ch1 254nm

峰号	保留时间	面积	高度	浓度	面积%
1	9.789	1946007	54835	0.000	50.112
2	12.111	1937288	46099	0.000	49.888
总计		3883295	100934		100.000

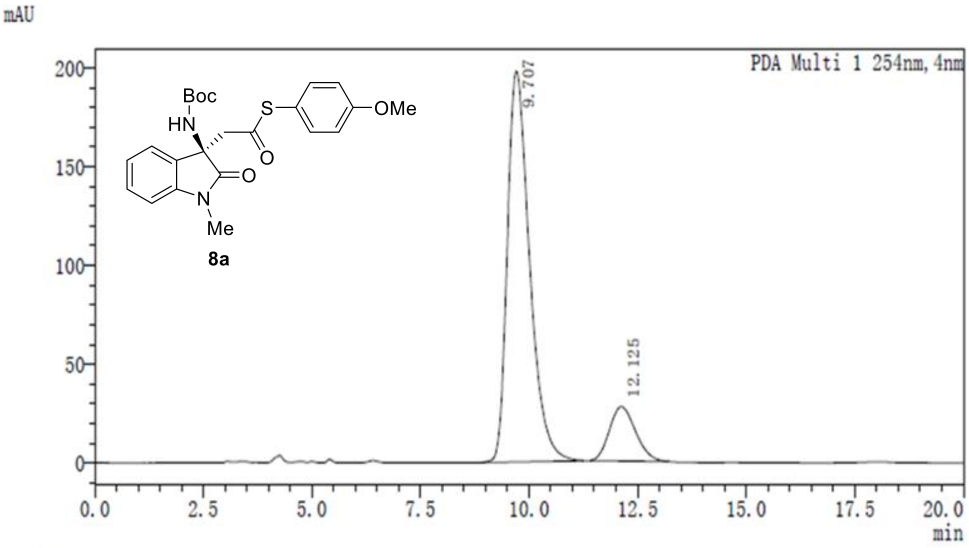

〈峰表〉

PDA Ch1 254nm

峰号	保留时间	面积	高度	浓度	面积%
1	9.707	7033891	197836	0.000	85.919
2	12.125	1152719	27471	0.000	14.081
总计		8186610	225307		100.000

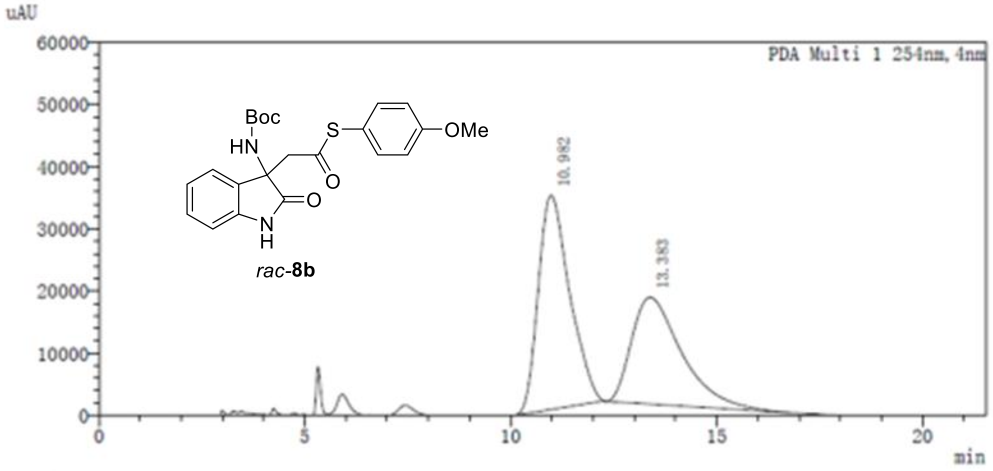

〈峰表〉
PDA Ch1 254nm

峰号	保留时间	面积	高度	浓度	面积%
1	10.982	1831467	34489	0.000	55.687
2	13.383	1457400	17186	0.000	44.313
总计		3288867	51675		100.000

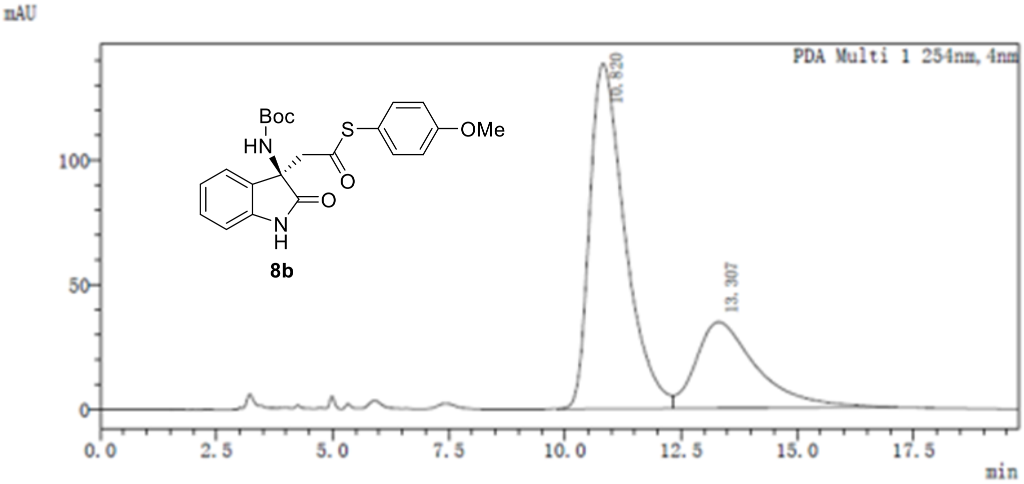

〈峰表〉
PDA Ch1 254nm

峰号	保留时间	面积	高度	浓度	面积%
1	10.820	7324254	138701	0.000	69.900
2	13.307	3153898	34598	0.000	30.100
总计		10478152	173299		100.000

〈色谱图〉

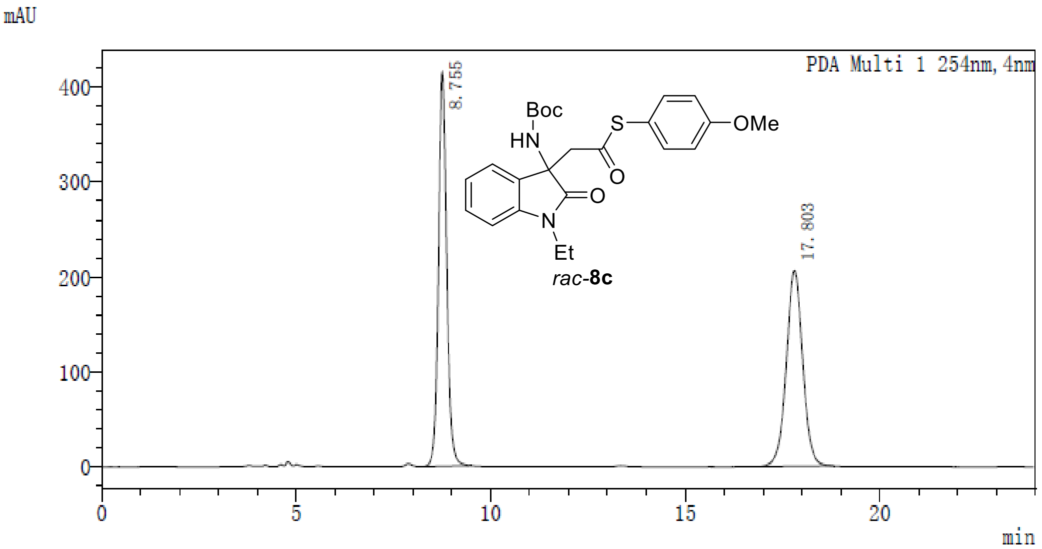

rac-**8c**

〈峰表〉

PDA Ch1 254nm

峰号	保留时间	面积	高度	浓度	面积%
1	8.755	6112636	414432	0.000	50.176
2	17.803	6069733	206014	0.000	49.824
总计		12182369	620447		100.000

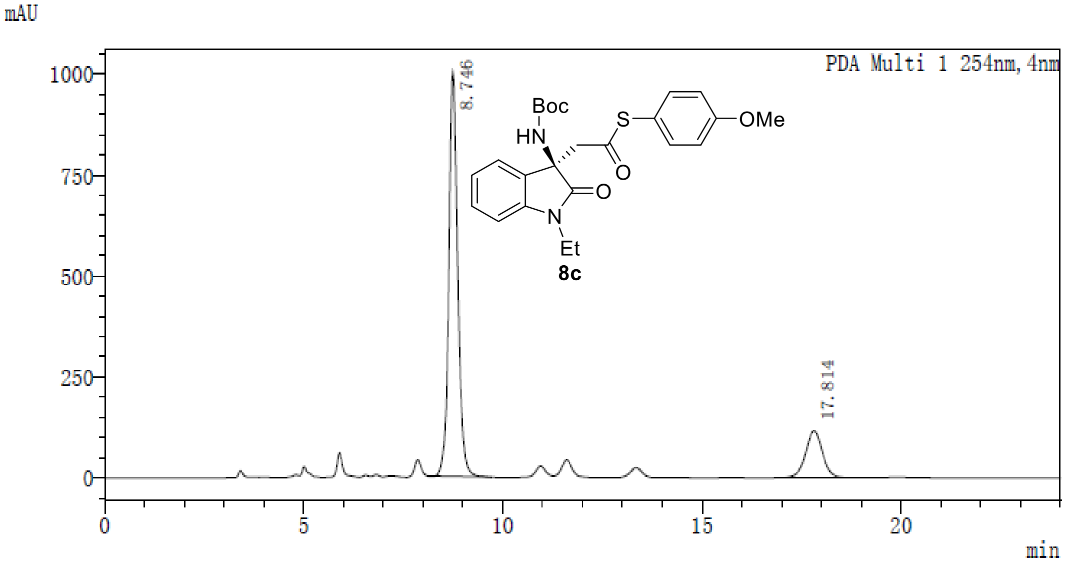

8c

〈峰表〉

PDA Ch1 254nm

峰号	保留时间	面积	高度	浓度	面积%
1	8.746	15428265	1001473	0.000	82.049
2	17.814	3375567	115767	0.000	17.951
总计		18803833	1117240		100.000

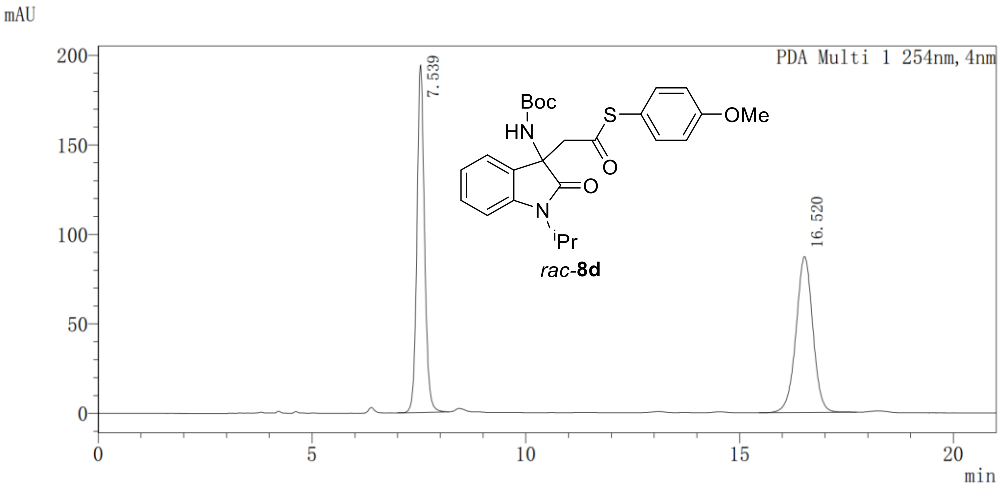

〈峰表〉
PDA Ch1 254nm

峰号	保留时间	面积	高度	浓度	面积%
1	7.539	2395184	193877	0.000	50.115
2	16.520	2384220	87147	0.000	49.885
总计		4779404	281024		100.000

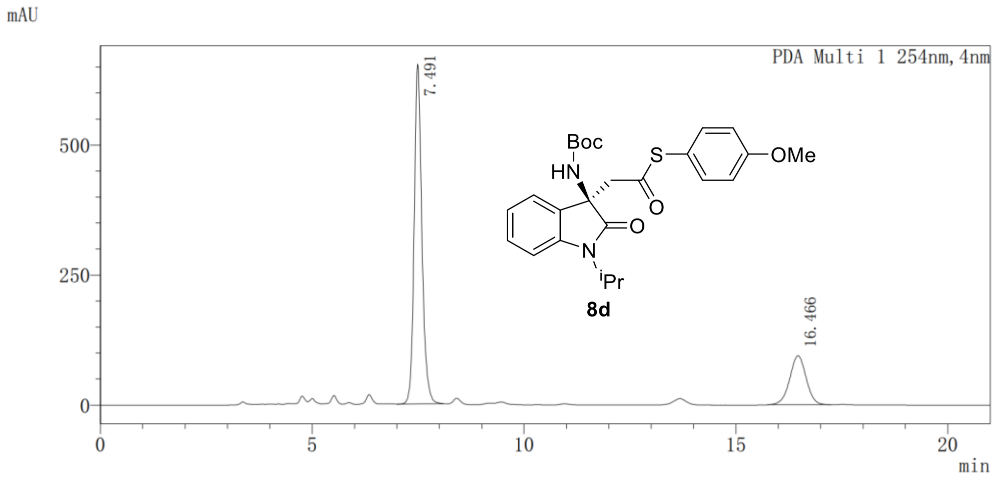

〈峰表〉
PDA Ch1 254nm

峰号	保留时间	面积	高度	浓度	面积%
1	7.491	8248820	652474	0.000	76.469
2	16.466	2538278	94229	0.000	23.531
总计		10787098	746703		100.000

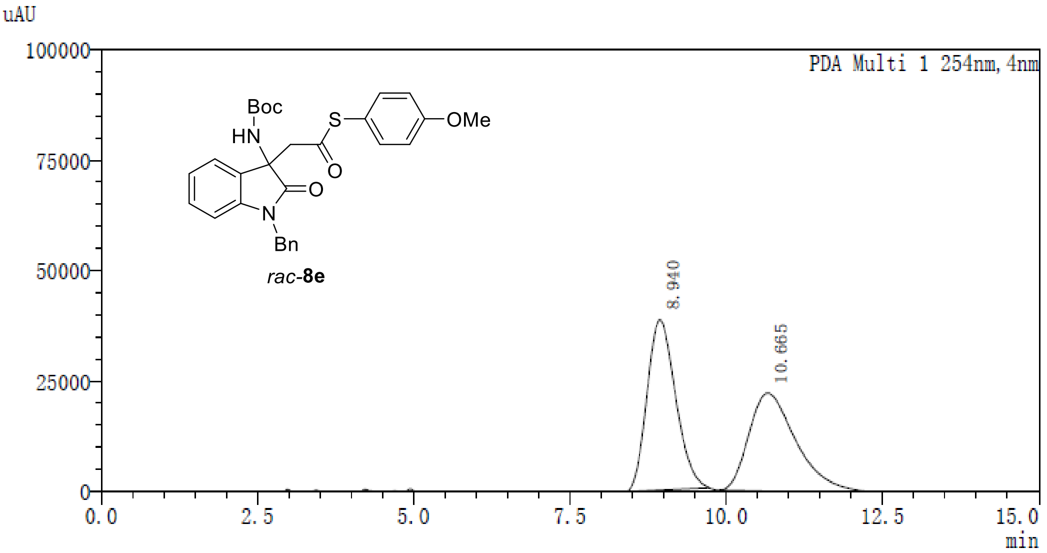

〈峰表〉

PDA Ch1 254nm

峰号	保留时间	面积	高度	浓度	面积%
1	8.940	1229256	38580	0.000	50.691
2	10.665	1195719	22240	0.000	49.309
总计		2424975	60820		100.000

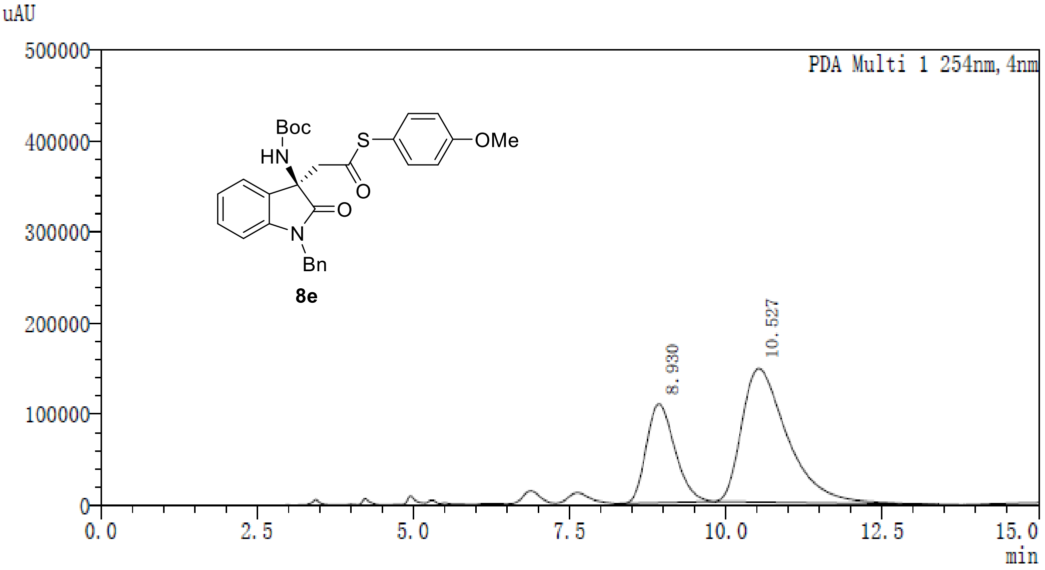

〈峰表〉

PDA Ch1 254nm

峰号	保留时间	面积	高度	浓度	面积%
1	8.930	3454245	108370	0.000	31.667
2	10.527	7453846	146375	0.000	68.333
总计		10908091	254746		100.000

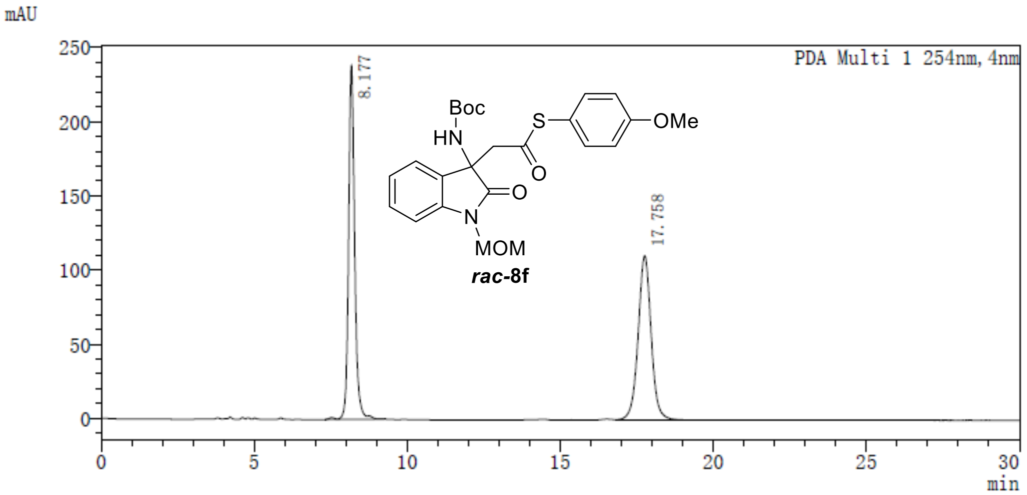

〈峰表〉

PDA Ch1 254nm

峰号	保留时间	面积	高度	浓度
1	8.177	3313185	238547	0.000
2	17.758	3272564	110586	0.000
总计		6585749	349132	

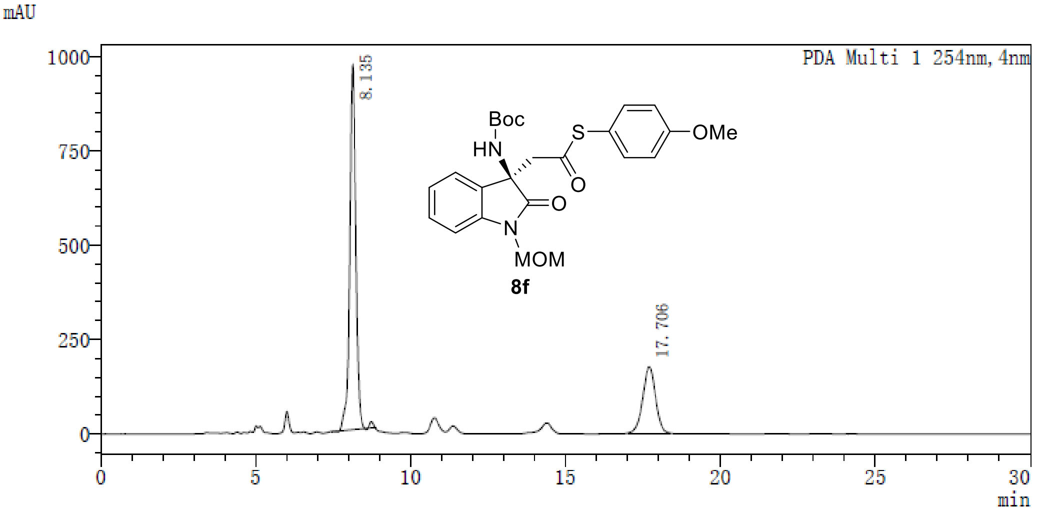

〈峰表〉

PDA Ch1 254nm

峰号	保留时间	面积	高度	浓度	面积%
1	8.135	13149577	964587	0.000	72.704
2	17.706	4936958	174493	0.000	27.296
总计		18086535	1139080		100.000

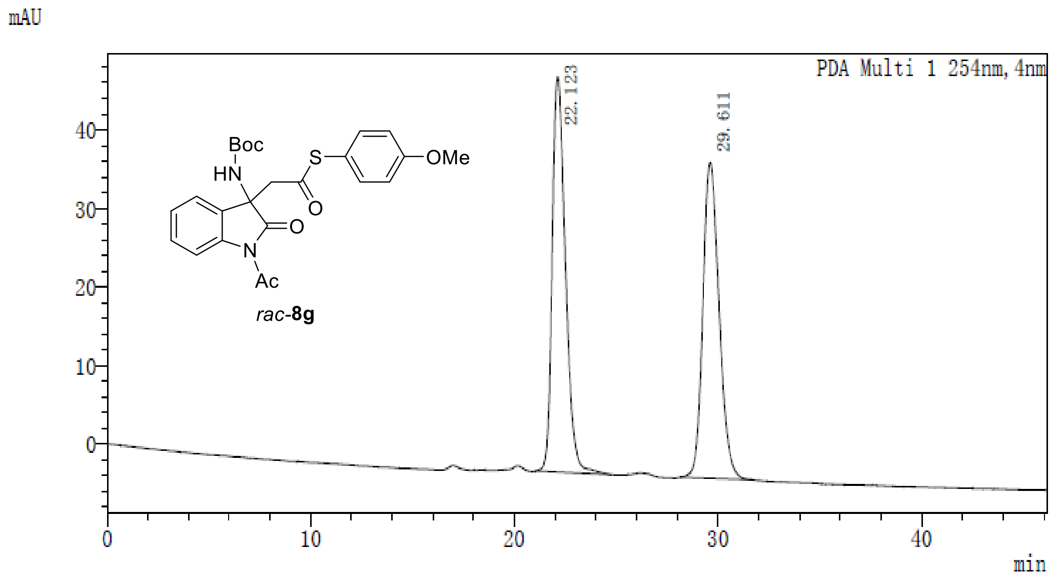

〈峰表〉

PDA Ch1 254nm

峰号	保留时间	面积	高度	浓度	面积%
1	22.123	2265217	50277	0.000	50.431
2	29.611	2226541	40237	0.000	49.569
总计		4491758	90514		100.000

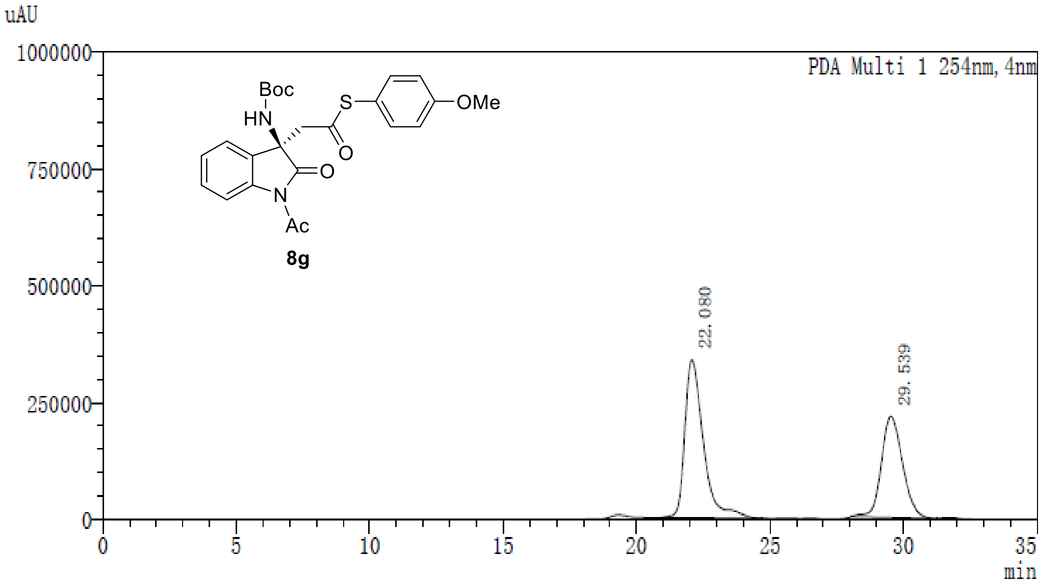

〈峰表〉

PDA Ch1 254nm

峰号	保留时间	面积	高度	浓度	面积%
1	22.080	16428902	338235	0.000	57.916
2	29.539	11937641	216310	0.000	42.084
总计		28366543	554546		100.000

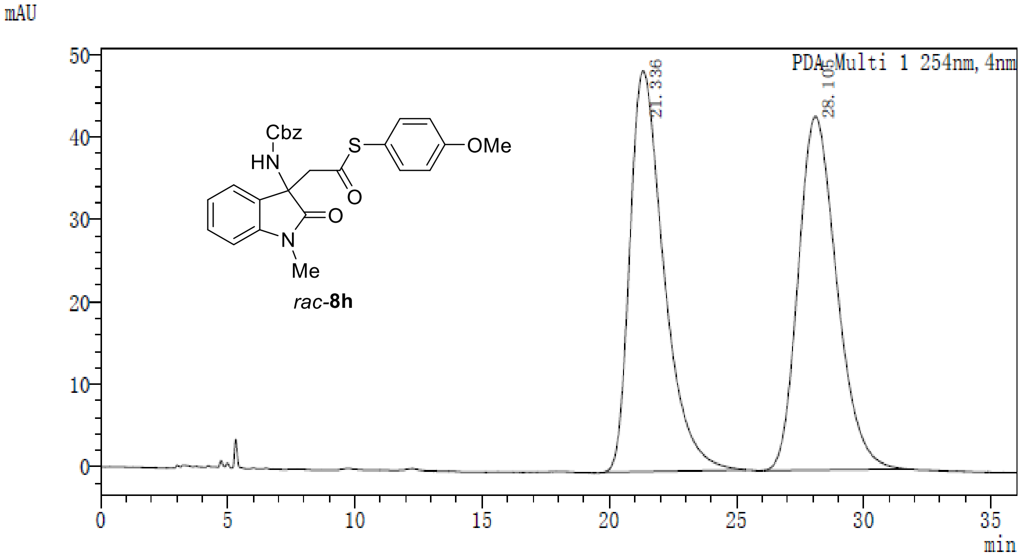

〈峰表〉

PDA Ch1 254nm

峰号	保留时间	面积	高度	浓度	面积%
1	21.336	4524060	48567	0.000	50.051
2	28.105	4514838	42857	0.000	49.949
总计		9038898	91424		100.000

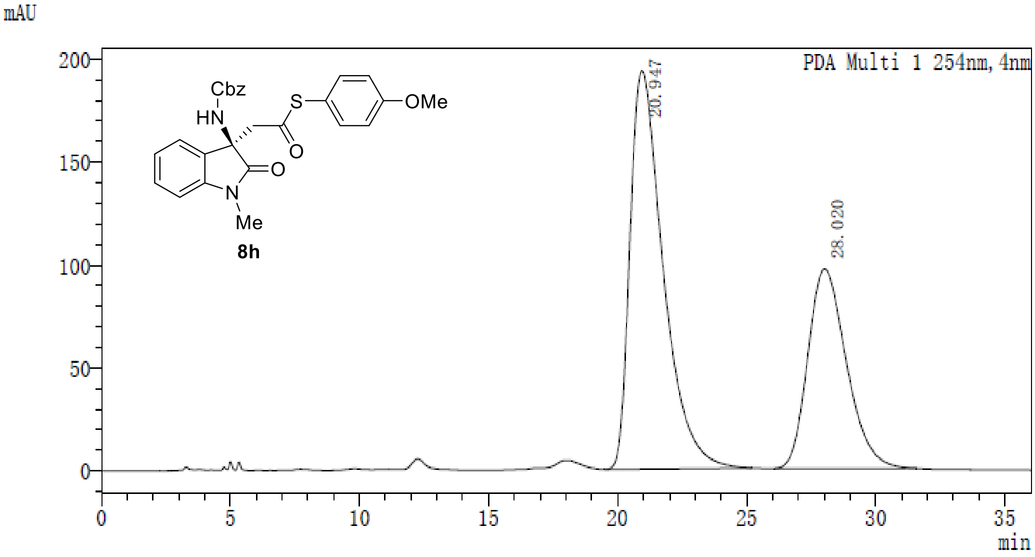

〈峰表〉

PDA Ch1 254nm

峰号	保留时间	面积	高度	浓度	面积%
1	20.947	17311634	193442	0.000	63.153
2	28.020	10100566	96915	0.000	36.847
总计		27412200	290357		100.000

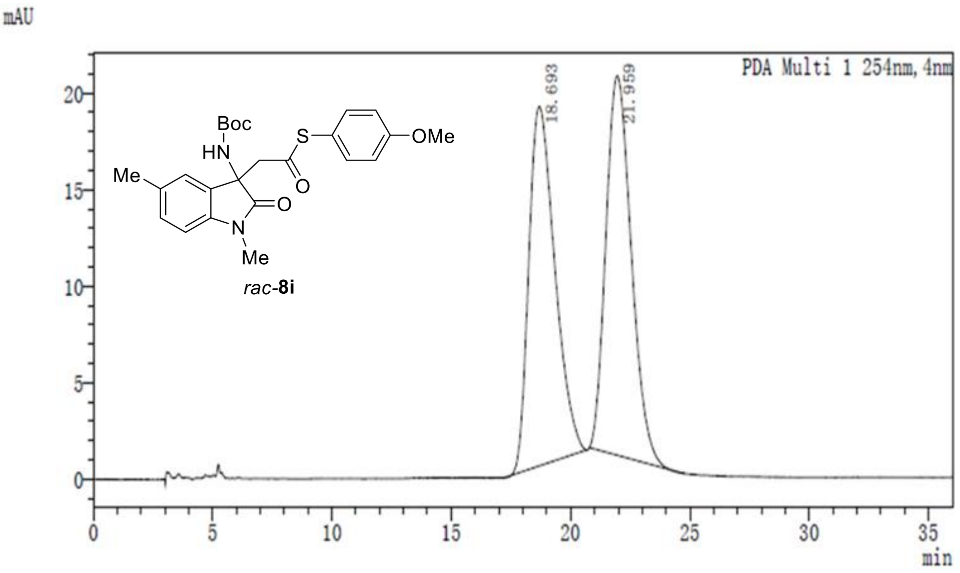

〈峰表〉

PDA Ch1 254nm

峰号	保留时间	面积	高度	浓度	面积%
1	18.693	1414011	18623	0.000	49.246
2	21.959	1457331	19659	0.000	50.754
总计		2871342	38282		100.000

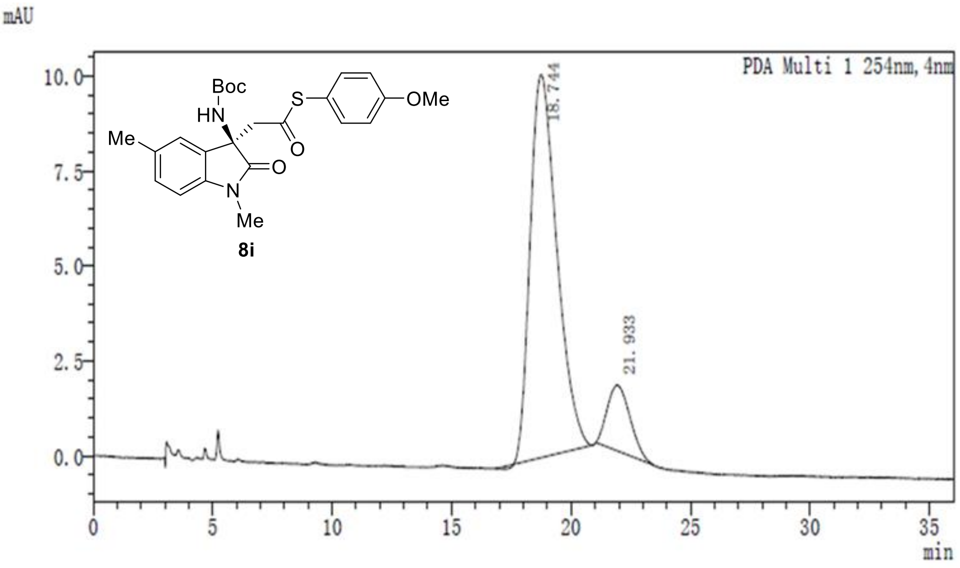

〈峰表〉

PDA Ch1 254nm

峰号	保留时间	面积	高度	浓度	面积%
1	18.744	784925	10089	0.000	87.206
2	21.933	115153	1728	0.000	12.794
总计		900078	11816		100.000

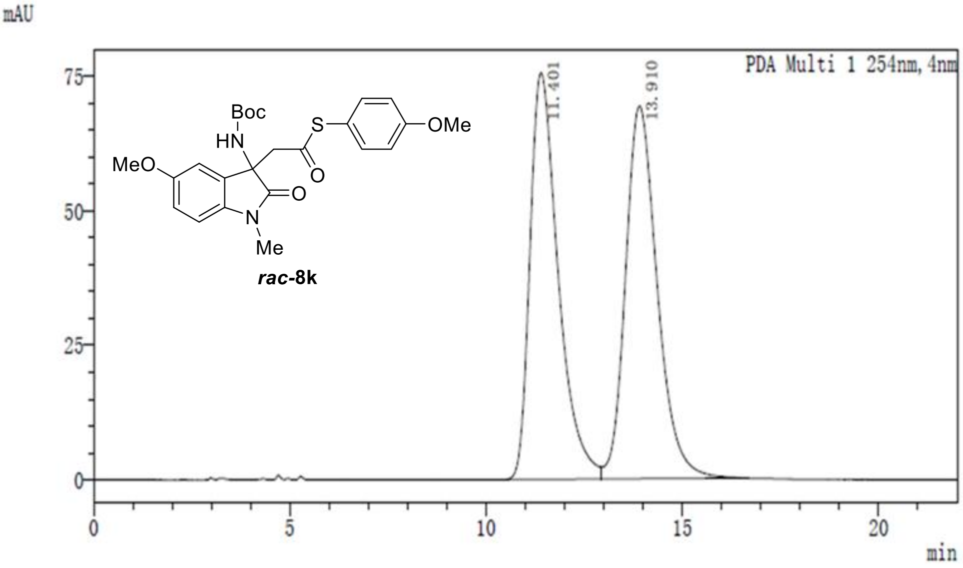

〈峰表〉

PDA Ch1 254nm

峰号	保留时间	面积	高度	浓度	面积%
1	11.401	3781435	75503	0.000	49.246
2	13.910	3897306	69246	0.000	50.754
总计		7678740	144748		100.000

〈色谱图〉

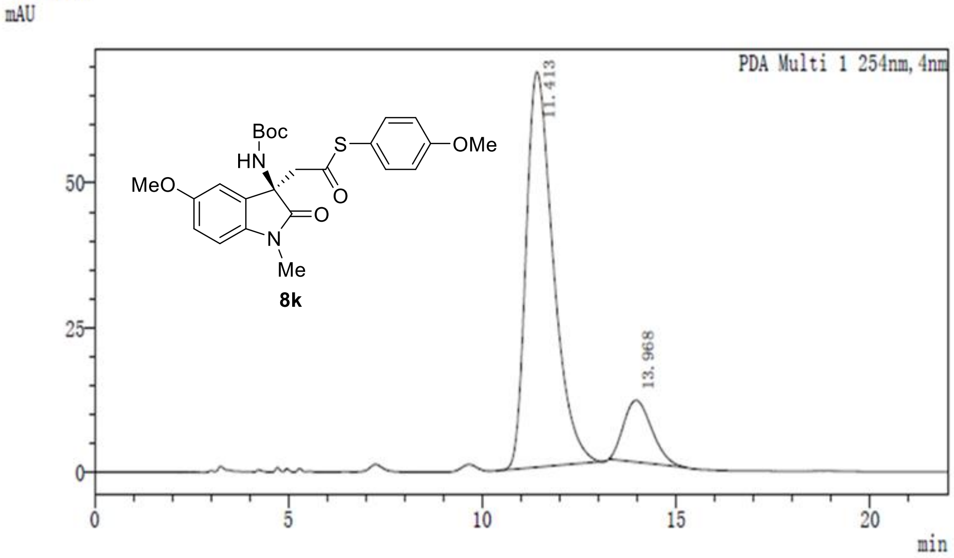

〈峰表〉

PDA Ch1 254nm

峰号	保留时间	面积	高度	浓度	面积%
1	11.413	3338786	68450	0.000	85.980
2	13.968	544422	10694	0.000	14.020
总计		3883208	79144		100.000

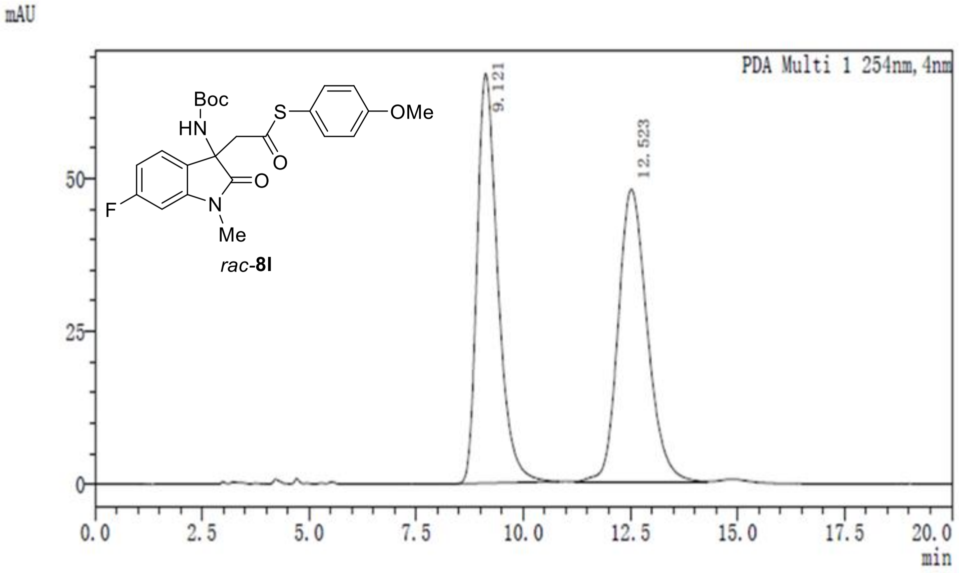

〈峰表〉

PDA Ch1 254nm

峰号	保留时间	面积	高度	浓度	面积%
1	9.121	2255680	67094	0.000	49.972
2	12.523	2258248	47911	0.000	50.028
总计		4513928	115005		100.000

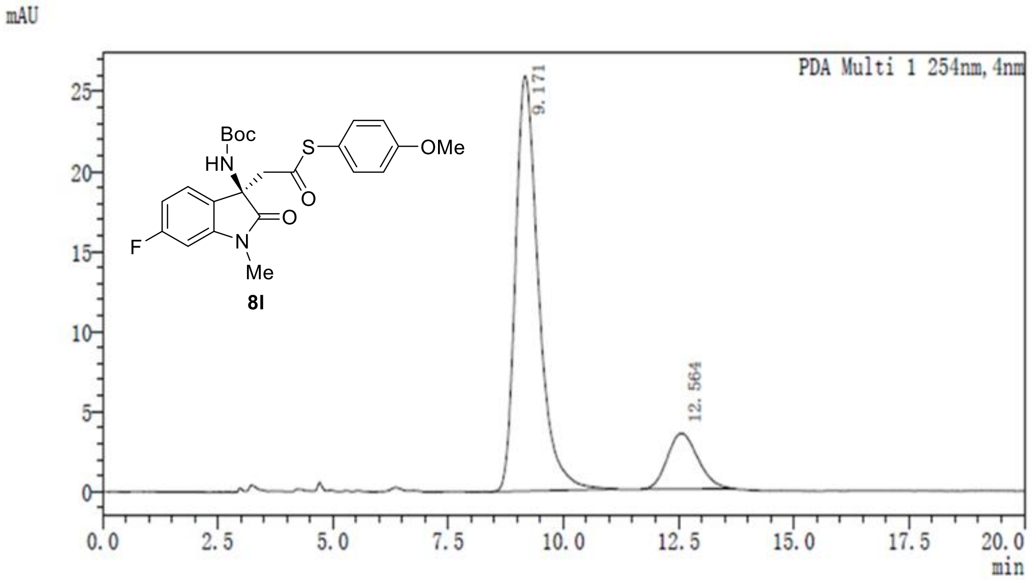

〈峰表〉

PDA Ch1 254nm

峰号	保留时间	面积	高度	浓度	面积%
1	9.171	901846	25934	0.000	84.799
2	12.564	161666	3481	0.000	15.201
总计		1063511	29415		100.000

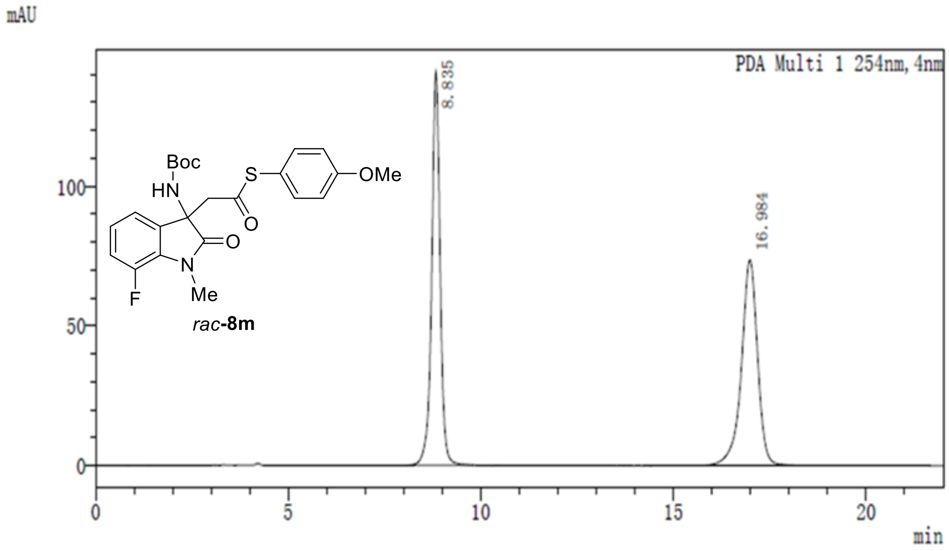

〈峰表〉
PDA Ch1 254nm

峰号	保留时间	面积	高度	浓度	面积%
1	8.835	2149350	140840	0.000	50.096
2	16.984	2141123	73574	0.000	49.904
总计		4290473	214415		100.000

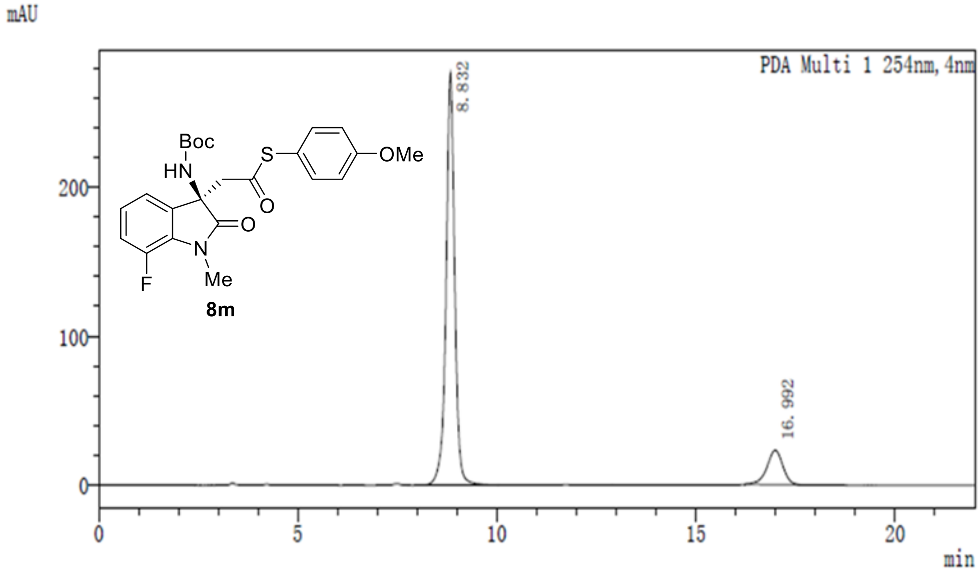

〈峰表〉
PDA Ch1 254nm

峰号	保留时间	面积	高度	浓度	面积%
1	8.832	4194467	276147	0.000	86.806
2	16.992	637544	23029	0.000	13.194
总计		4832011	299176		100.000

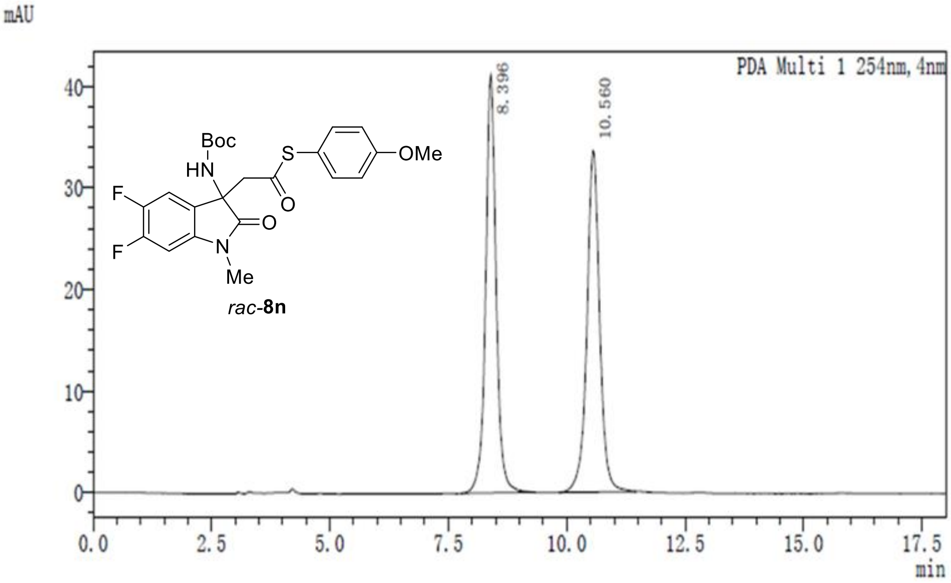

〈峰表〉

PDA Ch1 254nm

峰号	保留时间	面积	高度	浓度	面积%
1	8.396	634460	41090	0.000	50.162
2	10.560	630361	33589	0.000	49.838
总计		1264822	74679		100.000

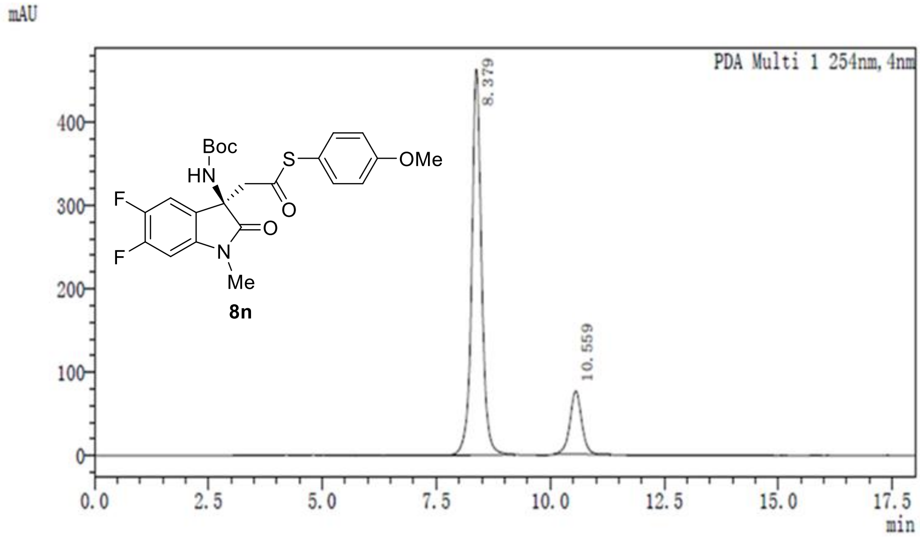

〈峰表〉

PDA Ch1 254nm

峰号	保留时间	面积	高度	浓度	面积%
1	8.379	6949587	461871	0.000	83.538
2	10.559	1369477	75794	0.000	16.462
总计		8319064	537664		100.000

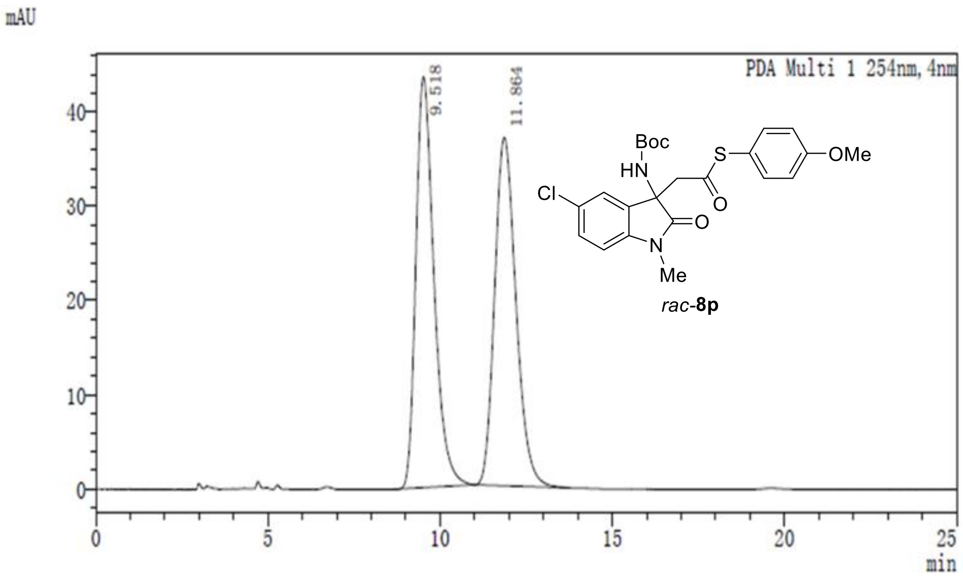

〈峰表〉

PDA Ch1 254nm

峰号	保留时间	面积	高度	浓度	面积%
1	9.518	1589244	43572	0.000	49.957
2	11.864	1591981	36964	0.000	50.043
总计		3181225	80537		100.000

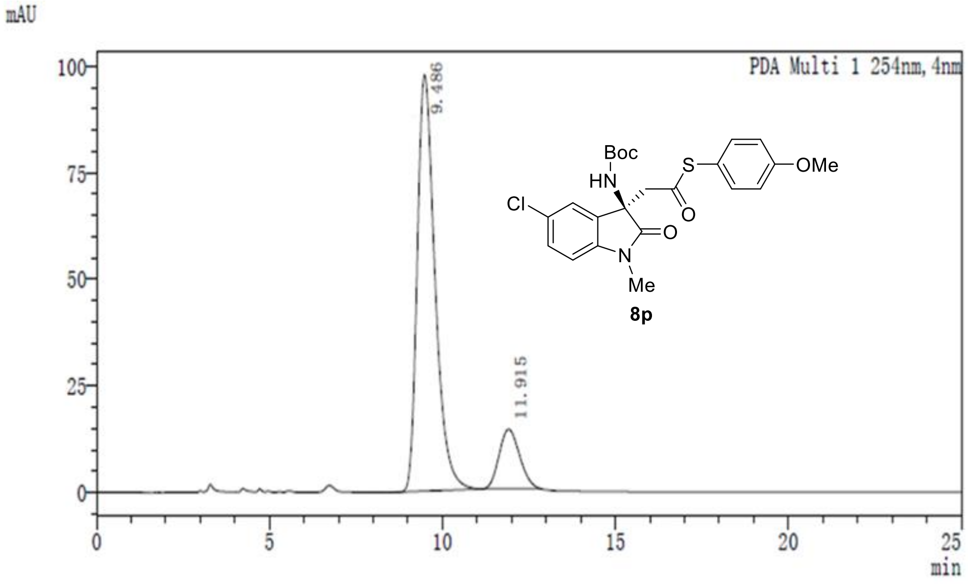

〈峰表〉

PDA Ch1 254nm

峰号	保留时间	面积	高度	浓度	面积%
1	9.486	3482044	97590	0.000	85.859
2	11.915	573511	13909	0.000	14.141
总计		4055555	111499		100.000

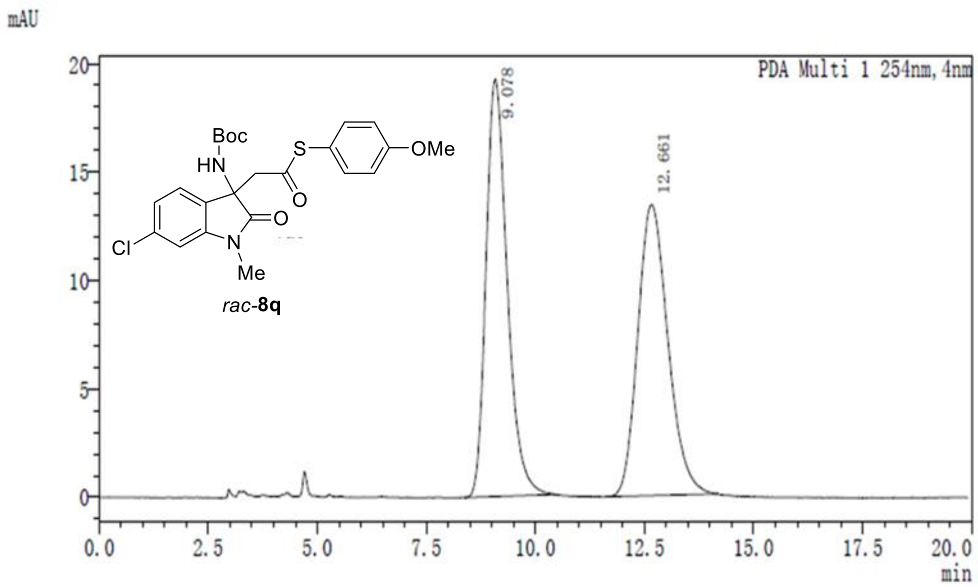

〈峰表〉

PDA Ch1 254nm

峰号	保留时间	面积	高度	浓度	面积%
1	9.078	649435	19242	0.000	50.196
2	12.661	644363	13414	0.000	49.804
总计		1293798	32656		100.000

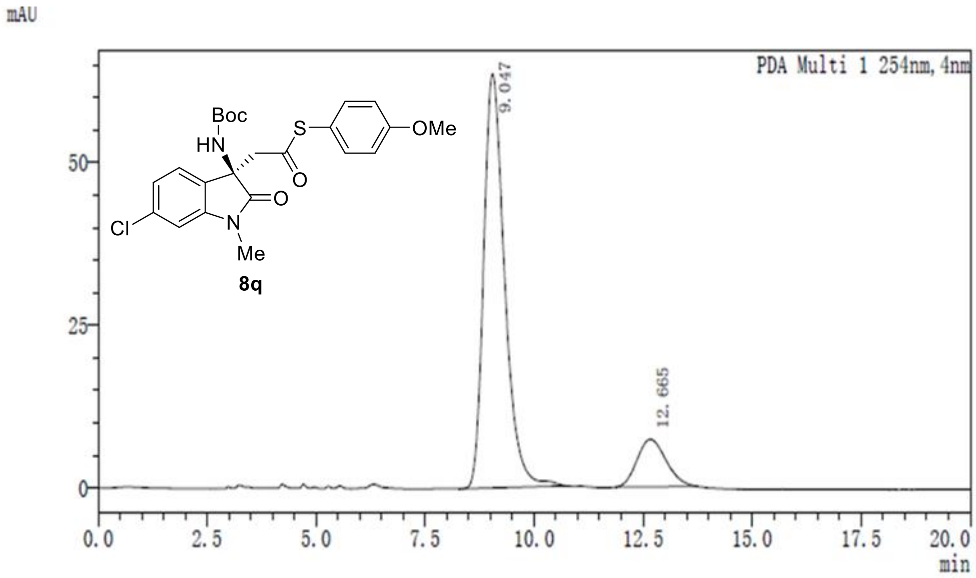

〈峰表〉

PDA Ch1 254nm

峰号	保留时间	面积	高度	浓度	面积%
1	9.047	2122092	63536	0.000	86.254
2	12.665	338182	7281	0.000	13.746
总计		2460274	70818		100.000

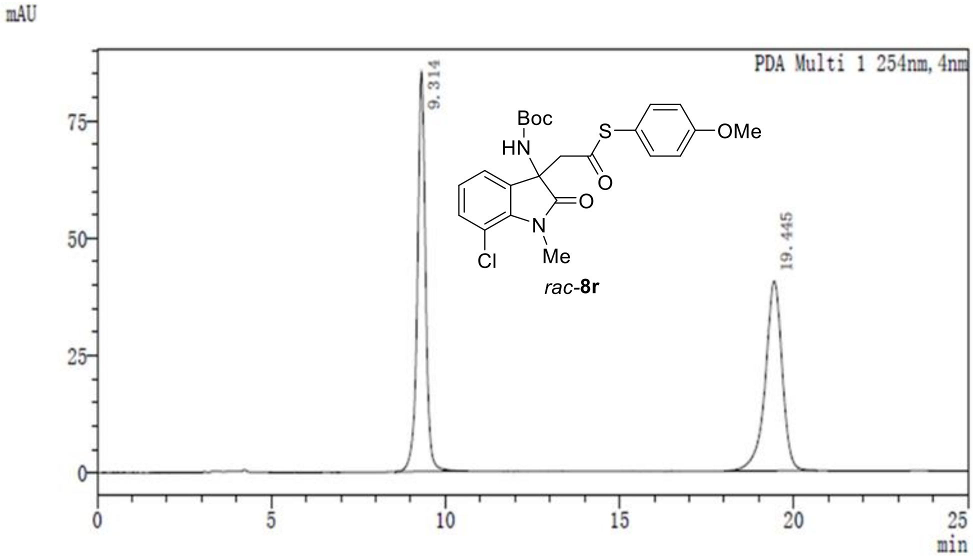

〈峰表〉

PDA Ch1 254nm

峰号	保留时间	面积	高度	浓度	面积%
1	9.314	1381821	85159	0.000	50.310
2	19.445	1364780	40400	0.000	49.690
总计		2746601	125559		100.000

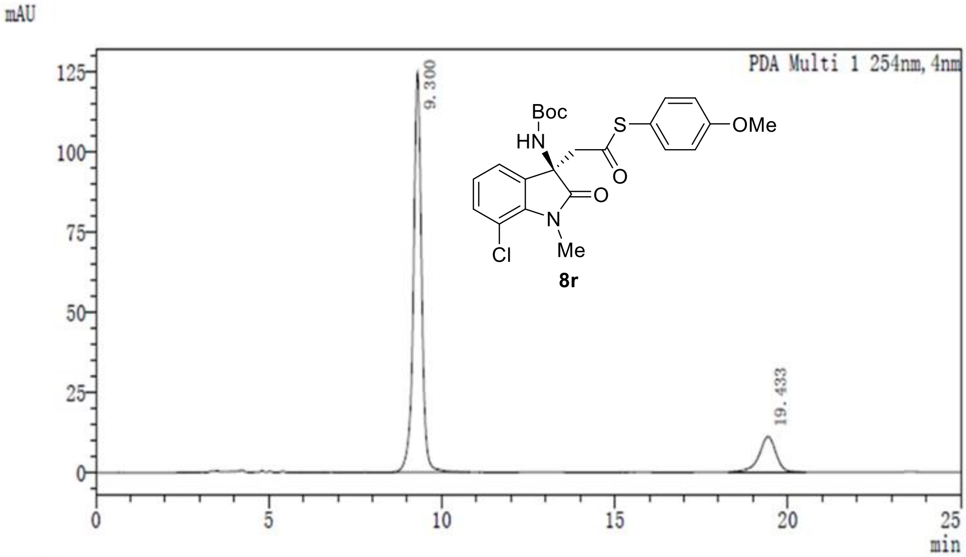

〈峰表〉

PDA Ch1 254nm

峰号	保留时间	面积	高度	浓度	面积%
1	9.300	2029257	124672	0.000	84.599
2	19.433	369428	11007	0.000	15.401
总计		2398685	135680		100.000

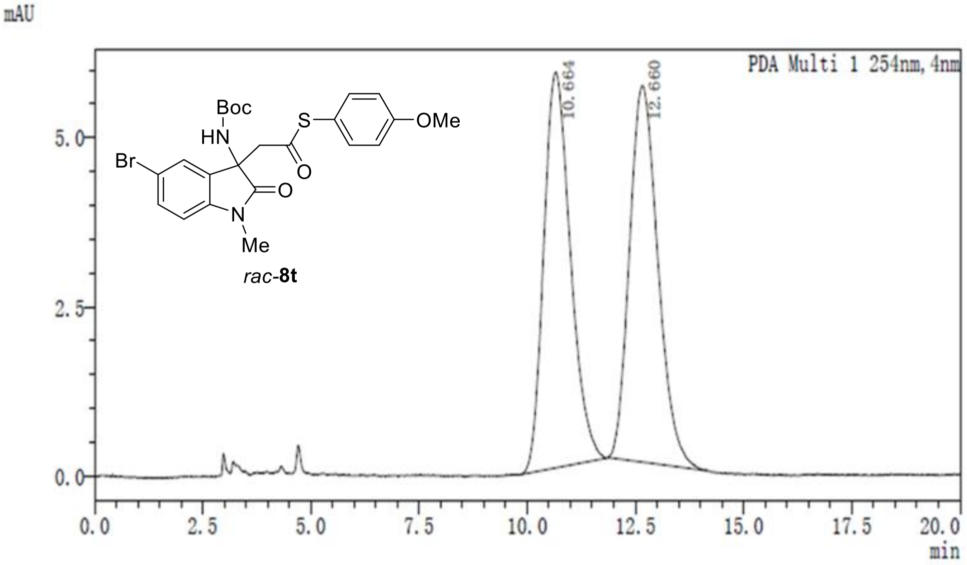

〈峰表〉

PDA Ch1 254nm

峰号	保留时间	面积	高度	浓度	面积%
1	10.664	250574	5838	0.000	49.631
2	12.660	254301	5558	0.000	50.369
总计		504875	11396		100.000

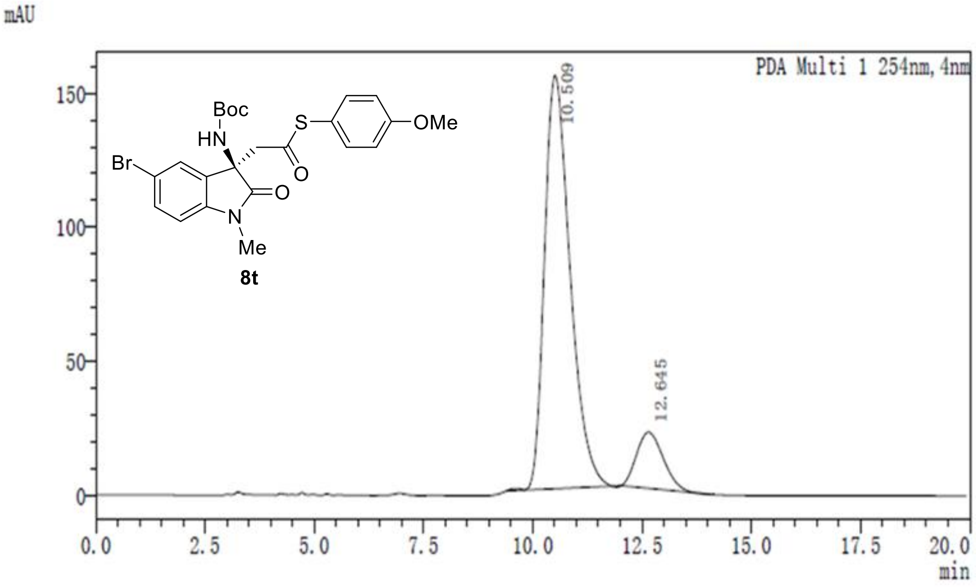

〈峰表〉

PDA Ch1 254nm

峰号	保留时间	面积	高度	浓度	面积%
1	10.509	6291145	154391	0.000	87.494
2	12.645	899188	20973	0.000	12.506
总计		7190333	175364		100.000

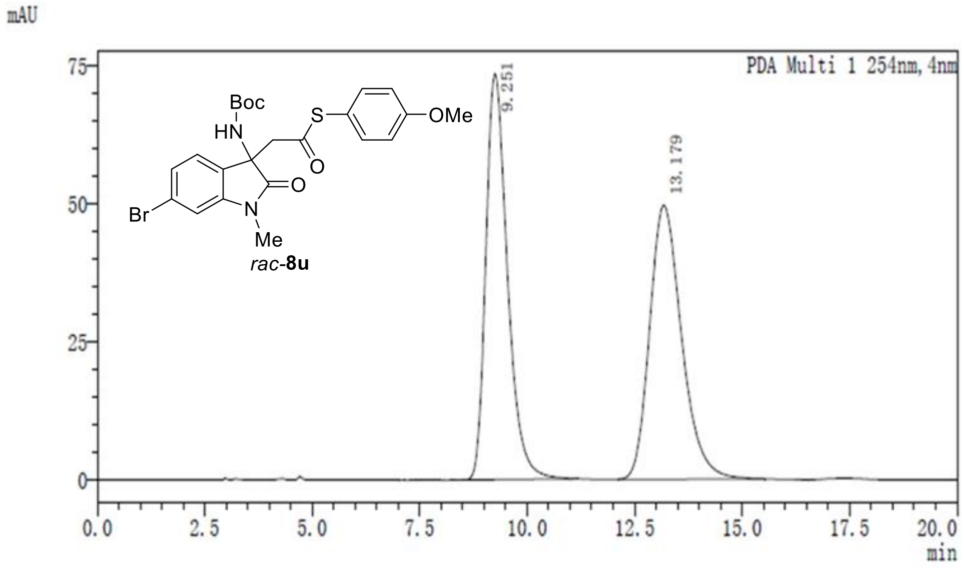

〈峰表〉

PDA Ch1 254nm

峰号	保留时间	面积	高度	浓度	高度%
1	9.251	2569205	73499	0.000	59.722
2	13.179	2573350	49571	0.000	40.278
总计		5142555	123070		100.000

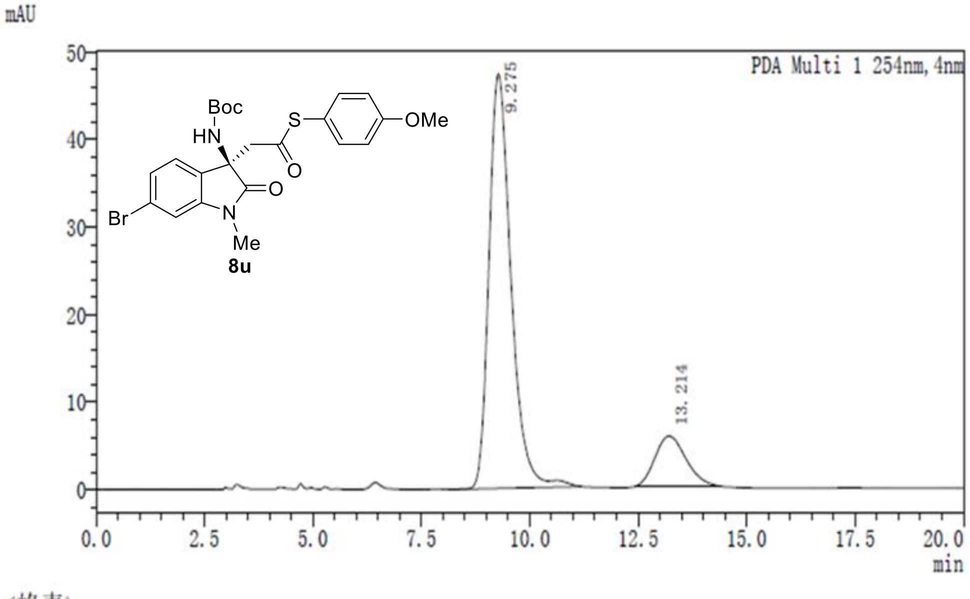

〈峰表〉

PDA Ch1 254nm

峰号	保留时间	面积	高度	浓度	面积%
1	9.275	1674261	47300	0.000	85.479
2	13.214	284410	5732	0.000	14.521
总计		1958671	53031		100.000

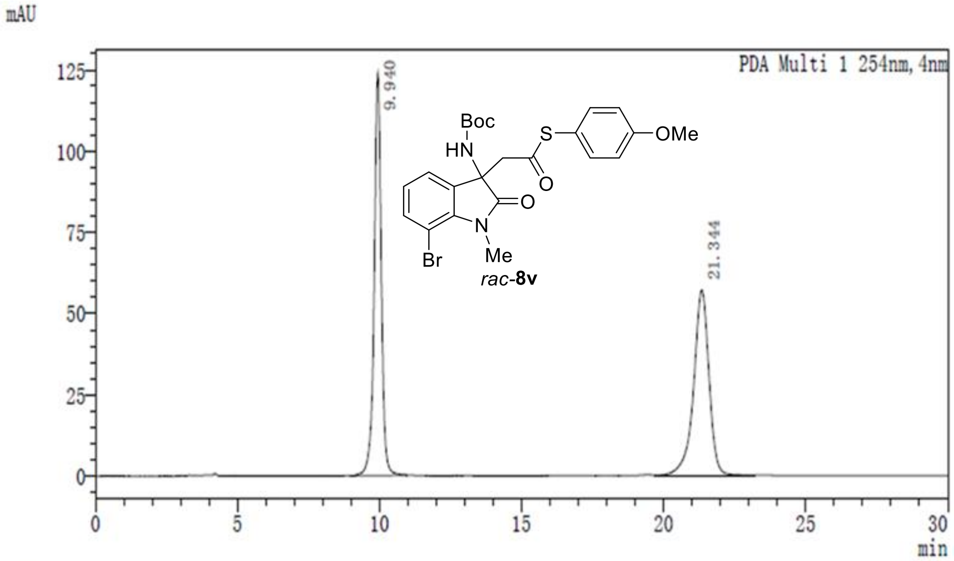

〈峰表〉

PDA Ch1 254nm

峰号	保留时间	面积	高度	浓度	面积%
1	9.940	2157561	123945	0.000	50.157
2	21.344	2144081	56942	0.000	49.843
总计		4301642	180887		100.000

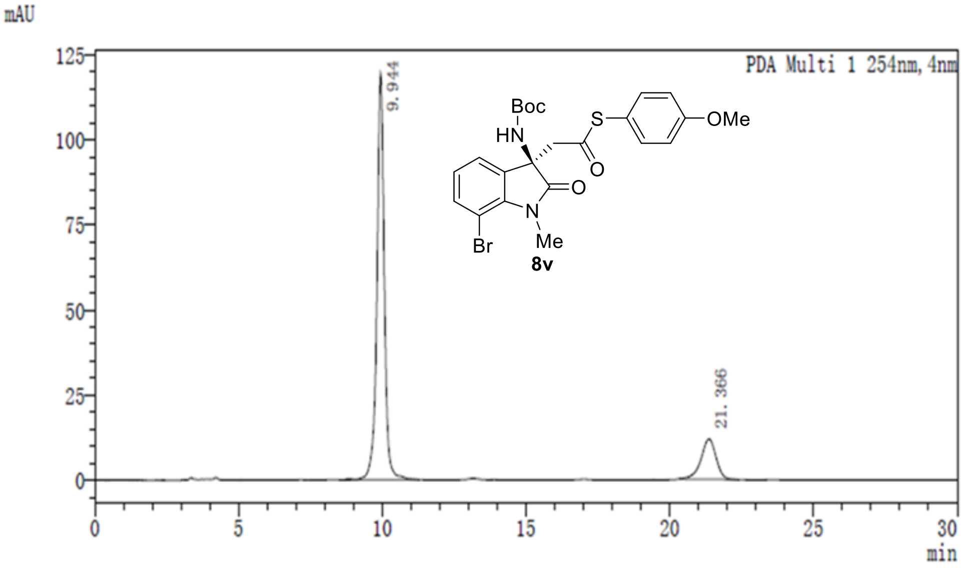

〈峰表〉

PDA Ch1 254nm

峰号	保留时间	面积	高度	浓度	面积%
1	9.944	2130311	119411	0.000	83.149
2	21.366	431738	11823	0.000	16.851
总计		2562049	131235		100.000

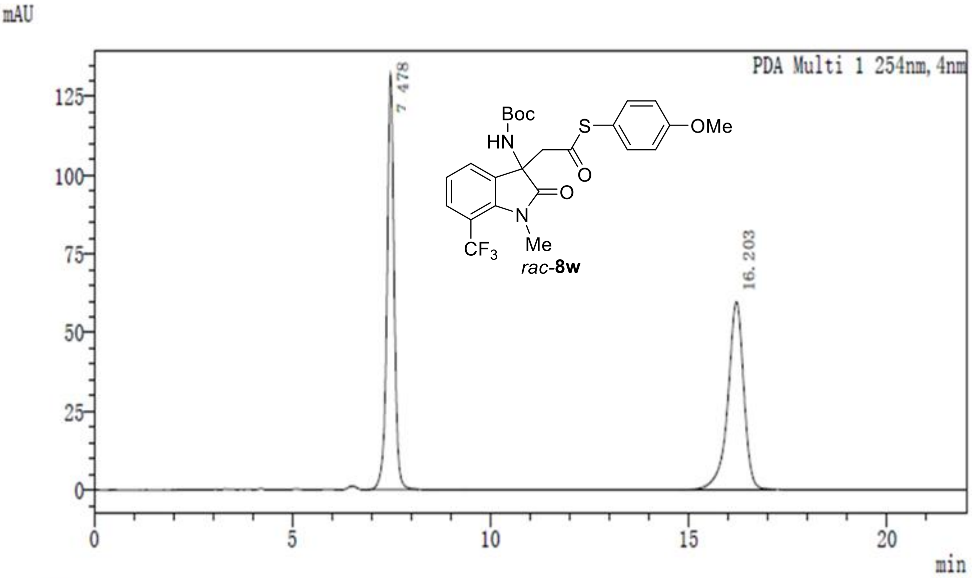

〈峰表〉
PDA Ch1 254nm

峰号	保留时间	面积	高度	浓度	面积%
1	7.478	1664886	131841	0.000	50.028
2	16.203	1663035	59596	0.000	49.972
总计		3327921	191437		100.000

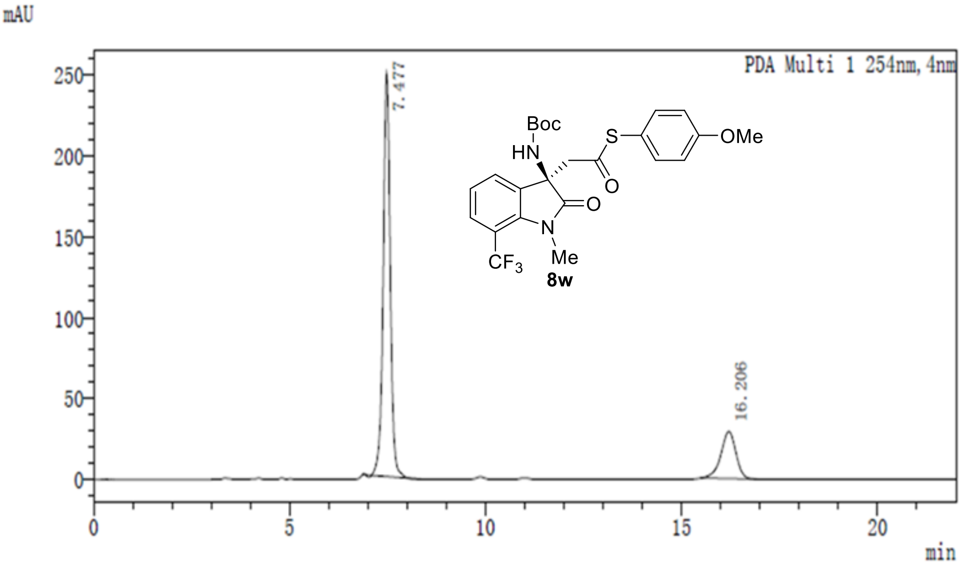

〈峰表〉
PDA Ch1 254nm

峰号	保留时间	面积	高度	浓度	面积%
1	7.477	3109390	248910	0.000	79.958
2	16.206	779387	29110	0.000	20.042
总计		3888777	278020		100.000

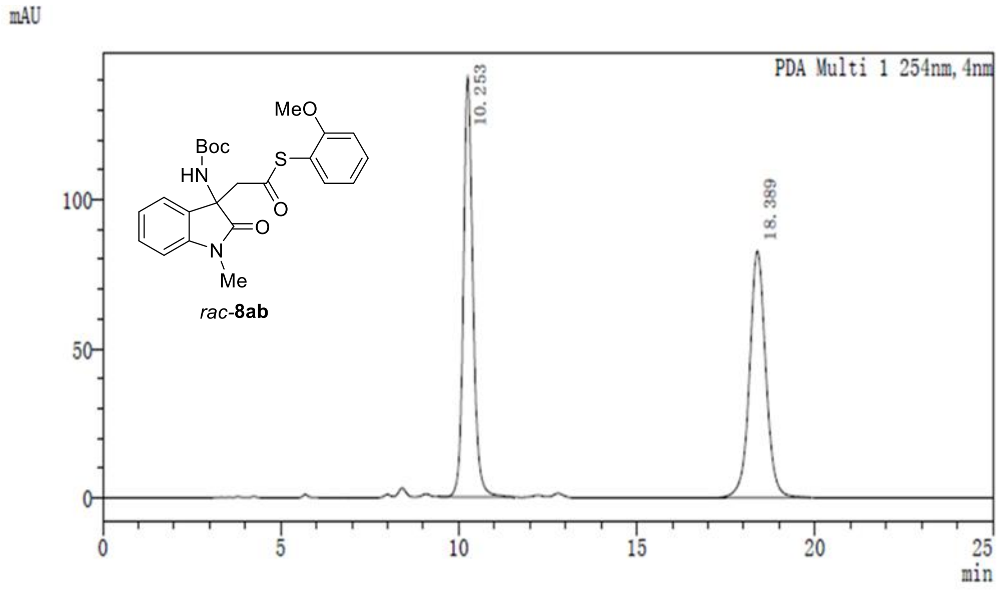

〈峰表〉

PDA Ch1 254nm

峰号	保留时间	面积	高度	浓度	面积%
1	10.253	2619447	140802	0.000	50.022
2	18.389	2617180	82690	0.000	49.978
总计		5236627	223492		100.000

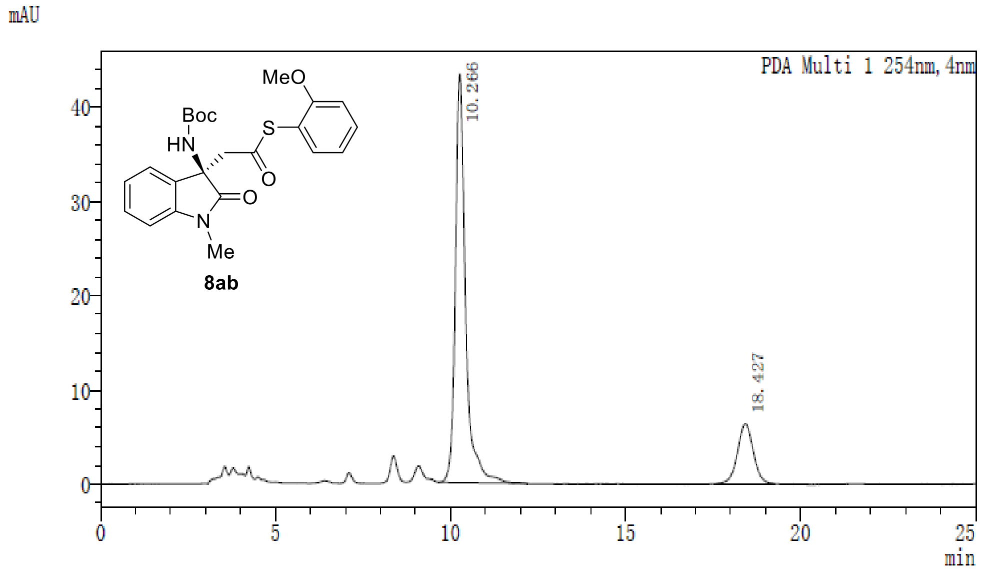

〈峰表〉

PDA Ch1 254nm

峰号	保留时间	面积	高度	浓度	面积%
1	10.266	882531	43316	0.000	81.547
2	18.427	199700	6367	0.000	18.453
总计		1082230	49683		100.000

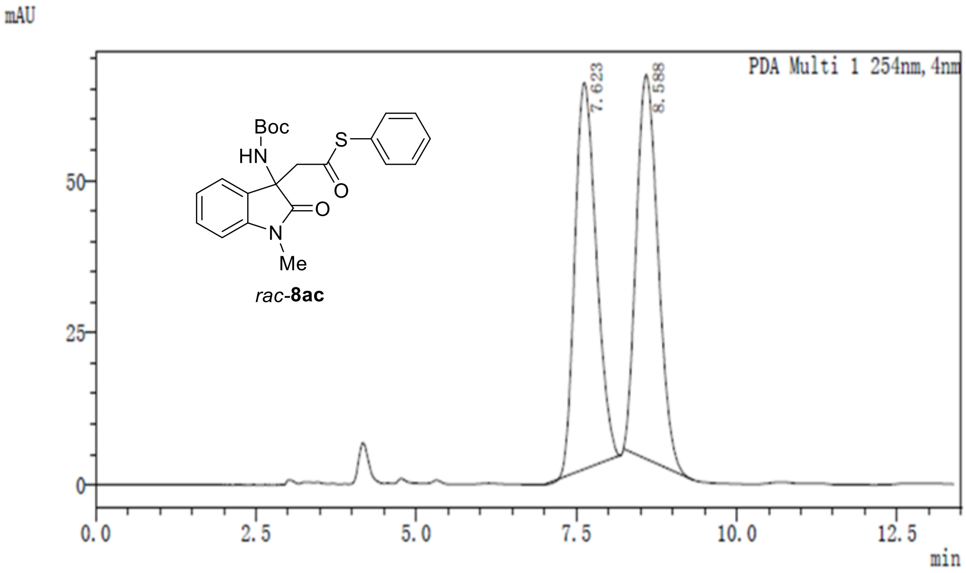

〈峰表〉
PDA Ch1 254nm

峰号	保留时间	面积	高度	浓度	面积%
1	7.623	1475176	63458	0.000	49.851
2	8.588	1483966	63103	0.000	50.149
总计		2959143	126561		100.000

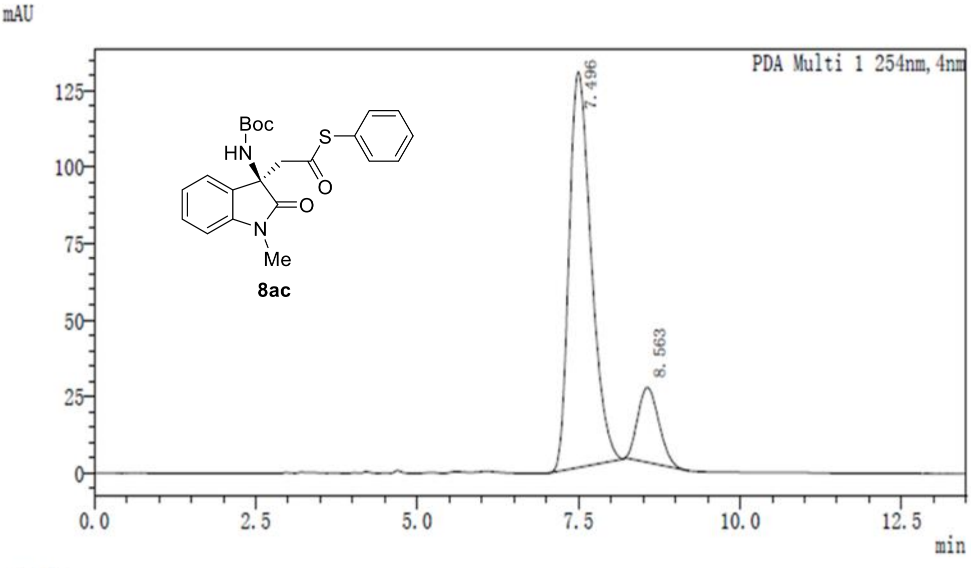

〈峰表〉
PDA Ch1 254nm

峰号	保留时间	面积	高度	浓度	面积%
1	7.496	3071329	129263	0.000	84.568
2	8.563	560455	24525	0.000	15.432
总计		3631784	153788		100.000

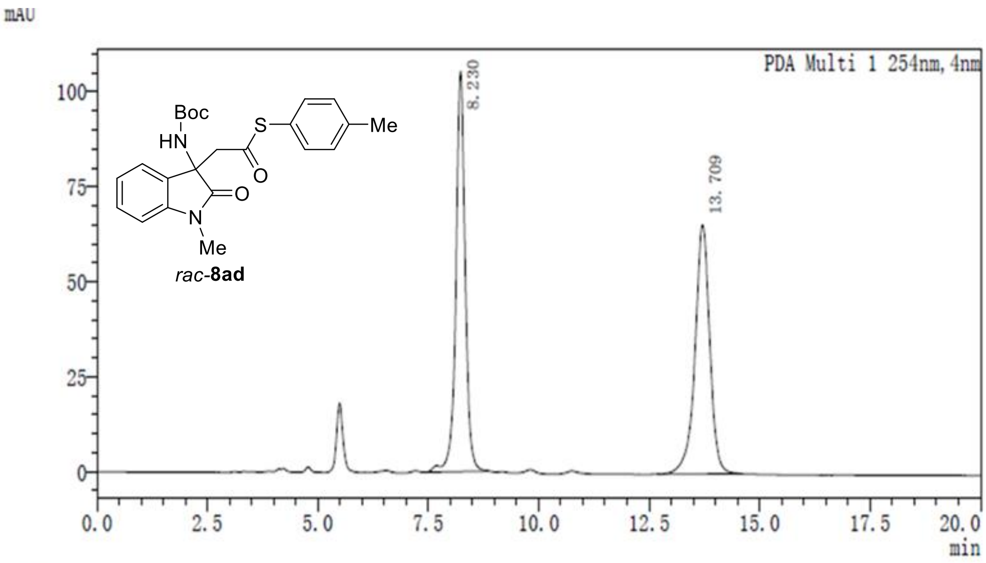

〈峰表〉

PDA Ch1 254nm

峰号	保留时间	面积	高度	浓度	面积%
1	8.230	1545812	105210	0.000	50.139
2	13.709	1537263	65603	0.000	49.861
总计		3083075	170812		100.000

〈色谱图〉

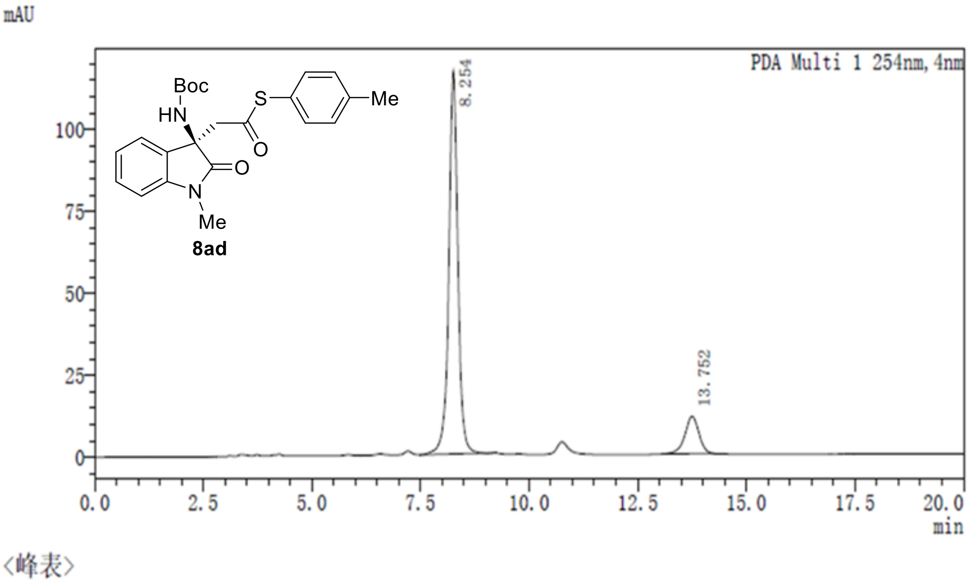

〈峰表〉

PDA Ch1 254nm

峰号	保留时间	面积	高度	浓度	面积%
1	8.254	1701699	117012	0.000	86.902
2	13.752	256479	11383	0.000	13.098
总计		1958179	128395		100.000

<色谱图>

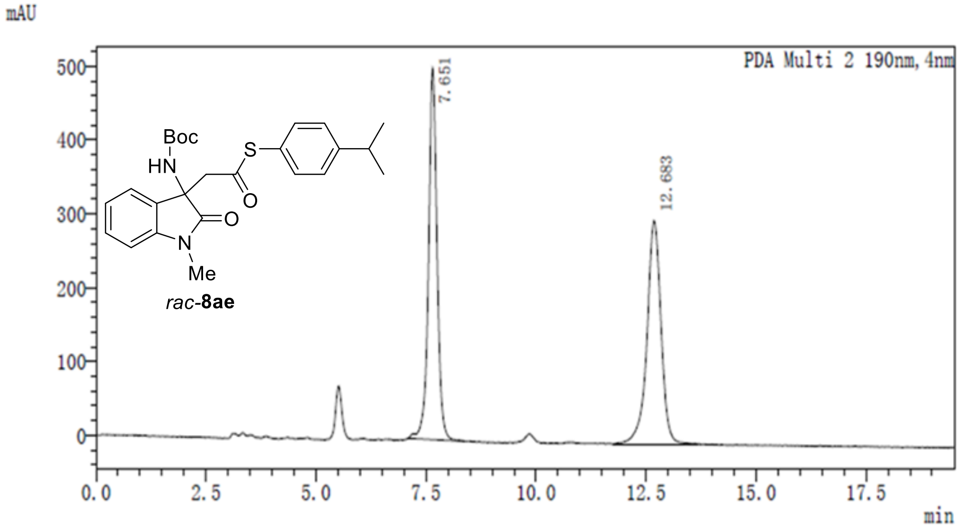

<峰表>

PDA Ch2 190nm

峰号	保留时间	面积	高度	浓度	面积%
1	7.651	6837933	503325	0.000	50.492
2	12.683	6704712	302569	0.000	49.508
总计		13542645	805894		100.000

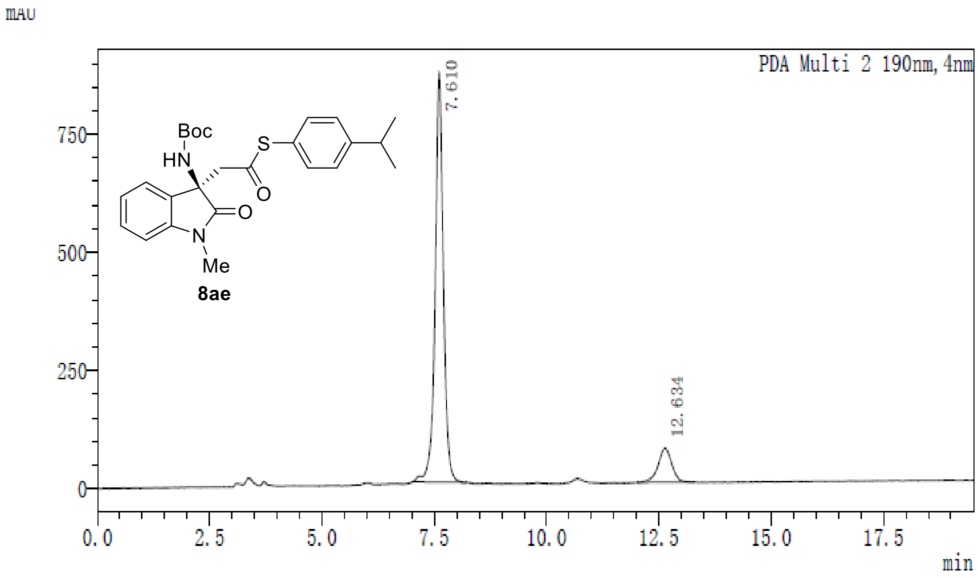

<峰表>

PDA Ch2 190nm

峰号	保留时间	面积	高度	浓度	面积%
1	7.610	11255712	866725	0.000	88.001
2	12.634	1534714	71715	0.000	11.999
总计		12790426	938440		100.000

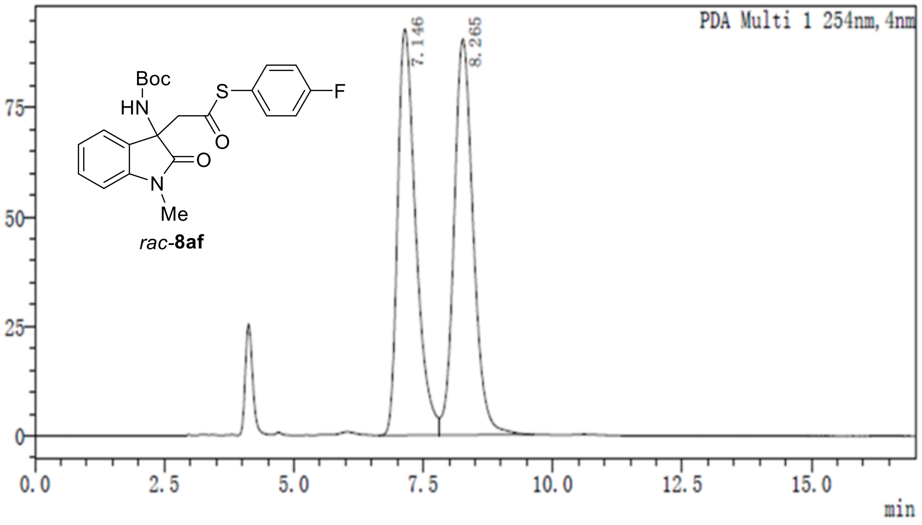

〈峰表〉

PDA Ch1 254nm

峰号	保留时间	面积	高度	浓度	面积%
1	7.146	2254949	92838	0.000	49.221
2	8.265	2326353	90466	0.000	50.779
总计		4581302	183304		100.000

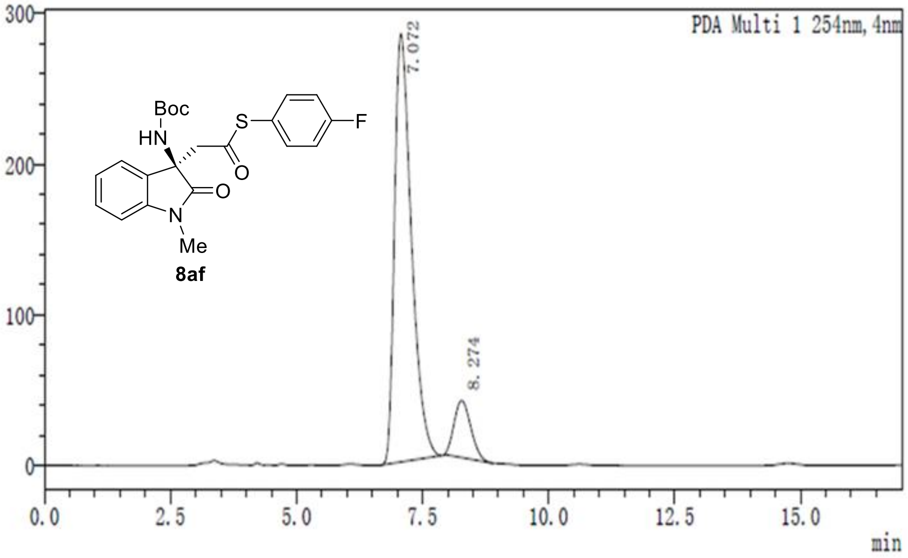

〈峰表〉

PDA Ch1 254nm

峰号	保留时间	面积	高度	浓度	面积%
1	7.072	6621035	283849	0.000	88.409
2	8.274	868097	37702	0.000	11.591
总计		7489132	321550		100.000

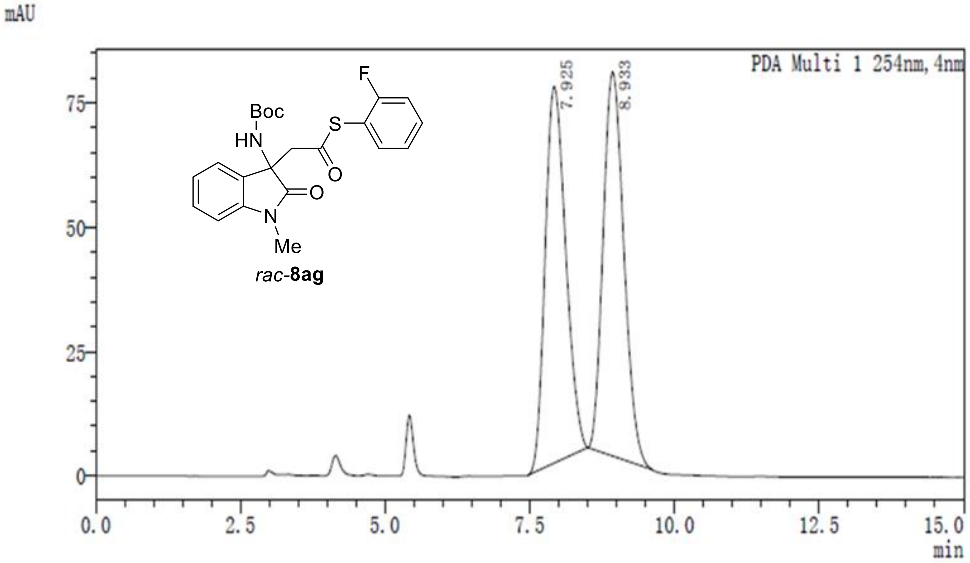

〈峰表〉

PDA Ch1 254nm

峰号	保留时间	面积	高度	浓度	面积%
1	7.925	1874320	75821	0.000	49.386
2	8.933	1920889	77255	0.000	50.614
总计		3795209	153077		100.000

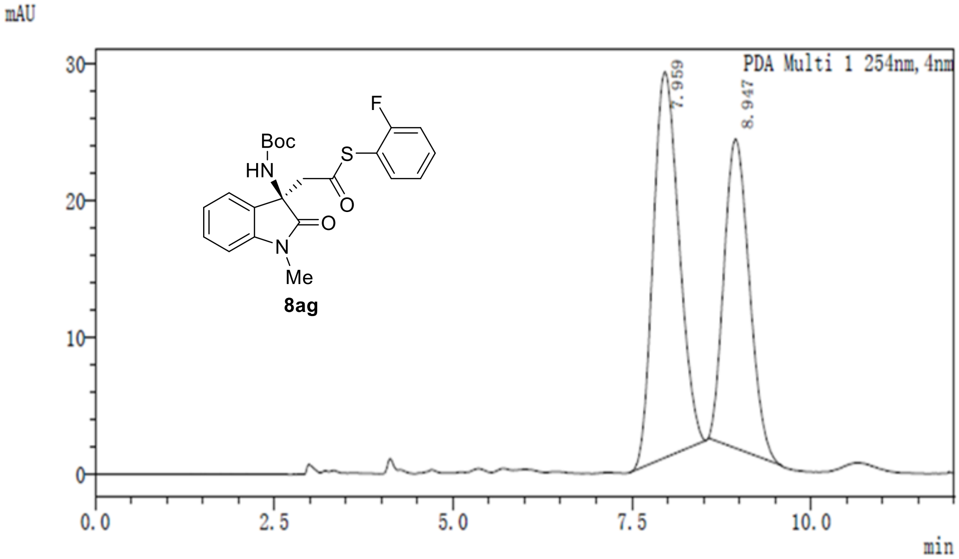

〈峰表〉

PDA Ch1 254nm

峰号	保留时间	面积	高度	浓度	面积%
1	7.959	708963	28263	0.000	56.079
2	8.947	555268	22639	0.000	43.921
总计		1264230	50901		100.000

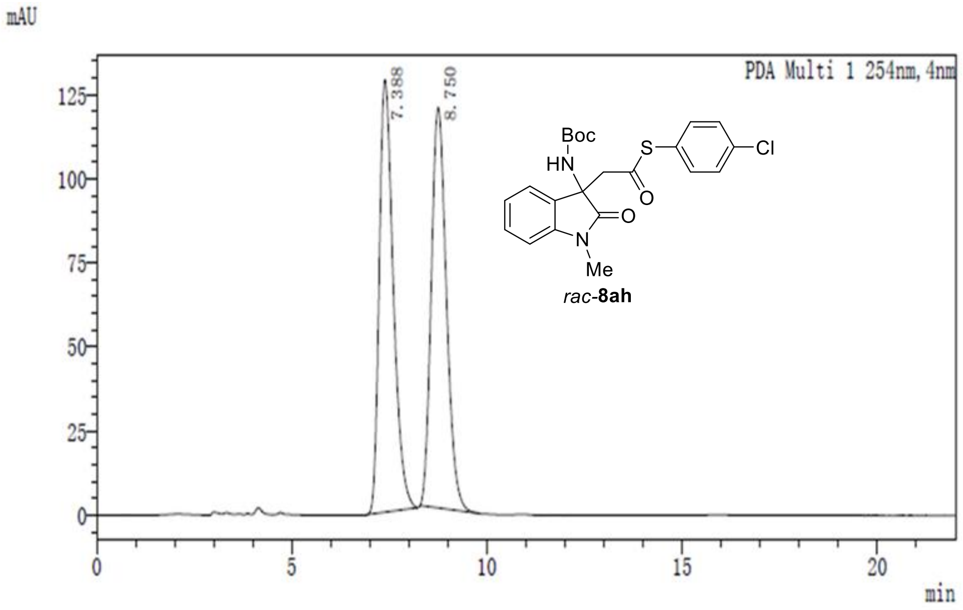

〈峰表〉

PDA Ch1 254nm

峰号	保留时间	面积	高度	浓度	面积%
1	7.388	3218332	128530	0.000	50.043
2	8.750	3212799	118997	0.000	49.957
总计		6431132	247527		100.000

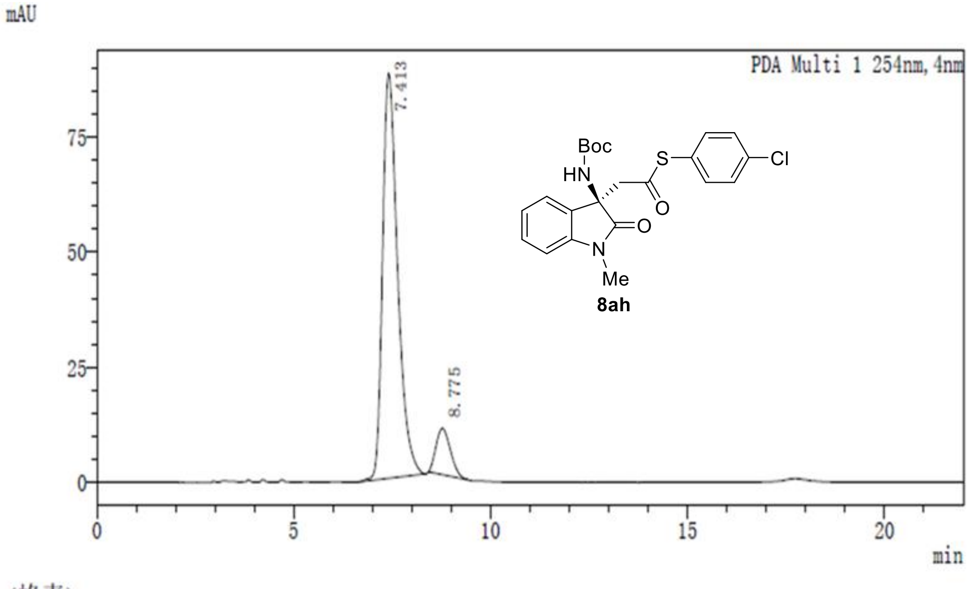

〈峰表〉

PDA Ch1 254nm

峰号	保留时间	面积	高度	浓度	面积%
1	7.413	2340103	88029	0.000	90.136
2	8.775	256083	10021	0.000	9.864
总计		2596187	98050		100.000

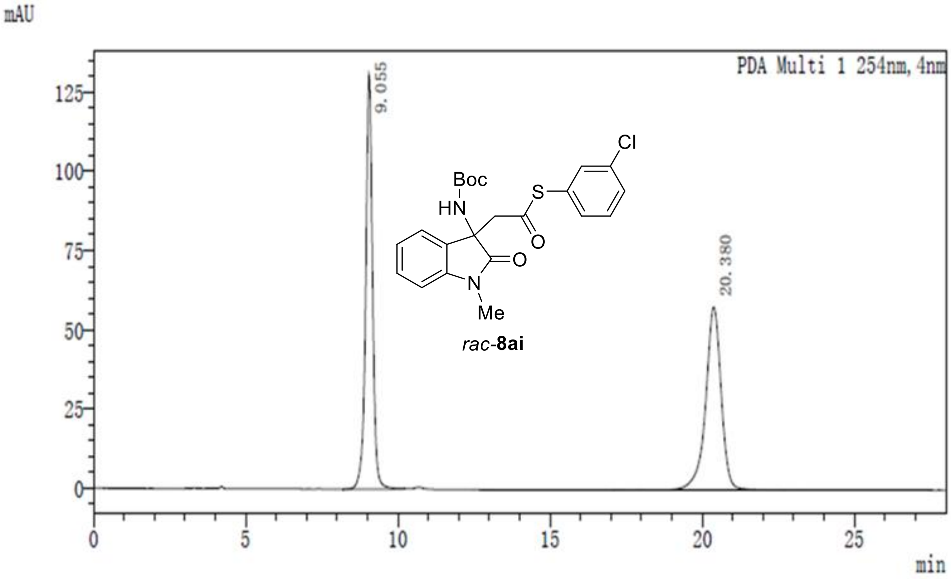

〈峰表〉

PDA Ch1 254nm

峰号	保留时间	面积	高度	浓度	面积%
1	9.055	2050309	130952	0.000	50.091
2	20.380	2042854	57702	0.000	49.909
总计		4093163	188654		100.000

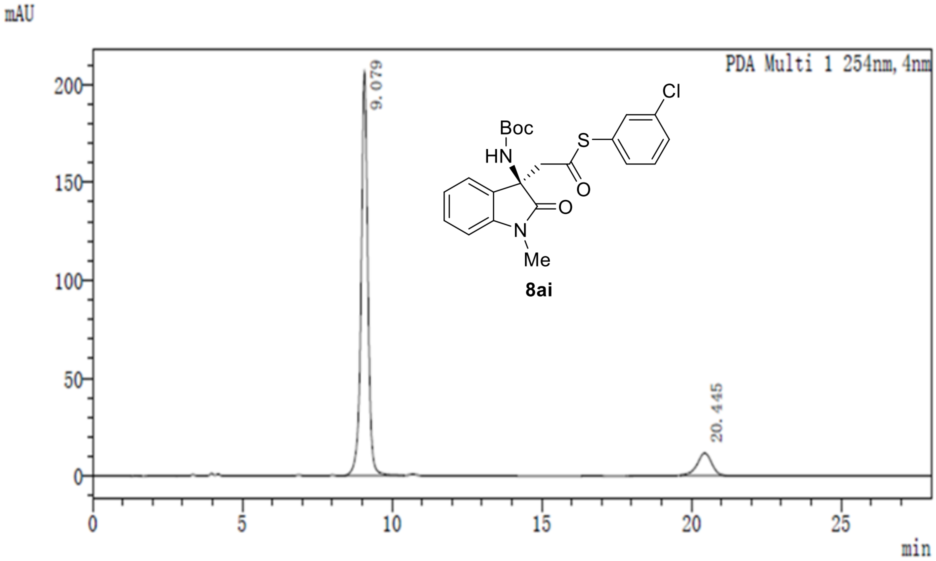

〈峰表〉

PDA Ch1 254nm

峰号	保留时间	面积	高度	浓度	面积%
1	9.079	3230588	206179	0.000	89.448
2	20.445	381102	11394	0.000	10.552
总计		3611690	217573		100.000

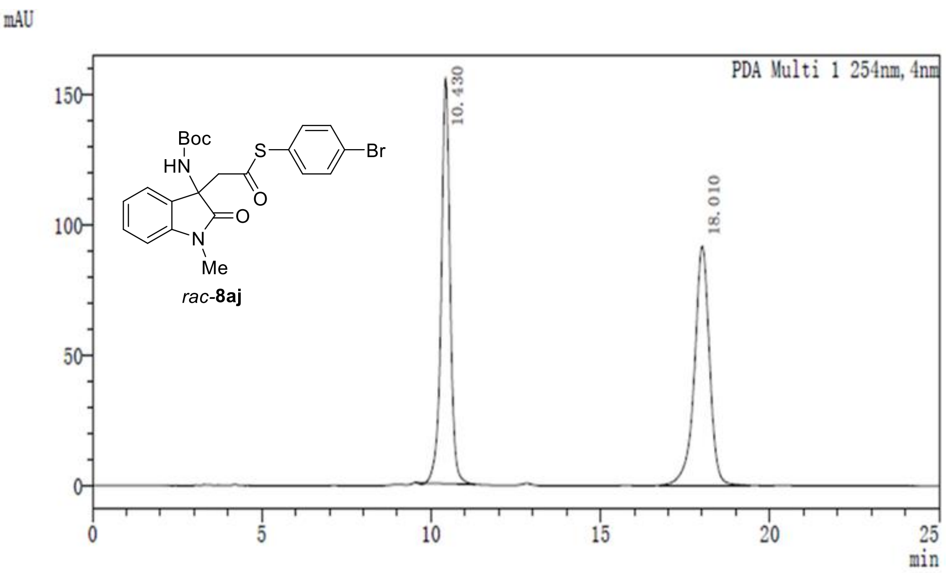

〈峰表〉

PDA Ch1 254nm

峰号	保留时间	面积	高度	浓度	面积%
1	10.430	2833404	155693	0.000	49.884
2	18.010	2846618	91851	0.000	50.116
总计		5680022	247544		100.000

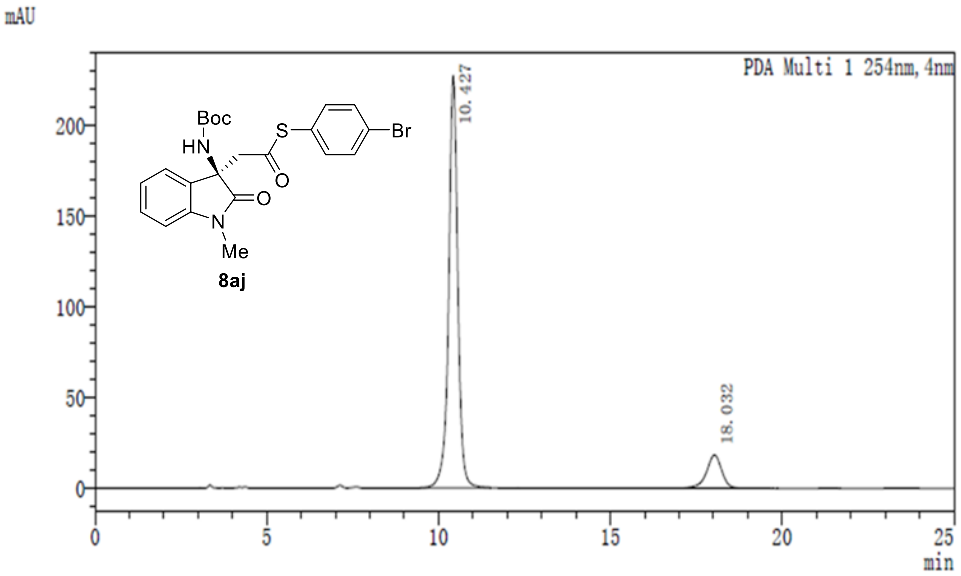

〈峰表〉

PDA Ch1 254nm

峰号	保留时间	面积	高度	浓度	面积%
1	10.427	4198095	227283	0.000	88.608
2	18.032	539738	18120	0.000	11.392
总计		4737833	245403		100.000

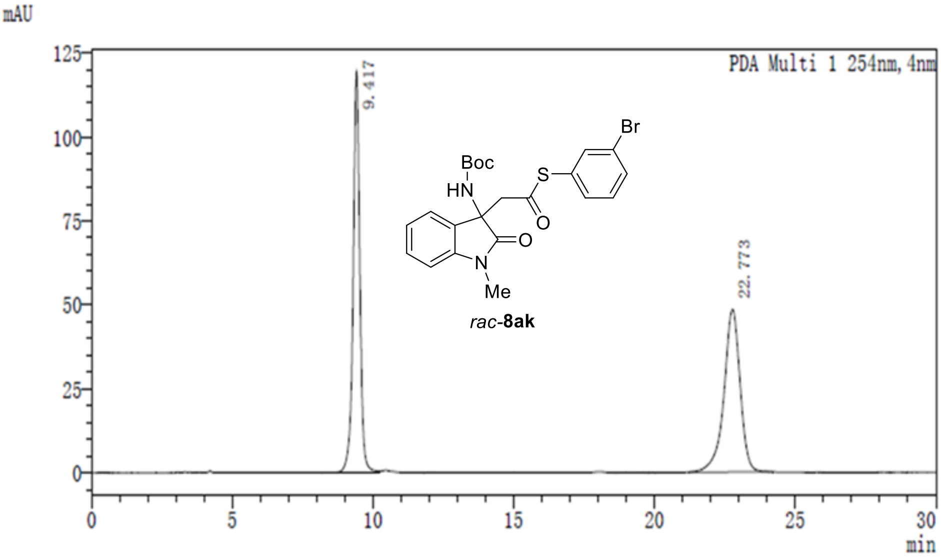

〈峰表〉
PDA Ch1 254nm

峰号	保留时间	面积	高度	浓度	面积%
1	9.417	1956010	119116	0.000	50.241
2	22.773	1937263	48415	0.000	49.759
总计		3893273	167531		100.000

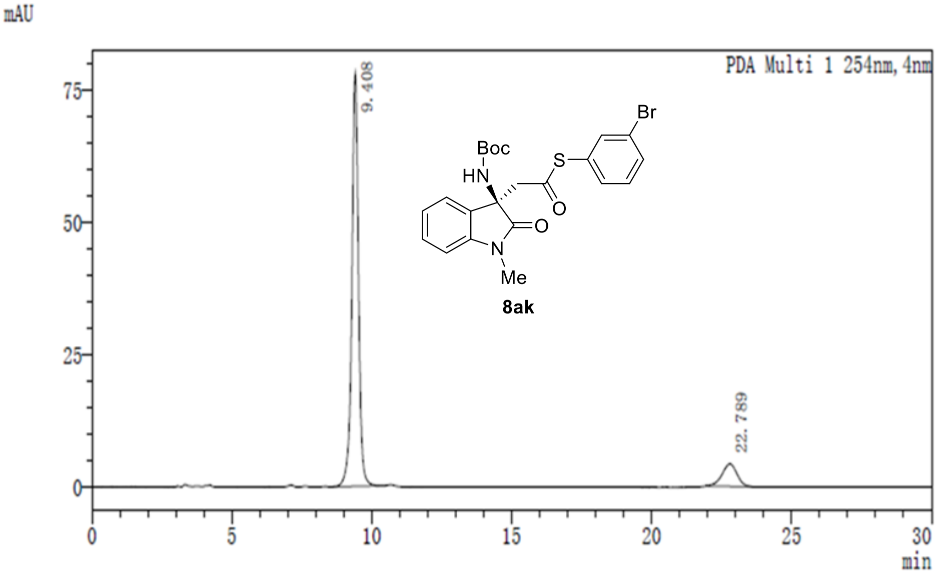

〈峰表〉
PDA Ch1 254nm

峰号	保留时间	面积	高度	浓度	面积%
1	9.408	1273730	77847	0.000	88.947
2	22.789	158286	4245	0.000	11.053
总计		1432016	82092		100.000

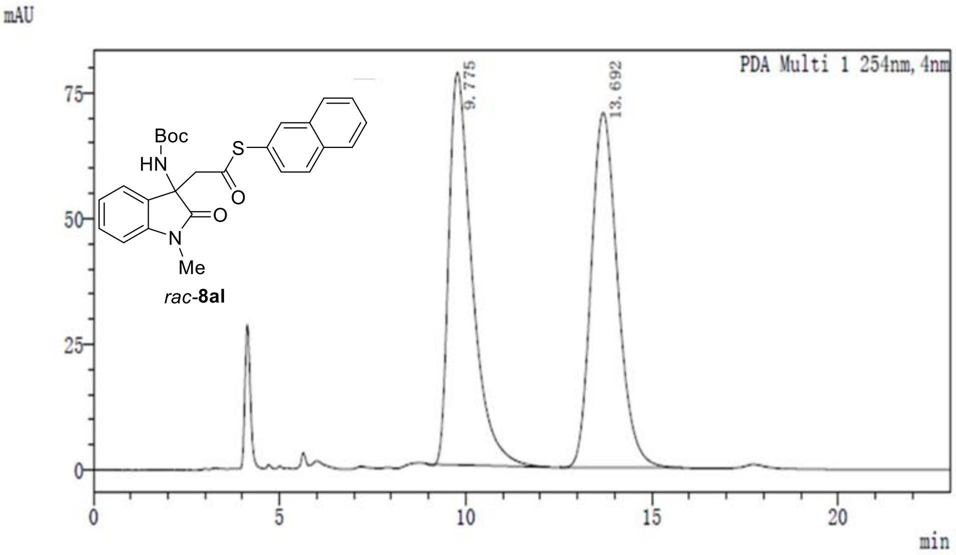

〈峰表〉

PDA Ch1 254nm

峰号	保留时间	面积	高度	浓度	面积%	峰结束
1	9.775	3396013	77965	0.000	49.284	12.224
2	13.692	3494711	70568	0.000	50.716	15.797
总计		6890724	148533		100.000	

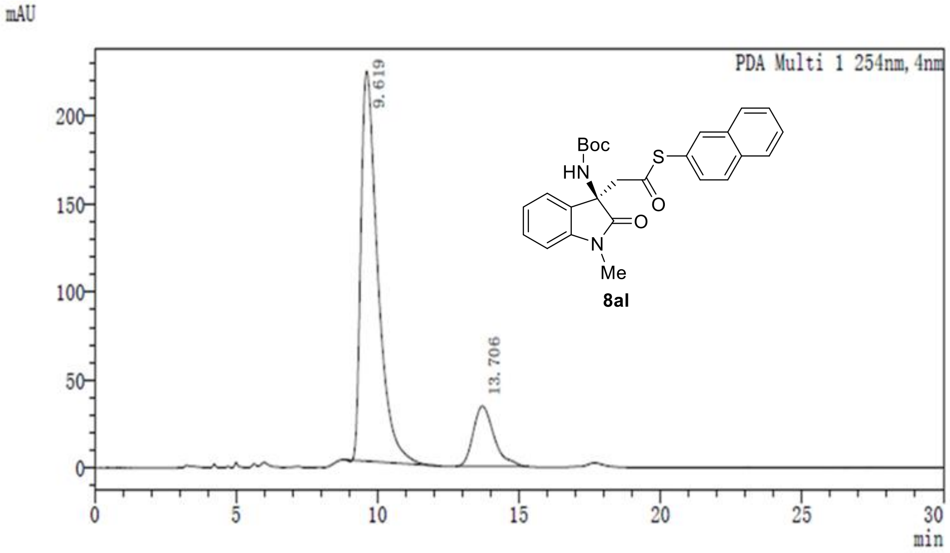

〈峰表〉

PDA Ch1 254nm

峰号	保留时间	面积	高度	浓度	面积%
1	9.619	9177315	221644	0.000	83.842
2	13.706	1768590	34265	0.000	16.158
总计		10945905	255909		100.000

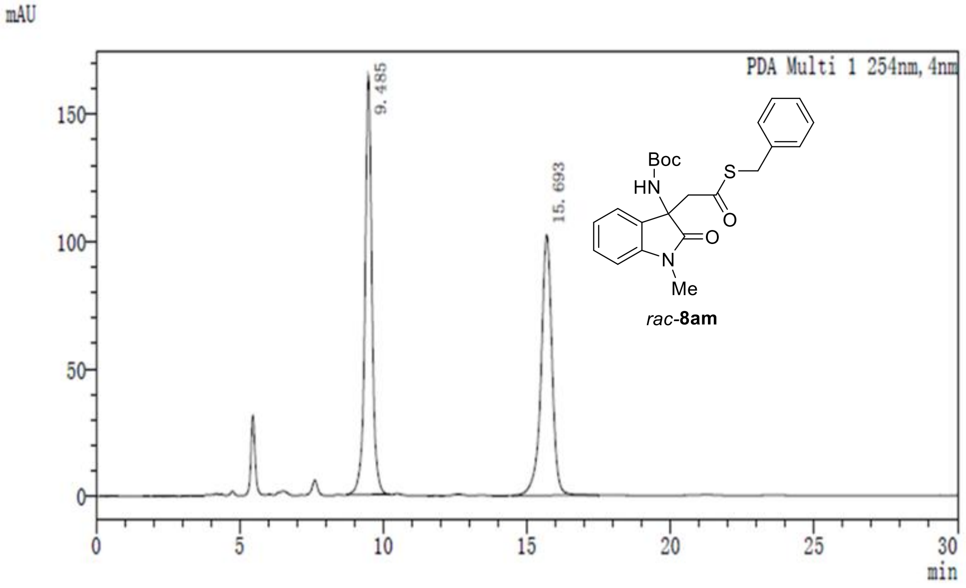

〈峰表〉
PDA Ch1 254nm

峰号	保留时间	面积	高度	浓度	面积%
1	9.485	2769211	164653	0.000	50.477
2	15.693	2716919	102384	0.000	49.523
总计		5486131	267037		100.000

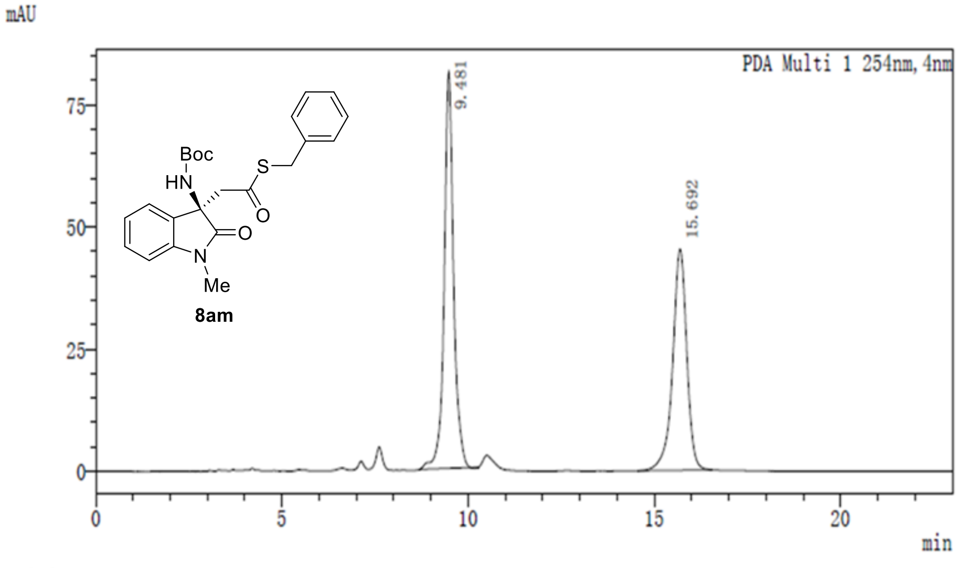

〈峰表〉
PDA Ch1 254nm

峰号	保留时间	面积	高度	浓度	面积%
1	9.481	1425507	81098	0.000	54.496
2	15.692	1190284	45268	0.000	45.504
总计		2615791	126366		100.000

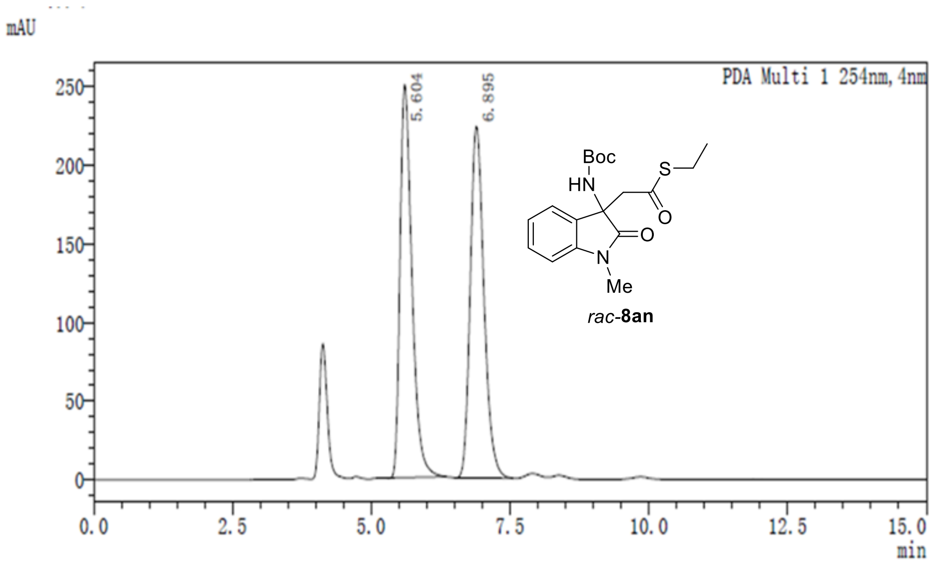

〈峰表〉

PDA Ch1 254nm

峰号	保留时间	面积	高度	浓度	面积%
1	5.604	3726912	249870	0.000	49.239
2	6.895	3842130	223694	0.000	50.761
总计		7569042	473564		100.000

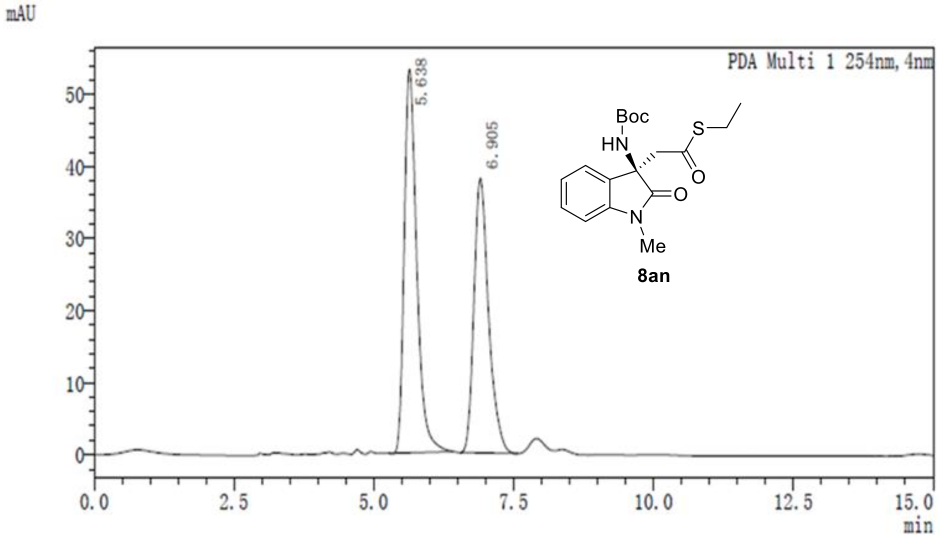

〈峰表〉

PDA Ch1 254nm

峰号	保留时间	面积	高度	浓度	面积%
1	5.638	824931	53208	0.000	54.270
2	6.905	695131	38122	0.000	45.730
总计		1520062	91330		100.000

6. References

[1]. Guo, H.; Zhang, L. W.; Zhou, H.; Meng, W.; Ao, Y. F.; Wang, D. X.; Wang, Q. Q. *Angew. Chem., Int. Ed.* **2020**, *59*, 2623.

[2]. Davies, S.; Mortlock, A. *Tetrahedron* **1993**, *49*, 4419.

[3]. Owsianik, K.; Wieczorek, W.; Bali´nska, A.; Mikołajczyk, M. *Heteroatom Chem*. **2014**, *25*, 690.

[4]. Chunhong, Z.; Liu, F.; Gou, S. Tetrahedron*: Asymmetry* **2014**, *25*, 278.

[5]. Mita, T.; Sugawara, M.; Saito, K. *Org. Lett*. **2014**, *16*, 3028.

[6]. Gould, A. E.; Adams, R.; Adhikari, S.; Aertgeerts, K.; Afroze, R.;Blackburn, C.; Calderwood, E. F.; Chau, R.; Chouitar, J.; Duffey, M. O.; England, D. B.; Farrer, C.; Forsyth, N.; Garcia, K.; Gaulin, J.; Greenspan, P. D.; Guo, R.; Harrison, S. J.; Huang, S.-C.; Iartchouk, N.; Janowick, D.; Kim, M.-S.; Kulkarni, B.; Langston, S. P.; Liu, J. X.; Ma, L.-T.; Menon, S.; Mizutani, H.; Paske, E.; Renou, C. C.; Rezaei, M.; Rowland, R. S.; Sintchak, M. D.; Smith, M. D.; Stroud, S. G.; Tregay, M.; Tian, Y.; Veiby, O. P.; Vos, T. J.; Vyskocil, S.; Williams, J.; Xu, T.; Yang, J. J.; Yano, J.; Zeng, H.; Zhang, D. M.; Zhang, Q.; Galvin, K. M. *J. Med. Chem*. **2011**, *54*, 1836.

[7]. a) Urban, M.; Franc, M.; Hofmanová, M.; Císařová, M.; Veselý, M. *Org. Biomol. Chem.* **2017**, *15*, 9071. b) Marques, C. S.; Burke, A. J. *Eur. J. Org. Chem*. **2016**, 806. c) Holmquist, M.; Blay, G.; Pedro, J. R. *Chem. Commun*. **2014**, *50*, 9309. d) Yan, W. J. W., D. Feng, J. C.; Li, P.; Zhao, D. P.; Wang, R. *Org. Lett*. **2012**, *14*, 2512.

[8]. Hara, N.; Nakamura, S.; Funahashi, Y.; Shibata, N. *Adv. Synth. Catal*. **2011**, *353*, 2976.

[9]. Hara, N.; Nakamura, S.; Sano, M.; Tamura, R.; Funahashi, Y.; Shibata, N. *Chem. Eur. J.* **2012**, *18*, 9276.
